# Safety profile of upadacitinib in inflammatory Bowel disease: a dual-source pharmacovigilance study integrating FAERS signal detection and real-world clinical cohort analysis

**DOI:** 10.3389/fphar.2026.1830935

**Published:** 2026-06-15

**Authors:** Wei Tan, Hao Wang, Hong Guo, Xiaomei Song, Wei Lei, Yan Liu, Lingya Xiang

**Affiliations:** Department of Gastroenterology, Chongqing Academy of Medical Sciences, Chongqing General Hospital, Chongqing University, Chongqing, China

**Keywords:** adverse events, inflammatory bowel disease, pharmacovigilance, therapeutic drug monitoring, upadacitinib

## Abstract

**Background:**

Upadacitinib, a selective Janus kinase 1 (JAK1) inhibitor, has demonstrated efficacy in inflammatory bowel disease (IBD). However, safety data in real-world Asian populations remain limited. This study aimed to characterize the adverse event (AE) profile of upadacitinib by integrating large-scale pharmacovigilance data with real-world clinical evidence.

**Methods:**

We conducted a dual-source analysis comprising spontaneous reports from the U.S. Food and Drug Administration Adverse Event Reporting System (FAERS; Q1 2018–Q3 2025) and a retrospective single-center clinical cohort in China (April 2023–September 2025). In the FAERS database, disproportionality analyses were performed using four algorithms (ROR, PRR, EBGM, and IC) to detect safety signals. In the clinical cohort, we assessed AE incidence, severity, timing, and management strategies. Subgroup analyses were descriptive, and Kaplan-Meier methods were used to estimate the time to the first AE.

**Results:**

The clinical cohort included 183 patients, of whom 85 (46.4%) experienced at least one AE. The most common events were laboratory abnormalities, infections, and dermatologic conditions. Serious events, including intestinal perforation and venous thrombosis, were rare. A distinct early clustering of toxicity was observed: 38.8% of first events occurred within 30 days and 67.0% within 60 days of treatment initiation. Male reproductive safety appeared favorable; one patient experienced transient semen discoloration, which resolved following dose reduction. The FAERS analysis identified rare signals absent in the clinical cohort, notably Pneumocystis jirovecii pneumonia (PJP) and pseudostroke, highlighting potential high-risk scenarios. Signal classification revealed strong signals for herpes zoster and acne, moderate signals for venous thrombosis and laboratory abnormalities, and weak signals for rare events. AE patterns varied by age, disease type, and induction dose, consistent with the mechanistic expectations of JAK1 inhibition.

**Conclusion:**

Upadacitinib demonstrates a manageable safety profile in patients with IBD, with most AEs controllable through vigilant monitoring and individualized management. The integration of clinical cohort data with FAERS pharmacovigilance provides a comprehensive view of both common and rare risks. These findings offer critical real-world evidence from an Asian population, complementing global data to inform clinical decision-making.

## Introduction

Inflammatory bowel disease (IBD), encompassing ulcerative colitis (UC) and Crohn’s disease (CD), represents a significant global health burden characterized by chronic gastrointestinal inflammation, immune dysregulation, and substantial morbidity (Kaplan, 2025). While biologic therapies have transformed management, many patients exhibit inadequate or waning responses, necessitating the development of novel therapeutic agents (Sandborn et al., 2020; Benucci et al., 2023; Danese et al., 2022; Loftus et al., 2023). Upadacitinib, a selective Janus kinase 1 (JAK1) inhibitor, has emerged as a potent oral treatment that modulates key cytokine pathways driving IBD pathogenesis (Sandborn et al., 2020; Danese et al., 2022). Following its initial approval for rheumatoid arthritis (European Medicines Agency, 2019), upadacitinib has been authorized for the treatment of moderate-to-severe UC and CD in both Western countries and China, demonstrating robust efficacy in inducing and maintaining clinical and endoscopic remission, even in biologic-refractory patients (Danese et al., 2022; Loftus et al., 2023).

Despite the established efficacy of upadacitinib, a comprehensive understanding of its safety profile in diverse, real-world settings remains incomplete. In women of childbearing age, recent pharmacovigilance data from VigiBase did not identify disproportionate reporting signals for spontaneous abortion or prematurity following JAK inhibitor exposure during pregnancy (Abolhassani et al., 2025). Moreover, no specific organ-related patterns emerged from individual case safety reports. Randomized controlled trials (RCTs), while foundational, often underrepresent specific populations and may fail to capture rare or long-term adverse events (AEs) (Ytterberg et al., 2022; Honap et al., 2024a). This limitation is particularly relevant given class-wide safety concerns associated with JAK inhibitors, including risks of major adverse cardiovascular events (MACE), malignancy, and thrombosis (Ytterberg et al., 2022). Furthermore, there is a distinct paucity of safety data regarding JAK inhibitors in Asian populations, as most large-scale RCTs have been conducted in Western cohorts (Kaplan, 2025; Honap et al., 2024a). Consequently, post-marketing pharmacovigilance is critical for identifying safety signals that may not emerge in controlled trial environments (Zheng et al., 2024; Al-Worafi, 2020; Bate and Evans, 2009).

To address these gaps, this study employs a dual-source methodology integrating data from the U.S. Food and Drug Administration Adverse Event Reporting System (FAERS) with a retrospective single-center clinical cohort in China. By combining large-scale signal detection with granular, real-world clinical phenotypes, we aim to identify both frequent and rare AEs associated with upadacitinib, delineate temporal patterns of toxicity, and provide actionable insights for risk stratification. This approach seeks to bridge the gap between global safety data and regional clinical practice, ultimately informing the safer use of upadacitinib in the management of IBD.

## Methods

### Study design and data sources

We conducted a dual-source safety evaluation of upadacitinib in inflammatory bowel disease (IBD). First, we analyzed spontaneous adverse event (AE) reports from the FDA Adverse Event Reporting System (FAERS) from Q1 2018 to Q3 2025 to detect pharmacovigilance signals. Second, we reviewed clinical records from Chongqing General Hospital (April 2023–September 2025) to capture real-world treatment outcomes and management strategies that registry data often do not record. This approach allowed detection of rare signals in FAERS while providing contextual assessment in routine clinical practice.

### FAERS data extraction

All reports for upadacitinib (Rinvoq®) were extracted from the FAERS public dashboard. Duplicates were removed following FDA guidelines, retaining the most recent report per case. IBD cases were identified using MedDRA-coded indications for Crohn’s disease, ulcerative colitis, or IBD. Reports missing age or sex were excluded from subgroup analyses but retained for overall signal detection.

### Clinical cohort

We included consecutive adults with Crohn’s disease or ulcerative colitis who received upadacitinib at our tertiary center. Patients with incomplete records or enrolled in interventional trials were excluded. Two gastroenterologists independently adjudicated each AE for causality, severity (CTCAE v5.0), timing, and management strategy (dose reduction, temporary interruption, or permanent discontinuation). For incidence analyses, only the first AE per patient was used to align with FAERS case-level assessment.

### Signal detection and classification

PT-level disproportionality was assessed using four established methods: reporting odds ratio (ROR), proportional reporting ratio (PRR), empirical Bayesian geometric mean (EBGM), and Bayesian confidence propagation neural network (BCPNN). Thresholds were defined as follows: ROR lower 95% confidence interval > 1; PRR ≥ 2 with χ^2^ ≥ 4; EB05 > 2; and IC025 > 0. A signal was considered present when at least two methods met their respective thresholds. Among the detected signals, strength was further classified as strong (identified by ≥ 3 methods) or moderate (identified by exactly two methods). Findings identified by only a single method were not considered confirmed signals and were treated as exploratory or suggestive associations. Estimates based on small counts or borderline metrics were interpreted with caution. These criteria are consistent with commonly applied pharmacovigilance practices, including those of the WHO Uppsala Monitoring Centre and relevant regulatory guidance.

### Statistical analysis in clinical cohort

Analyses were descriptive. Categorical variables are reported as counts and percentages; continuous variables as medians with interquartile ranges. Subgroup analyses by disease type and age were descriptive without formal hypothesis testing due to sample size imbalance. Kaplan-Meier methods were used to estimate time to first AE and time to treatment discontinuation.

### Ethics approval

The study was approved by the Ethics Committee of Chongqing General Hospital (IIT S2025-054-01) and conducted according to the Declaration of Helsinki. Written informed consent was waived due to the retrospective design and use of anonymized data.

## Results

Among 183 patients with IBD treated with upadacitinib, 85 (46.4%) experienced at least one adverse event during follow-up. This cohort had a high burden of prior treatment failure: all patients had previously received biologic therapy, and 58.8% had failed two or more agents, including anti-TNFs, vedolizumab, and ustekinumab. Most patients (63.5%) experienced a single AE, and among patients with AEs, 76.7% required intervention, including dose modification or treatment discontinuation. Baseline demographic characteristics, prior treatment exposure, and adverse event summaries are detailed in Table 1.

**TABLE 1:** Clinical cohort characteristics and adverse event summary (patients with ≥1 AE, n = 85).

Characteristic	Category	n (%)
Demographics	​	​
Sex	Female	37 (43.5%)
​	Male	48 (56.5%)
Age	<50 years	71 (83.5%)
​	≥50 years	14 (16.5%)
Disease	​	​
Indication	Crohn’s disease	48 (56.5%)
​	Ulcerative colitis	37 (43.5%)
Prior treatment exposure	​	​
Medication history^a^	Systemic steroids	32 (37.6%)
​	Immunomodulators^b^	25 (29.4%)
​	Biologics (any)	85 (100.0%)
​	Infliximab	70 (82.4%)^a^
​	Adalimumab	26 (30.6%)^a^
​	Vedolizumab	22 (25.9%)^a^
​	Ustekinumab	37 (43.5%)^a^
Number of prior biologics	1	25 (29.4%)
	2	50 (58.8%)
	3	10 (11.8%)
Adverse events	​	​
AEs per patient	1	54 (63.5%)
​	2	26 (30.6%)
​	3	5 (5.9%)
Management of AEs (event-level, n = 120)	Required intervention^c^	92 (76.7%)
	Hospitalization	14 (11.7%)

Percentages are calculated based on the number of patients unless otherwise specified.

^a^ Medication history refers to exposure prior to upadacitinib initiation. Percentages for specific biologic agents are calculated among patients with any biologic exposure (n = 85). Patients may have received multiple agents.

^b^ Includes thiopurines, methotrexate, and calcineurin inhibitors.

^c^ Includes dose adjustment, temporary interruption, or permanent discontinuation. Twelve patients discontinued treatment permanently due to 14 adverse events.

To provide broader context, we analyzed 8,824 upadacitinib-associated FAERS reports. Among reports with available reporting dates, 75% (n = 6,598) were submitted in 2024–2025, consistent with expanded post-marketing use following recent IBD indications. Detailed baseline characteristics of both the clinical cohort and FAERS reports are provided in Supplementary Table S3.

### Kinetics of adverse events: the early risk window

Time-to-event analyses demonstrated a non-uniform temporal distribution of adverse events following upadacitinib initiation, characterized by early clustering.

At the cohort level, the cumulative incidence of first adverse events was 18.0% at 30 days and increased to 32.0% by 60 days (Supplementary Table S4). Interval-specific event proportions, calculated using patients at risk at the start of each interval, declined over time, indicating a decreasing hazard after treatment initiation.

To further characterize temporal patterns among affected individuals, we examined patients who developed at least one adverse event (n = 85). Within this subgroup, 38.8% of first events occurred within 30 days and 67.0% within 60 days, demonstrating a marked early concentration of toxicity. These analyses are descriptive and do not imply causal relationships.

**FIGURE 1:**
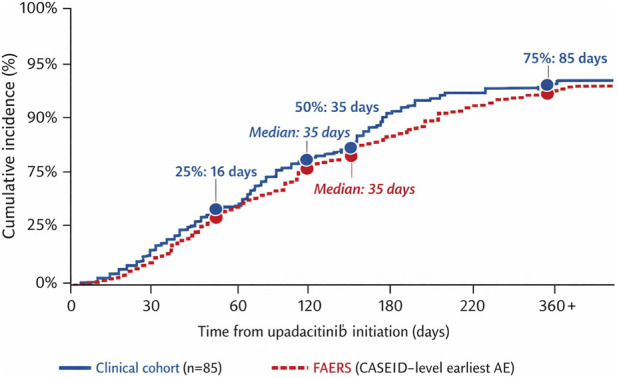
Illustrates the cumulative incidence of first AEs over time. Kaplan-Meier curve illustrating the cumulative probability of experiencing the first adverse event (AE) following upadacitinib initiation. The x-axis represents time in days, and the y-axis represents cumulative incidence. The steepest decline occurs within the first 60 days, indicating an early risk window.

These measures reflect complementary perspectives—absolute risk in the overall cohort *versus* temporal distribution among event-positive patients. Consistently, Kaplan-Meier analysis (Figure 1) showed a steep early increase in event probability followed by a plateau.

In the FAERS dataset, time-to-onset information was available for a subset of reports (n = 2,437), among which 36.1% of events occurred within the first 60 days (Supplementary Table S4). Interpretation is limited by substantial missingness in key variables, including time-to-onset (75.8%) and age (47.1%) (Supplementary Table S3). Accordingly, FAERS findings are considered supportive rather than confirmatory, but remain directionally consistent with the early clustering observed in the clinical cohort.

### SOC and PT-level signal analysis

Among patients with at least one adverse event (n = 85), events spanned multiple system organ classes (SOCs), with the most prominent being laboratory abnormalities (40.0%), infections (21.7%), dermatologic conditions (20.0%), and gastrointestinal disorders (6.7%) (Table 2). Most events were manageable with supportive care, dose modification, or temporary interruption, and relatively few required permanent discontinuation. The detailed preferred-term adverse events, CTCAE grades, management strategies, and clinical outcomes are presented in Table 3.

**TABLE 2:** Distribution of adverse events by dose at onset in the clinical cohort.

Dose at AE onset	AE events, n (%) (N = 120)	Patients with ≥1 AE at this dose, n (%) (N = 85)	Most frequent adverse events (preferred terms, n)
45 mg	86 (71.7%)	63 (74.1%)	Acne (15), white blood cell count decreased (9), folliculitis (8)
30 mg	30 (25.0%)	19 (22.4%)	Herpes zoster (5), herpes simplex (4), white blood cell count decreased (4)
15 mg	4 (3.3%)	3 (3.5%)	Various events (each n = 1), including upper respiratory tract infection, intestinal perforation, blood cholesterol increased, and expulsion of medication
Total	120 (100%)	85 (100%)	—

Adverse events were attributed to the upadacitinib dose at the time of onset and analyzed primarily at the event level (N = 120), allowing multiple events per patient. Therefore, individual patients may contribute to more than one dose category. The distribution of adverse events across dose groups may also reflect treatment phase (e.g., induction vs. maintenance) rather than a direct dose–response relationship.

**TABLE 3:** Common adverse events at the preferred term (PT) level in the clinical cohort.

System organ class/Preferred term	Events, n (%)	Grade 3–4, n (%)	Management strategy	Outcome
Infections and infestations	26 (21.7%)	3 (11.5%)	​	​
Herpes zoster	10 (8.3%)	0	Temporary D/C + antivirals	Resolved
Herpes simplex	8 (6.7%)	0	Topical treatment	Resolved
Upper respiratory tract infection	5 (4.2%)	0	Symptomatic treatment	Resolved
Clostridioides difficile infection	2 (1.7%)	2 (100%)	Temporary D/C + antibiotics	Resolved
Tuberculosis	1 (0.8%)	1 (100%)	Permanent D/C + Anti-TB therapy	Improved
Skin and subcutaneous tissue disorders	24 (20.0%)	0	​	​
Acne	15 (12.5%)	0	Topical treatment	Resolved/improved
Folliculitis	9 (7.5%)	0	Topical treatment	Resolved/improved
Gastrointestinal disorders	8 (6.7%)	2 (25.0%)	​	​
Intestinal perforation	2 (1.7%)	2 (100%)	Permanent D/C + surgical intervention	Improved
Constipation	3 (2.5%)	0	Symptomatic treatment	Improved
Nausea	3 (2.5%)	0	Symptomatic treatment	Resolved
Investigations	48 (40.0%)	5 (10.4%)	​	​
White blood cell count decreased	13 (10.8%)	1 (7.7%)	1 case with WBC <3.0 × 10^9^/L, permanent D/C; other symptomatic treatment	Resolved/improved
Neutrophil count decreased	9 (7.5%)	1 (11.1%)	1 case with N < 1.5 × 10^9^/L , permanent D/C; other symptomatic treatment	Resolved/improved
Lymphocyte count decreased	4 (3.3%)	0	Symptomatic treatment	Stable
Hemoglobin decreased	4 (3.3%)	3 (75.0%)	1 case iron supplementation; 3 cases with Hb < 70 g/L, blood transfusion + permanent D/C	Improved
Alanine aminotransferase increased	4 (3.3%)	0	Hepatoprotectives	Resolved
Aspartate aminotransferase increased	4 (3.3%)	0	Hepatoprotectives	Resolved
Blood bilirubin increased	1 (0.8%)	0	Monitoring	Stable
Blood cholesterol increased	6 (5.0%)	0	Statins	Improved/resolved
Blood creatine phosphokinase increased	3 (2.5%)	0	Monitoring	Stable
Reproductive system and breast disorders	4 (3.3%)	0	​	​
Amenorrhea	3 (2.5%)	0	No immediate intervention, dose reduced from 45 mg to 30 mg after induction	Resolved
Semen discoloration	1 (0.8%)	0	No immediate intervention, dose reduced from 45 mg to 15 mg after induction	Resolved
Injury, poisoning and procedural complications	4 (3.3%)	0	​	​
Expulsion of medication	4 (3.3%)	0	Dietary modification + antidiarrheals	Resolved
Musculoskeletal and connective tissue disorders	2 (1.7%)	0	​	​
Arthralgia	1 (0.8%)	0	Permanent D/C (patient preference)	Resolved
Myalgia	1 (0.8%)	0	Permanent D/C (patient preference)	Resolved
Vascular disorders	1 (0.8%)	1 (100%)	​	​
Venous thrombosis	1 (0.8%)	1 (100%)	Permanent D/C + anticoagulation	Improved
Eye disorders	1 (0.8%)	1 (100%)	​	​
Cataract	1 (0.8%)	1 (100%)	Permanent D/C + ophthalmologic follow-up	Stable
General disorders	2 (1.7%)	2 (100%)	​	​
Pyrexia	2 (1.7%)	2 (100%)	Permanent D/C + antipyretics	Resolved

Two cases of intestinal perforation were observed in patients with Crohn’s disease, occurring during both induction (45 mg) and maintenance (15 mg) therapy, and both required surgical intervention and treatment discontinuation. A single case of semen discoloration (gray-blue) with reduced sperm motility was observed during 45 mg dosing; no adjustment was made due to lack of fertility needs, but the discoloration resolved after dose reduction to 15 mg at the end of induction. Nausea was infrequent (2.5%) and responded to conservative management.

Laboratory abnormalities, particularly leukopenia and neutropenia, were among the most frequent events and occasionally required dose adjustment or temporary interruption. One patient developed venous thrombosis requiring anticoagulation and permanent discontinuation. Dermatologic events, including acne and folliculitis, were generally mild and manageable with topical therapy. Infections, including herpes zoster, were treated with antivirals and rarely led to discontinuation.

Comparison with FAERS demonstrated that key clinically relevant events showed concordance with signals identified through disproportionality analyses (Table 4). In contrast, rarer or less clinically apparent events were more frequently detected in FAERS, reflecting the complementary strengths of large-scale pharmacovigilance and real-world clinical observation.

**TABLE 4:** Preferred term-level signals detected in FAERS for upadacitinib-associated adverse events.

Preferred term	n	ROR (95% CI)	PRR (χ2)	EBGM (EBGM05)	IC (IC025)
Herpes zoster	125	2.72 (2.28–3.26)	2.72 (130.83)	2.65 (2.22)	1.41 (1.13)
Herpes simplex	9	3.37 (1.72–6.57)	3.37 (14.29)	3.26 (1.67)	1.70 (0.47)
*Clostridium difficile* infection	96	1.49 (1.21–1.82)	1.48 (14.90)	1.47 (1.20)	0.56 (0.26)
Acne	346	11.47 (10.24–12.86)	11.33 (2,818.84)	9.92 (8.85)	3.31 (3.11)
Folliculitis	28	2.29 (1.57–3.33)	2.28 (19.60)	2.24 (1.54)	1.17 (0.56)
Intestinal perforation	48	2.25 (1.68–2.99)	2.24 (32.11)	2.21 (1.65)	1.14 (0.69)
Constipation	142	1.39 (1.18–1.65)	1.39 (15.37)	1.38 (1.17)	0.47 (0.22)
White blood cell count decreased	28	2.39 (1.64–3.48)	2.39 (21.86)	2.34 (1.61)	1.23 (0.62)
Neutrophil count decreased	9	3.61 (1.85–7.05)	3.61 (16.17)	3.48 (1.78)	1.80 (0.54)
Lymphocyte count decreased	12	4.08 (2.28–7.31)	4.08 (26.43)	3.92 (2.19)	1.97 (0.85)
Haemoglobin decreased	111	2.49 (2.06–3.01)	2.48 (95.08)	2.43 (2.01)	1.28 (0.99)
Alanine aminotransferase increased	18	1.84 (1.15–2.94)	1.84 (6.71)	1.82 (1.14)	0.86 (0.13)
Aspartate aminotransferase increased	19	2.93 (1.85–4.63)	2.93 (23.17)	2.85 (1.80)	1.51 (0.72)
Blood cholesterol abnormal	9	7.79 (3.91–15.49)	7.78 (48.01)	7.12 (3.58)	2.83 (1.17)
Blood creatine phosphokinase increased	25	11.30 (7.41–17.23)	11.29 (202.58)	9.89 (6.49)	3.31 (2.27)
Amenorrhoea	15	7.33 (4.31–12.47)	7.33 (74.35)	6.74 (3.96)	2.75 (1.55)
Semen discolouration	36	862.69 (265.65–2,801.58)	861.47 (2,380.13)	67.19 (20.69)	6.07 (3.92)
Expulsion of medication	3	107.70 (17.99–644.57)	107.68 (126.83)	43.67 (7.30)	5.45 (0.02)
Venous thrombosis	4	2.84 (1.05–7.72)	2.84 (4.60)	2.77 (1.02)	1.47 (−0.30)
Cataract	69	2.65 (2.08–3.37)	2.64 (67.98)	2.58 (2.03)	1.37 (0.98)
Pyrexia	261	1.28 (1.13–1.45)	1.28 (15.44)	1.27 (1.12)	0.35 (0.16)

All listed preferred terms met positivity criteria in at least two of four algorithms (ROR, PRR, EBGM, BCPNN/IC). EBGM05 and IC025 represent lower bounds of 90% and 95% credibility intervals, respectively.

Overall, these findings indicate that while upadacitinib is associated with a spectrum of adverse events, most are clinically manageable, and serious events, although uncommon, require prompt recognition and individualized management strategies.

### Subgroup analysis

#### Age-stratified analysis

Among patients with ≥1 adverse event (n = 85), 83.5% were aged <50 years (Table 5). In this group, infections and dermatologic events were the most prominent, occurring in 30.6% and 28.2% of patients, respectively. Common infections included herpes zoster (11.8%), herpes simplex (9.4%), and upper respiratory tract infection (5.9%), while dermatologic events were mainly acne (17.6%) and folliculitis (10.6%). Reproductive events (amenorrhea: 3.5%; semen discoloration: 1.2%) were observed exclusively in this age group.

**TABLE 5:** Subgroup analysis of adverse events in the clinical cohort (descriptive analysis).

System organ class/Preferred term (PT)	Total patients n (%)	Age <50years n (%)	Age ≥50years n (%)	Crohn’s disease n (%)	Ulcerative colitis n (%)
Infections and infestations	26 (30.6%)	20 (23.5%)	6 (7.1%)	13 (15.3%)	13 (15.3%)
Herpes zoster	10 (11.8%)	8 (9.4%)	2 (2.4%)	4 (4.7%)	6 (7.1%)
Herpes simplex	8 (9.4%)	6 (7.1%)	2 (2.4%)	5 (5.9%)	3 (3.5%)
Upper respiratory tract infection	5 (5.9%)	4 (4.7%)	1 (1.2%)	3 (3.5%)	2 (2.4%)
Clostridioides difficile infection	2 (2.4%)	2 (2.4%)	0 (0.0%)	1 (1.2%)	1 (1.2%)
Tuberculosis	1 (1.2%)	0 (0.0%)	1 (1.2%)	0 (0.0%)	1 (1.2%)
Skin and subcutaneous tissue disorders	24 (28.2%)	20 (23.5%)	4 (4.7%)	12 (14.1%)	12 (14.1%)
Acne	15 (17.6%)	12 (14.1%)	3 (3.5%)	8 (9.4%)	7 (8.2%)
Folliculitis	9 (10.6%)	8 (9.4%)	1 (1.2%)	4 (4.7%)	5 (5.9%)
Gastrointestinal disorders	8 (9.4%)	6 (7.1%)	2 (2.4%)	6 (7.1%)	2 (2.4%)
Intestinal perforation	2 (2.4%)	2 (2.4%)	0 (0.0%)	2 (2.4%)	0 (0.0%)
Constipation	3 (3.5%)	2 (2.4%)	1 (1.2%)	2 (2.4%)	1 (1.2%)
Nausea	3 (3.5%)	2 (2.4%)	1 (1.2%)	2 (2.4%)	1 (1.2%)
Investigations	48 (56.5%)	39 (45.9%)	9 (10.6%)	28 (32.9%)	20 (23.5%)
White blood cell count decreased	13 (15.3%)	10 (11.8%)	3 (3.5%)	7 (8.2%)	6 (7.1%)
Neutrophil count decreased	9 (10.6%)	7 (8.2%)	2 (2.4%)	5 (5.9%)	4 (4.7%)
Lymphocyte count decreased	4 (4.7%)	3 (3.5%)	1 (1.2%)	2 (2.4%)	2 (2.4%)
Hemoglobin decreased	4 (4.7%)	4 (4.7%)	0 (0.0%)	3 (3.5%)	1 (1.2%)
Alanine aminotransferase increased	4 (4.7%)	4 (4.7%)	0 (0.0%)	2 (2.4%)	2 (2.4%)
Aspartate aminotransferase increased	4 (4.7%)	4 (4.7%)	0 (0.0%)	2 (2.4%)	2 (2.4%)
Blood bilirubin increased	1 (1.2%)	1 (1.2%)	0 (0.0%)	0 (0.0%)	1 (1.2%)
Blood cholesterol increased	6 (7.1%)	3 (3.5%)	3 (3.5%)	3 (3.5%)	3 (3.5%)
Blood creatine phosphokinase increased	3 (3.5%)	3 (3.5%)	0 (0.0%)	2 (2.4%)	1 (1.2%)
Reproductive system and breast disorders	4 (4.7%)	4 (4.7%)	0 (0.0%)	2 (2.4%)	2 (2.4%)
Amenorrhea	3 (3.5%)	3 (3.5%)	0 (0.0%)	2 (2.4%)	1 (1.2%)
Semen discoloration	1 (1.2%)	1 (1.2%)	0 (0.0%)	0 (0.0%)	1 (1.2%)
Musculoskeletal and connective tissue disorders	2 (2.4%)	2 (2.4%)	0 (0.0%)	2 (2.4%)	0 (0.0%)
Arthralgia	1 (1.2%)	1 (1.2%)	0 (0.0%)	1 (1.2%)	0 (0.0%)
Myalgia	1 (1.2%)	1 (1.2%)	0 (0.0%)	1 (1.2%)	0 (0.0%)
Vascular disorders	1 (1.2%)	0 (0.0%)	1 (1.2%)	1 (1.2%)	0 (0.0%)
Venous thrombosis	1 (1.2%)	0 (0.0%)	1 (1.2%)	1 (1.2%)	0 (0.0%)
Eye disorders	1 (1.2%)	0 (0.0%)	1 (1.2%)	1 (1.2%)	0 (0.0%)
Cataract	1 (1.2%)	0 (0.0%)	1 (1.2%)	1 (1.2%)	0 (0.0%)
General disorders	2 (2.4%)	2 (2.4%)	0 (0.0%)	1 (1.2%)	1 (1.2%)
Pyrexia	2 (2.4%)	2 (2.4%)	0 (0.0%)	1 (1.2%)	1 (1.2%)

Among patients aged ≥50 years (16.5%), metabolic and vascular events were more prominent, including increased blood cholesterol (3.5%), venous thrombosis (1.2%), and cataract (1.2%). Laboratory abnormalities occurred in both age groups but were more frequent in younger patients (<50 years: 45.9%; ≥50 years: 10.6%).

In FAERS (Supplementary Table S5), reported preferred terms (PTs) varied by age group. Among patients <50 years, the most frequently reported terms included colitis ulcerative (6.7%), Crohn’s disease (6.0%), drug ineffective (5.5%), abdominal pain (5.0%), and acne (3.9%). Among patients ≥50 years, the most frequent terms were diarrhoea (6.6%), fatigue (5.0%), colitis ulcerative (4.9%), arthralgia (4.7%), and drug ineffective (3.9%). Acne appeared among the most frequently reported terms in both groups, whereas cataract was reported only in older patients (1.5%). These findings should be interpreted within the context of spontaneous reporting data.

#### Indication-based analysis

Among patients with ≥1 adverse event (n = 85), those with Crohn’s disease (56.5%) more frequently exhibited gastrointestinal events, including intestinal perforation (2.4%), whereas patients with ulcerative colitis (43.5%) showed a higher proportion of laboratory abnormalities, including haemoglobin decrease (1.2%) and cholesterol increase (3.5%) (Table 5). Laboratory abnormalities were also common in Crohn’s disease (32.9%), particularly decreased white blood cell count (8.2%) and neutrophils (5.9%).

In FAERS (Supplementary Table S5), reported PTs differed by indication. Crohn’s disease reports more frequently included colitis ulcerative (10.6%), drug ineffective (5.9%), diarrhoea (4.5%), acne (4.5%), and fatigue (3.9%), whereas ulcerative colitis reports more commonly included Crohn’s disease (9.4%), product residue (4.9%), surgery (4.7%), diarrhoea (4.6%), and drug ineffective (4.5%). Surgery and treatment ineffectiveness were reported in both groups, while acne appeared somewhat more frequently in Crohn’s disease.

These subgroup analyses are descriptive and should be considered hypothesis-generating rather than indicative of causal relationships or comparative risk.

## Discussion

This study integrates FAERS pharmacovigilance with a real-world Asian cohort to characterize upadacitinib safety in IBD. By design, clinical adjudication captures manageable, frequently observed events with granular phenotypic detail, whereas disproportionality analyses detect rare signals that single-center cohorts lack statistical power to identify (Zheng et al., 2024; Al-Worafi, 2020; Bate and Evans, 2009). Given the underrepresentation of Asian populations in global registration trials (Kaplan, 2025), this dual-source approach offers risk data that are complementary rather than merely confirmatory.

Compared with the integrated Phase 2/3 safety analysis by Panaccione et al. (Panaccione et al., 2026), our cohort demonstrated broadly concordant AE frequencies. Variations in infection and thromboembolic rates likely reflect differences in prior immunosuppressive exposure, genetic background, and baseline disease activity—factors that controlled trials underrepresent (Ytterberg et al., 2022; Panaccione et al., 2026). These observations underscore that real-world Asian cohorts provide distinct risk profiles essential for contextualizing global safety data. Additionally, as a small-molecule agent, upadacitinib does not induce anti-drug antibodies (ADAs)—a limitation that frequently complicates long-term biologic therapy and contributes to secondary loss of response (European Medicines Agency, 2019). This mechanistic distinction confers an advantage for sustained immunosuppression, positioning upadacitinib as a favorable option for maintenance treatment in patients requiring durable disease control.

The reversible gray-blue semen discoloration observed in one patient during 45 mg therapy—resolving upon dose reduction—aligns with recent multicenter reports describing transient blue-green seminal changes that reverse with dose attenuation (Lee et al., 2026). Unlike filgotinib, which carries irreversible spermatotoxicity signals from rodent and canine studies and necessitated dedicated sperm trials, upadacitinib showed no impairment of mating or fertility in male rats at exposures exceeding human doses by over 30-fold (U.S. Food and Drug Administration, 2019). The discoloration likely reflects excretion of drug metabolites into seminal fluid rather than germ-cell injury, consistent with preserved sperm concentration, motility, and DNA integrity in reported cases (Lee et al., 2026). Nevertheless, the absence of pre- and post-event semen analysis in our patient precludes causal inference, and the lack of dedicated spermatogenesis trials equivalent to the MANTA program warrants cautious counseling for men attempting conception.

Although Pneumocystis jirovecii pneumonia (PJP) and pseudostroke were absent from our 183-patient cohort, FAERS signals (Li et al., 2025) and published case reports (Jones et al., 2021; Chin et al., 2024) validate their occurrence in upadacitinib-exposed patients. PJP, confirmed by bronchoalveolar lavage polymerase chain reaction (BAL-PCR) in both rheumatoid arthritis (RA) and IBD cases (Jones et al., 2021; Chin et al., 2024), typically arises within the first 2 months of therapy in the setting of high-dose induction, corticosteroid co-therapy, and lymphopenia—a temporal window that overlaps the early risk clustering identified here. Clinicians should maintain heightened suspicion for subacute dyspnea with ground-glass opacities in these high-risk contexts and pursue prompt diagnostic evaluation when clinical presentation is suggestive. Given the rarity of this signal and the absence of controlled evidence establishing net benefit for routine chemoprophylaxis, a risk-stratified approach emphasizing vigilant monitoring over universal prophylaxis is warranted at present (Li et al., 2025; Caldera et al., 2025). Pseudostroke, most commonly manifesting as reversible posterior encephalopathy syndrome (PRES), presents with acute headache, visual disturbance, and focal deficits without diffusion-weighted imaging (DWI)-restricted diffusion; while routine neurological screening is impractical given its rarity, prompt drug discontinuation and blood pressure control are curative (Ferreira-da-Silva et al., 2025). Both signals remain subject to pharmacovigilance reporting bias and should not be interpreted as confirmed incidence elevations above background IBD risk (Al-Worafi, 2020; Bate and Evans, 2009). Yet their biological plausibility—JAK-mediated immunosuppression for PJP and interferon-related endothelial disruption for PRES—supports proactive risk stratification over passive surveillance (Benucci et al., 2023; Gupta et al., 2023).

Several AEs observed in our cohort have plausible molecular bases. JAK1 inhibition impairs interleukin-6 (IL-6) and interferon signaling, compromising host defense and hematopoiesis while altering endothelial anticoagulant properties (Benucci et al., 2023; Wu et al., 2025). The single venous thrombosis event aligns with a recognized class effect: cytokine blockade promotes a proadhesive endothelial milieu that synergizes with IBD-related hypercoagulability, dehydration, and immobilization (Ytterberg et al., 2022; Ferreira-da-Silva et al., 2025; Chen et al., 2022). Concurrent metabolic perturbations, including dose-dependent elevations in low-density lipoprotein (LDL) cholesterol and triglycerides, further augment cardiovascular vulnerability, particularly in older patients with reduced cytochrome P450 3A4 (CYP3A4)-mediated clearance (Ferreira-da-Silva et al., 2025; Wu et al., 2025). The divergent age-specific patterns—infections and dermatologic events predominating in younger patients, metabolic and vascular complications in those aged ≥50 years—likely reflect the interplay between drug exposure, immunosenescence, and accumulated comorbidity rather than qualitatively distinct toxicities (Ytterberg et al., 2022; Panaccione et al., 2025; Honap et al., 2026).

These findings inform a tiered preventive strategy. Baseline evaluation should encompass latent infection screening (tuberculosis, hepatitis B), varicella zoster immune status, and cardiovascular risk profiling including blood pressure and fasting lipids (Caldera et al., 2025; Gordon et al., 2024; Moran et al., 2025). Complete blood counts and metabolic panels require close monitoring during induction, when drug exposure peaks and AEs cluster temporally (Panaccione et al., 2026; Panaccione et al., 2025). High-risk subgroups—those with prior thrombosis, multiple cardiovascular risk factors, or prolonged high-dose corticosteroid exposure—merit individualized risk-benefit counseling (Ytterberg et al., 2022; Ferreira-da-Silva et al., 2025; Honap et al., 2026). The availability of three distinct dosing regimens—45 mg daily for induction, 30 mg as an intermediate dose, and 15 mg for maintenance—renders therapeutic drug monitoring (TDM) essential for precision dosing in IBD. Postoperative alterations in gastrointestinal anatomy may impair drug absorption, while active inflammation can modulate hepatic and intestinal metabolism through phenoconversion, thereby altering upadacitinib disposition (Mohamed-Eslam et al., 2017). Recent evaluations have demonstrated the clinical utility of TDM for upadacitinib and other JAK inhibitors, establishing dose-response relationships that support individualized exposure targeting (Honap et al., 2024b; Tachet et al., 2025). Furthermore, the development and validation of multiplex HPLC-MS/MS assays enables reliable quantification of upadacitinib plasma concentrations, providing the analytical infrastructure necessary for routine TDM implementation (Tachet et al., 2023). Beyond clinical and demographic determinants, variability in adverse event susceptibility within this Asian cohort may partly reflect pharmacogenomic differences influencing upadacitinib exposure—notably CYP3A-mediated metabolism—and downstream JAK-STAT signaling pathways. Future studies integrating pharmacogenomic profiling with real-world pharmacovigilance and exposure data could refine individualized safety prediction.

The retrospective design and absence of systematically recorded disease activity indices, including Mayo score, Crohn’s Disease Endoscopic Index of Severity (CDEIS), C-reactive protein (CRP), and fecal calprotectin, preclude causal attribution of AEs to drug toxicity *versus* underlying disease severity. During induction, the concordance of maximal inflammation and highest dose creates unresolvable confounding. Moreover, the extreme imbalance in events across dose strata reflects treatment-phase allocation and shorter maintenance duration rather than a true dose–response relationship, rendering formal statistical comparison uninformative. Similarly, the small number of patients aged ≥50 years (n = 14) limits the precision of age-stratified estimates. These constraints underscore that the observed associations are temporal rather than etiologic. Accordingly, these findings should be interpreted as hypothesis-generating rather than confirmatory evidence of risk.

Upadacitinib exhibits a manageable safety profile in biologic-experienced Asian patients with IBD, with most AEs amenable to dose modification or temporary interruption. Integration of clinical cohort data with FAERS pharmacovigilance provides a comprehensive framework for anticipating common, rare, and mechanism-based toxicities. Prospective multicenter studies incorporating standardized disease activity metrics and pharmacokinetic monitoring are needed to refine risk stratification and establish causality where current observational data cannot.

## Conclusion

Upadacitinib demonstrates a manageable safety profile in patients with inflammatory bowel disease, with most adverse events identifiable and controllable through appropriate monitoring and individualized management strategies. Integration of clinical cohort data with FAERS pharmacovigilance highlights both common and rare adverse events, while emphasizing population-specific factors, age- and disease-specific risk patterns, and preventive measures.

These findings should be interpreted within the broader context of JAK inhibitor class effects, as shared immunomodulatory mechanisms contribute to overlapping safety profiles across agents, while differences in selectivity and pharmacokinetics may modulate specific risks. The study provides real-world evidence from an underrepresented Asian population, complementing global data and informing clinical decision-making. Prospective, multi-center studies are needed to further confirm these observations and refine strategies for optimizing safety in IBD patients receiving JAK inhibitors.

## Data Availability

The original contributions presented in the study are included in the article/Supplementary Material, further inquiries can be directed to the corresponding authors.
