# Supplementary material for: Safety profile of upadacitinib in inflammatory Bowel disease: a dual-source pharmacovigilance study integrating FAERS signal detection and real-world clinical cohort analysis
Source: Front Pharmacol. 2026 Jun 15;17:1830935. doi: 10.3389/fphar.2026.1830935 (PMC13310892; doi:10.3389/fphar.2026.1830935)
Supplement: Supplementary file 1 [file DataSheet1.pdf]

Supplementary Data

Table 1S. 2 × 2 contingency table of disproportionality method

Item	Target adverse events reported	Other adverse events reported	Total
Reports with the target drug	a	b	a+b
All other drugs	c	d	c+d
Total	a+c	b+d	a+b+c+d

Note: a, b, c, d, indicated the number of reports that meet the specified conditions in the table.

Table 2S. Principles of Disproportionality Analysis and Signal Detection Criteria

Method	Calculation Principle	Signal Detection Criteria
ROR (Reporting Odds Ratio)	b/da/c	a ≥ 3; ROR > 1; 95%CI lower bound > 1
PRR (Proportional Reporting Ratio)	c/(c+d)a/(a+b)	a ≥ 3; PRR ≥ 2; χ^2 ≥ 4; 95%CI lower bound > 1
MGPS (Multi-item Gamma Poisson Shrinker) / EBGM	Empirical Bayes geometric mean	$EBGM_{05}$ > 2 (lower bound of 90% credibility interval)
BCPNN (Bayesian Confidence Propagation Neural Network) / IC	Bayesian information component	IC_{025} > 0 (lower bound of 95% credibility interval)

Abbreviations: a, target adverse events with target drug; b, other adverse events with target drug; c, target adverse events with all other drugs; d, other adverse events with all other drugs; CI, confidence interval; $EBGM_{05}$, lower bound of 90% credibility interval for EBGM; IC_{025}, lower bound of 95% credibility interval for IC; χ^2, chi-squared statistic.

Table 3S. Baseline Characteristics of Spontaneous Reports for Upadacitinib-Associated Adverse Events (FAERS, 2018–2025)

Characteristic	Category	n (%)
Total reports		8,824
Sex	Female	4,170 (47.3)
	Male	3,821 (43.3)
	Missing	833 (9.4)
Age	<50 years	2,415 (27.4)
	≥50 years	2,275 (25.8)
	Missing	4,158 (47.1)
Indication	Ulcerative colitis	4,108 (46.6)
	Crohn's disease	4,635 (52.5)
	Missing	104 (1.2)
Reporting period	2018–2023	2,226 (25.2)
	2024–2025	6,598 (74.8)
Reporter country	United States	6,222 (70.5)
	Other	2,602 (29.5)
Reporter type	Consumer	5,811 (65.9)

	Physician	1,630 (18.5)
	Other health professional	1,114 (12.6)
	Pharmacist	169 (1.9)
	Missing	100 (1.1)
Primary outcome	Death	218 (2.5)
	Life-threatening	76 (0.9)
	Hospitalization	2,874 (32.6)
	Disability	45 (0.5)
	Required intervention	17 (0.2)
	Congenital anomaly	2 (<0.1)
	Other	4,485 (50.8)
	Missing	1,107 (12.5)
Time to onset§	0–30 days	610 (6.9)
	31–60 days	271 (3.1)
	61–90 days	201 (2.3)
	91–180 days	343 (3.9)
	>180 days	712 (8.1)
	Missing	6,687 (75.8)

§Time from drug initiation to event onset. High missing rate reflects limitations of spontaneous reporting where onset dates are often incomplete.

Table 4S. Temporal Distribution of Adverse Events During Follow-up.

Time Interval	AE Events (n)	Patients under follow-up (n)	Interval event proportion (%)	Cumulative proportion (%)
0–30 days	33	183	18.0%	18.0%
31–60 days	24	141	17.0%	32.0%
61–90 days	14	105	13.3%	41.0%
91–180 days	12	82	14.6%	49.7%
>180 days	2	64	3.1%	51.2%

Patients at risk: Number of patients who had not experienced an AE and remained under observation at the start of each interval.

Interval event proportions were calculated using the number of patients at risk at the start of each time interval as the denominator.

Cumulative proportions were calculated using the total cohort (n=183) as the denominator.

The number of patients at risk at each time interval is provided to contextualize cumulative incidence estimates.

Table 5S. PT-level signals detected by two or more disproportionality methods (strong and moderate signals) in FAERS reports of upadacitinib-associated adverse events (n=444)。

SOC	PT	n	ROR (95% CI)	PRR(χ^2)	EBGM	IC(IC025)

					(EBGM05)	
Surgical and medical procedures	SURGERY	325	6.89 (6.15 - 7.73)	6.82 (1476.59)	6.31 (5.63)	2.66 (2.47)
	COLECTOMY	187	4.63 (3.99 - 5.37)	4.60 (495.81)	4.38 (3.78)	2.13 (1.89)
	ILEOSTOMY	154	8.57 (7.25 - 10.13)	8.52 (914.93)	7.73 (6.53)	2.95 (2.64)
	HOSPITALISATION	75	1.33 (1.06 - 1.67)	1.33 (6.09)	1.33 (1.05)	0.41 (0.07)
	INTESTINAL RESECTION	72	1.41 (1.12 - 1.78)	1.41 (8.45)	1.40 (1.11)	0.49 (0.14)
	STOMA CREATION	71	15.39 (11.91 - 19.90)	15.35 (784.99)	12.82 (9.92)	3.68 (3.09)
	OSTOMY BAG PLACEMENT	67	31.31 (23.50 - 41.73)	31.23 (1366.49)	22.07 (16.56)	4.46 (3.67)
	INTESTINAL OPERATION	40	4.44 (3.23 - 6.12)	4.44 (100.38)	4.24 (3.08)	2.08 (1.51)
	KNEE ARTHROPLASTY	36	3.40 (2.44 - 4.76)	3.40 (58.27)	3.29 (2.36)	1.72 (1.14)
	SPINAL OPERATION	36	9.08 (6.42 - 12.84)	9.07 (229.48)	8.16 (5.77)	3.03 (2.27)
	KNEE OPERATION	35	8.47 (5.97 - 12.03)	8.46 (206.00)	7.67 (5.40)	2.94 (2.18)
	COLOSTOMY	31	3.67 (2.56 - 5.27)	3.67 (57.21)	3.54 (2.46)	1.82 (1.19)
	HIP ARTHROPLASTY	29	3.28 (2.26 - 4.76)	3.28 (43.93)	3.18 (2.19)	1.67 (1.03)
	THERAPY INTERRUPTED	27	1.78 (1.22 - 2.61)	1.78 (9.07)	1.76 (1.20)	0.82 (0.23)
	SMALL INTESTINAL RESECTION	27	2.95 (2.01 - 4.34)	2.95 (33.45)	2.87 (1.95)	1.52 (0.87)
	COLECTOMY TOTAL	24	4.78 (3.16 - 7.22)	4.77 (67.13)	4.54 (3.00)	2.18 (1.39)
	HIP SURGERY	24	9.63 (6.29 - 14.75)	9.63 (163.59)	8.61 (5.62)	3.11 (2.11)
	THERAPY CESSATION	23	2.58 (1.70 - 3.91)	2.58 (21.41)	2.52 (1.66)	1.33 (0.64)
	CHOLECYSTECTOMY	23	2.55 (1.68 - 3.86)	2.55 (20.90)	2.50 (1.65)	1.32 (0.63)

INTESTINAL ANASTOMOSIS	22	4.38 (2.85 - 6.73)	4.37 (54.00)	4.18 (2.72)	2.06 (1.25)
TRANSFUSION	21	5.69 (3.65 - 8.88)	5.69 (75.21)	5.34 (3.43)	2.42 (1.52)
APPENDICECTOMY	21	3.41 (2.20 - 5.29)	3.41 (34.17)	3.30 (2.13)	1.72 (0.95)
COLON OPERATION	19	5.71 (3.58 - 9.11)	5.71 (68.34)	5.36 (3.36)	2.42 (1.46)
SHOULDER OPERATION	18	8.68 (5.32 - 14.15)	8.67 (109.02)	7.85 (4.81)	2.97 (1.82)
HYSTERECTOMY	17	2.60 (1.60 - 4.22)	2.60 (16.19)	2.55 (1.57)	1.35 (0.53)
ABORTION INDUCED	17	6.14 (3.74 - 10.07)	6.13 (67.29)	5.73 (3.49)	2.52 (1.47)
MEDICAL PROCEDURE	14	11.97 (6.80 - 21.08)	11.96 (120.59)	10.40 (5.90)	3.38 (1.87)
ILEOCAECAL RESECTION	14	4.43 (2.58 - 7.60)	4.43 (34.99)	4.23 (2.46)	2.08 (1.03)
MEDICAL DEVICE IMPLANTATION	13	12.79 (7.09 - 23.08)	12.78 (119.87)	11.00 (6.10)	3.46 (1.84)
PROCTECTOMY	12	6.84 (3.78 - 12.37)	6.84 (54.60)	6.33 (3.50)	2.66 (1.32)
THERAPY CHANGE	11	6.08 (3.28 - 11.25)	6.07 (43.00)	5.68 (3.07)	2.51 (1.16)
FOOT OPERATION	11	7.05 (3.80 - 13.10)	7.05 (52.01)	6.51 (3.50)	2.70 (1.28)
STOMA CLOSURE	10	5.09 (2.68 - 9.67)	5.09 (30.70)	4.82 (2.54)	2.27 (0.93)
TONSILLECTOMY	9	5.82 (2.95 - 11.49)	5.82 (33.24)	5.46 (2.77)	2.45 (0.96)
STENT PLACEMENT	9	6.04 (3.06 - 11.93)	6.04 (34.90)	5.65 (2.86)	2.50 (0.99)
DENTAL OPERATION	9	5.39 (2.74 - 10.60)	5.38 (29.89)	5.08 (2.58)	2.34 (0.90)
LIVER TRANSPLANT	9	7.26 (3.66 - 14.42)	7.26 (44.12)	6.68 (3.37)	2.74 (1.13)
SPINAL FUSION SURGERY	8	4.75 (2.32 - 9.71)	4.75 (22.19)	4.51 (2.21)	2.17 (0.70)
REHABILITATION THERAPY	8	6.31 (3.06 - 13.01)	6.31 (32.87)	5.88 (2.85)	2.56 (0.92)

ABDOMINAL OPERATION	8	3.32 (1.63 - 6.75)	3.32 (12.40)	3.22 (1.58)	1.69 (0.38)
GASTROINTESTINAL SURGERY	8	4.35 (2.13 - 8.89)	4.35 (19.47)	4.16 (2.04)	2.06 (0.62)
PROCTOCOLECTOMY	8	2.65 (1.31 - 5.36)	2.65 (7.91)	2.59 (1.28)	1.37 (0.15)
SHOULDER ARTHROPLASTY	7	7.08 (3.26 - 15.39)	7.08 (33.26)	6.53 (3.00)	2.71 (0.87)
LIMB OPERATION	7	4.41 (2.06 - 9.46)	4.41 (17.38)	4.21 (1.96)	2.07 (0.53)
WISDOM TEETH REMOVAL	7	2.72 (1.28 - 5.78)	2.72 (7.32)	2.65 (1.25)	1.41 (0.09)
GALLBLADDER OPERATION	7	2.81 (1.32 - 5.98)	2.81 (7.84)	2.74 (1.29)	1.45 (0.12)
CARDIAC OPERATION	7	3.54 (1.66 - 7.56)	3.54 (12.15)	3.42 (1.60)	1.77 (0.34)
RADIOTHERAPY	5	14.96 (5.71 - 39.21)	14.96 (53.89)	12.55 (4.79)	3.65 (0.80)
COLOSTOMY CLOSURE	5	2.62 (1.07 - 6.40)	2.62 (4.83)	2.56 (1.05)	1.36 (-0.19)
EYE OPERATION	5	3.49 (1.42 - 8.55)	3.48 (8.45)	3.37 (1.37)	1.75 (0.05)
ORAL SURGERY	5	5.79 (2.33 - 14.40)	5.79 (18.33)	5.43 (2.18)	2.44 (0.40)
DRAIN PLACEMENT	5	3.99 (1.62 - 9.82)	3.99 (10.61)	3.83 (1.56)	1.94 (0.16)
ANKLE OPERATION	5	9.97 (3.91 - 25.42)	9.97 (35.43)	8.88 (3.48)	3.15 (0.67)
MINERAL SUPPLEMENTATION	5	4.85 (1.96 - 12.00)	4.85 (14.32)	4.61 (1.86)	2.20 (0.29)
METABOLIC SURGERY	4	7.98 (2.84 - 22.42)	7.98 (21.97)	7.28 (2.59)	2.86 (0.31)
INFUSION	4	10.64 (3.72 - 30.40)	10.64 (30.41)	9.39 (3.29)	3.23 (0.41)
DENTAL IMPLANTATION	4	3.38 (1.24 - 9.21)	3.38 (6.40)	3.27 (1.20)	1.71 (-0.17)
ILEOCOLECTOMY	4	4.10 (1.50 - 11.24)	4.10 (8.88)	3.93 (1.44)	1.98 (-0.03)
INTERVERTEB	4	6.53 (2.35 -	6.53	6.07	2.60

	RAL DISC OPERATION		18.17)	(17.16)	(2.18)	(0.22)
	TENOPLASTY	3	16.57 (4.72 - 58.15)	16.57 (35.65)	13.65 (3.89)	3.77 (0.09)
	POLYPECTOMY	3	5.38 (1.67 - 17.41)	5.38 (9.96)	5.08 (1.57)	2.34 (-0.19)
	MASTECTOMY	3	4.31 (1.34 - 13.81)	4.31 (7.19)	4.12 (1.29)	2.04 (-0.30)
	MEDICAL DEVICE REMOVAL	3	5.52 (1.71 - 17.87)	5.52 (10.32)	5.20 (1.61)	2.38 (-0.18)
	LARGE INTESTINE OPERATION	3	4.49 (1.40 - 14.41)	4.49 (7.65)	4.28 (1.33)	2.10 (-0.27)
	BLADDER OPERATION	3	4.40 (1.37 - 14.10)	4.40 (7.41)	4.20 (1.31)	2.07 (-0.29)
	NECK SURGERY	3	3.92 (1.23 - 12.52)	3.92 (6.18)	3.76 (1.18)	1.91 (-0.35)
	INTRA-UTERINE CONTRACEPTIVE DEVICE INSERTION	3	35.90 (8.98 - 143.55)	35.89 (67.85)	24.26 (6.07)	4.60 (0.11)
	THERAPEUTIC PROCEDURE	3	6.33 (1.95 - 20.63)	6.33 (12.38)	5.90 (1.81)	2.56 (-0.12)
	ROTATOR CUFF REPAIR	3	6.33 (1.95 - 20.63)	6.33 (12.38)	5.90 (1.81)	2.56 (-0.12)
	EMERGENCY CARE	3	7.18 (2.19 - 23.53)	7.18 (14.50)	6.62 (2.02)	2.73 (-0.08)
	HOSPICE CARE	3	5.25 (1.63 - 16.97)	5.25 (9.63)	4.96 (1.54)	2.31 (-0.20)
	DENTAL CARE	3	4.40 (1.37 - 14.10)	4.40 (7.41)	4.20 (1.31)	2.07 (-0.29)
	DENTAL PROSTHESIS PLACEMENT	3	26.92 (7.14 - 101.49)	26.92 (54.45)	19.85 (5.27)	4.31 (0.11)
	PHARYNGEAL OPERATION	3	15.39 (4.42 - 53.54)	15.38 (33.23)	12.85 (3.69)	3.68 (0.09)
Infections and infestations	HERPES ZOSTER	125	2.72 (2.28 - 3.26)	2.72 (130.83)	2.65 (2.22)	1.41 (1.13)
	INFECTION	113	1.42 (1.18 - 1.72)	1.42 (13.93)	1.41 (1.17)	0.50 (0.22)
	URINARY	106	1.41 (1.16 -	1.41	1.40	0.49

TRACT INFECTION		1.71)	(12.34)	(1.15)	(0.20)
SEPSIS	102	2.48 (2.04 - 3.03)	2.48 (86.93)	2.43 (1.99)	1.28 (0.97)
CLOSTRIDIUM DIFFICILE INFECTION	96	1.49 (1.21 - 1.82)	1.48 (14.90)	1.47 (1.20)	0.56 (0.26)
ORAL HERPES	39	3.17 (2.30 - 4.38)	3.17 (55.54)	3.08 (2.23)	1.62 (1.08)
VIRAL INFECTION	37	1.74 (1.26 - 2.41)	1.74 (11.37)	1.72 (1.24)	0.78 (0.28)
APPENDICITIS	35	3.47 (2.47 - 4.88)	3.47 (58.74)	3.36 (2.39)	1.75 (1.16)
DIVERTICULITIS	33	2.07 (1.46 - 2.92)	2.07 (17.68)	2.04 (1.44)	1.03 (0.48)
CYTOMEGALOVIRUS INFECTION	28	1.99 (1.37 - 2.90)	1.99 (13.39)	1.96 (1.35)	0.97 (0.38)
FOLLICULITIS	28	2.29 (1.57 - 3.33)	2.28 (19.60)	2.24 (1.54)	1.17 (0.56)
ESCHERICHIA INFECTION	21	4.11 (2.65 - 6.38)	4.11 (46.71)	3.94 (2.54)	1.98 (1.16)
NOROVIRUS INFECTION	21	16.95 (10.54 - 27.28)	16.94 (254.85)	13.90 (8.64)	3.80 (2.45)
PNEUMOCYSTIS JIROVECII PNEUMONIA	19	5.17 (3.24 - 8.24)	5.17 (59.57)	4.89 (3.07)	2.29 (1.36)
LARGE INTESTINE INFECTION	16	7.41 (4.43 - 12.41)	7.41 (80.43)	6.81 (4.07)	2.77 (1.60)
APPENDICITIS PERFORATED	14	6.53 (3.78 - 11.29)	6.53 (60.06)	6.07 (3.51)	2.60 (1.40)
GASTRIC INFECTION	12	3.26 (1.83 - 5.82)	3.26 (18.02)	3.16 (1.77)	1.66 (0.62)
CYTOMEGALOVIRUS COLITIS	12	2.17 (1.22 - 3.86)	2.17 (7.35)	2.14 (1.20)	1.09 (0.16)
HERPES VIRUS INFECTION	11	2.88 (1.58 - 5.27)	2.88 (13.00)	2.81 (1.54)	1.49 (0.43)
MENINGITIS	11	3.54 (1.93 - 6.49)	3.54 (19.12)	3.42 (1.87)	1.77 (0.65)
OPHTHALMIC HERPES	10	4.95 (2.61 - 9.40)	4.95 (29.50)	4.70 (2.47)	2.23 (0.91)

	ZOSTER					
	RESPIRATORY SYNCYTIAL VIRUS INFECTION	10	4.49 (2.37 - 8.50)	4.49 (25.51)	4.28 (2.26)	2.10 (0.82)
	INFECTED FISTULA	9	2.05 (1.06 - 3.98)	2.05 (4.71)	2.02 (1.04)	1.02 (-0.05)
	HERPES SIMPLEX	9	3.37 (1.72 - 6.57)	3.37 (14.29)	3.26 (1.67)	1.70 (0.47)
	COLONIC ABSCESS	9	2.52 (1.30 - 4.91)	2.52 (8.00)	2.47 (1.27)	1.31 (0.17)
	GENITAL HERPES	7	4.88 (2.27 - 10.49)	4.88 (20.21)	4.63 (2.15)	2.21 (0.61)
	INFECTION SUSCEPTIBILITY INCREASED	7	3.87 (1.81 - 8.27)	3.87 (14.11)	3.72 (1.74)	1.89 (0.42)
	ENCEPHALITIS	7	3.78 (1.77 - 8.08)	3.78 (13.59)	3.64 (1.70)	1.86 (0.40)
	RHINOVIRUS INFECTION	7	11.69 (5.26 - 25.99)	11.69 (58.83)	10.19 (4.58)	3.35 (1.14)
	HORDEOLUM	7	2.38 (1.12 - 5.06)	2.38 (5.43)	2.34 (1.10)	1.22 (-0.04)
	STOMA SITE INFECTION	6	3.03 (1.34 - 6.87)	3.03 (7.85)	2.95 (1.30)	1.56 (0.08)
	PUSTULE	6	3.08 (1.36 - 6.97)	3.08 (8.07)	2.99 (1.32)	1.58 (0.10)
	CORONAVIRUS INFECTION	6	2.63 (1.16 - 5.93)	2.63 (5.83)	2.57 (1.14)	1.36 (-0.05)
	HERPES ZOSTER MENINGITIS	5	23.93 (8.70 - 65.86)	23.93 (82.39)	18.20 (6.61)	4.19 (0.88)
	CYTOMEGALOVIRUS ENTEROCOLITIS	5	4.66 (1.89 - 11.52)	4.66 (13.50)	4.44 (1.80)	2.15 (0.27)
	ATYPICAL PNEUMONIA	5	2.51 (1.03 - 6.12)	2.51 (4.39)	2.46 (1.01)	1.30 (-0.22)
	VAGINAL ABSCESS	5	4.72 (1.91 - 11.68)	4.72 (13.77)	4.49 (1.82)	2.17 (0.28)
	GASTROENTERITIS ESCHERICHIA COLI	5	6.19 (2.48 - 15.43)	6.19 (20.03)	5.78 (2.32)	2.53 (0.44)

	ARTHRITIS INFECTIVE	5	2.46 (1.01 - 6.00)	2.46 (4.18)	2.41 (0.99)	1.27 (-0.24)
	LYMPH NODE TUBERCULOSIS	4	3.30 (1.21 - 8.99)	3.30 (6.13)	3.20 (1.17)	1.68 (-0.18)
	GANGRENE	4	5.63 (2.04 - 15.58)	5.63 (14.13)	5.29 (1.91)	2.40 (0.15)
	ARTHRITIS BACTERIAL	4	2.96 (1.09 - 8.05)	2.96 (4.99)	2.88 (1.06)	1.53 (-0.26)
	MENINGITIS ASEPTIC	4	2.87 (1.06 - 7.80)	2.87 (4.69)	2.80 (1.03)	1.49 (-0.29)
	CYTOMEGALOVIRUS INFECTION REACTIVATION	3	4.14 (1.29 - 13.26)	4.14 (6.76)	3.97 (1.24)	1.99 (-0.32)
	PILONIDAL DISEASE	3	4.14 (1.29 - 13.26)	4.14 (6.76)	3.97 (1.24)	1.99 (-0.32)
	PAROTITIS	3	8.28 (2.51 - 27.37)	8.28 (17.23)	7.53 (2.28)	2.91 (-0.03)
	INTESTINAL SEPSIS	3	6.53 (2.00 - 21.28)	6.53 (12.87)	6.07 (1.86)	2.60 (-0.11)
	HEPATITIS B REACTIVATION	3	10.77 (3.20 - 36.24)	10.77 (23.12)	9.49 (2.82)	3.25 (0.03)
	SUPERINFECTION	3	4.40 (1.37 - 14.10)	4.40 (7.41)	4.20 (1.31)	2.07 (-0.29)
	ORCHITIS	3	3.31 (1.04 - 10.54)	3.31 (4.63)	3.21 (1.01)	1.68 (-0.45)
	PSOAS ABSCESS	3	3.65 (1.14 - 11.65)	3.65 (5.49)	3.52 (1.10)	1.82 (-0.39)
	PNEUMONIA FUNGAL	3	3.26 (1.03 - 10.38)	3.26 (4.50)	3.16 (0.99)	1.66 (-0.46)
	PNEUMONIA CRYPTOCOCCAL	3	6.33 (1.95 - 20.63)	6.33 (12.38)	5.90 (1.81)	2.56 (-0.12)
	PULMONARY SEPSIS	3	6.73 (2.06 - 21.98)	6.73 (13.38)	6.24 (1.91)	2.64 (-0.10)
	APPENDICEAL ABSCESS	3	11.97 (3.52 - 40.63)	11.96 (25.84)	10.40 (3.06)	3.38 (0.05)
Investigations	PRODUCT RESIDUE PRESENT	308	152.25 (125.04 - 185.38)	150.42 (14772.83)	49.27 (40.47)	5.62 (5.20)
	HAEMOGLOBI	111	2.49 (2.06 -	2.48	2.43	1.28

N DECREASED		3.01)	(95.08)	(2.01)	(0.99)
BLOOD CHOLESTEROL INCREASED	95	5.44 (4.41 - 6.70)	5.42 (318.73)	5.11 (4.15)	2.35 (1.99)
HEPATIC ENZYME INCREASED	69	2.06 (1.62 - 2.62)	2.06 (36.70)	2.03 (1.60)	1.02 (0.65)
INFLAMMATORY MARKER INCREASED	38	4.16 (3.00 - 5.77)	4.15 (86.00)	3.98 (2.87)	1.99 (1.41)
BLOOD IRON DECREASED	36	1.55 (1.11 - 2.15)	1.55 (6.83)	1.54 (1.10)	0.62 (0.12)
BLOOD POTASSIUM DECREASED	31	1.60 (1.12 - 2.28)	1.60 (6.74)	1.58 (1.11)	0.66 (0.12)
WHITE BLOOD CELL COUNT DECREASED	28	2.39 (1.64 - 3.48)	2.39 (21.86)	2.34 (1.61)	1.23 (0.62)
LIVER FUNCTION TEST INCREASED	28	4.58 (3.13 - 6.72)	4.58 (73.64)	4.36 (2.98)	2.13 (1.41)
RED BLOOD CELL COUNT DECREASED	27	4.31 (2.92 - 6.36)	4.31 (64.70)	4.12 (2.79)	2.04 (1.33)
BLOOD CREATINE PHOSPHOKINASE INCREASED	25	11.30 (7.41 - 17.23)	11.29 (202.58)	9.89 (6.49)	3.31 (2.27)
FULL BLOOD COUNT DECREASED	25	11.09 (7.28 - 16.90)	11.08 (198.60)	9.73 (6.39)	3.28 (2.26)
LIPIDS INCREASED	24	61.59 (35.70 - 106.26)	61.53 (769.59)	33.60 (19.47)	5.07 (3.15)
BLOOD CREATININE INCREASED	23	3.07 (2.02 - 4.65)	3.06 (30.67)	2.98 (1.96)	1.57 (0.86)
HAEMOGLOBIN ABNORMAL	23	6.53 (4.26 - 10.01)	6.53 (98.68)	6.07 (3.96)	2.60 (1.71)
BLOOD TRIGLYCERID	22	7.06 (4.55 - 10.93)	7.05 (104.03)	6.51 (4.20)	2.70 (1.76)

ES					
INCREASED PLATELET COUNT INCREASED	20	3.49 (2.23 - 5.46)	3.48 (33.80)	3.37 (2.15)	1.75 (0.95)
ASPARTATE AMINOTRANS FERASE INCREASED	19	2.93 (1.85 - 4.63)	2.93 (23.17)	2.85 (1.80)	1.51 (0.72)
LOW DENSITY LIPOPROTEIN INCREASED	19	27.86 (16.40 - 47.32)	27.84 (354.24)	20.34 (11.97)	4.35 (2.63)
SARS-COV-2 TEST POSITIVE	18	1.80 (1.13 - 2.87)	1.80 (6.21)	1.78 (1.11)	0.83 (0.10)
ALANINE AMINOTRANS FERASE INCREASED	18	1.84 (1.15 - 2.94)	1.84 (6.71)	1.82 (1.14)	0.86 (0.13)
PLATELET COUNT DECREASED	16	2.02 (1.23 - 3.31)	2.02 (7.96)	1.99 (1.21)	0.99 (0.20)
WHITE BLOOD CELL COUNT ABNORMAL	13	8.98 (5.04 - 15.98)	8.97 (81.88)	8.09 (4.54)	3.02 (1.60)
LYMPHOCYT E COUNT DECREASED	12	4.08 (2.28 - 7.31)	4.08 (26.43)	3.92 (2.19)	1.97 (0.85)
BLOOD SODIUM DECREASED	11	2.69 (1.47 - 4.91)	2.69 (11.22)	2.63 (1.44)	1.39 (0.36)
BLOOD CALCIUM DECREASED	11	3.46 (1.89 - 6.35)	3.46 (18.39)	3.35 (1.83)	1.74 (0.63)
FAECAL CALPROTECT IN ABNORMAL	10	2.58 (1.37 - 4.85)	2.58 (9.36)	2.53 (1.34)	1.34 (0.26)
BLOOD CHOLESTERO L ABNORMAL	9	7.79 (3.91 - 15.49)	7.78 (48.01)	7.12 (3.58)	2.83 (1.17)
RENAL FUNCTION	9	6.46 (3.27 - 12.79)	6.46 (38.11)	6.01 (3.04)	2.59 (1.04)

TEST ABNORMAL					
NEUTROPHIL COUNT DECREASED	9	3.61 (1.85 - 7.05)	3.61 (16.17)	3.48 (1.78)	1.80 (0.54)
CLOSTRIDIUM TEST POSITIVE	7	3.51 (1.65 - 7.51)	3.51 (12.00)	3.40 (1.59)	1.76 (0.33)
GASTROINTESTINAL STOMA OUTPUT INCREASED	6	3.14 (1.39 - 7.12)	3.14 (8.41)	3.05 (1.35)	1.61 (0.12)
GLOMERULAR FILTRATION RATE DECREASED	6	5.19 (2.27 - 11.89)	5.19 (18.93)	4.91 (2.14)	2.29 (0.51)
BLOOD UREA INCREASED	6	3.65 (1.61 - 8.29)	3.65 (10.99)	3.52 (1.55)	1.82 (0.24)
FULL BLOOD COUNT ABNORMAL	5	3.45 (1.41 - 8.47)	3.45 (8.31)	3.34 (1.36)	1.74 (0.05)
URINE OUTPUT DECREASED	4	3.19 (1.17 - 8.69)	3.19 (5.76)	3.10 (1.14)	1.63 (-0.21)
BLOOD ELECTROLYTES DECREASED	4	3.99 (1.46 - 10.92)	3.99 (8.49)	3.83 (1.40)	1.94 (-0.05)
LIPIDS ABNORMAL	4	23.93 (7.72 - 74.21)	23.93 (65.92)	18.20 (5.87)	4.19 (0.55)
HIGH DENSITY LIPOPROTEIN INCREASED	4	23.93 (7.72 - 74.21)	23.93 (65.92)	18.20 (5.87)	4.19 (0.55)
BLOOD CREATINE INCREASED	4	7.76 (2.77 - 21.78)	7.76 (21.26)	7.10 (2.53)	2.83 (0.30)
FIBRIN D DIMER INCREASED	4	4.79 (1.74 - 13.17)	4.79 (11.23)	4.55 (1.65)	2.19 (0.06)
INVESTIGATION ABNORMAL	4	3.12 (1.15 - 8.50)	3.12 (5.53)	3.03 (1.11)	1.60 (-0.22)
BLOOD	4	5.63 (2.04 -	5.63	5.29	2.40

	CREATININE ABNORMAL		15.58)	(14.13)	(1.91)	(0.15)
	BLOOD CREATINE PHOSPHOKINASE ABNORMAL	4	287.20 (32.10 - 2569.77)	287.16 (228.13)	58.23 (6.51)	5.86 (0.46)
	CATHETERISATION CARDIAC	3	5.01 (1.55 - 16.15)	5.01 (9.00)	4.75 (1.47)	2.25 (-0.22)
	BLOOD IRON ABNORMAL	3	4.22 (1.32 - 13.53)	4.22 (6.97)	4.04 (1.26)	2.02 (-0.31)
	NEUTROPHIL COUNT ABNORMAL	3	14.36 (4.16 - 49.60)	14.36 (31.07)	12.13 (3.51)	3.60 (0.08)
	HARVEY-BRADSHAW INDEX ABNORMAL	3	16.57 (4.72 - 58.15)	16.57 (35.65)	13.65 (3.89)	3.77 (0.09)
	MEAN CELL VOLUME INCREASED	3	5.13 (1.59 - 16.55)	5.13 (9.30)	4.85 (1.50)	2.28 (-0.21)
	STOOL ANALYSIS ABNORMAL	3	3.71 (1.16 - 11.85)	3.71 (5.66)	3.58 (1.12)	1.84 (-0.38)
Gastrointestinal disorders	COLITIS ULCERATIVE	505	1.38 (1.26 - 1.51)	1.37 (51.18)	1.37 (1.25)	0.45 (0.32)
	ABDOMINAL PAIN UPPER	148	1.28 (1.09 - 1.51)	1.28 (8.95)	1.28 (1.08)	0.35 (0.11)
	CONSTIPATION	142	1.39 (1.18 - 1.65)	1.39 (15.37)	1.38 (1.17)	0.47 (0.22)
	GASTROINTESTINAL DISORDER	137	2.46 (2.07 - 2.91)	2.45 (113.77)	2.40 (2.02)	1.26 (1.00)
	ABDOMINAL DISTENSION	127	1.43 (1.20 - 1.71)	1.43 (16.10)	1.42 (1.19)	0.51 (0.24)
	COLITIS	126	2.01 (1.68 - 2.40)	2.01 (61.96)	1.98 (1.66)	0.98 (0.71)
	FLATULENCE	112	2.20 (1.82 - 2.66)	2.19 (70.80)	2.16 (1.79)	1.11 (0.82)
	DEFAECATION URGENCY	78	1.96 (1.57 - 2.46)	1.96 (35.76)	1.93 (1.54)	0.95 (0.60)
	MALABSORPTION	64	15.15 (11.57 - 19.84)	15.11 (696.95)	12.66 (9.67)	3.66 (3.03)

	SMALL INTESTINAL OBSTRUCTION	61	1.42 (1.10 - 1.83)	1.42 (7.32)	1.41 (1.09)	0.49 (0.11)
	GASTROINTESTINAL HAEMORRHAGE	59	3.18 (2.45 - 4.13)	3.18 (84.37)	3.09 (2.38)	1.63 (1.19)
	INTESTINAL PERFORATION	48	2.25 (1.68 - 2.99)	2.24 (32.11)	2.21 (1.65)	1.14 (0.69)
	GASTROINTESTINAL PAIN	36	1.84 (1.32 - 2.56)	1.84 (13.46)	1.82 (1.31)	0.86 (0.35)
	LARGE INTESTINE PERFORATION	35	4.06 (2.89 - 5.71)	4.06 (76.40)	3.90 (2.77)	1.96 (1.35)
	PROCTALGIA	30	1.54 (1.07 - 2.21)	1.54 (5.50)	1.52 (1.06)	0.61 (0.06)
	INTESTINAL HAEMORRHAGE	29	3.30 (2.27 - 4.78)	3.29 (44.32)	3.19 (2.20)	1.68 (1.03)
	LARGE INTESTINAL HAEMORRHAGE	28	6.49 (4.41 - 9.56)	6.48 (119.14)	6.03 (4.09)	2.59 (1.80)
	LARGE INTESTINE POLYP	28	1.78 (1.23 - 2.59)	1.78 (9.38)	1.76 (1.21)	0.82 (0.24)
	INFLAMMATORY BOWEL DISEASE	27	1.68 (1.15 - 2.47)	1.68 (7.33)	1.67 (1.14)	0.74 (0.15)
	ENTERITIS	24	1.71 (1.14 - 2.56)	1.71 (6.88)	1.69 (1.13)	0.76 (0.13)
	FAECES DISCOLOURED	22	1.59 (1.04 - 2.43)	1.59 (4.76)	1.58 (1.04)	0.66 (0.01)
	GASTROINTESTINAL SCARRING	20	3.42 (2.18 - 5.36)	3.42 (32.67)	3.31 (2.11)	1.73 (0.93)
	SMALL INTESTINAL PERFORATION	16	5.09 (3.06 - 8.44)	5.08 (49.00)	4.81 (2.90)	2.27 (1.25)

LARGE INTESTINAL OBSTRUCTION	16	1.94 (1.18 - 3.18)	1.94 (7.06)	1.91 (1.16)	0.94 (0.15)
GASTROINTESTINAL PERFORATION	15	6.91 (4.07 - 11.73)	6.90 (69.08)	6.39 (3.76)	2.67 (1.50)
GASTROINTESTINAL WALL THICKENING	13	2.21 (1.27 - 3.84)	2.21 (8.37)	2.18 (1.25)	1.12 (0.22)
SMALL INTESTINAL HAEMORRHAGE	12	11.65 (6.33 - 21.44)	11.64 (100.45)	10.16 (5.52)	3.34 (1.71)
GASTRIC HAEMORRHAGE	11	4.57 (2.48 - 8.40)	4.56 (28.79)	4.35 (2.37)	2.12 (0.90)
GASTRIC PERFORATION	8	7.56 (3.65 - 15.66)	7.56 (41.18)	6.93 (3.35)	2.79 (1.04)
COLON DYSPLASIA	8	3.44 (1.69 - 6.99)	3.44 (13.21)	3.33 (1.64)	1.73 (0.41)
IMPAIRED GASTRIC EMPTYING	8	2.58 (1.27 - 5.22)	2.58 (7.44)	2.52 (1.25)	1.33 (0.12)
LOWER GASTROINTESTINAL HAEMORRHAGE	7	5.35 (2.48 - 11.53)	5.35 (23.02)	5.04 (2.34)	2.33 (0.68)
ORAL MUCOSAL BLISTERING	5	3.45 (1.41 - 8.47)	3.45 (8.31)	3.34 (1.36)	1.74 (0.05)
COLITIS ISCHAEMIC	5	5.44 (2.19 - 13.50)	5.44 (16.84)	5.13 (2.06)	2.36 (0.37)
INTESTINAL CYST	4	3.19 (1.17 - 8.69)	3.19 (5.76)	3.10 (1.14)	1.63 (-0.21)
GASTRIC ULCER HAEMORRHAGE	4	4.35 (1.59 - 11.94)	4.35 (9.73)	4.16 (1.52)	2.06 (0.00)
GASTROINTESTINAL	4	5.04 (1.83 - 13.89)	5.04 (12.10)	4.77 (1.73)	2.25 (0.09)

	ULCER HAEMORRHAGE					
	PNEUMOPERITONEUM	4	6.68 (2.40 - 18.61)	6.68 (17.67)	6.19 (2.22)	2.63 (0.24)
	HAEMORRHOIDS THROMBOSED	4	6.11 (2.20 - 16.96)	6.11 (15.75)	5.71 (2.06)	2.51 (0.19)
	GASTROINTESTINAL TRACT MUCOSAL DISCOLOURATION	3	30.77 (7.96 - 119.00)	30.77 (60.48)	21.84 (5.65)	4.45 (0.11)
	AUTOIMMUNE PANCREATITIS	3	3.47 (1.09 - 11.07)	3.47 (5.04)	3.36 (1.05)	1.75 (-0.42)
	LIP BLISTER	3	5.25 (1.63 - 16.97)	5.25 (9.63)	4.96 (1.54)	2.31 (-0.20)
	ORAL PRURITUS	3	6.53 (2.00 - 21.28)	6.53 (12.87)	6.07 (1.86)	2.60 (-0.11)
	LIP PAIN	3	4.40 (1.37 - 14.10)	4.40 (7.41)	4.20 (1.31)	2.07 (-0.29)
	PEPTIC ULCER	3	4.68 (1.46 - 15.06)	4.68 (8.15)	4.46 (1.39)	2.16 (-0.25)
Injury, poisoning and procedural complications	FALL	139	1.74 (1.47 - 2.06)	1.73 (42.32)	1.72 (1.45)	0.78 (0.52)
	MATERNAL EXPOSURE DURING PREGNANCY	76	1.69 (1.34 - 2.12)	1.68 (20.64)	1.67 (1.33)	0.74 (0.39)
	POST PROCEDURAL COMPLICATION	30	1.47 (1.03 - 2.12)	1.47 (4.49)	1.46 (1.02)	0.55 (0.00)
	SCAR	27	2.34 (1.59 - 3.43)	2.34 (19.97)	2.29 (1.56)	1.20 (0.58)
	HIP FRACTURE	20	2.56 (1.64 - 4.00)	2.56 (18.35)	2.51 (1.60)	1.33 (0.58)
	HEAD INJURY	20	2.17 (1.39 -	2.17	2.13	1.09

		3.38)	(12.18)	(1.37)	(0.37)
PRODUCT DISPENSING ERROR	19	5.07 (3.19 - 8.08)	5.07 (58.00)	4.80 (3.02)	2.26 (1.34)
SPINAL FRACTURE	18	2.86 (1.79 - 4.58)	2.86 (20.93)	2.79 (1.74)	1.48 (0.67)
UPPER LIMB FRACTURE	17	2.23 (1.37 - 3.61)	2.23 (11.15)	2.19 (1.35)	1.13 (0.35)
POUCHITIS	17	2.58 (1.59 - 4.18)	2.57 (15.81)	2.52 (1.55)	1.33 (0.52)
FOOT FRACTURE	16	2.31 (1.40 - 3.80)	2.31 (11.48)	2.27 (1.38)	1.18 (0.36)
PELVIC FRACTURE	13	5.43 (3.09 - 9.54)	5.43 (43.64)	5.11 (2.91)	2.35 (1.18)
ACCIDENT	13	2.19 (1.26 - 3.81)	2.19 (8.17)	2.16 (1.24)	1.11 (0.21)
BACK INJURY	11	2.42 (1.32 - 4.41)	2.41 (8.82)	2.37 (1.30)	1.24 (0.24)
PRODUCT USE COMPLAINT	10	6.84 (3.57 - 13.09)	6.84 (45.50)	6.33 (3.31)	2.66 (1.17)
TENDON RUPTURE	10	2.56 (1.36 - 4.80)	2.55 (9.14)	2.50 (1.33)	1.32 (0.25)
WOUND COMPLICATI ON	9	2.52 (1.30 - 4.91)	2.52 (8.00)	2.47 (1.27)	1.31 (0.17)
FEMUR FRACTURE	9	2.85 (1.46 - 5.54)	2.85 (10.37)	2.78 (1.43)	1.47 (0.30)
SHOULDER FRACTURE	8	7.66 (3.69 - 15.88)	7.66 (41.84)	7.02 (3.38)	2.81 (1.05)
LIGAMENT RUPTURE	8	2.14 (1.06 - 4.33)	2.14 (4.74)	2.11 (1.04)	1.08 (-0.07)
STOMA SITE PAIN	7	3.40 (1.59 - 7.25)	3.40 (11.30)	3.29 (1.54)	1.72 (0.30)
STOMA SITE HAEMORRHA GE	7	2.18 (1.03 - 4.62)	2.18 (4.32)	2.14 (1.01)	1.10 (-0.14)
STOMA SITE DISCHARGE	6	3.59 (1.58 - 8.15)	3.59 (10.68)	3.47 (1.53)	1.79 (0.23)
SPINAL COMPRESSIO N FRACTURE	6	2.34 (1.04 - 5.28)	2.34 (4.46)	2.30 (1.02)	1.20 (-0.16)
FACE INJURY	6	4.49 (1.97 - 10.24)	4.49 (15.30)	4.28 (1.88)	2.10 (0.41)

	STOMA PROLAPSE	5	9.70 (3.81 - 24.69)	9.70 (34.38)	8.67 (3.41)	3.12 (0.66)
	FRACTURE	5	2.48 (1.02 - 6.04)	2.48 (4.25)	2.43 (0.99)	1.28 (-0.24)
	STOMA SITE INFLAMMATION	5	5.52 (2.22 - 13.72)	5.52 (17.19)	5.20 (2.09)	2.38 (0.38)
	INCISION SITE HAEMORRHAGE	5	14.36 (5.50 - 37.52)	14.36 (51.78)	12.13 (4.64)	3.60 (0.79)
	POST PROCEDURAL INFLAMMATION	5	2.53 (1.04 - 6.17)	2.53 (4.46)	2.48 (1.01)	1.31 (-0.22)
	COMPRESSION FRACTURE	5	4.79 (1.94 - 11.84)	4.79 (14.04)	4.55 (1.84)	2.19 (0.28)
	PROCEDURAL INTESTINAL PERFORATION	5	3.15 (1.29 - 7.71)	3.15 (7.02)	3.06 (1.25)	1.61 (-0.03)
	LUMBAR VERTEBRAL FRACTURE	4	3.46 (1.27 - 9.44)	3.46 (6.67)	3.35 (1.23)	1.74 (-0.15)
	GASTROINTESTINAL ANASTOMOTIC HAEMORRHAGE	4	inf (inf - inf)	inf (287.16)	72.79 (0.00)	6.19 (0.40)
	GASTROINTESTINAL INJURY	4	2.90 (1.07 - 7.88)	2.90 (4.79)	2.83 (1.04)	1.50 (-0.28)
	FOREIGN BODY IN GASTROINTESTINAL TRACT	3	5.38 (1.67 - 17.41)	5.38 (9.96)	5.08 (1.57)	2.34 (-0.19)
	STOMA SITE OEDEMA	3	4.58 (1.43 - 14.72)	4.58 (7.90)	4.37 (1.36)	2.13 (-0.26)
	STOMA SITE ULCER	3	3.31 (1.04 - 10.54)	3.31 (4.63)	3.21 (1.01)	1.68 (-0.45)
	STOMA SITE REACTION	3	3.53 (1.11 - 11.25)	3.53 (5.19)	3.41 (1.07)	1.77 (-0.41)

	EXPULSION OF MEDICATION	3	107.70 (17.99 - 644.57)	107.68 (126.83)	43.67 (7.30)	5.45 (0.02)
General disorders and administration site conditions	PAIN	274	1.20 (1.06 - 1.35)	1.20 (8.86)	1.19 (1.06)	0.26 (0.08)
	PYREXIA	261	1.28 (1.13 - 1.45)	1.28 (15.44)	1.27 (1.12)	0.35 (0.16)
	ASTHENIA	167	1.21 (1.04 - 1.41)	1.21 (5.80)	1.20 (1.03)	0.27 (0.04)
	ILLNESS	126	2.49 (2.08 - 2.97)	2.48 (107.95)	2.43 (2.04)	1.28 (1.00)
	DEATH	121	1.59 (1.33 - 1.90)	1.59 (25.71)	1.57 (1.31)	0.65 (0.38)
	THERAPEUTIC PRODUCT EFFECT INCOMPLETE	119	2.38 (1.98 - 2.86)	2.37 (91.51)	2.33 (1.94)	1.22 (0.93)
	UNEVALUABLE EVENT	110	3.56 (2.94 - 4.31)	3.55 (192.15)	3.43 (2.83)	1.78 (1.47)
	INFLAMMATION	107	1.93 (1.59 - 2.34)	1.93 (46.65)	1.90 (1.57)	0.93 (0.63)
	CHILLS	106	1.80 (1.48 - 2.18)	1.80 (36.57)	1.78 (1.46)	0.83 (0.54)
	THERAPEUTIC PRODUCT EFFECT DECREASED	96	2.11 (1.72 - 2.58)	2.10 (54.01)	2.07 (1.69)	1.05 (0.74)
	PERIPHERAL SWELLING	86	1.48 (1.19 - 1.83)	1.48 (13.00)	1.47 (1.18)	0.55 (0.23)
	HERNIA	58	2.13 (1.64 - 2.77)	2.13 (33.75)	2.10 (1.61)	1.07 (0.66)
	ADVERSE DRUG REACTION	57	1.96 (1.51 - 2.55)	1.96 (26.03)	1.93 (1.48)	0.95 (0.54)
	OBSTRUCTION	35	2.14 (1.53 - 3.00)	2.14 (20.71)	2.11 (1.51)	1.08 (0.54)
	SWELLING FACE	33	1.62 (1.15 - 2.29)	1.62 (7.61)	1.60 (1.14)	0.68 (0.15)
	STENOSIS	30	4.60 (3.18 - 6.65)	4.59 (79.25)	4.38 (3.02)	2.13 (1.44)
	GAIT INABILITY	26	2.08 (1.41 - 3.07)	2.08 (14.15)	2.05 (1.39)	1.03 (0.41)

	POLYP	19	1.69 (1.08 - 2.67)	1.69 (5.28)	1.68 (1.06)	0.75 (0.04)
	PERFORATION	8	6.92 (3.35 - 14.30)	6.92 (36.95)	6.40 (3.10)	2.68 (0.98)
	ULCER HAEMORRHAGE	8	2.83 (1.40 - 5.74)	2.83 (9.10)	2.76 (1.36)	1.46 (0.22)
	DYSPLASIA	7	2.81 (1.32 - 5.98)	2.81 (7.84)	2.74 (1.29)	1.45 (0.12)
	PRECANCEROUS CONDITION	5	6.77 (2.71 - 16.95)	6.77 (22.48)	6.27 (2.51)	2.65 (0.49)
	THERAPEUTIC PRODUCT EFFECT VARIABLE	5	6.65 (2.66 - 16.62)	6.65 (21.96)	6.17 (2.47)	2.62 (0.48)
	TEMPERATURE INTOLERANCE	4	2.93 (1.08 - 7.96)	2.93 (4.89)	2.85 (1.05)	1.51 (-0.27)
	MEDICAL DEVICE SITE PAIN	4	9.57 (3.37 - 27.18)	9.57 (27.09)	8.56 (3.02)	3.10 (0.38)
	SUDDEN DEATH	3	4.06 (1.27 - 13.01)	4.06 (6.56)	3.90 (1.22)	1.96 (-0.33)
Neoplasms benign, malignant and unspecified (incl cysts and polyps)	NEOPLASM MALIGNANT	49	2.42 (1.82 - 3.21)	2.41 (39.31)	2.37 (1.78)	1.24 (0.79)
	BASAL CELL CARCINOMA	42	2.31 (1.70 - 3.14)	2.31 (30.09)	2.26 (1.67)	1.18 (0.69)
	SKIN CANCER	35	1.81 (1.30 - 2.54)	1.81 (12.42)	1.79 (1.28)	0.84 (0.32)
	COLON CANCER	34	3.77 (2.67 - 5.32)	3.77 (65.67)	3.63 (2.57)	1.86 (1.25)
	MALIGNANT MELANOMA	28	2.44 (1.67 - 3.55)	2.44 (22.95)	2.39 (1.64)	1.26 (0.64)
	PROSTATE CANCER	20	2.28 (1.46 - 3.56)	2.28 (13.98)	2.24 (1.44)	1.17 (0.44)
	BREAST CANCER FEMALE	18	3.29 (2.05 - 5.28)	3.29 (27.41)	3.19 (1.99)	1.67 (0.84)
	SQUAMOUS	16	3.48 (2.11 -	3.48	3.37	1.75

	CELL CARCINOMA OF SKIN		5.75)	(26.98)	(2.04)	(0.84)
	RECTAL CANCER	12	3.68 (2.06 - 6.58)	3.68 (22.30)	3.55 (1.99)	1.83 (0.74)
	BRAIN NEOPLASM	9	2.66 (1.37 - 5.17)	2.66 (8.98)	2.60 (1.34)	1.38 (0.23)
	PANCREATIC CARCINOMA	9	3.57 (1.83 - 6.97)	3.57 (15.86)	3.45 (1.77)	1.79 (0.53)
	GASTRIC CANCER	8	5.37 (2.62 - 11.01)	5.37 (26.45)	5.06 (2.47)	2.34 (0.80)
	ADENOCARCINOMA	6	2.83 (1.25 - 6.41)	2.83 (6.85)	2.76 (1.22)	1.47 (0.02)
	CHOLANGIOCARCINOMA	6	5.13 (2.24 - 11.74)	5.13 (18.61)	4.85 (2.12)	2.28 (0.51)
	CHRONIC MYELOID LEUKAEMIA	5	3.52 (1.43 - 8.64)	3.52 (8.60)	3.40 (1.39)	1.77 (0.06)
	PAPILLARY THYROID CANCER	5	5.52 (2.22 - 13.72)	5.52 (17.19)	5.20 (2.09)	2.38 (0.38)
	INVASIVE DUCTAL BREAST CARCINOMA	4	4.35 (1.59 - 11.94)	4.35 (9.73)	4.16 (1.52)	2.06 (0.00)
	HEPATIC NEOPLASM	4	3.59 (1.32 - 9.80)	3.59 (7.12)	3.47 (1.27)	1.79 (-0.12)
	RECTAL ADENOCARCINOMA	3	3.78 (1.18 - 12.07)	3.78 (5.82)	3.64 (1.14)	1.86 (-0.37)
	LUNG CARCINOMA CELL TYPE UNSPECIFIED STAGE IV	3	5.82 (1.79 - 18.88)	5.82 (11.08)	5.46 (1.68)	2.45 (-0.16)
	APPENDIX CANCER	3	6.53 (2.00 - 21.28)	6.53 (12.87)	6.07 (1.86)	2.60 (-0.11)
Nervous system disorders	LOSS OF CONSCIOUSNESS	95	2.40 (1.95 - 2.94)	2.39 (74.60)	2.35 (1.91)	1.23 (0.91)
	CEREBROVASCULAR ACCIDENT	45	1.75 (1.30 - 2.35)	1.75 (14.11)	1.73 (1.29)	0.79 (0.34)
	SYNCOPE	40	1.54 (1.13 -	1.54	1.53	0.62

			2.11)	(7.49)	(1.12)	(0.14)
	SEIZURE	34	1.69 (1.20 - 2.37)	1.69 (9.27)	1.67 (1.19)	0.74 (0.22)
	TRANSIENT ISCHAEMIC ATTACK	30	4.30 (2.98 - 6.22)	4.30 (71.67)	4.11 (2.84)	2.04 (1.37)
	BALANCE DISORDER	30	2.24 (1.56 - 3.22)	2.24 (19.90)	2.20 (1.53)	1.14 (0.55)
	BRAIN FOG	21	5.96 (3.82 - 9.31)	5.96 (80.03)	5.58 (3.57)	2.48 (1.56)
	NERVE COMPRESSION	12	2.30 (1.29 - 4.08)	2.30 (8.52)	2.26 (1.27)	1.17 (0.22)
	DYSSTASIA	12	2.07 (1.16 - 3.67)	2.07 (6.42)	2.04 (1.15)	1.03 (0.10)
	MOVEMENT DISORDER	11	2.24 (1.23 - 4.08)	2.24 (7.30)	2.20 (1.21)	1.14 (0.15)
	CEREBRAL THROMBOSIS	6	4.79 (2.09 - 10.94)	4.79 (16.85)	4.55 (1.99)	2.19 (0.46)
	SLEEP DEFICIT	4	5.22 (1.89 - 14.41)	5.22 (12.73)	4.93 (1.79)	2.30 (0.11)
	CEREBRAL DISORDER	4	2.87 (1.06 - 7.80)	2.87 (4.69)	2.80 (1.03)	1.49 (-0.29)
	POST HERPETIC NEURALGIA	3	5.38 (1.67 - 17.41)	5.38 (9.96)	5.08 (1.57)	2.34 (-0.19)
	PSEUDOSTROKE	3	43.08 (10.29 - 180.27)	43.07 (77.06)	27.30 (6.52)	4.77 (0.10)
	HAEMORRHAGIC STROKE	3	3.53 (1.11 - 11.25)	3.53 (5.19)	3.41 (1.07)	1.77 (-0.41)
	IDIOPATHIC INTRACRANIAL HYPERTENSION	3	4.14 (1.29 - 13.26)	4.14 (6.76)	3.97 (1.24)	1.99 (-0.32)
Vascular disorders	HAEMORRHAGE	136	2.42 (2.04 - 2.87)	2.41 (108.93)	2.37 (1.99)	1.24 (0.98)
	THROMBOSIS	67	2.44 (1.92 - 3.12)	2.44 (55.15)	2.39 (1.88)	1.26 (0.87)
	DEEP VEIN THROMBOSIS	46	2.87 (2.14 - 3.85)	2.87 (53.80)	2.79 (2.08)	1.48 (1.00)
	INTERNAL HAEMORRHAGE	22	5.18 (3.36 - 7.99)	5.18 (69.19)	4.90 (3.18)	2.29 (1.44)

	EMBOLISM VENOUS	15	15.84 (9.06 - 27.72)	15.84 (170.82)	13.15 (7.52)	3.72 (2.11)
	EMBOLISM	11	9.19 (4.90 - 17.21)	9.18 (71.11)	8.25 (4.41)	3.05 (1.47)
	ARTERIAL OCCLUSIVE DISEASE	9	3.15 (1.62 - 6.15)	3.15 (12.67)	3.06 (1.57)	1.61 (0.41)
	VENOUS THROMBOSIS LIMB	6	8.79 (3.77 - 20.53)	8.79 (36.91)	7.94 (3.40)	2.99 (0.83)
	ANEURYSM	4	3.73 (1.36 - 10.19)	3.73 (7.60)	3.59 (1.32)	1.85 (-0.10)
	INTERMITTENT CLAUDICATION	4	16.89 (5.68 - 50.21)	16.89 (48.41)	13.86 (4.66)	3.79 (0.51)
	SHOCK HAEMORRHAGIC	4	5.63 (2.04 - 15.58)	5.63 (14.13)	5.29 (1.91)	2.40 (0.15)
	ANGIOPATHY	4	3.26 (1.20 - 8.89)	3.26 (6.01)	3.16 (1.16)	1.66 (-0.19)
	VENOUS THROMBOSIS	4	2.84 (1.05 - 7.72)	2.84 (4.60)	2.77 (1.02)	1.47 (-0.30)
	ORTHOSTATIC HYPOTENSION	4	3.64 (1.33 - 9.93)	3.63 (7.27)	3.51 (1.28)	1.81 (-0.11)
	PELVIC VENOUS THROMBOSIS	3	8.97 (2.70 - 29.81)	8.97 (18.89)	8.09 (2.44)	3.02 (-0.01)
	VENA CAVA THROMBOSIS	3	4.68 (1.46 - 15.06)	4.68 (8.15)	4.46 (1.39)	2.16 (-0.25)
Eye disorders	CATARACT	69	2.65 (2.08 - 3.37)	2.64 (67.98)	2.58 (2.03)	1.37 (0.98)
	VISION BLURRED	43	1.39 (1.03 - 1.88)	1.39 (4.61)	1.38 (1.02)	0.47 (0.01)
	VISUAL IMPAIRMENT	39	1.45 (1.06 - 1.99)	1.45 (5.31)	1.44 (1.05)	0.53 (0.05)
	BLINDNESS	25	3.65 (2.44 - 5.46)	3.65 (45.74)	3.52 (2.35)	1.82 (1.10)
	VITREOUS FLOATERS	22	10.13 (6.48 - 15.84)	10.12 (158.56)	9.00 (5.76)	3.17 (2.09)
	RETINAL DETACHMEN	20	5.99 (3.79 - 9.45)	5.98 (76.62)	5.60 (3.55)	2.49 (1.54)

	T					
	RETINAL TEAR	9	5.62 (2.85 - 11.08)	5.62 (31.69)	5.28 (2.68)	2.40 (0.93)
	EYE HAEMORRHAGE	7	2.86 (1.34 - 6.08)	2.86 (8.12)	2.78 (1.31)	1.48 (0.14)
	RETINAL VEIN OCCLUSION	6	5.67 (2.47 - 13.02)	5.67 (21.38)	5.33 (2.32)	2.41 (0.57)
	RETINAL HAEMORRHAGE	4	6.24 (2.25 - 17.35)	6.24 (16.20)	5.82 (2.10)	2.54 (0.20)
	VITREOUS DETACHMENT	3	5.01 (1.55 - 16.15)	5.01 (9.00)	4.75 (1.47)	2.25 (-0.22)
	RETINAL DEGENERATION	3	15.39 (4.42 - 53.54)	15.38 (33.23)	12.85 (3.69)	3.68 (0.09)
	CORNEAL DISORDER	3	8.97 (2.70 - 29.81)	8.97 (18.89)	8.09 (2.44)	3.02 (-0.01)
	EYE OEDEMA	3	3.99 (1.25 - 12.76)	3.99 (6.36)	3.83 (1.20)	1.94 (-0.34)
Skin and subcutaneous tissue disorders	ACNE	346	11.47 (10.24 - 12.86)	11.33 (2818.84)	9.92 (8.85)	3.31 (3.11)
	SKIN DISORDER	32	2.17 (1.52 - 3.08)	2.17 (19.49)	2.13 (1.50)	1.09 (0.53)
	ACNE CYSTIC	23	24.67 (15.36 - 39.62)	24.64 (388.43)	18.60 (11.58)	4.22 (2.75)
	ROSACEA	17	7.23 (4.39 - 11.90)	7.22 (82.80)	6.65 (4.04)	2.73 (1.62)
	DERMATITIS ACNEIFORM	14	5.32 (3.09 - 9.16)	5.32 (45.70)	5.02 (2.92)	2.33 (1.21)
	PAIN OF SKIN	14	3.12 (1.83 - 5.33)	3.12 (19.35)	3.03 (1.78)	1.60 (0.65)
	SEBORRHOEA	11	18.37 (9.47 - 35.63)	18.36 (143.82)	14.83 (7.65)	3.89 (1.85)
	SKIN ATROPHY	5	3.36 (1.37 - 8.23)	3.35 (7.90)	3.25 (1.33)	1.70 (0.02)
	CUTANEOUS VASCULITIS	5	3.02 (1.23 - 7.38)	3.02 (6.47)	2.94 (1.20)	1.55 (-0.06)
	PURPURA FULMINANS	4	71.80 (17.96 - 287.11)	71.79 (139.61)	36.39 (9.10)	5.19 (0.54)
	SENSITIVE SKIN	4	2.90 (1.07 - 7.88)	2.90 (4.79)	2.83 (1.04)	1.50 (-0.28)

	ACNE FULMINANS	3	13.46 (3.92 - 46.20)	13.46 (29.14)	11.49 (3.35)	3.52 (0.07)
	BLOOD BLISTER	3	3.53 (1.11 - 11.25)	3.53 (5.19)	3.41 (1.07)	1.77 (-0.41)
Musculoskeletal and connective tissue disorders	MUSCLE SPASMS	86	1.27 (1.02 - 1.57)	1.27 (4.73)	1.26 (1.02)	0.33 (0.02)
	INTERVERTEBRAL DISC PROTRUSION	28	2.35 (1.61 - 3.42)	2.35 (21.00)	2.31 (1.58)	1.21 (0.60)
	MUSCULOSKELETAL DISORDER	20	3.99 (2.54 - 6.26)	3.99 (42.43)	3.83 (2.44)	1.94 (1.11)
	BACK DISORDER	18	2.89 (1.81 - 4.63)	2.89 (21.41)	2.82 (1.76)	1.49 (0.68)
	ROTATOR CUFF SYNDROME	15	2.08 (1.25 - 3.48)	2.08 (8.21)	2.05 (1.23)	1.04 (0.21)
	OSTEONECROSIS	10	2.76 (1.47 - 5.19)	2.76 (10.82)	2.70 (1.43)	1.43 (0.33)
	RHABDOMYOLYSIS	8	3.38 (1.66 - 6.87)	3.38 (12.79)	3.27 (1.61)	1.71 (0.39)
	SPINAL DISORDER	7	3.31 (1.55 - 7.06)	3.31 (10.77)	3.20 (1.50)	1.68 (0.28)
	SPINAL STENOSIS	7	3.93 (1.83 - 8.40)	3.93 (14.47)	3.77 (1.76)	1.92 (0.43)
	TENDON DISORDER	4	3.83 (1.40 - 10.47)	3.83 (7.94)	3.69 (1.35)	1.88 (-0.08)
	FRACTURE PAIN	3	26.92 (7.14 - 101.49)	26.92 (54.45)	19.85 (5.27)	4.31 (0.11)
	CHONDROCALCINOSIS	3	23.93 (6.48 - 88.41)	23.93 (49.44)	18.20 (4.93)	4.19 (0.11)
Respiratory, thoracic and mediastinal disorders	PULMONARY EMBOLISM	86	3.43 (2.77 - 4.27)	3.43 (141.16)	3.32 (2.67)	1.73 (1.37)
	PULMONARY THROMBOSIS	55	9.74 (7.35 - 12.92)	9.73 (379.27)	8.68 (6.55)	3.12 (2.52)
	INTERSTITIAL LUNG DISEASE	22	2.50 (1.63 - 3.83)	2.50 (19.12)	2.45 (1.60)	1.29 (0.59)
	RESPIRATORY DISORDER	22	1.95 (1.28 - 2.98)	1.95 (9.88)	1.92 (1.26)	0.94 (0.27)
	PNEUMOTHO	16	2.84 (1.73 -	2.84	2.77	1.47

	RAX		4.69)	(18.39)	(1.68)	(0.61)
	PULMONARY OEDEMA	15	2.02 (1.21 - 3.37)	2.02 (7.48)	1.99 (1.19)	0.99 (0.17)
	CHOKING	11	3.36 (1.84 - 6.15)	3.36 (17.42)	3.25 (1.78)	1.70 (0.60)
	PHARYNGEAL SWELLING	9	3.20 (1.64 - 6.24)	3.20 (13.02)	3.10 (1.59)	1.63 (0.42)
	PHARYNGEAL DISORDER	7	5.71 (2.65 - 12.33)	5.71 (25.20)	5.36 (2.48)	2.42 (0.72)
	PULMONARY HYPERTENSION	7	5.98 (2.77 - 12.94)	5.98 (26.81)	5.60 (2.59)	2.49 (0.76)
	RESPIRATORY SYMPTOM	5	4.17 (1.69 - 10.29)	4.17 (11.40)	4.00 (1.62)	2.00 (0.19)
	PAINFUL RESPIRATION	5	2.85 (1.17 - 6.96)	2.85 (5.77)	2.78 (1.14)	1.47 (-0.11)
Cardiac disorders	CARDIAC DISORDER	39	2.52 (1.83 - 3.47)	2.52 (34.50)	2.47 (1.79)	1.30 (0.78)
	ATRIAL FIBRILLATION	30	1.89 (1.31 - 2.71)	1.89 (12.21)	1.86 (1.30)	0.90 (0.33)
	ACUTE MYOCARDIAL INFARCTION	9	3.57 (1.83 - 6.97)	3.57 (15.86)	3.45 (1.77)	1.79 (0.53)
	CARDIAC FLUTTER	8	4.91 (2.40 - 10.05)	4.91 (23.31)	4.66 (2.28)	2.22 (0.72)
	VENTRICULAR EXTRASYSTOLES	5	3.78 (1.54 - 9.29)	3.78 (9.71)	3.64 (1.48)	1.86 (0.12)
	VENTRICULAR TACHYCARDIA	4	3.50 (1.28 - 9.56)	3.50 (6.82)	3.39 (1.24)	1.76 (-0.14)
	CORONARY ARTERY STENOSIS	3	4.79 (1.49 - 15.40)	4.79 (8.42)	4.55 (1.41)	2.19 (-0.24)
Psychiatric disorders	SUICIDAL IDEATION	25	2.26 (1.51 - 3.36)	2.25 (16.93)	2.22 (1.49)	1.15 (0.51)
	DISORIENTATION	9	2.01 (1.03 - 3.89)	2.01 (4.42)	1.98 (1.02)	0.98 (-0.08)
	PANIC REACTION	6	3.95 (1.74 - 8.99)	3.95 (12.54)	3.80 (1.67)	1.93 (0.31)
	COMPLETED SUICIDE	6	3.95 (1.74 - 8.99)	3.95 (12.54)	3.80 (1.67)	1.93 (0.31)

	ABNORMAL DREAMS	5	4.60 (1.86 - 11.37)	4.60 (13.25)	4.38 (1.78)	2.13 (0.26)
	SCHIZOPHRENIA	4	4.16 (1.52 - 11.41)	4.16 (9.08)	3.99 (1.46)	2.00 (-0.02)
	SUICIDE THREAT	3	10.26 (3.06 - 34.39)	10.26 (21.93)	9.10 (2.71)	3.19 (0.02)
Blood and lymphatic system disorders	ANAEMIA	126	1.87 (1.57 - 2.24)	1.87 (49.78)	1.85 (1.55)	0.89 (0.62)
	LEUKOPENIA	16	1.84 (1.12 - 3.03)	1.84 (6.02)	1.82 (1.11)	0.87 (0.09)
	NEUTROPENIA	15	2.35 (1.41 - 3.93)	2.35 (11.28)	2.31 (1.38)	1.21 (0.36)
	THROMBOCYTOSIS	6	4.22 (1.85 - 9.62)	4.22 (13.94)	4.04 (1.77)	2.02 (0.36)
	MYELOSUPPRESSION	5	3.74 (1.52 - 9.19)	3.74 (9.54)	3.60 (1.47)	1.85 (0.11)
	MICROCYTIC ANAEMIA	4	4.10 (1.50 - 11.24)	4.10 (8.88)	3.93 (1.44)	1.98 (-0.03)
Metabolism and nutrition disorders	DEHYDRATION	111	1.58 (1.31 - 1.90)	1.58 (22.87)	1.56 (1.29)	0.64 (0.36)
	FEEDING DISORDER	25	2.29 (1.54 - 3.41)	2.29 (17.54)	2.25 (1.51)	1.17 (0.52)
	FLUID INTAKE REDUCED	10	3.65 (1.93 - 6.88)	3.64 (18.26)	3.52 (1.86)	1.81 (0.62)
	HYPONATRAEMIA	7	2.26 (1.07 - 4.80)	2.26 (4.79)	2.23 (1.05)	1.15 (-0.09)
	HYPERLIPIDAEMIA	6	6.73 (2.92 - 15.55)	6.73 (26.76)	6.24 (2.70)	2.64 (0.68)
	HYPERVOLAEMIA	3	6.95 (2.12 - 22.73)	6.95 (13.93)	6.42 (1.96)	2.68 (-0.09)
Reproductive system and breast disorders	SEMEN DISCOLOURATION	36	862.69 (265.65 - 2801.58)	861.47 (2380.13)	67.19 (20.69)	6.07 (3.92)
	AMENORRHOEA	15	7.33 (4.31 - 12.47)	7.33 (74.35)	6.74 (3.96)	2.75 (1.55)
	HEAVY MENSTRUAL BLEEDING	8	4.79 (2.34 - 9.79)	4.79 (22.46)	4.55 (2.22)	2.19 (0.70)
	PROSTATOMEGALY	6	2.52 (1.12 - 5.69)	2.52 (5.31)	2.47 (1.09)	1.30 (-0.09)
	INTERMENSTRUAL	3	7.18 (2.19 - 23.53)	7.18 (14.50)	6.62 (2.02)	2.73 (-0.08)

	BLEEDING					
Product issues	PRODUCT SOLUBILITY ABNORMAL	20	39.91 (23.10 - 68.96)	39.88 (487.40)	26.00 (15.05)	4.70 (2.82)
	DEVICE DISLOCATION	7	3.37 (1.58 - 7.20)	3.37 (11.16)	3.27 (1.53)	1.71 (0.30)
	PRODUCT LOT NUMBER ISSUE	4	7.56 (2.70 - 21.18)	7.56 (20.59)	6.93 (2.47)	2.79 (0.29)
	DEVICE LOOSENING	3	7.43 (2.26 - 24.38)	7.43 (15.12)	6.82 (2.08)	2.77 (-0.07)
Hepatobiliary disorders	PORTAL VEIN THROMBOSIS	9	5.39 (2.74 - 10.60)	5.38 (29.89)	5.08 (2.58)	2.34 (0.90)
	HEPATIC VEIN THROMBOSIS	3	11.97 (3.52 - 40.63)	11.96 (25.84)	10.40 (3.06)	3.38 (0.05)
	HEPATIC MASS	3	3.92 (1.23 - 12.52)	3.92 (6.18)	3.76 (1.18)	1.91 (-0.35)
Renal and urinary disorders	NEPHROLITHIASIS	75	1.49 (1.18 - 1.87)	1.49 (11.72)	1.48 (1.17)	0.56 (0.22)
	CHROMATURIA	13	2.48 (1.43 - 4.32)	2.48 (11.12)	2.43 (1.40)	1.28 (0.35)
	URETEROLITHIASIS	6	5.19 (2.27 - 11.89)	5.19 (18.93)	4.91 (2.14)	2.29 (0.51)
Immune system disorders	IMMUNODEFICIENCY	27	2.11 (1.44 - 3.10)	2.11 (15.40)	2.08 (1.42)	1.06 (0.45)
	IMMUNOSUPPRESSION	22	2.66 (1.74 - 4.07)	2.66 (21.96)	2.60 (1.70)	1.38 (0.67)
Social circumstances	IMPAIRED WORK ABILITY	21	1.76 (1.14 - 2.72)	1.76 (6.77)	1.74 (1.13)	0.80 (0.13)
	SOCIAL PROBLEM	19	6.32 (3.95 - 10.10)	6.31 (78.13)	5.89 (3.68)	2.56 (1.56)
	WALKING AID USER	8	2.40 (1.19 - 4.86)	2.40 (6.34)	2.36 (1.17)	1.24 (0.05)
Ear and labyrinth disorders	DEAFNESS UNILATERAL	6	2.50 (1.11 - 5.65)	2.50 (5.24)	2.45 (1.09)	1.29 (-0.09)
	AUDITORY DISORDER	3	6.95 (2.12 - 22.73)	6.95 (13.93)	6.42 (1.96)	2.68 (-0.09)

Table 5S. Top Preferred Terms by Age and Disease Indication Subgroups in FAERS Reports of Upadacitinib-Associated Adverse Events

Patients <50 Years	Patients ≥50Years	Ulcerative Colitis	Crohn’s Disease

n= 2,415		n= 2,275		n= 4,158		n= 4,635	
PT	n (%)	PT	n (%)	PT	n(%)	PT	n (%)
COLITIS ULCERATIVE	161 (6.7)	DIARRHOEA	150 (6.6)	CROHN'S DISEASE	388 (9.4)	COLITIS ULCERATIVE	492 (10.6)
CROHN'S DISEASE	146 (6.0)	FATIGUE	113 (5.0)	PRODUCT RESIDUE PRESENT	203 (4.9)	DRUG INEFFECTIVE	272 (5.9)
DRUG INEFFECTIVE	132 (5.5)	COLITIS ULCERATIVE	111 (4.9)	SURGERY	194 (4.7)	DIARRHOEA	210 (4.5)
ABDOMINAL PAIN	120 (5.0)	ARTHRALGIA	107 (4.7)	DIARRHOEA	189 (4.6)	ACNE	209 (4.5)
DIARRHOEA	106 (4.4)	DRUG INEFFECTIVE	89 (3.9)	DRUG INEFFECTIVE	183 (4.5)	FATIGUE	182 (3.9)
PYREXIA	99 (4.1)	PAIN	87 (3.8)	FATIGUE	149 (3.6)	ARTHRALGIA	170 (3.7)
FATIGUE	98 (4.1)	CROHN'S DISEASE	83 (3.6)	ABDOMINAL PAIN	146 (3.6)	PYREXIA	147 (3.2)
SURGERY	97 (4.0)	FALL	82 (3.6)	PAIN	133 (3.2)	PAIN	140 (3.0)
NAUSEA	95 (3.9)	PYREXIA	80 (3.5)	INTESTINAL OBSTRUCTION	132 (3.2)	NAUSEA	136 (2.9)
ACNE	93 (3.9)	ABDOMINAL PAIN	79 (3.5)	ACNE	128 (3.1)	ABDOMINAL PAIN	134 (2.9)
PRODUCT RESIDUE PRESENT	84 (3.5)	SURGERY	77 (3.4)	OFF LABEL USE	115 (2.8)	HAEMATOCHEZIA	133 (2.9)

OFF LABEL USE	83 (3.4)	NAUSEA	75 (3.3)	PYREXIA	114 (2.8)	SURGERY	131 (2.8)
COLECTOMY	82 (3.4)	ASTHENIA	69 (3.0)	NAUSEA	112 (2.7)	COLECTOMY	124 (2.7)
PAIN	75 (3.1)	ACNE	65 (2.9)	ILEOSTOMY	108 (2.6)	FREQUENT BOWEL MOVEMENTS	114 (2.5)
VOMITING	75 (3.1)	HEADACHE	62 (2.7)	ARTHRALGIA	99 (2.4)	HEADACHE	112 (2.4)
HAEMATOCHEZIA	72 (3.0)	DIZZINESS	58 (2.5)	VOMITING	98 (2.4)	ASTHENIA	109 (2.4)
FREQUENT BOWEL MOVEMENTS	62 (2.6)	PNEUMONIA	57 (2.5)	HEADACHE	95 (2.3)	OFF LABEL USE	105 (2.3)
HEADACHE	60 (2.5)	DYSPNOEA	57 (2.5)	MALAISE	94 (2.3)	PRODUCT RESIDUE PRESENT	102 (2.2)
ARTHRALGIA	59 (2.4)	URINARY TRACT INFECTION	54 (2.4)	ABDOMINAL PAIN UPPER	93 (2.3)	DYSPNOEA	97 (2.1)
ILEOSTOMY	57 (2.4)	CONSTIPATION	50 (2.2)	GASTROINTESTINAL DISORDER	92 (2.2)	CONSTIPATION	90 (1.9)
ABDOMINAL PAIN UPPER	56 (2.3)	COVID-19	50 (2.2)	DIZZINESS	72 (1.8)	DIZZINESS	90 (1.9)
ASTHENIA	51 (2.1)	MALAISE	48 (2.1)	FISTULA	70 (1.7)	HAEMORRHAGE	88 (1.9)
HAEMORRHAGE	49 (2.0)	DEATH	47 (2.1)	ANAEMIA	69 (1.7)	COVID-19	84 (1.8)
INTESTINAL OBSTRUCTION	49 (2.0)	PAIN IN EXTREMITY	47 (2.1)	HAEMOGLOBIN DECREASED	68 (1.7)	FLATULENCE	81 (1.7)
COLITIS	47 (1.9)	VOMITING	47 (2.1)	DEHYDRATION	65 (1.6)	FALL	79 (1.7)
GASTROINT	47	HAEMATOC	47	INTESTINAL	64	VOMITING	79 (1.7)

ESTINAL DISORDER	(1.9)	HEZIA	(2.1)	RESECTION	(1.6)		
MALAISE	47 (1.9)	CHILLS	45 (2.0)	DEATH	64 (1.6)	RASH	79 (1.7)
DIZZINESS	43 (1.8)	PULMONARY EMBOLISM	42 (1.8)	INFECTION	64 (1.6)	COLITIS	75 (1.6)
CONSTIPATION	42 (1.7)	ANAEMIA	41 (1.8)	DYSPNOEA	64 (1.6)	ABDOMINAL DISTENSION	72 (1.6)
FISTULA	41 (1.7)	ILLNESS	41 (1.8)	COLECTOMY	63 (1.5)	BLOOD CHOLESTEROL INCREASED	71 (1.5)
INFLAMMATION	39 (1.6)	ABDOMINAL PAIN UPPER	41 (1.8)	FALL	61 (1.5)	NASOPHARYNGITIS	71 (1.5)
THERAPEUTIC PRODUCT EFFECT INCOMPLETE	38 (1.6)	RASH	41 (1.8)	HAEMATOCHEZIA	59 (1.4)	PAIN IN EXTREMITY	71 (1.5)
MATERNAL EXPOSURE DURING PREGNANCY	37 (1.5)	ABDOMINAL DISTENSION	41 (1.8)	ILLNESS	59 (1.4)	HERPES ZOSTER	70 (1.5)
DYSPNOEA	37 (1.5)	BACK PAIN	40 (1.8)	ASTHENIA	58 (1.4)	UNEVALUABLE EVENT	69 (1.5)
ABDOMINAL DISTENSION	36 (1.5)	LOSS OF CONSCIOUSNESS	40 (1.8)	SMALL INTESTINAL OBSTRUCTION	57 (1.4)	PNEUMONIA	67 (1.4)
CLOSTRIDIUM DIFFICILE INFECTION	36 (1.5)	FREQUENT BOWEL MOVEMENTS	38 (1.7)	SEPSIS	57 (1.4)	CLOSTRIDIUM DIFFICILE INFECTION	66 (1.4)
CHILLS	35 (1.4)	COLECTOMY	38 (1.7)	WEIGHT INCREASED	56 (1.4)	ILLNESS	66 (1.4)
NASOPHARYNGITIS	35 (1.4)	DEHYDRATION	38 (1.7)	ABDOMINAL DISTENSION	55 (1.3)	URINARY TRACT INFECTION	65 (1.4)
INTESTINAL RESECTION	35 (1.4)	NASOPHARYNGITIS	37 (1.6)	RASH	55 (1.3)	THERAPEUTIC PRODUCT EFFECT	64 (1.4)

						INCOMPLETE	
SEPSIS	34 (1.4)	SEPSIS	37 (1.6)	THERAPEUTIC PRODUCT EFFECT INCOMPLETE	54 (1.3)	INSOMNIA	63 (1.4)
UNEVALUABLE EVENT	34 (1.4)	INTESTINAL OBSTRUCTION	37 (1.6)	PNEUMONIA	54 (1.3)	DECREASED APPETITE	62 (1.3)
DEHYDRATION	34 (1.4)	BLOOD CHOLESTEROL INCREASED	37 (1.6)	OSTOMY BAG PLACEMENT	53 (1.3)	WEIGHT INCREASED	61 (1.3)
COUGH	34 (1.4)	COUGH	36 (1.6)	HERPES ZOSTER	52 (1.3)	INFLAMMATION	60 (1.3)
INFECTION	31 (1.3)	FLATULENCE	36 (1.6)	STOMA CREATION	52 (1.3)	MALAISE	60 (1.3)
WEIGHT INCREASED	30 (1.2)	INFECTION	35 (1.5)	COLITIS	51 (1.2)	CHILLS	60 (1.3)
HAEMOGLOBIN DECREASED	29 (1.2)	PERIPHERAL SWELLING	35 (1.5)	ABSCESS	51 (1.2)	DEFAECATION URGENCY	59 (1.3)
INSOMNIA	29 (1.2)	PRODUCT RESIDUE PRESENT	34 (1.5)	CONSTIPATION	50 (1.2)	COUGH	58 (1.3)
ANAEMIA	29 (1.2)	DECREASED APPETITE	34 (1.5)	HAEMORRHAGE	48 (1.2)	MUSCLE SPASMS	57 (1.2)
WEIGHT DECREASED	28 (1.2)	COLITIS	34 (1.5)	INFLAMMATION	47 (1.1)	DEATH	57 (1.2)
DEFAECATION URGENCY	28 (1.2)	CATARACT	33 (1.5)	CHILLS	47 (1.1)	ANAEMIA	57 (1.2)
PAIN IN EXTREMITY	27 (1.1)	WEIGHT INCREASED	33 (1.5)	LOSS OF CONSCIOUSNESS	46 (1.1)	PULMONARY EMBOLISM	56 (1.2)
COVID-19	27 (1.1)	HAEMOGLOBIN DECREASED	33 (1.5)	WEIGHT DECREASED	46 (1.1)	ABDOMINAL PAIN UPPER	55 (1.2)
DECREASED APPETITE	26 (1.1)	MUSCLE SPASMS	31 (1.4)	MALABSORPTION	45 (1.1)	THERAPEUTIC PRODUCT EFFECT DECREASED	53 (1.1)
LOSS OF CONSCIOUS	26 (1.1)	HEPATIC ENZYME	30 (1.3)	FREQUENT BOWEL	45 (1.1)	BACK PAIN	52 (1.1)

NESS		INCREASED		MOVEMENTS			
FLATULENCE	26 (1.1)	GASTROINTESTINAL DISORDER	29 (1.3)	NEPHROLITHIASIS	44 (1.1)	LOSS OF CONSCIOUSNESS	50 (1.1)
ILLNESS	26 (1.1)	INFLUENZA	29 (1.3)	BACK PAIN	44 (1.1)	PERIPHERAL SWELLING	47 (1.0)
HERPES ZOSTER	26 (1.1)	PULMONARY THROMBOSIS	26 (1.1)	THERAPEUTIC PRODUCT EFFECT DECREASED	43 (1.0)	DEHYDRATION	47 (1.0)
PNEUMONIA	25 (1.0)	HAEMORRHAGE	26 (1.1)	COVID-19	43 (1.0)	INFECTION	47 (1.0)
RASH	25 (1.0)	NEPHROLITHIASIS	26 (1.1)	NASOPHARYNGITIS	42 (1.0)	SEPSIS	46 (1.0)
DEATH	25 (1.0)	INFLAMMATION	26 (1.1)	HOSPITALISATION	41 (1.0)	ILEOSTOMY	46 (1.0)
RECTAL HAEMORRHAGE	24 (1.0)	ARTHRITIS	26 (1.1)	UNEVALUABLE EVENT	41 (1.0)	GASTROINTESTINAL DISORDER	45 (1.0)
INFLUENZA	24 (1.0)	KNEE ARTHROPLASTY	25 (1.1)	PERIPHERAL SWELLING	40 (1.0)	CATARACT	44 (0.9)
ABSCESS	23 (1.0)	ILEOSTOMY	25 (1.1)	INFLUENZA	40 (1.0)	RECTAL HAEMORRHAGE	44 (0.9)
HOSPITALISATION	23 (1.0)	MYALGIA	25 (1.1)	FEELING ABNORMAL	39 (0.9)	HAEMOGLOBIN DECREASED	44 (0.9)
THERAPEUTIC PRODUCT EFFECT DECREASED	23 (1.0)	OFF LABEL USE	25 (1.1)	URINARY TRACT INFECTION	38 (0.9)	INFLUENZA	42 (0.9)
MALABSORPTION	22 (0.9)	HERPES ZOSTER	25 (1.1)	MATERNAL EXPOSURE DURING PREGNANCY	36 (0.9)	CONDITION AGGRAVATED	42 (0.9)
NEPHROLITHIASIS	21 (0.9)	DEFAECATION URGENCY	24 (1.1)	INTESTINAL OPERATION	36 (0.9)	MATERNAL EXPOSURE DURING PREGNANCY	40 (0.9)
INTESTINAL PERFORATION	21 (0.9)	CEREBROVASCULAR ACCIDENT	24 (1.1)	HEPATIC ENZYME INCREASED	34 (0.8)	CHEST PAIN	40 (0.9)

GASTROINTESTINAL HAEMORRHAGE	19 (0.8)	CLOSTRIDIUM DIFFICILE INFECTION	24 (1.1)	FLATULENCE	33 (0.8)	WEIGHT DECREASED	38 (0.8)
SMALL INTESTINAL OBSTRUCTION	19 (0.8)	WEIGHT DECREASED	23 (1.0)	COUGH	33 (0.8)	HYPERTENSION	37 (0.8)
CHEST PAIN	19 (0.8)	UNEVALUABLE EVENT	23 (1.0)	PAIN IN EXTREMITY	33 (0.8)	PRURITUS	37 (0.8)
PARAESTHESIA	19 (0.8)	BASAL CELL CARCINOMA	23 (1.0)	INTESTINAL PERFORATION	33 (0.8)	ARTHRITIS	35 (0.8)
APPENDICITIS	19 (0.8)	CHEST PAIN	23 (1.0)	HERNIA	32 (0.8)	THROMBOSIS	35 (0.8)
PERIPHERAL SWELLING	19 (0.8)	INTESTINAL RESECTION	23 (1.0)	INTESTINAL STENOSIS	32 (0.8)	HOSPITALISATION	34 (0.7)
GASTROINTESTINAL INFLAMMATION	19 (0.8)	HOSPITALISATION	22 (1.0)	CHEST PAIN	32 (0.8)	PULMONARY THROMBOSIS	34 (0.7)
OSTOMY BAG PLACEMENT	18 (0.7)	INSOMNIA	22 (1.0)	DECREASED APPETITE	31 (0.8)	MYALGIA	33 (0.7)
FEELING ABNORMAL	18 (0.7)	HYPOTENSION	22 (1.0)	GASTROINTESTINAL INFLAMMATION	31 (0.8)	CEREBROVASCULAR ACCIDENT	33 (0.7)
MUSCLE SPASMS	18 (0.7)	THERAPEUTIC PRODUCT EFFECT INCOMPLETE	21 (0.9)	THROMBOSIS	31 (0.8)	HEPATIC ENZYME INCREASED	33 (0.7)
INTESTINAL OPERATION	18 (0.7)	THROMBOSIS	21 (0.9)	CLOSTRIDIUM DIFFICILE INFECTION	30 (0.7)	GASTROINTESTINAL HAEMORRHAGE	32 (0.7)
OROPHARYNGEAL PAIN	17 (0.7)	DIVERTICULITIS	20 (0.9)	OBSTRUCTION	29 (0.7)	MUCOUS STOOLS	32 (0.7)
DIARRHOEA HAEMORRHAGIC	17 (0.7)	ORAL HERPES	20 (0.9)	ADVERSE DRUG REACTION	28 (0.7)	NEPHROLITHIASIS	31 (0.7)
STRESS	17 (0.7)	JOINT SWELLING	19 (0.8)	STENOSIS	28 (0.7)	ALOPECIA	30 (0.6)

PULMONARY EMBOLISM	17 (0.7)	SKIN CANCER	19 (0.8)	ABDOMINAL DISCOMFORT	28 (0.7)	HYPOTENSION	29 (0.6)
C-REACTIVE PROTEIN INCREASED	16 (0.7)	ADVERSE DRUG REACTION	19 (0.8)	PULMONARY EMBOLISM	28 (0.7)	ADVERSE DRUG REACTION	28 (0.6)
BLOOD CHOLESTEROL INCREASED	16 (0.7)	SINUSITIS	19 (0.8)	MUSCLE SPASMS	28 (0.7)	CROHN'S DISEASE	28 (0.6)
CONDITION AGGRAVATED	16 (0.7)	GASTROINTESTINAL HAEMORRHAGE	19 (0.8)	COLOSTOMY	27 (0.7)	OROPHARYNGEAL PAIN	28 (0.6)
LARGE INTESTINE PERFORATION	16 (0.7)	MYOCARDIAL INFARCTION	18 (0.8)	GASTROINTESTINAL HAEMORRHAGE	27 (0.7)	KNEE ARTHROPLASTY	27 (0.6)
THROMBOSIS	16 (0.7)	RECTAL HAEMORRHAGE	18 (0.8)	SMALL INTESTINAL RESECTION	25 (0.6)	CARDIAC DISORDER	27 (0.6)
URINARY TRACT INFECTION	15 (0.6)	CARDIAC DISORDER	18 (0.8)	ANAL FISTULA	25 (0.6)	ABDOMINAL DISCOMFORT	27 (0.6)
FAECAL CALPROTECTIN INCREASED	15 (0.6)	GAIT DISTURBANCE	18 (0.8)	CATARACT	25 (0.6)	NEOPLASM MALIGNANT	26 (0.6)
DEEP VEIN THROMBOSIS	15 (0.6)	ABDOMINAL DISCOMFORT	18 (0.8)	BLOOD CHOLESTEROL INCREASED	24 (0.6)	DEEP VEIN THROMBOSIS	26 (0.6)
STOMA CREATION	15 (0.6)	STOMA CREATION	18 (0.8)	GAIT DISTURBANCE	23 (0.6)	HERNIA	26 (0.6)
PRURITUS	15 (0.6)	HYPERTENSION	18 (0.8)	NEOPLASM MALIGNANT	23 (0.6)	VISION BLURRED	26 (0.6)
MIGRAINE	14 (0.6)	PROCEDURAL PAIN	17 (0.7)	STRESS	23 (0.6)	HYPOAESTHESIA	25 (0.5)
ANXIETY	14 (0.6)	PRURITUS	17 (0.7)	ENTERITIS	23 (0.6)	FEELING ABNORMAL	25 (0.5)
ANAL FISTULA	14 (0.6)	SMALL INTESTINAL OBSTRUCTI	17 (0.7)	SWELLING	21 (0.5)	JOINT SWELLING	25 (0.5)

		ON					
SYNCOPE	14 (0.6)	KNEE OPERATION	17 (0.7)	ANAL ABSCESS	21 (0.5)	STRESS	24 (0.5)
INTESTINAL STENOSIS	14 (0.6)	LARGE INTESTINE POLYP	17 (0.7)	CONTUSION	21 (0.5)	SYNCOPE	24 (0.5)
ABDOMINAL ABSCESS	14 (0.6)	HERNIA	16 (0.7)	ERYTHEMA	21 (0.5)	KNEE OPERATION	24 (0.5)
DEPRESSION	14 (0.6)	ATRIAL FIBRILLATION	16 (0.7)	BLOOD IRON DECREASED	21 (0.5)	ERYTHEMA	24 (0.5)
HYPOAESTHESIA	14 (0.6)	CONTUSION	16 (0.7)	RECTAL HAEMORRHAGE	21 (0.5)	BASAL CELL CARCINOMA	24 (0.5)
COLOSTOMY	14 (0.6)	FEELING ABNORMAL	16 (0.7)	ARTHRITIS	21 (0.5)	SPINAL OPERATION	24 (0.5)
VISION BLURRED	14 (0.6)	HIP SURGERY	16 (0.7)	PULMONARY THROMBOSIS	21 (0.5)	APPENDICITIS	24 (0.5)
BACK PAIN	14 (0.6)	THERAPEUTIC PRODUCT EFFECT DECREASED	15 (0.7)	DEFAECATION URGENCY	20 (0.5)	DEPRESSION	24 (0.5)
ABDOMINAL DISCOMFORT	13 (0.5)	BALANCE DISORDER	15 (0.7)	INFLAMMATORY MARKER INCREASED	20 (0.5)	DIVERTICULITIS	23 (0.5)
STENOSIS	13 (0.5)	HEAD INJURY	15 (0.7)	PRURITUS	20 (0.5)	MUSCULAR WEAKNESS	23 (0.5)
ANAL ABSCESS	13 (0.5)	STRESS	15 (0.7)	DYSPEPSIA	20 (0.5)	VISUAL IMPAIRMENT	22 (0.5)
BLOOD IRON DECREASED	13 (0.5)	TRANSIENT ISCHAEMIC ATTACK	15 (0.7)	PARAESTHESIA	20 (0.5)	COLON CANCER	22 (0.5)
SMALL INTESTINAL RESECTION	13 (0.5)	PALPITATIONS	15 (0.7)	HYPERTENSION	20 (0.5)	PARAESTHESIA	22 (0.5)
VISUAL IMPAIRMENT	13 (0.5)	HIP ARTHROPLASTY	14 (0.6)	ILEAL STENOSIS	19 (0.5)	HYPERHIDROSIS	22 (0.5)
FALL	13 (0.5)	GASTROINTESTINAL PAIN	14 (0.6)	LARGE INTESTINE PERFORATION	19 (0.5)	HIP ARTHROPLASTY	22 (0.5)

UPPER RESPIRATORY TRACT INFECTION	13 (0.5)	SYNCOPE	14 (0.6)	BLOOD POTASSIUM DECREASED	19 (0.5)	SEMEN DISCOLOURATION	22 (0.5)
VIRAL INFECTION	13 (0.5)	MALIGNANT MELANOMA	14 (0.6)	VIRAL INFECTION	19 (0.5)	SKIN CANCER	22 (0.5)
ROAD TRAFFIC ACCIDENT	12 (0.5)	SPINAL OPERATION	14 (0.6)	OROPHARYNGEAL PAIN	19 (0.5)	ORAL HERPES	22 (0.5)
ENTERITIS	12 (0.5)	ERYTHEMA	14 (0.6)	RENAL FAILURE	19 (0.5)	SINUSITIS	21 (0.5)
FOLLICULITIS	12 (0.5)	GAIT INABILITY	14 (0.6)	POST PROCEDURAL COMPLICATION	19 (0.5)	LARGE INTESTINAL HAEMORRHAGE	21 (0.5)
LARGE INTESTINAL HAEMORRHAGE	12 (0.5)	VISION BLURRED	14 (0.6)	DEEP VEIN THROMBOSIS	19 (0.5)	LIVER FUNCTION TEST INCREASED	21 (0.5)
ERYTHEMA	12 (0.5)	OROPHARYNGEAL PAIN	13 (0.6)	ABDOMINAL ABSCESS	19 (0.5)	SEIZURE	21 (0.5)
HYSTERECTOMY	12 (0.5)	DEEP VEIN THROMBOSIS	13 (0.6)	LARGE INTESTINAL STENOSIS	18 (0.4)	NIGHT SWEATS	21 (0.5)
HERNIA	12 (0.5)	MALABSORPTION	13 (0.6)	INTESTINAL ANASTOMOSIS	18 (0.4)	LARGE INTESTINE POLYP	20 (0.4)
INFLAMMATORY MARKER INCREASED	12 (0.5)	THERAPY CESSATION	13 (0.6)	MYALGIA	18 (0.4)	BALANCE DISORDER	20 (0.4)
HYPOTENSION	11 (0.5)	FISTULA	13 (0.6)	FAECAL CALPROTECTIN INCREASED	18 (0.4)	INTERVERTEBRAL DISC PROTRUSION	20 (0.4)
PANCREATITIS	11 (0.5)	INFLAMMATORY BOWEL DISEASE	13 (0.6)	GASTROINTESTINAL PAIN	18 (0.4)	CYTOMEGALOVIRUS INFECTION	20 (0.4)
ILEAL STENOSIS	11 (0.5)	INTERSTITIAL LUNG DISEASE	13 (0.6)	VISION BLURRED	18 (0.4)	PALPITATIONS	20 (0.4)
MUCOUS STOOLS	11 (0.5)	FAECAL CALPROTEC	13 (0.6)	DISCOMFORT	18 (0.4)	DIARRHOEA HAEMORRH	20 (0.4)

		TIN INCREASED				AGIC	
SEIZURE	11 (0.5)	FOOT FRACTURE	13 (0.6)	BASAL CELL CARCINOMA	18 (0.4)	CONTUSION	20 (0.4)
SEMEN DISCOLOURATION	11 (0.5)	WHITE BLOOD CELL COUNT INCREASED	13 (0.6)	IMPAIRED WORK ABILITY	17 (0.4)	IMMUNODEFICIENCY	19 (0.4)
HYPERHIDROSIS	11 (0.5)	VISUAL IMPAIRMENT	12 (0.5)	C-REACTIVE PROTEIN INCREASED	17 (0.4)	C-REACTIVE PROTEIN INCREASED	19 (0.4)
HYPERTENSION	11 (0.5)	CONDITION AGGRAVATED	12 (0.5)	SINUSITIS	17 (0.4)	PROCTALGIA	19 (0.4)
DYSPEPSIA	11 (0.5)	IMMUNOSUPPRESSION	12 (0.5)	CONDITION AGGRAVATED	17 (0.4)	FAECAL CALPROTECTIN INCREASED	19 (0.4)
MYALGIA	11 (0.5)	OSTEOARTHRITIS	12 (0.5)	VISUAL IMPAIRMENT	17 (0.4)	MIGRAINE	19 (0.4)
INTESTINAL HAEMORRHAGE	11 (0.5)	PROSTATE CANCER	12 (0.5)	SWELLING FACE	17 (0.4)	STOMA CREATION	18 (0.4)
COLON CANCER	11 (0.5)	SPINAL FRACTURE	12 (0.5)	GASTROINTESTINAL INFECTION	17 (0.4)	ATRIAL FIBRILLATION	18 (0.4)
POST PROCEDURAL COMPLICATION	11 (0.5)	BLOOD POTASSIUM DECREASED	12 (0.5)	SOCIAL PROBLEM	17 (0.4)	ANXIETY	18 (0.4)
WHITE BLOOD CELL COUNT INCREASED	11 (0.5)	NECK PAIN	12 (0.5)	WHITE BLOOD CELL COUNT INCREASED	17 (0.4)	MALIGNANT MELANOMA	18 (0.4)
ABORTION INDUCED	11 (0.5)	HYPERHIDROSIS	12 (0.5)	ORAL HERPES	17 (0.4)	GASTROINTESTINAL PAIN	18 (0.4)
OBSTRUCTION	11 (0.5)	VERTIGO	12 (0.5)	ROAD TRAFFIC ACCIDENT	16 (0.4)	ACNE CYSTIC	18 (0.4)
NOROVIRUS	10	CYTOMEGA	11	PALPITATION	16	TRANSIENT	18 (0.4)

INFECTION	(0.4)	LOVIRUS INFECTION	(0.5)	S	(0.4)	ISCHAEMIC ATTACK	
TACHYCARDIA	10 (0.4)	RED BLOOD CELL COUNT DECREASED	11 (0.5)	HYPERHIDROSIS	16 (0.4)	UPPER RESPIRATORY TRACT INFECTION	18 (0.4)
SWELLING FACE	10 (0.4)	SWELLING FACE	11 (0.5)	GASTROINTESTINAL SCARRING	16 (0.4)	COLECTOMY TOTAL	18 (0.4)
ALOPECIA	10 (0.4)	CHOLECYSTECTOMY	11 (0.5)	CHEST DISCOMFORT	16 (0.4)	VIRAL INFECTION	18 (0.4)
ABDOMINAL PAIN LOWER	10 (0.4)	HYPOPHAGIA	11 (0.5)	SYNCOPE	16 (0.4)	RESPIRATORY DISORDER	17 (0.4)
APPENDICECTOMY	10 (0.4)	PNEUMOCYSTIS JIROVECII PNEUMONIA	11 (0.5)	TREMOR	16 (0.4)	SKIN DISORDER	17 (0.4)
HEART RATE INCREASED	10 (0.4)	RASH PAPULAR	11 (0.5)	MEMORY IMPAIRMENT	16 (0.4)	MUSCULOSKELETAL STIFFNESS	17 (0.4)
AMENORRHOEA	10 (0.4)	GASTROOESOPHAGEAL REFLUX DISEASE	11 (0.5)	SCAR	16 (0.4)	HEART RATE INCREASED	17 (0.4)
ANAL FISSURE	10 (0.4)	VITREOUS FLOATERS	11 (0.5)	PRODUCT SOLUBILITY ABNORMAL	16 (0.4)	APPENDICECTOMY	17 (0.4)
PROCTALGIA	10 (0.4)	IMMUNODEFICIENCY	11 (0.5)	FAECES DISCOLOURED	15 (0.4)	FOLLICULITIS	17 (0.4)
PLATELET COUNT INCREASED	9 (0.4)	PLATELET COUNT DECREASED	11 (0.5)	THERAPY INTERRUPTED	15 (0.4)	MALABSORPTION	17 (0.4)
ORAL HERPES	9 (0.4)	BLOOD CREATINE PHOSPHOKINASE INCREASED	11 (0.5)	WRONG TECHNIQUE IN PRODUCT USAGE PROCESS	15 (0.4)	GAIT DISTURBANCE	17 (0.4)
GASTROENTERITIS VIRAL	9 (0.4)	MUSCULOSKELETAL DISORDER	10 (0.4)	SKIN DISORDER	15 (0.4)	INFLAMMATORY MARKER INCREASED	16 (0.3)

CHOLELITHIASIS	9 (0.4)	BACK DISORDER	10 (0.4)	HYPOTENSION	15 (0.4)	POLYP	16 (0.3)
DRY SKIN	9 (0.4)	ALOPECIA	10 (0.4)	SMALL INTESTINAL PERFORATION	15 (0.4)	SWELLING FACE	16 (0.3)
HYPOPHAGIA	9 (0.4)	NASAL CONGESTION	10 (0.4)	RED BLOOD CELL COUNT DECREASED	15 (0.4)	DYSPEPSIA	16 (0.3)
BLOOD POTASSIUM DECREASED	9 (0.4)	NEOPLASM MALIGNANT	10 (0.4)	INSOMNIA	14 (0.3)	FISTULA	16 (0.3)
ILEOCAECAL RESECTION	9 (0.4)	SWELLING	10 (0.4)	INTERNAL HAEMORRHAGE	14 (0.3)	LIPIDS INCREASED	16 (0.3)
INTESTINAL ANASTOMOSIS	9 (0.4)	BLOOD IRON DECREASED	10 (0.4)	HAEMOGLOBIN ABNORMAL	14 (0.3)	LARGE INTESTINE PERFORATION	16 (0.3)
GAIT DISTURBANCE	9 (0.4)	SKIN DISORDER	10 (0.4)	ABSCESS INTESTINAL	14 (0.3)	LOW DENSITY LIPOPROTEIN INCREASED	16 (0.3)
THERAPY INTERRUPTED	9 (0.4)	ROTATOR CUFF SYNDROME	10 (0.4)	ILEOCAECAL RESECTION	14 (0.3)	MYOCARDIAL INFARCTION	16 (0.3)
ACNE CYSTIC	9 (0.4)	FEEDING DISORDER	10 (0.4)	JOINT SWELLING	14 (0.3)	HIP SURGERY	15 (0.3)
SCAR	9 (0.4)	MUCOUS STOOLS	10 (0.4)	INTESTINAL HAEMORRHAGE	14 (0.3)	HYPOPHAGIA	15 (0.3)
CHEST DISCOMFORT	8 (0.3)	VIRAL INFECTION	10 (0.4)	BLOOD PRESSURE INCREASED	14 (0.3)	BLOOD IRON DECREASED	15 (0.3)
IMMUNODEFICIENCY	8 (0.3)	C-REACTIVE PROTEIN INCREASED	10 (0.4)	IMMUNOSUPPRESSION	14 (0.3)	PROSTATE CANCER	15 (0.3)
TRANSFUSION	8 (0.3)	PROCTALGIA	10 (0.4)	DRY SKIN	13 (0.3)	VITREOUS FLOATERS	15 (0.3)
GASTROINTESTINAL INFECTION	8 (0.3)	LARGE INTESTINAL STENOSIS	10 (0.4)	ANXIETY	13 (0.3)	BLOOD CREATINE PHOSPHOKINASE	15 (0.3)

						INCREASED	
COLON OPERATION	8 (0.3)	LARGE INTESTINAL OBSTRUCTION	10 (0.4)	FEEDING DISORDER	13 (0.3)	INTESTINAL HAEMORRHAGE	15 (0.3)
GASTROINTESTINAL PAIN	8 (0.3)	THERAPY INTERRUPTED	10 (0.4)	HEAD INJURY	13 (0.3)	IMPAIRED HEALING	15 (0.3)
LYMPHADENOPATHY	8 (0.3)	DYSPEPSIA	10 (0.4)	PANCREATITIS	13 (0.3)	INTESTINAL PERFORATION	15 (0.3)
CYTOMEGALOVIRUS COLITIS	8 (0.3)	RETINAL DETACHMENT	10 (0.4)	FULL BLOOD COUNT DECREASED	13 (0.3)	CELLULITIS	15 (0.3)
SPINAL OPERATION	8 (0.3)	UPPER LIMB FRACTURE	10 (0.4)	SEIZURE	13 (0.3)	PROCEDURAL PAIN	15 (0.3)
CELLULITIS	8 (0.3)	INFLUENZA LIKE ILLNESS	10 (0.4)	COLITIS ULCERATIVE	13 (0.3)	WHITE BLOOD CELL COUNT INCREASED	15 (0.3)
DRUG HYPERSENSITIVITY	8 (0.3)	HIP FRACTURE	10 (0.4)	SUICIDAL IDEATION	13 (0.3)	NECK PAIN	15 (0.3)
BRONCHITIS	8 (0.3)	OSTOMY BAG PLACEMENT	10 (0.4)	WHITE BLOOD CELL COUNT DECREASED	13 (0.3)	INTESTINAL OBSTRUCTION	15 (0.3)
ULCER	8 (0.3)	INFLAMMATORY MARKER INCREASED	10 (0.4)	SKIN CANCER	13 (0.3)	GAIT INABILITY	15 (0.3)
OEDEMA PERIPHERAL	8 (0.3)	HYPOAESTHESIA	9 (0.4)	MYOCARDIAL INFARCTION	13 (0.3)	HYPERSENSITIVITY	15 (0.3)
ALANINE AMINOTRANSFERASE INCREASED	8 (0.3)	MUSCULOSKELETAL STIFFNESS	9 (0.4)	DEPRESSION	13 (0.3)	WHITE BLOOD CELL COUNT DECREASED	15 (0.3)
CEREBROVASCULAR ACCIDENT	8 (0.3)	STOMATITIS	9 (0.4)	ATRIAL FIBRILLATION	12 (0.3)	INTERSTITIAL LUNG DISEASE	15 (0.3)
ABSCESS INTESTINAL	8 (0.3)	DRUG HYPERSENSITIVITY	9 (0.4)	MIGRAINE	12 (0.3)	RHINORRHOEA	15 (0.3)

FAECES DISCOLOURED	8 (0.3)	SHOULDER OPERATION	9 (0.4)	PROCEDURAL PAIN	12 (0.3)	ROTATOR CUFF SYNDROME	14 (0.3)
BLOOD CREATINE PHOSPHOKINASE INCREASED	7 (0.3)	CELLULITIS	9 (0.4)	NECK PAIN	12 (0.3)	LYMPHADENOPATHY	14 (0.3)
ESCHERICHIA INFECTION	7 (0.3)	COLON CANCER	9 (0.4)	HYPOAESTHESIA	12 (0.3)	OSTOMY BAG PLACEMENT	14 (0.3)
CHOLECYSTECTOMY	7 (0.3)	OBSTRUCTION	9 (0.4)	INFLAMMATORY BOWEL DISEASE	12 (0.3)	DYSPHAGIA	14 (0.3)
HYPERSENSITIVITY	7 (0.3)	FAECES DISCOLOURED	9 (0.4)	CHOLECYSTECTOMY	12 (0.3)	CHEST DISCOMFORT	14 (0.3)
BRAIN FOG	7 (0.3)	WHITE BLOOD CELL COUNT DECREASED	9 (0.4)	DRUG HYPERSENSITIVITY	12 (0.3)	LIVER DISORDER	14 (0.3)
DERMATITIS ACNEIFORM	7 (0.3)	ORAL CANDIDIASIS	9 (0.4)	ACUTE KIDNEY INJURY	12 (0.3)	ASPARTATE AMINOTRANSFERASE INCREASED	14 (0.3)
SOCIAL PROBLEM	7 (0.3)	TOOTH EXTRACTION	9 (0.4)	SPINAL OPERATION	12 (0.3)	PNEUMOCYSTIS JIROVECII PNEUMONIA	14 (0.3)
SKIN DISORDER	7 (0.3)	CYSTITIS	9 (0.4)	SEMEN DISCOLOURATION	12 (0.3)	PANCREATITIS	14 (0.3)
GAIT INABILITY	7 (0.3)	RENAL FAILURE	9 (0.4)	GASTROOESOPHAGEAL REFLUX DISEASE	12 (0.3)	TACHYCARDIA	13 (0.3)
MALIGNANT MELANOMA	7 (0.3)	ACUTE KIDNEY INJURY	9 (0.4)	PLATELET COUNT INCREASED	12 (0.3)	HIP FRACTURE	13 (0.3)
DYSCHEZIA	7 (0.3)	INTESTINAL PERFORATION	9 (0.4)	CEREBROVASCULAR ACCIDENT	12 (0.3)	ROAD TRAFFIC ACCIDENT	13 (0.3)
PNEUMOTH	7 (0.3)	CHEST	9 (0.4)	THERAPY	12	BLINDNESS	13 (0.3)

ORAX		DISCOMFORT		CESSATION	(0.3)		
FEEDING DISORDER	7 (0.3)	SQUAMOUS CELL CARCINOMA OF SKIN	9 (0.4)	BRAIN FOG	12 (0.3)	BRONCHITIS	13 (0.3)
COLECTOMY TOTAL	7 (0.3)	MUSCULAR WEAKNESS	9 (0.4)	ALOPECIA	12 (0.3)	INFLAMMATORY BOWEL DISEASE	13 (0.3)
INTERVERTEBRAL DISC PROTRUSION	7 (0.3)	INCORRECT DOSE ADMINISTERED	9 (0.4)	RASH PAPULAR	12 (0.3)	SPINAL FRACTURE	13 (0.3)
MENTAL DISORDER	7 (0.3)	FULL BLOOD COUNT DECREASED	9 (0.4)	PROCTALGIA	12 (0.3)	NEUTROPENIA	13 (0.3)
LARGE INTESTINAL STENOSIS	7 (0.3)	ROSACEA	9 (0.4)	ABDOMINAL PAIN LOWER	12 (0.3)	BLOOD PRESSURE INCREASED	13 (0.3)
CONTUSION	7 (0.3)	ROAD TRAFFIC ACCIDENT	9 (0.4)	TRANSIENT ISCHAEMIC ATTACK	12 (0.3)	FOOT FRACTURE	13 (0.3)
LARGE INTESTINAL ULCER	7 (0.3)	SEIZURE	9 (0.4)	BLOOD CREATININE INCREASED	11 (0.3)	GASTROINTESTINAL INFLAMMATION	13 (0.3)
PRODUCT DISPENSING ERROR	7 (0.3)	MEMORY IMPAIRMENT	9 (0.4)	COLON CANCER	11 (0.3)	HAEMORRHOIDS	13 (0.3)
HAEMORRHOIDS	7 (0.3)	GASTROINTESTINAL INFLAMMATION	8 (0.4)	HAEMORRHOIDS	11 (0.3)	BREAST CANCER FEMALE	13 (0.3)
WHITE BLOOD CELL COUNT DECREASED	7 (0.3)	DYSPHAGIA	8 (0.4)	ANAL FISSURE	11 (0.3)	NASAL CONGESTION	12 (0.3)
PROCTECTOMY	7 (0.3)	BLOOD MAGNESIUM DECREASED	8 (0.4)	GAIT INABILITY	11 (0.3)	OXYGEN SATURATION DECREASED	12 (0.3)
PROCEDURAL PAIN	7 (0.3)	GENERAL PHYSICAL	8 (0.4)	UPPER RESPIRATOR	11 (0.3)	FULL BLOOD COUNT	12 (0.3)

		HEALTH DETERIORATION		Y TRACT INFECTION		DECREASED	
CYTOMEGALOVIRUS INFECTION	7 (0.3)	LOCALISED INFECTION	8 (0.4)	LETHARGY	11 (0.3)	SQUAMOUS CELL CARCINOMA OF SKIN	12 (0.3)
PALPITATIONS	7 (0.3)	LARGE INTESTINAL HAEMORRHAGE	8 (0.4)	SMALL INTESTINAL HAEMORRHAGE	11 (0.3)	RASH PAPULAR	12 (0.3)
SUICIDAL IDEATION	7 (0.3)	SCAR	8 (0.4)	RENAL DISORDER	11 (0.3)	HYSTERECTOMY	12 (0.3)
ECZEMA	7 (0.3)	ANAL INCONTINENCE	8 (0.4)	KNEE OPERATION	11 (0.3)	MOBILITY DECREASED	12 (0.3)
ADVERSE DRUG REACTION	7 (0.3)	BLOOD TRIGLYCERIDES INCREASED	8 (0.4)	CARDIAC DISORDER	11 (0.3)	OSTEOARTHRITIS	12 (0.3)
DISCOMFORT	7 (0.3)	LARGE INTESTINE PERFORATION	8 (0.4)	NIGHT SWEATS	11 (0.3)	GASTROOESOPHAGEAL REFLUX DISEASE	12 (0.3)
SWELLING	7 (0.3)	PULMONARY OEDEMA	8 (0.4)	APPENDICITIS	11 (0.3)	RED BLOOD CELL COUNT DECREASED	12 (0.3)
NEOPLASM MALIGNANT	7 (0.3)	LOSS OF PERSONAL INDEPENDENCE IN DAILY ACTIVITIES	8 (0.4)	FOLLICULITIS	11 (0.3)	GASTROENTERITIS VIRAL	12 (0.3)
PNEUMOCYSTIS JIROVECII PNEUMONIA	7 (0.3)	BLINDNESS	8 (0.4)	BLINDNESS	11 (0.3)	SYSTEMIC LUPUS ERYTHEMATOSUS	12 (0.3)
LIVER DISORDER	7 (0.3)	BLOOD PRESSURE INCREASED	8 (0.4)	CELLULITIS	11 (0.3)	SUICIDAL IDEATION	12 (0.3)
DYSPHAGIA	6 (0.2)	TREMOR	8 (0.4)	VERTIGO	11 (0.3)	BLOOD POTASSIUM DECREASED	12 (0.3)
POSTOPERA	6 (0.2)	NIGHT	8 (0.4)	GASTROINTE	11	FEEDING	12 (0.3)

TIVE WOUND INFECTION		SWEATS		STINAL OBSTRUCTION	(0.3)	DISORDER	
SMALL INTESTINAL PERFORATION	6 (0.2)	RHINORRHOEA	8 (0.4)	STOMA CLOSURE	10 (0.2)	CYTOMEGALOVIRUS COLITIS	12 (0.3)
HAEMOGLOBIN ABNORMAL	6 (0.2)	BRONCHITIS	8 (0.4)	MUSCULOSKELETAL DISORDER	10 (0.2)	BLOOD TRIGLYCERIDES INCREASED	12 (0.3)
LETHARGY	6 (0.2)	MEDICAL DEVICE IMPLANTATION	8 (0.4)	MALNUTRITION	10 (0.2)	DISCOMFORT	12 (0.3)
RENAL FAILURE	6 (0.2)	IMPAIRED WORK ABILITY	8 (0.4)	SKIN ULCER	10 (0.2)	PRODUCTIVE COUGH	12 (0.3)
CARDIAC FAILURE	6 (0.2)	RESPIRATORY DISORDER	8 (0.4)	RESPIRATORY TRACT INFECTION	10 (0.2)	CONFUSIONAL STATE	12 (0.3)
FULL BLOOD COUNT DECREASED	6 (0.2)	INAPPROPRIATE SCHEDULE OF PRODUCT ADMINISTRATION	8 (0.4)	BALANCE DISORDER	10 (0.2)	STOMATITIS	12 (0.3)
EMBOLISM	6 (0.2)	CARDIAC ARREST	8 (0.4)	LARGE INTESTINAL OBSTRUCTION	10 (0.2)	BACK DISORDER	12 (0.3)
STAPHYLOCOCCAL INFECTION	6 (0.2)	INTERNAL HAEMORRHAGE	8 (0.4)	INCORRECT DOSE ADMINISTERED	10 (0.2)	SARS-COV-2 TEST POSITIVE	11 (0.2)
APHTHOUS ULCER	6 (0.2)	GASTROINTESTINAL SCARRING	8 (0.4)	DIVERTICULITIS	10 (0.2)	RETINAL DETACHMENT	11 (0.2)
GASTROINTESTINAL WALL THICKENING	6 (0.2)	BLOOD CREATININE INCREASED	7 (0.3)	BURNING SENSATION	10 (0.2)	TRANSFUSION	11 (0.2)

IMPAIRED WORK ABILITY	6 (0.2)	LIMB INJURY	7 (0.3)	POST PROCEDURAL INFECTION	10 (0.2)	ABSCESS	11 (0.2)
MEMORY IMPAIRMENT	6 (0.2)	POST PROCEDURAL INFECTION	7 (0.3)	BLOOD CREATINE PHOSPHOKINASE INCREASED	10 (0.2)	DRUG HYPERSENSITIVITY	11 (0.2)
BACTERIAL INFECTION	6 (0.2)	SKIN LACERATION	7 (0.3)	PRODUCT DISPENSING ERROR	10 (0.2)	ULCER	11 (0.2)
LIVER FUNCTION TEST INCREASED	6 (0.2)	BLOOD PRESSURE DECREASED	7 (0.3)	TRANSFUSION	10 (0.2)	SKIN DISCOLOURATION	11 (0.2)
TREMOR	6 (0.2)	CONFUSIONAL STATE	7 (0.3)	LYMPHOCYTE COUNT DECREASED	10 (0.2)	CYST	11 (0.2)
SINUSITIS	6 (0.2)	OXYGEN SATURATION DECREASED	7 (0.3)	APHTHOUS ULCER	10 (0.2)	THERAPY CESSATION	11 (0.2)
LARGE INTESTINE POLYP	6 (0.2)	SMALL INTESTINAL PERFORATION	7 (0.3)	MALIGNANT MELANOMA	10 (0.2)	SCAR	11 (0.2)
URTICARIA	6 (0.2)	LUNG DISORDER	7 (0.3)	DIARRHOEA HAEMORRHAGIC	10 (0.2)	BLOOD CREATININE INCREASED	11 (0.2)
IMPAIRED HEALING	6 (0.2)	PARAESTHESIA	7 (0.3)	MENTAL DISORDER	10 (0.2)	THERAPY INTERRUPTED	11 (0.2)
SLEEP DISORDER	6 (0.2)	JOINT INJURY	7 (0.3)	NOROVIRUS INFECTION	10 (0.2)	MEMORY IMPAIRMENT	11 (0.2)
MUSCULAR WEAKNESS	6 (0.2)	ARTHROPATHY	7 (0.3)	HYPOPHAGIA	10 (0.2)	EMBOLISM VENOUS	11 (0.2)
JOINT SWELLING	6 (0.2)	DISCOMFORT	7 (0.3)	CHOLELITHIASIS	10 (0.2)	CHOLECYSTECTOMY	11 (0.2)
PULMONARY THROMBOSIS	6 (0.2)	SOCIAL PROBLEM	7 (0.3)	PELVIC FRACTURE	10 (0.2)	NOROVIRUS INFECTION	11 (0.2)
CONFUSIONAL STATE	6 (0.2)	HEART RATE INCREASED	7 (0.3)	COLON OPERATION	10 (0.2)	OEDEMA PERIPHERAL	11 (0.2)

ASPARTATE AMINOTRANSFERASE INCREASED	6 (0.2)	PNEUMOTHORAX	7 (0.3)	MOUTH ULCERATION	10 (0.2)	ESCHERICHIA INFECTION	11 (0.2)
RED BLOOD CELL COUNT DECREASED	6 (0.2)	MOBILITY DECREASED	7 (0.3)	MOBILITY DECREASED	9 (0.2)	MOUTH ULCERATION	11 (0.2)
SEPTIC SHOCK	6 (0.2)	INTERVERTEBRAL DISC PROTRUSION	7 (0.3)	STAPHYLOCOCCAL INFECTION	9 (0.2)	ABNORMAL FAECES	11 (0.2)
SPEECH DISORDER	6 (0.2)	SUICIDAL IDEATION	7 (0.3)	DYSPHAGIA	9 (0.2)	LOCALISED INFECTION	11 (0.2)
ILEUS	6 (0.2)	IMPAIRED HEALING	7 (0.3)	URTICARIA	9 (0.2)	POST PROCEDURAL COMPLICATION	11 (0.2)
UVEITIS	6 (0.2)	RASH MACULAR	7 (0.3)	KNEE ARTHROPLASTY	9 (0.2)	MEDICAL DEVICE IMPLANTATION	11 (0.2)
HYPOKALAEMIA	6 (0.2)	BLOOD CALCIUM DECREASED	7 (0.3)	ABORTION INDUCED	9 (0.2)	GASTRIC INFECTION	10 (0.2)
PSORIASIS	6 (0.2)	WRONG TECHNIQUE IN PRODUCT USAGE PROCESS	7 (0.3)	HIP SURGERY	9 (0.2)	RHEUMATOID ARTHRITIS	10 (0.2)
SMALL INTESTINAL STENOSIS	6 (0.2)	DEPRESSION	7 (0.3)	HEART RATE INCREASED	9 (0.2)	LARGE INTESTINE INFECTION	10 (0.2)
INCORRECT DOSE ADMINISTERED	6 (0.2)	ARTERIAL OCCLUSIVE DISEASE	7 (0.3)	GENERAL PHYSICAL HEALTH DETERIORATION	9 (0.2)	ANAL ABSCESS	10 (0.2)
SKIN DISCOLOURATION	6 (0.2)	POLYP	7 (0.3)	SYSTEMIC LUPUS ERYTHEMATOSUS	9 (0.2)	STAPHYLOCOCCAL INFECTION	10 (0.2)
TOOTHACH	5 (0.2)	STAPHYLOC	7 (0.3)	PRODUCT	9	UPPER LIMB	10 (0.2)

E		OCCAL INFECTION		USE ISSUE	(0.2)	FRACTURE	
HOT FLUSH	5 (0.2)	POST PROCEDURAL COMPLICATION	7 (0.3)	BLOOD TRIGLYCERIDES INCREASED	9 (0.2)	KIDNEY INFECTION	10 (0.2)
INFLAMMATORY BOWEL DISEASE	5 (0.2)	SPINAL FUSION SURGERY	6 (0.3)	CARDIAC FAILURE	9 (0.2)	EPISTAXIS	10 (0.2)
RENAL DISORDER	5 (0.2)	ABDOMINAL PAIN LOWER	6 (0.3)	GASTRITIS	9 (0.2)	SHOULDER OPERATION	10 (0.2)
LOCALISED INFECTION	5 (0.2)	NERVE COMPRESSION	6 (0.3)	POUCHITIS	9 (0.2)	LABORATORY TEST ABNORMAL	10 (0.2)
GASTROOESOPHAGEAL REFLUX DISEASE	5 (0.2)	RETINAL TEAR	6 (0.3)	ESCHERICHIA INFECTION	9 (0.2)	DYSURIA	10 (0.2)
CYST	5 (0.2)	HEPATIC STEATOSIS	6 (0.3)	BRONCHITIS	9 (0.2)	MUSCULOSKELETAL DISORDER	10 (0.2)
THERAPEUTIC RESPONSE UNEXPECTED	5 (0.2)	BLOOD URINE PRESENT	6 (0.3)	PSORIASIS	9 (0.2)	GALLBLADDER DISORDER	10 (0.2)
SYSTEMIC LUPUS ERYTHEMATOSUS	5 (0.2)	FUNGAL INFECTION	6 (0.3)	RETINAL DETACHMENT	9 (0.2)	SWELLING	10 (0.2)
RASH MACULAR	5 (0.2)	HAEMATOCRIT DECREASED	6 (0.3)	STOMATITIS	9 (0.2)	GENERAL PHYSICAL HEALTH DETERIORATION	10 (0.2)
PARAESTHESIA ORAL	5 (0.2)	ABSCESS	6 (0.3)	FURUNCLE	9 (0.2)	URTICARIA	10 (0.2)
RESPIRATORY FAILURE	5 (0.2)	EPISTAXIS	6 (0.3)	HYPERSENSITIVITY	9 (0.2)	TREMOR	10 (0.2)
SARS-COV-2 TEST POSITIVE	5 (0.2)	TENDON RUPTURE	6 (0.3)	LOCALISED INFECTION	9 (0.2)	BREAST CANCER	10 (0.2)
FLUID	5 (0.2)	INTESTINAL	6 (0.3)	SMALL	8	FUNGAL	10 (0.2)

RETENTION		OPERATION		INTESTINAL STENOSIS	(0.2)	INFECTION	
MENSTRUATION IRREGULAR	5 (0.2)	GASTROINTESTINAL INFECTION	6 (0.3)	ASTHMA	8 (0.2)	LIMB INJURY	10 (0.2)
LEUKOPENIA	5 (0.2)	ANGINA PECTORIS	6 (0.3)	GASTROINTESTINAL STOMA COMPLICATION	8 (0.2)	INFLUENZA LIKE ILLNESS	10 (0.2)
PROCTITIS	5 (0.2)	UPPER RESPIRATORY TRACT INFECTION	6 (0.3)	LOSS OF PERSONAL INDEPENDENCE IN DAILY ACTIVITIES	8 (0.2)	DIABETES MELLITUS	10 (0.2)
CHROMATURIA	5 (0.2)	BRAIN FOG	6 (0.3)	INTERVERTEBRAL DISC PROTRUSION	8 (0.2)	INCORRECT DOSE ADMINISTERED	10 (0.2)
ANORECTAL DISORDER	5 (0.2)	SYSTEMIC LUPUS ERYTHEMATOSUS	6 (0.3)	SHOULDER OPERATION	8 (0.2)	ALANINE AMINOTRANSFERASE INCREASED	10 (0.2)
GALLBLADDER DISORDER	5 (0.2)	FOOT OPERATION	6 (0.3)	COLONIC ABSCESS	8 (0.2)	LEUKOPENIA	10 (0.2)
FUNGAL INFECTION	5 (0.2)	APPENDICECTOMY	6 (0.3)	ABNORMAL FAECES	8 (0.2)	HOT FLUSH	10 (0.2)
ACUTE KIDNEY INJURY	5 (0.2)	RASH PRURITIC	6 (0.3)	NEUROPATHY PERIPHERAL	8 (0.2)	COLON OPERATION	9 (0.2)
WRONG TECHNIQUE IN PRODUCT USAGE PROCESS	5 (0.2)	DIARRHOEA HAEMORRHAGIC	6 (0.3)	ANAL INCONTINENCE	8 (0.2)	AMENORRHOEA	9 (0.2)
MOVEMENT DISORDER	5 (0.2)	LABORATORY TEST ABNORMAL	6 (0.3)	LIPIDS INCREASED	8 (0.2)	ROSACEA	9 (0.2)
INTENTIONAL DOSE OMISSION	5 (0.2)	SHOULDER FRACTURE	6 (0.3)	BLOOD MAGNESIUM DECREASED	8 (0.2)	APPENDICITIS PERFORATED	9 (0.2)

IMMUNE SYSTEM DISORDER	5 (0.2)	BURNING SENSATION	6 (0.3)	HOT FLUSH	8 (0.2)	ANAL FISSURE	9 (0.2)
PANCYTOPE NIA	5 (0.2)	PRODUCTIV E COUGH	6 (0.3)	GASTROINTE STINAL PERFORATIO N	8 (0.2)	PANCYTOPE NIA	9 (0.2)
EPISTAXIS	5 (0.2)	ABNORMAL FAECES	6 (0.3)	CYSTITIS	8 (0.2)	OPHTHALMI C HERPES ZOSTER	9 (0.2)
SMALL INTESTINAL HAEMORRH AGE	5 (0.2)	RENAL IMPAIRMEN T	6 (0.3)	ECZEMA	8 (0.2)	LUNG DISORDER	9 (0.2)
MALNUTRITI ON	5 (0.2)	URINARY RETENTION	6 (0.3)	ROSACEA	8 (0.2)	DYSCHEZIA	9 (0.2)
LIVE BIRTH	5 (0.2)	INTESTINAL STENOSIS	6 (0.3)	GASTROINTE STINAL WALL THICKENING	8 (0.2)	LOSS OF PERSONAL INDEPENDE NCE IN DAILY ACTIVITIES	9 (0.2)
PRODUCT USE ISSUE	5 (0.2)	SKIN LESION	6 (0.3)	PLATELET COUNT DECREASED	8 (0.2)	ABDOMINAL PAIN LOWER	9 (0.2)
BASAL CELL CARCINOM A	5 (0.2)	ACUTE MYOCARDIA L INFARCTION	6 (0.3)	HEPATIC STEATOSIS	8 (0.2)	IMMUNE SYSTEM DISORDER	9 (0.2)
LARGE INTESTINE INFECTION	5 (0.2)	AMNESIA	6 (0.3)	PNEUMOTH ORAX	8 (0.2)	HYPOACUSI S	9 (0.2)
THERAPY CHANGE	5 (0.2)	HERPES VIRUS INFECTION	6 (0.3)	FAECAL CALPROTEC TIN ABNORMAL	8 (0.2)	CYSTITIS	9 (0.2)
CHOLANGITI S SCLEROSIN G	5 (0.2)	SARS-COV-2 TEST POSITIVE	6 (0.3)	LARGE INTESTINAL ULCER	8 (0.2)	HAEMOGLO BIN ABNORMAL	9 (0.2)
IMMUNOSU PPRESSION	5 (0.2)	TACHYCARD IA	6 (0.3)	EAR INFECTION	8 (0.2)	ARTHROPAT HY	9 (0.2)
PROCTOCO LECTOMY	5 (0.2)	GASTRIC CANCER	6 (0.3)	ALANINE AMINOTRAN	8 (0.2)	VERTIGO	9 (0.2)

				SFERASE INCREASED			
NASAL CONGESTION	5 (0.2)	POUCHITIS	6 (0.3)	RASH PRURITIC	8 (0.2)	PHARYNGITIS STREPTOCOCCAL	9 (0.2)
DISORIENTATION	5 (0.2)	NOROVIRUS INFECTION	6 (0.3)	PAIN OF SKIN	8 (0.2)	PROCTITIS	9 (0.2)
CONCUSSION	5 (0.2)	TRANSFUSION	6 (0.3)	CYTOMEGALOVIRUS INFECTION	8 (0.2)	SKIN LACERATION	9 (0.2)
LYMPHOCYTE COUNT DECREASED	5 (0.2)	SCIATICA	6 (0.3)	TACHYCARDIA	8 (0.2)	JOINT INJURY	8 (0.2)
IMPAIRED QUALITY OF LIFE	5 (0.2)	SMALL INTESTINAL RESECTION	6 (0.3)	ULCER	8 (0.2)	SOMNOLENCE	8 (0.2)
MOUTH ULCERATION	5 (0.2)	EXOSTOSIS	6 (0.3)	PULMONARY OEDEMA	8 (0.2)	BLOOD PRESSURE DECREASED	8 (0.2)
PLEURAL EFFUSION	5 (0.2)	BREAST CANCER FEMALE	6 (0.3)	IMMUNODEFICIENCY	8 (0.2)	MEDICAL PROCEDURE	8 (0.2)
SWOLLEN TONGUE	5 (0.2)	SKIN ULCER	6 (0.3)	MUSCULOSKELETAL STIFFNESS	7 (0.2)	FAECES DISCOLOURED	8 (0.2)
FEELING COLD	5 (0.2)	INTESTINAL HAEMORRHAGE	5 (0.2)	DEAFNESS	7 (0.2)	PLATELET COUNT DECREASED	8 (0.2)
ASTHMA	5 (0.2)	INTESTINAL ANASTOMOSIS	5 (0.2)	SEPTIC SHOCK	7 (0.2)	BRAIN FOG	8 (0.2)
DRUG-INDUCED LIVER INJURY	5 (0.2)	FEELING HOT	5 (0.2)	SECRETION DISCHARGE	7 (0.2)	RESPIRATORY SYNCYTIAL VIRUS INFECTION	8 (0.2)
BLOOD BILIRUBIN INCREASED	4 (0.2)	ENTERITIS	5 (0.2)	EPISTAXIS	7 (0.2)	PRODUCT DISPENSING ERROR	8 (0.2)
GENERAL PHYSICAL HEALTH DETERIORA	4 (0.2)	DYSPHONIA	5 (0.2)	ILEAL ULCER	7 (0.2)	FEELING COLD	8 (0.2)

TION							
LOWER RESPIRATORY TRACT INFECTION	4 (0.2)	CARDIAC FAILURE	5 (0.2)	VITREOUS FLOATERS	7 (0.2)	PNEUMOTHORAX	8 (0.2)
PRODUCTIVE COUGH	4 (0.2)	DYSURIA	5 (0.2)	ABSCESS LIMB	7 (0.2)	LIGAMENT RUPTURE	8 (0.2)
GASTRIC INFECTION	4 (0.2)	LIVER FUNCTION TEST INCREASED	5 (0.2)	CHROMATURIA	7 (0.2)	BLOOD TEST ABNORMAL	8 (0.2)
LOWER GASTROINTESTINAL HAEMORRHAGE	4 (0.2)	RESPIRATORY TRACT CONGESTION	5 (0.2)	PRODUCT USE COMPLAINT	7 (0.2)	CARDIAC FAILURE CONGESTIVE	8 (0.2)
RHABDOMYOLYSIS	4 (0.2)	PANCREATITIS	5 (0.2)	RASH MACULAR	7 (0.2)	ANAL INCONTINENCE	8 (0.2)
ATRIAL FIBRILLATION	4 (0.2)	EYE PAIN	5 (0.2)	CHOKING	7 (0.2)	PLATELET COUNT INCREASED	8 (0.2)
PERITONITIS	4 (0.2)	SPINAL STENOSIS	5 (0.2)	RESPIRATORY FAILURE	7 (0.2)	IMMUNOSUPPRESSION	8 (0.2)
LOSS OF PERSONAL INDEPENDENCE IN DAILY ACTIVITIES	4 (0.2)	FEELING COLD	5 (0.2)	DYSPHONIA	7 (0.2)	HYPERSOMNIA	8 (0.2)
CLOSTRIDIUM TEST POSITIVE	4 (0.2)	LYMPHADENOPATHY	5 (0.2)	CONFUSIONAL STATE	7 (0.2)	BACTERIAL INFECTION	8 (0.2)
VITREOUS FLOATERS	4 (0.2)	RESPIRATORY TRACT INFECTION	5 (0.2)	INFLUENZA LIKE ILLNESS	7 (0.2)	EAR INFECTION	8 (0.2)
UNRESPONSIVE TO STIMULI	4 (0.2)	APHTHOUS ULCER	5 (0.2)	KIDNEY INFECTION	7 (0.2)	DYSPHONIA	8 (0.2)
PANIC ATTACK	4 (0.2)	PAIN OF SKIN	5 (0.2)	DERMATITIS ACNEIFORM	7 (0.2)	RECTAL CANCER	8 (0.2)
VAGINAL ABSCESS	4 (0.2)	RHEUMATOID ARTHRITIS	5 (0.2)	OEDEMA PERIPHERAL	7 (0.2)	ORAL CANDIDIASIS	8 (0.2)

RHINORRHOEA	4 (0.2)	LEUKOPENIA	5 (0.2)	DISORIENTATION	7 (0.2)	ASTHMA	8 (0.2)
AUTOIMMUNE DISORDER	4 (0.2)	EYE DISORDER	5 (0.2)	HIP ARTHROPLASTY	7 (0.2)	POUCHITIS	8 (0.2)
GASTROINTESTINAL OBSTRUCTION	4 (0.2)	RENAL FUNCTION TEST ABNORMAL	5 (0.2)	INTERSTITIAL LUNG DISEASE	7 (0.2)	THERAPY CHANGE	8 (0.2)
ABNORMAL FAECES	4 (0.2)	DEAFNESS	5 (0.2)	STOMA SITE PAIN	7 (0.2)	LETHARGY	8 (0.2)
RASH PAPULAR	4 (0.2)	RESPIRATORY SYNCYTIAL VIRUS INFECTION	5 (0.2)	LARGE INTESTINAL HAEMORRHAGE	7 (0.2)	INTERNAL HAEMORRHAGE	8 (0.2)
COMPLETED SUICIDE	4 (0.2)	KIDNEY INFECTION	5 (0.2)	BACTERIAL INFECTION	7 (0.2)	INAPPROPRIATE SCHEDULE OF PRODUCT ADMINISTRATION	8 (0.2)
FAECAL CALPROTECTIN ABNORMAL	4 (0.2)	ACCIDENT	5 (0.2)	DEVICE DISLOCATION	7 (0.2)	EYE SWELLING	8 (0.2)
NERVOUSNESS	4 (0.2)	BLOOD SODIUM DECREASED	5 (0.2)	ILEUS	7 (0.2)	ECZEMA	8 (0.2)
THERAPEUTIC PRODUCT EFFECT VARIABLE	4 (0.2)	WHITE BLOOD CELL COUNT ABNORMAL	5 (0.2)	HIP FRACTURE	7 (0.2)	HEAD INJURY	7 (0.2)
BLOOD PRESSURE INCREASED	4 (0.2)	COLON OPERATION	5 (0.2)	DRUG INTOLERANCE	7 (0.2)	TENDON RUPTURE	7 (0.2)
RESPIRATORY DISORDER	4 (0.2)	RECTAL CANCER	5 (0.2)	PERFORATION	7 (0.2)	PSORIASIS	7 (0.2)
SKIN WARM	4 (0.2)	LOWER LIMB FRACTURE	5 (0.2)	DEPRESSED MOOD	7 (0.2)	PULMONARY OEDEMA	7 (0.2)

POST PROCEDURAL INFECTION	4 (0.2)	LARGE INTESTINE INFECTION	5 (0.2)	IMPAIRED QUALITY OF LIFE	7 (0.2)	HERPES SIMPLEX	7 (0.2)
GASTROINTESTINAL PERFORATION	4 (0.2)	CYTOMEGALOVIRUS ENTEROCOLITIS	5 (0.2)	STOMA SITE HAEMORRHAGE	7 (0.2)	INTESTINAL RESECTION	7 (0.2)
VENOUS THROMBOSIS	4 (0.2)	SQUAMOUS CELL CARCINOMA	5 (0.2)	TOOTH EXTRACTION	7 (0.2)	ACUTE KIDNEY INJURY	7 (0.2)
SCHIZOPHRENIA	4 (0.2)	BLOOD GLUCOSE INCREASED	5 (0.2)	IMPAIRED HEALING	7 (0.2)	DRUG INTOLERANCE	7 (0.2)
BLOOD TEST ABNORMAL	4 (0.2)	BACTERIAL INFECTION	5 (0.2)	WHITE BLOOD CELL COUNT ABNORMAL	7 (0.2)	FLUID INTAKE REDUCED	7 (0.2)
TRANSIENT ISCHAEMIC ATTACK	4 (0.2)	STENT PLACEMENT	5 (0.2)	CHRONIC KIDNEY DISEASE	7 (0.2)	CHOLELITHIASIS	7 (0.2)
PANIC REACTION	4 (0.2)	FRACTURE	5 (0.2)	INAPPROPRIATE SCHEDULE OF PRODUCT ADMINISTRATION	7 (0.2)	HERPES VIRUS INFECTION	7 (0.2)
HEPATIC ENZYME INCREASED	4 (0.2)	DRY SKIN	5 (0.2)	LYMPHADENOPATHY	7 (0.2)	MASS	7 (0.2)
GASTROINTESTINAL SCARRING	4 (0.2)	MIGRAINE	5 (0.2)	THERAPEUTIC RESPONSE UNEXPECTED	7 (0.2)	GENERAL SYMPTOM	7 (0.2)
RASH PRURITIC	4 (0.2)	GASTROINTESTINAL WALL THICKENING	5 (0.2)	SARS-COV-2 TEST POSITIVE	7 (0.2)	DYSSTASIA	7 (0.2)
KNEE OPERATION	4 (0.2)	MALNUTRITION	5 (0.2)	ORAL CANDIDIASIS	7 (0.2)	PLEURAL EFFUSION	7 (0.2)
BREAST CANCER FEMALE	4 (0.2)	HAEMOGLOBIN ABNORMAL	5 (0.2)	RENAL IMPAIRMENT	7 (0.2)	JOINT STIFFNESS	7 (0.2)

NIGHT SWEATS	4 (0.2)	CARDIAC OPERATION	5 (0.2)	RHEUMATOID ARTHRITIS	7 (0.2)	FEMUR FRACTURE	7 (0.2)
POUCHITIS	4 (0.2)	BREAST CANCER	5 (0.2)	LIVER INJURY	7 (0.2)	DERMATITIS ACNEIFORM	7 (0.2)
CATARACT	4 (0.2)	GASTROENTERITIS	5 (0.2)	BONE PAIN	7 (0.2)	MENTAL DISORDER	7 (0.2)
ACCIDENT	4 (0.2)	HOT FLUSH	5 (0.2)	FEELING HOT	7 (0.2)	ABORTION INDUCED	7 (0.2)
STOMA SITE HAEMORRHAGE	4 (0.2)	TOOTH FRACTURE	5 (0.2)	LARGE INTESTINE POLYP	7 (0.2)	NEURALGIA	7 (0.2)
BALANCE DISORDER	4 (0.2)	FLUID RETENTION	5 (0.2)	DIABETES MELLITUS	7 (0.2)	POST PROCEDURAL HAEMORRHAGE	7 (0.2)
ILEAL PERFORATION	4 (0.2)	EMBOLISM VENOUS	5 (0.2)	UPPER LIMB FRACTURE	7 (0.2)	FOOT OPERATION	7 (0.2)
THERAPY CESSATION	4 (0.2)	SKIN EXFOLIATION	5 (0.2)	RESPIRATORY TRACT CONGESTION	6 (0.1)	APHONIA	7 (0.2)
PULMONARY OEDEMA	4 (0.2)	OSTEOPOROSIS	5 (0.2)	PYODERMA GANGRENOSUM	6 (0.1)	DRY SKIN	7 (0.2)
TINNITUS	4 (0.2)	PELVIC FRACTURE	5 (0.2)	NERVE COMPRESSION	6 (0.1)	CARDIAC FAILURE	7 (0.2)
BONE PAIN	4 (0.2)	ANAL FISSURE	5 (0.2)	SWOLLEN TONGUE	6 (0.1)	PHARYNGEAL SWELLING	7 (0.2)
RESPIRATORY TRACT CONGESTION	4 (0.2)	SINUS DISORDER	4 (0.2)	MENSTRUATION IRREGULAR	6 (0.1)	BLOOD CHOLESTEROL ABNORMAL	7 (0.2)
PARALYSIS	4 (0.2)	DRUG INTOLERANCE	4 (0.2)	PROCTECTOMY	6 (0.1)	GASTROINTESTINAL INFECTION	7 (0.2)
PERIRECTAL ABSCESS	4 (0.2)	PRODUCT USE ISSUE	4 (0.2)	LEUKOPENIA	6 (0.1)	BLOOD URINE PRESENT	7 (0.2)
WHITE BLOOD CELL	4 (0.2)	FISTULA DISCHARGE	4 (0.2)	HAEMATOCRIT DECREASED	6 (0.1)	SINUS CONGESTION	7 (0.2)

COUNT ABNORMAL							
FAECES SOFT	4 (0.2)	APHONIA	4 (0.2)	GASTROINTESTINAL STOMA OUTPUT INCREASED	6 (0.1)	LIVER TRANSPLANT	7 (0.2)
GASTRITIS	4 (0.2)	ANAL FISTULA	4 (0.2)	PRODUCT QUALITY ISSUE	6 (0.1)	CARDIAC FLUTTER	7 (0.2)
IMPAIRED GASTRIC EMPTYING	4 (0.2)	GASTROINTESTINAL PERFORATION	4 (0.2)	ARTERIAL OCCLUSIVE DISEASE	6 (0.1)	ACCIDENT	7 (0.2)
NEUTROPHIL COUNT INCREASED	4 (0.2)	LACRIMATION INCREASED	4 (0.2)	ACCIDENT	6 (0.1)	ANGINA PECTORIS	7 (0.2)
FACIAL PAIN	4 (0.2)	MOUTH ULCERATION	4 (0.2)	ANKYLOSING SPONDYLITIS	6 (0.1)	MOVEMENT DISORDER	7 (0.2)
SUBCUTANEOUS ABSCESS	4 (0.2)	ECZEMA	4 (0.2)	BLOOD PRESSURE DECREASED	6 (0.1)	OSTEOPOROSIS	7 (0.2)
POST PROCEDURAL DIARRHOEA	4 (0.2)	THERAPY CHANGE	4 (0.2)	FLUID RETENTION	6 (0.1)	POLLAKIURIA	7 (0.2)
EMBOLISM VENOUS	4 (0.2)	ALANINE AMINOTRANSFERASE INCREASED	4 (0.2)	COLECTOMY TOTAL	6 (0.1)	RESPIRATORY TRACT CONGESTION	7 (0.2)
STOMATITIS	4 (0.2)	CYST	4 (0.2)	INFECTED FISTULA	6 (0.1)	GASTRIC HAEMORRHAGE	7 (0.2)
MOBILITY DECREASED	4 (0.2)	BURSITIS	4 (0.2)	LARGE INTESTINE INFECTION	6 (0.1)	PANCREATITIS ACUTE	7 (0.2)
HYPERSOMNIA	4 (0.2)	ABDOMINAL ABSCESS	4 (0.2)	BACK DISORDER	6 (0.1)	SPEECH DISORDER	7 (0.2)
GANGRENE	4 (0.2)	FOLLICULITIS	4 (0.2)	BLOOD SODIUM DECREASED	6 (0.1)	NERVE COMPRESSION	7 (0.2)
KIDNEY	4 (0.2)	LETHARGY	4 (0.2)	COLD	6	WHITE	6 (0.1)

INFECTION				SWEAT	(0.1)	BLOOD CELL COUNT ABNORMAL	
LIP SWELLING	4 (0.2)	APPENDICITIS	4 (0.2)	MUSCULAR WEAKNESS	6 (0.1)	SEASONAL ALLERGY	6 (0.1)
BLOOD CREATININE INCREASED	4 (0.2)	HORDEOLUM	4 (0.2)	AMENORRHOEA	6 (0.1)	IRON DEFICIENCY ANAEMIA	6 (0.1)
GALLBLADDER OPERATION	3 (0.1)	SOMNOLENCE	4 (0.2)	RENAL FUNCTION TEST ABNORMAL	6 (0.1)	LARGE INTESTINAL STENOSIS	6 (0.1)
SURGICAL FAILURE	3 (0.1)	ARRHYTHMIA	4 (0.2)	CHOLANGITIS SCLEROSING	6 (0.1)	LARGE INTESTINAL OBSTRUCTION	6 (0.1)
THROMBOCYTOSIS	3 (0.1)	ILEAL STENOSIS	4 (0.2)	IRON DEFICIENCY	6 (0.1)	EYE HAEMORRHAGE	6 (0.1)
PYELONEPHRITIS	3 (0.1)	PSORIASIS	4 (0.2)	PROCTOCOLECTOMY	6 (0.1)	SPINAL STENOSIS	6 (0.1)
COLD SWEAT	3 (0.1)	INJURY	4 (0.2)	FUNGAL INFECTION	6 (0.1)	IMPAIRED QUALITY OF LIFE	6 (0.1)
ANAL INCONTINENCE	3 (0.1)	INTERVERTEBRAL DISC OPERATION	4 (0.2)	BLOOD CALCIUM DECREASED	6 (0.1)	EXOSTOSIS	6 (0.1)
THROAT IRRITATION	3 (0.1)	ESCHERICHIA INFECTION	4 (0.2)	ABDOMINAL OPERATION	6 (0.1)	BACTERAEMIA	6 (0.1)
TONSILLECTOMY	3 (0.1)	COLECTOMY TOTAL	4 (0.2)	PRODUCT STORAGE ERROR	6 (0.1)	BACK INJURY	6 (0.1)
RIB FRACTURE	3 (0.1)	MENISCUS INJURY	4 (0.2)	TOOTH INFECTION	6 (0.1)	SCIATICA	6 (0.1)
STOMA SITE PAIN	3 (0.1)	DENTAL OPERATION	4 (0.2)	MEDICAL PROCEDURE	6 (0.1)	PYODERMA GANGRENOSUM	6 (0.1)
HYPOAESTHESIA ORAL	3 (0.1)	NEURALGIA	4 (0.2)	PANCYTOPENIA	6 (0.1)	GASTROENTERITIS	6 (0.1)
OVERDOSE	3 (0.1)	MELAENA	4 (0.2)	SKIN DISCOLOURATION	5 (0.1)	GASTRIC CANCER	6 (0.1)
SEBORRHOEA	3 (0.1)	LIGAMENT RUPTURE	4 (0.2)	SKIN LESION	5 (0.1)	GASTRIC PERFORATI	6 (0.1)

						ON	
RECTAL TENESMUS	3 (0.1)	HYPERSOMNIA	4 (0.2)	OSTEOARTHRITIS	5 (0.1)	ACUTE MYOCARDIAL INFARCTION	6 (0.1)
HAEMATURIA	3 (0.1)	NERVOUSNESS	4 (0.2)	EYE PAIN	5 (0.1)	EYE DISORDER	6 (0.1)
PROCEDURAL HAEMORRHAGE	3 (0.1)	WALKING AID USER	4 (0.2)	DYSCHEZIA	5 (0.1)	SECRETION DISCHARGE	6 (0.1)
RESPIRATORY SYMPTOM	3 (0.1)	CHOKING	4 (0.2)	HAEMATEMESIS	5 (0.1)	JOINT DISLOCATION	6 (0.1)
ABSCESS LIMB	3 (0.1)	THERAPEUTIC RESPONSE UNEXPECTED	4 (0.2)	CHRONIC OBSTRUCTIVE PULMONARY DISEASE	5 (0.1)	BLOOD GLUCOSE INCREASED	6 (0.1)
EAR PAIN	3 (0.1)	INFECTED FISTULA	4 (0.2)	ABDOMINAL HERNIA	5 (0.1)	BOWEL MOVEMENT IRREGULARITY	6 (0.1)
NECK PAIN	3 (0.1)	SEPTIC SHOCK	4 (0.2)	NASAL CONGESTION	5 (0.1)	NERVOUSNESS	6 (0.1)
DEPRESSED LEVEL OF CONSCIOUSNESS	3 (0.1)	PROSTATOMEGALY	4 (0.2)	OSTEOPOROSIS	5 (0.1)	CHOLANGITIS SCLEROSING	6 (0.1)
DYSURIA	3 (0.1)	ABSCESS LIMB	4 (0.2)	CARDIAC OPERATION	5 (0.1)	MENINGITIS	6 (0.1)
HEAVY MENSTRUAL BLEEDING	3 (0.1)	HYPERLIPIDAEMIA	4 (0.2)	RHABDOMYOLYSIS	5 (0.1)	EMBOLISM	6 (0.1)
POST PROCEDURAL HAEMORRHAGE	3 (0.1)	HAEMORRHOIDS	4 (0.2)	PNEUMOCYSTIS JIROVECII PNEUMONIA	5 (0.1)	PANCREATIC CARCINOMA	6 (0.1)
INTRA-UTERINE CONTRACEPTIVE	3 (0.1)	ABDOMINAL HERNIA	4 (0.2)	APPENDICITIS PERFORATED	5 (0.1)	ANKLE FRACTURE	6 (0.1)

DEVICE INSERTION							
HEPATIC FAILURE	3 (0.1)	SECRETION DISCHARGE	4 (0.2)	STOMA PROLAPSE	5 (0.1)	JOINT RANGE OF MOTION DECREASED	6 (0.1)
EPSTEIN-BARR VIRUS INFECTION	3 (0.1)	ANAL HAEMORRHAGE	4 (0.2)	WOUND DEHISCENCE	5 (0.1)	GASTROINTESTINAL PERFORATION	6 (0.1)
HAEMATEMESIS	3 (0.1)	VITAMIN D DECREASED	4 (0.2)	TOOTH FRACTURE	5 (0.1)	WALKING AID USER	6 (0.1)
ARTHRITIS	3 (0.1)	RENAL DISORDER	4 (0.2)	FISTULA DISCHARGE	5 (0.1)	PERICARDIAL EFFUSION	6 (0.1)
FLANK PAIN	3 (0.1)	BACK INJURY	4 (0.2)	RECTAL ABSCESS	5 (0.1)	URINARY RETENTION	6 (0.1)
HEART RATE ABNORMAL	3 (0.1)	LIVER INJURY	4 (0.2)	SUBILEUS	5 (0.1)	OBSTRUCTION	6 (0.1)
SKIN ULCER	3 (0.1)	ANKLE FRACTURE	4 (0.2)	HYSTERECTOMY	5 (0.1)	MENISCUS INJURY	6 (0.1)
DYSSTASIA	3 (0.1)	SINUS CONGESTION	4 (0.2)	CYST	5 (0.1)	SLEEP DISORDER	6 (0.1)
FAECALOMA	3 (0.1)	SKIN DISCOLOURATION	4 (0.2)	AUTOIMMUNE DISORDER	5 (0.1)	PHOTOPHOBIA	6 (0.1)
DYSPLASIA	3 (0.1)	SPINAL COMPRESSION FRACTURE	4 (0.2)	SEBORRHOEA	5 (0.1)	PAIN OF SKIN	6 (0.1)
ADRENAL INSUFFICIENCY	3 (0.1)	FOOT DEFORMITY	4 (0.2)	ADRENAL INSUFFICIENCY	5 (0.1)	INTENTIONAL PRODUCT MISUSE	6 (0.1)
CONJUNCTIVITIS	3 (0.1)	HYPERSENSITIVITY	4 (0.2)	ASPARTATE AMINOTRANSFERASE INCREASED	5 (0.1)	NEUTROPHIL COUNT DECREASED	6 (0.1)
INTENTIONAL PRODUCT MISUSE	3 (0.1)	TACHYPNOEA	4 (0.2)	STENT PLACEMENT	5 (0.1)	RENAL FAILURE	6 (0.1)
GAMMA-GLUTAMYLTRANSFERASE	3 (0.1)	ASPARTATE AMINOTRANSFERASE	4 (0.2)	GASTROENTERITIS VIRAL	5 (0.1)	PROCTECTOMY	6 (0.1)

INCREASED		INCREASED					
HEAD INJURY	3 (0.1)	URTICARIA	4 (0.2)	MENINGITIS	5 (0.1)	ABORTION SPONTANEOUS	6 (0.1)
BACK INJURY	3 (0.1)	INTENTIONAL PRODUCT MISUSE	4 (0.2)	COLOSTOMY CLOSURE	5 (0.1)	DENTAL OPERATION	6 (0.1)
PYODERMA GANGRENOSUM	3 (0.1)	URINARY INCONTINENCE	4 (0.2)	C-REACTIVE PROTEIN ABNORMAL	5 (0.1)	SEBORRHOEA	6 (0.1)
FLUID INTAKE REDUCED	3 (0.1)	POST PROCEDURAL HAEMORRHAGE	4 (0.2)	ANAL HAEMORRHAGE	5 (0.1)	PORTAL VEIN THROMBOSIS	6 (0.1)
DISEASE PROGRESSION	3 (0.1)	CARPAL TUNNEL SYNDROME	4 (0.2)	MELAENA	5 (0.1)	BONE DISORDER	6 (0.1)
RESPIRATORY TRACT INFECTION	3 (0.1)	PULMONARY HYPERTENSION	4 (0.2)	DISABILITY	5 (0.1)	FLUID RETENTION	6 (0.1)
JAUNDICE	3 (0.1)	WRIST FRACTURE	4 (0.2)	FLANK PAIN	5 (0.1)	LIVE BIRTH	6 (0.1)
ENTEROCOLITIS	3 (0.1)	BLOOD POTASSIUM INCREASED	4 (0.2)	UNRESPONSIVE TO STIMULI	5 (0.1)	DEAFNESS	5 (0.1)
GENITAL HERPES	3 (0.1)	SLEEP APNOEA SYNDROME	4 (0.2)	GASTRIC ULCER	5 (0.1)	LIMB OPERATION	5 (0.1)
DIVERTICULITIS	3 (0.1)	GASTRITIS	4 (0.2)	HYPOKALAEMIA	5 (0.1)	BURNING SENSATION	5 (0.1)
RECTAL ADENOCARCINOMA	3 (0.1)	INCONTINENCE	4 (0.2)	HYPONATRAEMIA	5 (0.1)	LOWER GASTROINTESTINAL HAEMORRHAGE	5 (0.1)
SHOCK	3 (0.1)	CORONAVIRUS INFECTION	4 (0.2)	POSTOPERATIVE WOUND INFECTION	5 (0.1)	BLOOD SODIUM DECREASED	5 (0.1)
BLISTER	3 (0.1)	DYSSTASIA	4 (0.2)	STOMA SITE INFECTION	5 (0.1)	RENAL DISORDER	5 (0.1)
HAEMORRHOIDAL	3 (0.1)	BLOOD UREA	4 (0.2)	RECTAL DISCHARGE	5 (0.1)	ENCEPHALITIS	5 (0.1)

HAEMORRHAGE		INCREASED					
RENAL PAIN	3 (0.1)	HAEMOPTYSIS	4 (0.2)	STOMAL HERNIA	5 (0.1)	OSTEONECROSIS	5 (0.1)
APPENDICITIS PERFORATED	3 (0.1)	WOUND HAEMORRHAGE	3 (0.1)	SKIN EXFOLIATION	5 (0.1)	PRODUCT STORAGE ERROR	5 (0.1)
LOW DENSITY LIPOPROTEIN INCREASED	3 (0.1)	RETINAL VEIN OCCLUSION	3 (0.1)	ARTHROPATHY	5 (0.1)	RESPIRATORY SYMPTOM	5 (0.1)
POOR QUALITY SLEEP	3 (0.1)	SKIN ATROPHY	3 (0.1)	WRIST FRACTURE	5 (0.1)	TONSILLECTOMY	5 (0.1)
GASTRIC HAEMORRHAGE	3 (0.1)	DENTAL IMPLANTATION	3 (0.1)	BREAST CANCER FEMALE	5 (0.1)	AUTOIMMUNE DISORDER	5 (0.1)
ACNE FULMINANS	3 (0.1)	EMPHYSEMA	3 (0.1)	SPINAL FRACTURE	5 (0.1)	PAPILLARY THYROID CANCER	5 (0.1)
PELVIC FRACTURE	3 (0.1)	DYSPNOEA EXERTIONAL	3 (0.1)	SPEECH DISORDER	5 (0.1)	SMALL INTESTINAL OBSTRUCTION	5 (0.1)
GENERALISED OEDEMA	3 (0.1)	HYPOACUSIS	3 (0.1)	ACNE CYSTIC	5 (0.1)	MELANOCYTIC NAEVUS	5 (0.1)
JOINT DISLOCATION	3 (0.1)	SPEECH DISORDER	3 (0.1)	PROSTATE CANCER	5 (0.1)	CARDIAC ARREST	5 (0.1)
ABORTION SPONTANEOUS	3 (0.1)	SKIN HAEMORRHAGE	3 (0.1)	HEPATIC CIRRHOSIS	5 (0.1)	SHOULDER ARTHROPLASTY	5 (0.1)
INAPPROPRIATE SCHEDULE OF PRODUCT ADMINISTRATION	3 (0.1)	MASS	3 (0.1)	ANORECTAL DISORDER	5 (0.1)	BODY TEMPERATURE INCREASED	5 (0.1)
C-REACTIVE PROTEIN ABNORMAL	3 (0.1)	UROSEPSIS	3 (0.1)	PERIPHERAL COLDNESS	5 (0.1)	THERAPY NON-RESPONDER	5 (0.1)

SUPRAVENTRICULAR TACHYCARDIA	3 (0.1)	GASTRIC ULCER	3 (0.1)	IMPAIRED GASTRIC EMPTYING	5 (0.1)	OEDEMA	5 (0.1)
CAMPYLOBACTER INFECTION	3 (0.1)	TOOTH INFECTION	3 (0.1)	DRY MOUTH	5 (0.1)	DRUG-INDUCED LIVER INJURY	5 (0.1)
WOUND COMPLICATION	3 (0.1)	ACUTE RESPIRATORY FAILURE	3 (0.1)	MUCOUS STOOLS	5 (0.1)	ARTHRITIS INFECTIVE	5 (0.1)
HYPONATRAEMIA	3 (0.1)	RIB FRACTURE	3 (0.1)	SOMNOLENCE	5 (0.1)	IRON DEFICIENCY	5 (0.1)
COAGULOPATHY	3 (0.1)	GLAUCOMA	3 (0.1)	VOLVULUS	5 (0.1)	RETINAL TEAR	5 (0.1)
RESPIRATORY SYNCYTIAL VIRUS INFECTION	3 (0.1)	ANXIETY	3 (0.1)	BACK INJURY	5 (0.1)	ULCER HAEMORRHAGE	5 (0.1)
OVARIAN CYST	3 (0.1)	BRAIN NEOPLASM	3 (0.1)	DYSSTASIA	5 (0.1)	SUBCUTANEOUS ABSCESS	5 (0.1)
MASS	3 (0.1)	OSTEONECROSIS	3 (0.1)	SLEEP APNOEA SYNDROME	5 (0.1)	HAEMATURIA	5 (0.1)
MELANOCYTIC NAEVUS	3 (0.1)	BONE DISORDER	3 (0.1)	FAECES SOFT	5 (0.1)	UVEITIS	5 (0.1)
WOUND DEHISCENCE	3 (0.1)	MUSCULOSKELETAL DISCOMFORT	3 (0.1)	BLOOD GLUCOSE DECREASED	5 (0.1)	POOR QUALITY SLEEP	5 (0.1)
PORTAL VEIN THROMBOSIS	3 (0.1)	CORONARY ARTERY DISEASE	3 (0.1)	BRAIN NEOPLASM	5 (0.1)	BLOOD CALCIUM DECREASED	5 (0.1)
POSTOPERATIVE ABSCESS	3 (0.1)	INVASIVE DUCTAL BREAST CARCINOMA	3 (0.1)	LIVER FUNCTION TEST INCREASED	5 (0.1)	WOUND COMPLICATION	5 (0.1)
CLOSTRIDIUM DIFFICILE COLITIS	3 (0.1)	EPSTEIN-BARR VIRUS INFECTION	3 (0.1)	PERIRECTAL ABSCESS	5 (0.1)	THERAPEUTIC PRODUCT EFFECT DELAYED	5 (0.1)
ABSCESS	3 (0.1)	BACTERAEM	3 (0.1)	REHABILITAT	5	PERICARDITI	5 (0.1)

RUPTURE		IA		ION THERAPY	(0.1)	S	
PHOTOPHOBIA	3 (0.1)	ULCER HAEMORRHAGE	3 (0.1)	RESPIRATORY DISORDER	5 (0.1)	BLISTER	5 (0.1)
URINARY RETENTION	3 (0.1)	ACNE CYSTIC	3 (0.1)	MASS	4 (0.1)	GASTROINTESTINAL WALL THICKENING	5 (0.1)
SKIN EXFOLIATION	3 (0.1)	SEASONAL ALLERGY	3 (0.1)	SHOULDER FRACTURE	4 (0.1)	TRIGGER FINGER	5 (0.1)
WISDOM TEETH REMOVAL	3 (0.1)	RHABDOMYOLYSIS	3 (0.1)	RETINAL TEAR	4 (0.1)	EPSTEIN-BARR VIRUS INFECTION	5 (0.1)
BLOOD PRESSURE DECREASED	3 (0.1)	FULL BLOOD COUNT ABNORMAL	3 (0.1)	WOUND COMPLICATION	4 (0.1)	PARALYSIS	5 (0.1)
SKIN LACERATION	3 (0.1)	ASTHMA	3 (0.1)	INJURY	4 (0.1)	CARDIOVASCULAR DISORDER	5 (0.1)
PALLOR	3 (0.1)	DRAIN PLACEMENT	3 (0.1)	SQUAMOUS CELL CARCINOMA OF SKIN	4 (0.1)	POSTOPERATIVE WOUND INFECTION	5 (0.1)
COLON DYSPLASIA	3 (0.1)	CEREBRAL THROMBOSIS	3 (0.1)	COMA	4 (0.1)	CORONAVIRUS INFECTION	5 (0.1)
NEURALGIA	3 (0.1)	STOMA SITE INFECTION	3 (0.1)	BLOOD TEST ABNORMAL	4 (0.1)	ANORECTAL DISORDER	5 (0.1)
DEPRESSED MOOD	3 (0.1)	PUSTULE	3 (0.1)	PHARYNGEAL DISORDER	4 (0.1)	COLON DYSPLASIA	5 (0.1)
TERMINAL ILEITIS	3 (0.1)	CORONARY ARTERY STENOSIS	3 (0.1)	EMBOLISM	4 (0.1)	RASH MACULAR	5 (0.1)
PAIN OF SKIN	3 (0.1)	ANKLE OPERATION	3 (0.1)	RIB FRACTURE	4 (0.1)	CARPAL TUNNEL SYNDROME	5 (0.1)
PROCEDURAL COMPLICATION	3 (0.1)	CARDIAC FLUTTER	3 (0.1)	MYOSITIS	4 (0.1)	LOWER LIMB FRACTURE	5 (0.1)
ULCER HAEMORRH	3 (0.1)	BLOOD ELECTROLY	3 (0.1)	ILEOCOLECTOMY	4 (0.1)	PRODUCT SOLUBILITY	5 (0.1)

AGE		TES DECREASED				ABNORMAL	
FIBROMYALGIA	3 (0.1)	EYE OPERATION	3 (0.1)	PURULENT DISCHARGE	4 (0.1)	HYPERLIPIDAEMIA	5 (0.1)
EMERGENCY CARE	3 (0.1)	SPINAL OSTEOARTHRITIS	3 (0.1)	EAR PAIN	4 (0.1)	RENAL IMPAIRMENT	5 (0.1)
CEREBRAL INFARCTION	3 (0.1)	FACE INJURY	3 (0.1)	IRRITABILITY	4 (0.1)	CYTOMEGALOVIRUS ENTEROCOLITIS	5 (0.1)
GASTROINTESTINAL TRACT MUCOSAL DISCOLOURATION	3 (0.1)	COLD SWEAT	3 (0.1)	BLISTER	4 (0.1)	TRANSAMINASES INCREASED	5 (0.1)
HUNGER	3 (0.1)	MOVEMENT DISORDER	3 (0.1)	ILEECTOMY	4 (0.1)	SKIN MASS	5 (0.1)
PANCREATITIS ACUTE	3 (0.1)	PANCREATIC CARCINOMA	3 (0.1)	NERVOUSNESS	4 (0.1)	THROMBOCYTOSIS	5 (0.1)
FOREIGN BODY IN GASTROINTESTINAL TRACT	3 (0.1)	SEBORRHOEA	3 (0.1)	RENAL PAIN	4 (0.1)	SKIN EXFOLIATION	5 (0.1)
CHOLANGIOCARCINOMA	3 (0.1)	FLUSHING	3 (0.1)	PROCTITIS	4 (0.1)	MALNUTRITION	5 (0.1)
ILEECTOMY	3 (0.1)	BLOOD PRESSURE ABNORMAL	3 (0.1)	LACRIMATION INCREASED	4 (0.1)	BLOOD BILIRUBIN INCREASED	5 (0.1)
ILEAL ULCER	3 (0.1)	ANORECTAL DISCOMFORT	3 (0.1)	TINNITUS	4 (0.1)	BURSITIS	5 (0.1)
OPHTHALMIC HERPES ZOSTER	3 (0.1)	ILEAL ULCER	3 (0.1)	ILEAL PERFORATION	4 (0.1)	VENOUS THROMBOSIS LIMB	5 (0.1)
SKIN MASS	3 (0.1)	CONCUSSION	3 (0.1)	PANIC ATTACK	4 (0.1)	SINUS DISORDER	5 (0.1)
GASTROINTESTINAL STOMA	3 (0.1)	CEREBRAL HAEMORRHAGE	3 (0.1)	GASTROINTESTINAL SURGERY	4 (0.1)	CHROMATURIA	5 (0.1)

COMPLICATION							
DEVICE DISLOCATION	3 (0.1)	BLOOD GLUCOSE DECREASED	3 (0.1)	CONCUSSION	4 (0.1)	LIP SWELLING	5 (0.1)
BLINDNESS	3 (0.1)	URINE OUTPUT DECREASED	3 (0.1)	DRUG RESISTANCE	4 (0.1)	PROSTATOMEGALY	4 (0.1)
ABDOMINAL TENDERNESS	3 (0.1)	JOINT DISLOCATION	3 (0.1)	STOMA SITE ABSCESS	4 (0.1)	SPINAL COMPRESSION FRACTURE	4 (0.1)
EAR INFECTION	3 (0.1)	EYE HAEMORRHAGE	3 (0.1)	SALMONELLOSIS	4 (0.1)	SKIN WARM	4 (0.1)
PRESYNCOPE	3 (0.1)	BODY TEMPERATURE INCREASED	3 (0.1)	SHORT-BOWEL SYNDROME	4 (0.1)	GASTROINTESTINAL SURGERY	4 (0.1)
CHRONIC MYELOID LEUKAEMIA	3 (0.1)	REHABILITATION THERAPY	3 (0.1)	NEUTROPHIL COUNT INCREASED	4 (0.1)	GENERALISED OEDEMA	4 (0.1)
ENTERITIS INFECTIOUS	3 (0.1)	ISCHAEMIC STROKE	3 (0.1)	GASTROINTESTINAL ULCER HAEMORRHAGE	4 (0.1)	INTESTINAL ANASTOMOSIS	4 (0.1)
SUBILEUS	3 (0.1)	SKIN FISSURES	3 (0.1)	GLAUCOMA	4 (0.1)	GLOMERULAR FILTRATION RATE DECREASED	4 (0.1)
ANKYLOSING SPONDYLITIS	3 (0.1)	FURUNCLE	3 (0.1)	GASTROINTESTINAL OEDEMA	4 (0.1)	SHOULDER FRACTURE	4 (0.1)
INFLUENZA LIKE ILLNESS	3 (0.1)	CHRONIC KIDNEY DISEASE	3 (0.1)	FLUSHING	4 (0.1)	SPINAL FUSION SURGERY	4 (0.1)
BURNING SENSATION	3 (0.1)	LIVER DISORDER	3 (0.1)	FOOD POISONING	4 (0.1)	LIVER INJURY	4 (0.1)
POLYP	3 (0.1)	GASTRIC INFECTION	3 (0.1)	ORAL PAIN	4 (0.1)	HEAVY MENSTRUAL BLEEDING	4 (0.1)

PERICARDITIS	3 (0.1)	CANDIDA INFECTION	3 (0.1)	HAEMATOMA	4 (0.1)	PSORIATIC ARTHROPATHY	4 (0.1)
GASTROENTERITIS	3 (0.1)	POLLAKIURIA	3 (0.1)	BLOOD GLUCOSE INCREASED	4 (0.1)	APHTHOUS ULCER	4 (0.1)
HIP SURGERY	3 (0.1)	DEPRESSED MOOD	3 (0.1)	JOINT STIFFNESS	4 (0.1)	SEPTIC SHOCK	4 (0.1)
VENOUS THROMBOSIS LIMB	3 (0.1)	HAEMOLYTIC ANAEMIA	3 (0.1)	EAR DISCOMFORT	4 (0.1)	GASTROENTERITIS ESCHERICHIA COLI	4 (0.1)
BLOOD TRIGLYCERIDES INCREASED	3 (0.1)	PRODUCT SOLUBILITY ABNORMAL	3 (0.1)	MOVEMENT DISORDER	4 (0.1)	SPINAL OSTEOARTHRITIS	4 (0.1)
ABDOMINAL INFECTION	3 (0.1)	HYPERKERATOSIS	3 (0.1)	CARDIAC ARREST	4 (0.1)	HEART RATE DECREASED	4 (0.1)
STOMA CLOSURE	3 (0.1)	TRIGGER FINGER	3 (0.1)	HAEMOPTYSIS	4 (0.1)	CARTILAGE INJURY	4 (0.1)
LIPIDS INCREASED	3 (0.1)	DEVICE DISLOCATION	3 (0.1)	GASTROINTESTINAL INJURY	4 (0.1)	GASTROINTESTINAL MOTILITY DISORDER	4 (0.1)
SECRETION DISCHARGE	3 (0.1)	CHOLELITHIASIS	3 (0.1)	PRODUCTIVE COUGH	4 (0.1)	BLOOD POTASSIUM INCREASED	4 (0.1)
URETEROLITHIASIS	3 (0.1)	IRON DEFICIENCY ANAEMIA	3 (0.1)	INTESTINAL CYST	4 (0.1)	NEUROPATHY PERIPHERAL	4 (0.1)
VERTIGO	3 (0.1)	COLOSTOMY	3 (0.1)	OSTEONECROSIS	4 (0.1)	COMPRESSION FRACTURE	4 (0.1)
INFECTION SUSCEPTIBILITY INCREASED	3 (0.1)	LIMB OPERATION	3 (0.1)	ANASTOMOTIC ULCER	4 (0.1)	OCULAR DISCOMFORT	4 (0.1)
OSTEONECROSIS	3 (0.1)	PANCREATITIS ACUTE	3 (0.1)	UMBILICAL HERNIA	4 (0.1)	FOOT DEFORMITY	4 (0.1)
IRON DEFICIENCY	3 (0.1)	RASH VESICULAR	3 (0.1)	DRAIN PLACEMENT	4 (0.1)	PARANASAL SINUS HYPERSECRETION	4 (0.1)
DRUG	3 (0.1)	DIABETES	3 (0.1)	GASTROINTE	4	RETINAL	4 (0.1)

ERUPTION		MELLITUS		STINAL ANASTOMOTIC HAEMORRHAGE	(0.1)	HAEMORRHAGE	
IDIOPATHIC INTRACRANIAL HYPERTENSION	2 (0.1)	PHARYNGEAL DISORDER	3 (0.1)	INCISION SITE HAEMORRHAGE	4 (0.1)	ORTHOSTATIC HYPOTENSION	4 (0.1)
EPILEPSY	2 (0.1)	PNEUMONIA CRYPTOCOCCAL	3 (0.1)	HAEMATURIA	4 (0.1)	EYE INFLAMMATION	4 (0.1)
MOOD ALTERED	2 (0.1)	PROCTECTOMY	3 (0.1)	IRRITABLE BOWEL SYNDROME	4 (0.1)	HAEMORRHOIDAL HAEMORRHAGE	4 (0.1)
CELLULITE	2 (0.1)	MENINGITIS ASEPTIC	3 (0.1)	EXOSTOSIS	4 (0.1)	INVESTIGATION ABNORMAL	4 (0.1)
VASCULITIS	2 (0.1)	HYPONATRAEMIA	3 (0.1)	GALLBLADDER OPERATION	4 (0.1)	COMPLETED SUICIDE	4 (0.1)
GASTROENTERITIS ESCHERICHIA COLI	2 (0.1)	EYE SWELLING	3 (0.1)	SPINAL FUSION SURGERY	4 (0.1)	EYE PAIN	4 (0.1)
PRODUCT USE COMPLAINT	2 (0.1)	ARTHRITIS INFECTIVE	3 (0.1)	ANORECTAL DISCOMFORT	4 (0.1)	CHOKING	4 (0.1)
FOOD POISONING	2 (0.1)	CHRONIC OBSTRUCTIVE PULMONARY DISEASE	3 (0.1)	PERITONITIS	4 (0.1)	THERAPEUTIC RESPONSE UNEXPECTED	4 (0.1)
ENTEROCOLITIS VIRAL	2 (0.1)	STOMA SITE OEDEMA	3 (0.1)	APPENDICECTOMY	4 (0.1)	HORDEOLUM	4 (0.1)
ABDOMINAL OPERATION	2 (0.1)	RETINAL HAEMORRHAGE	3 (0.1)	NEURALGIA	4 (0.1)	WRONG TECHNIQUE IN PRODUCT USAGE PROCESS	4 (0.1)
MUSCULOSKELETAL	2 (0.1)	ORTHOSTATIC	3 (0.1)	FOOT OPERATION	4 (0.1)	GASTRITIS	4 (0.1)

DISORDER		HYPOTENSION					
ESCHERICHIA URINARY TRACT INFECTION	2 (0.1)	FRACTURE PAIN	3 (0.1)	RECTAL CANCER	4 (0.1)	ABSCESS LIMB	4 (0.1)
ORAL INFECTION	2 (0.1)	MEDICAL PROCEDURE	3 (0.1)	VITAMIN D DECREASED	4 (0.1)	ANAPHYLACTIC REACTION	4 (0.1)
PERONEAL NERVE PALSY	2 (0.1)	IRRITABILITY	3 (0.1)	FEELING COLD	4 (0.1)	LOWER RESPIRATORY TRACT INFECTION	4 (0.1)
HYPERTENSIVE CRISIS	2 (0.1)	STENOSIS	3 (0.1)	THYROID CANCER	4 (0.1)	SKIN ATROPHY	4 (0.1)
SLEEP APNOEA SYNDROME	2 (0.1)	PANCYTOPENIA	3 (0.1)	WOUND	4 (0.1)	TOOTH EXTRACTION	4 (0.1)
HAEMATOCRIT DECREASED	2 (0.1)	PARANASAL SINUS HYPERSECRETION	3 (0.1)	GASTRIC HAEMORRHAGE	4 (0.1)	SPINAL DISORDER	4 (0.1)
DECUBITUS ULCER	2 (0.1)	JOINT STIFFNESS	3 (0.1)	PANIC REACTION	4 (0.1)	HIGH DENSITY LIPOPROTEIN INCREASED	4 (0.1)
ANASTOMOTIC COMPLICATION	2 (0.1)	FOOD POISONING	3 (0.1)	HIATUS HERNIA	4 (0.1)	GINGIVITIS	4 (0.1)
MINERAL SUPPLEMENTATION	2 (0.1)	SLUGGISHNESS	3 (0.1)	HEAVY MENSTRUAL BLEEDING	4 (0.1)	BLOOD CREATINE INCREASED	4 (0.1)
DIPLOPIA	2 (0.1)	GASTRIC HAEMORRHAGE	3 (0.1)	SINUS DISORDER	4 (0.1)	RECTAL ABSCESS	4 (0.1)
CEREBRAL THROMBOSIS	2 (0.1)	RESPIRATORY FAILURE	3 (0.1)	LIMB DISCOMFORT	4 (0.1)	INTERVERTEBRAL DISC OPERATION	4 (0.1)
EYE PRURITUS	2 (0.1)	EAR INFECTION	3 (0.1)	CEREBRAL THROMBOSIS	4 (0.1)	PANIC ATTACK	4 (0.1)
DIABETES	2 (0.1)	INGUINAL	3 (0.1)	EYE	4	RASH	4 (0.1)

MELLITUS		HERNIA		DISORDER	(0.1)	ERYTHEMATOUS	
EYE PAIN	2 (0.1)	PROSTATIC DISORDER	3 (0.1)	HIDRADENITIS	4 (0.1)	ANKYLOSING SPONDYLITIS	4 (0.1)
DRUG DEPENDENCE	2 (0.1)	HIATUS HERNIA	3 (0.1)	LUNG DISORDER	4 (0.1)	OSTEOMYELITIS	4 (0.1)
HEMIPLEGIA	2 (0.1)	CHROMATURIA	3 (0.1)	PALLOR	4 (0.1)	TREATMENT FAILURE	4 (0.1)
INTESTINAL CYST	2 (0.1)	ABDOMINAL OPERATION	3 (0.1)	BLOOD URINE PRESENT	4 (0.1)	BLOOD UREA INCREASED	4 (0.1)
MIDDLE INSOMNIA	2 (0.1)	SKIN BURNING SENSATION	3 (0.1)	HERPES VIRUS INFECTION	4 (0.1)	DYSPLASIA	4 (0.1)
FOOT OPERATION	2 (0.1)	DEVICE PHYSICAL PROPERTY ISSUE	3 (0.1)	OVERDOSE	4 (0.1)	PRODUCT USE ISSUE	4 (0.1)
THERMAL BURN	2 (0.1)	FEMUR FRACTURE	3 (0.1)	WOUND INFECTION	4 (0.1)	FAECES HARD	4 (0.1)
EYE SWELLING	2 (0.1)	PLATELET COUNT INCREASED	3 (0.1)	INTENTIONAL PRODUCT MISUSE	4 (0.1)	BLOOD MAGNESIUM DECREASED	4 (0.1)
TUBERCULOSIS	2 (0.1)	PULMONARY MASS	3 (0.1)	ORAL MUCOSAL BLISTERING	4 (0.1)	HALLUCINATION	4 (0.1)
DRAIN PLACEMENT	2 (0.1)	ERUCTATION	3 (0.1)	MUSCULOSKELETAL DISCOMFORT	4 (0.1)	GASTROINTESTINAL SCARRING	4 (0.1)
CYANOSIS	2 (0.1)	ASPIRATION	3 (0.1)	ENTERITIS INFECTIOUS	4 (0.1)	MUSCULOSKELETAL PAIN	4 (0.1)
DRY MOUTH	2 (0.1)	STOMA SITE INFLAMMATION	3 (0.1)	OEDEMA	4 (0.1)	RIB FRACTURE	4 (0.1)
HARVEY-BRADSHAW INDEX ABNORMAL	2 (0.1)	STOMA SITE DISCHARGE	3 (0.1)	TERMINAL ILEITIS	4 (0.1)	GENITAL HERPES	4 (0.1)
ENDOCARDI	2 (0.1)	METABOLIC	3 (0.1)	TONSILLECT	4	THROAT	4 (0.1)

TIS		SURGERY		OMY	(0.1)	IRRITATION	
MUSCULOSKELETAL PAIN	2 (0.1)	BLOOD PRESSURE FLUCTUATION	3 (0.1)	OVARIAN CANCER	3 (0.1)	CHRONIC OBSTRUCTIVE PULMONARY DISEASE	4 (0.1)
ASCITES	2 (0.1)	LIPIDS INCREASED	3 (0.1)	DYSPLASIA	3 (0.1)	WRIST FRACTURE	4 (0.1)
ARTHROPATHY	2 (0.1)	IRON DEFICIENCY	3 (0.1)	POST PROCEDURAL INFLAMMATION	3 (0.1)	INJURY	4 (0.1)
SPINAL DISORDER	2 (0.1)	GALLBLADDER DISORDER	3 (0.1)	MINERAL SUPPLEMENTATION	3 (0.1)	BLOOD PRESSURE ABNORMAL	4 (0.1)
MEDICAL DEVICE IMPLANTATION	2 (0.1)	DRUG EFFECT LESS THAN EXPECTED	2 (0.1)	INTENTIONAL DOSE OMISSION	3 (0.1)	IMPAIRED WORK ABILITY	4 (0.1)
WALKING AID USER	2 (0.1)	MENINGITIS	2 (0.1)	ANASTOMOTIC COMPLICATION	3 (0.1)	SHOCK HAEMORRHAGIC	4 (0.1)
PILONIDAL DISEASE	2 (0.1)	STOMA SITE PAIN	2 (0.1)	NEUTROPHIL COUNT DECREASED	3 (0.1)	CONCUSSION	4 (0.1)
CARDIAC FLUTTER	2 (0.1)	CLAVICLE FRACTURE	2 (0.1)	ISCHAEMIC STROKE	3 (0.1)	SKIN BURNING SENSATION	4 (0.1)
OMENTAL INFARCTION	2 (0.1)	PULMONARY SEPSIS	2 (0.1)	PAIN IN JAW	3 (0.1)	EMOTIONAL DISTRESS	4 (0.1)
BIPOLAR DISORDER	2 (0.1)	HEPATIC CYTOLYSIS	2 (0.1)	PAROTITIS	3 (0.1)	CLOSTRIDIUM TEST POSITIVE	4 (0.1)
PRODUCT USE IN UNAPPROVED INDICATION	2 (0.1)	EMBOLISM	2 (0.1)	HYPOAESTHESIA ORAL	3 (0.1)	ABDOMINAL ABSCESS	4 (0.1)
COLONIC FISTULA	2 (0.1)	DENTAL RESTORATION FAILURE	2 (0.1)	RHINORRHOEA	3 (0.1)	APHASIA	4 (0.1)
ILEOSTOMY	2 (0.1)	EYE	2 (0.1)	INTESTINAL	3	SKIN LESION	4 (0.1)

CLOSURE		INFLAMMATION		MASS	(0.1)		
COLONIC ABSCESS	2 (0.1)	POST PROCEDURAL INFLAMMATION	2 (0.1)	PEPTIC ULCER	3 (0.1)	ARRHYTHMIA	4 (0.1)
STOMAL HERNIA	2 (0.1)	ABDOMINAL RIGIDITY	2 (0.1)	SURGICAL FAILURE	3 (0.1)	POST PROCEDURAL INFECTION	4 (0.1)
JOINT RANGE OF MOTION DECREASED	2 (0.1)	COLONIC ABSCESS	2 (0.1)	SLEEP DISORDER	3 (0.1)	FACIAL PAIN	4 (0.1)
INVESTIGATION ABNORMAL	2 (0.1)	UMBILICAL HERNIA	2 (0.1)	PRODUCT TEMPERATURE EXCURSION ISSUE	3 (0.1)	OVARIAN CYST	4 (0.1)
CUTANEOUS VASCULITIS	2 (0.1)	RENAL PAIN	2 (0.1)	HEAD DISCOMFORT	3 (0.1)	PULMONARY HYPERTENSION	4 (0.1)
INCREASED TENDENCY TO BRUISE	2 (0.1)	MULTIPLE ALLERGIES	2 (0.1)	GASTRIC ULCER HAEMORRHAGE	3 (0.1)	INFECTION SUSCEPTIBILITY INCREASED	4 (0.1)
SQUAMOUS CELL CARCINOMA OF SKIN	2 (0.1)	ORAL SURGERY	2 (0.1)	INTESTINAL DILATATION	3 (0.1)	INTESTINAL MASS	4 (0.1)
GROIN ABSCESS	2 (0.1)	SUDDEN DEATH	2 (0.1)	NERVOUS SYSTEM DISORDER	3 (0.1)	GASTROINTESTINAL CARCINOMA	4 (0.1)
FACE INJURY	2 (0.1)	MONOCYTE COUNT INCREASED	2 (0.1)	FAECALOMA	3 (0.1)	RETINAL VEIN OCCLUSION	4 (0.1)
NECK SURGERY	2 (0.1)	VOLVULUS	2 (0.1)	ELECTROLYTE IMBALANCE	3 (0.1)	INCREASED APPETITE	4 (0.1)
RENAL FUNCTION TEST ABNORMAL	2 (0.1)	LUNG OPACITY	2 (0.1)	ELECTROCARDIOGRAM ABNORMAL	3 (0.1)	INTESTINAL OPERATION	4 (0.1)

APHONIA	2 (0.1)	HYPOKALAEMIA	2 (0.1)	GANGRENE	3 (0.1)	COLITIS ISCHAEMIC	4 (0.1)
ECCHYMOSIS	2 (0.1)	VENTRICULAR TACHYCARDIA	2 (0.1)	LOW DENSITY LIPOPROTEIN INCREASED	3 (0.1)	COLD SWEAT	4 (0.1)
DISEASE RECURRENCE	2 (0.1)	ABDOMINAL INFECTION	2 (0.1)	LOWER RESPIRATORY TRACT INFECTION	3 (0.1)	COLOSTOMY	4 (0.1)
ABDOMINAL ADHESIONS	2 (0.1)	RENAL INJURY	2 (0.1)	POSTOPERATIVE ABSCESS	3 (0.1)	DEAFNESS UNILATERAL	4 (0.1)
OESOPHAGITIS	2 (0.1)	OESOPHAGEAL CARCINOMA	2 (0.1)	VAGINAL INFECTION	3 (0.1)	HEPATIC FAILURE	4 (0.1)
PREGNANCY	2 (0.1)	SPINAL DISORDER	2 (0.1)	SMALL INTESTINE ULCER	3 (0.1)	CANDIDA INFECTION	4 (0.1)
DIZZINESS POSTURAL	2 (0.1)	DEVICE LOOSENING	2 (0.1)	THERMAL BURN	3 (0.1)	SPUTUM DISCOLOURED	4 (0.1)
PULMONARY MASS	2 (0.1)	OESOPHAGEAL DYSPLASIA	2 (0.1)	VENTRICULAR TACHYCARDIA	3 (0.1)	JAUNDICE	4 (0.1)
OXYGEN SATURATION DECREASED	2 (0.1)	URINARY BLADDER HAEMORRHAGE	2 (0.1)	CANDIDA INFECTION	3 (0.1)	MULTIPLE ORGAN DYSFUNCTION SYNDROME	4 (0.1)
PHARYNGITIS STREPTOCOCCAL	2 (0.1)	BLOOD CHOLESTEROL ABNORMAL	2 (0.1)	FACE INJURY	3 (0.1)	TOOTHACHE	4 (0.1)
GOITRE	2 (0.1)	PNEUMONIA ASPIRATION	2 (0.1)	PULMONARY HYPERTENSION	3 (0.1)	PARAESTHESIA ORAL	4 (0.1)
ANKLE OPERATION	2 (0.1)	AORTIC ANEURYSM RUPTURE	2 (0.1)	OVARIAN CYST	3 (0.1)	INTESTINAL POLYP	4 (0.1)
EYE MOVEMENT	2 (0.1)	DEAFNESS UNILATERAL	2 (0.1)	INVASIVE DUCTAL	3 (0.1)	FLUSHING	4 (0.1)

DISORDER				BREAST CARCINOMA			
PURPURA FULMINANS	2 (0.1)	DIVERTICULUM INTESTINAL	2 (0.1)	HYPERVOLAEMIA	3 (0.1)	LACRIMATION INCREASED	4 (0.1)
MYELITIS	2 (0.1)	DIVERTICULUM	2 (0.1)	SKIN INFECTION	3 (0.1)	RESPIRATORY TRACT INFECTION	4 (0.1)
PLEURITIC PAIN	2 (0.1)	ESSENTIAL HYPERTENSION	2 (0.1)	AUTOIMMUNE PANCREATITIS	3 (0.1)	SQUAMOUS CELL CARCINOMA	4 (0.1)
SUICIDE ATTEMPT	2 (0.1)	GASTROINTESTINAL INJURY	2 (0.1)	HORDEOLUM	3 (0.1)	RHINOVIRUS INFECTION	4 (0.1)
SALMONELLOSIS	2 (0.1)	HEPATITIS B	2 (0.1)	RASH VESICULAR	3 (0.1)	EPILEPSY	4 (0.1)
SKIN CANCER	2 (0.1)	CYST RUPTURE	2 (0.1)	SQUAMOUS CELL CARCINOMA	3 (0.1)	WISDOM TEETH REMOVAL	4 (0.1)
ROSACEA	2 (0.1)	WOUND SECRETION	2 (0.1)	HARVEY-BRADSHAW INDEX ABNORMAL	3 (0.1)	RHABDOMYOLYSIS	4 (0.1)
STOMA COMPLICATION	2 (0.1)	TENOPLASTY	2 (0.1)	PRODUCT LOT NUMBER ISSUE	3 (0.1)	HYPOKALAEMIA	4 (0.1)
PERITONSILLAR ABSCESS	2 (0.1)	PARANASAL SINUS DISCOMFORT	2 (0.1)	PROCEDURAL INTESTINAL PERFORATION	3 (0.1)	PRECANCEROUS CONDITION	4 (0.1)
PRODUCT QUALITY ISSUE	2 (0.1)	BODY TEMPERATURE ABNORMAL	2 (0.1)	ULCER HAEMORRHAGE	3 (0.1)	BRAIN NEOPLASM	4 (0.1)
LYMPHOPENIA	2 (0.1)	GINGIVAL ULCERATION	2 (0.1)	PANCREATIC CARCINOMA	3 (0.1)	RESPIRATORY FAILURE	4 (0.1)
AUTOIMMUNE PANCREATITIS	2 (0.1)	MIDDLE INSOMNIA	2 (0.1)	URETEROLITHIASIS	3 (0.1)	SENSATION OF FOREIGN BODY	4 (0.1)

PERFORATION	2 (0.1)	SMALL INTESTINAL STENOSIS	2 (0.1)	INFECTION SUSCEPTIBILITY INCREASED	3 (0.1)	MEDICAL DEVICE SITE PAIN	4 (0.1)
DRY EYE	2 (0.1)	WOUND DEHISCENCE	2 (0.1)	BREAST CANCER	3 (0.1)	INTERVERTEBRAL DISC DEGENERATION	4 (0.1)
RESTLESSNESS	2 (0.1)	GLOMERULAR FILTRATION RATE DECREASED	2 (0.1)	POLYP	3 (0.1)	STENT PLACEMENT	4 (0.1)
NEUROPATHY PERIPHERAL	2 (0.1)	CARBON DIOXIDE DECREASED	2 (0.1)	VENOUS THROMBOSIS	3 (0.1)	SWOLLEN TONGUE	4 (0.1)
CHOKING	2 (0.1)	BLINDNESS UNILATERAL	2 (0.1)	FLUID INTAKE REDUCED	3 (0.1)	DEMENTIA	3 (0.1)
DYSPHONIA	2 (0.1)	COLOSTOMY CLOSURE	2 (0.1)	CHOLANGIOCARCINOMA	3 (0.1)	AUDITORY DISORDER	3 (0.1)
WOUND	2 (0.1)	CORONARY ARTERY OCCLUSION	2 (0.1)	DENTAL OPERATION	3 (0.1)	CUTANEOUS VASCULITIS	3 (0.1)
JOINT INJURY	2 (0.1)	INVESTIGATION ABNORMAL	2 (0.1)	NEPHRECTOMY	3 (0.1)	CORONARY ARTERY DISEASE	3 (0.1)
TONSILLAR HAEMORRHAGE	2 (0.1)	PRODUCT QUALITY ISSUE	2 (0.1)	PUSTULE	3 (0.1)	GINGIVAL PAIN	3 (0.1)
HERPES ZOSTER MENINGITIS	2 (0.1)	LEFT ATRIAL APPENDAGE CLOSURE IMPLANT	2 (0.1)	PAPULE	3 (0.1)	INFECTED FISTULA	3 (0.1)
DEVICE RELATED INFECTION	2 (0.1)	SCOLIOSIS	2 (0.1)	FAECES HARD	3 (0.1)	PSEUDOSTROKE	3 (0.1)
MENSTRUATION DELAYED	2 (0.1)	ATYPICAL PNEUMONIA	2 (0.1)	GASTROENTERITIS	3 (0.1)	URINARY INCONTINENCE	3 (0.1)
MEDICAL DEVICE SITE PAIN	2 (0.1)	OESOPHAGEAL PAIN	2 (0.1)	STOMA SITE DISCHARGE	3 (0.1)	EMOTIONAL DISORDER	3 (0.1)

BLOOD GLUCOSE DECREASED	2 (0.1)	CORONARY ARTERY BYPASS	2 (0.1)	ANKLE OPERATION	3 (0.1)	DEVICE BREAKAGE	3 (0.1)
REHABILITATION THERAPY	2 (0.1)	BRONCHIECTASIS	2 (0.1)	OCULAR HYPERAEMIA	3 (0.1)	PELVIC FRACTURE	3 (0.1)
PHARYNGEAL SWELLING	2 (0.1)	RED CELL DISTRIBUTION WIDTH INCREASED	2 (0.1)	DERMATITIS	3 (0.1)	MASTECTOMY	3 (0.1)
MEDICAL PROCEDURE	2 (0.1)	COELIAC DISEASE	2 (0.1)	CHRONIC MYELOID LEUKAEMIA	3 (0.1)	CHOLANGITIS	3 (0.1)
COLOSTOMY CLOSURE	2 (0.1)	HAEMATOMA	2 (0.1)	BLOOD PRESSURE FLUCTUATION	3 (0.1)	INTENTIONAL DOSE OMISSION	3 (0.1)
THYROID NEOPLASM	2 (0.1)	LYMPHOCYTE COUNT DECREASED	2 (0.1)	AMNESIA	3 (0.1)	RADICULOPATHY	3 (0.1)
ANGINA PECTORIS	2 (0.1)	SYNOVIAL CYST	2 (0.1)	LARGE INTESTINE OPERATION	3 (0.1)	ADVERSE FOOD REACTION	3 (0.1)
LIVER ABSCESS	2 (0.1)	FOREIGN BODY IN THROAT	2 (0.1)	GASTROINTESTINAL ULCER	3 (0.1)	IRRITABILITY	3 (0.1)
LARGE INTESTINAL OBSTRUCTION	2 (0.1)	ROTAVIRUS INFECTION	2 (0.1)	EPILEPSY	3 (0.1)	RASH PRURITIC	3 (0.1)
EMBOLIC STROKE	2 (0.1)	PLASMA CELL MYELOMA	2 (0.1)	HEPATOTOXICITY	3 (0.1)	PLEURITIC PAIN	3 (0.1)
RETINAL VASCULAR THROMBOSIS	2 (0.1)	PANCREATIC FAILURE	2 (0.1)	RENAL CANCER	3 (0.1)	ABNORMAL DREAMS	3 (0.1)
HEAD DISCOMFORT	2 (0.1)	THROAT CLEARING	2 (0.1)	JOINT INJURY	3 (0.1)	ATYPICAL PNEUMONIA	3 (0.1)
INTERNAL HAEMORRHAGE	2 (0.1)	EAR PAIN	2 (0.1)	BLOOD PRESSURE ABNORMAL	3 (0.1)	MUSCULOSKELETAL DISCOMFORT	3 (0.1)

RASH ERYTHEMATOUS	2 (0.1)	UPPER-AIRWAY COUGH SYNDROME	2 (0.1)	CORONARY ARTERY OCCLUSION	3 (0.1)	PULMONARY TUBERCULOSIS	3 (0.1)
ORAL MUCOSAL BLISTERING	2 (0.1)	ILEOSTOMY CLOSURE	2 (0.1)	PROCEDURAL COMPLICATION	3 (0.1)	FRACTURE	3 (0.1)
ANORECTAL DISCOMFORT	2 (0.1)	EAR DISCOMFORT	2 (0.1)	RASH PUSTULAR	3 (0.1)	ILEOSTOMY CLOSURE	3 (0.1)
BODY TEMPERATURE INCREASED	2 (0.1)	DEVICE RELATED INFECTION	2 (0.1)	ORAL SURGERY	3 (0.1)	CLOSTRIDIAL INFECTION	3 (0.1)
LABORATORY TEST ABNORMAL	2 (0.1)	GASTROINTESTINAL STOMA OUTPUT INCREASED	2 (0.1)	FOOT FRACTURE	3 (0.1)	SPONDYLITIS	3 (0.1)
HEPATIC STEATOSIS	2 (0.1)	PROCTITIS	2 (0.1)	ERUCTATION	3 (0.1)	VENTRICULAR EXTRASYSTOLES	3 (0.1)
HYPOACUSIS	2 (0.1)	SKIN WEEPING	2 (0.1)	SUPRAVENTRICULAR TACHYCARDIA	3 (0.1)	INCONTINENCE	3 (0.1)
WEIGHT GAIN POOR	2 (0.1)	RASH PUSTULAR	2 (0.1)	COLON DYSPLASIA	3 (0.1)	ANORECTAL DISCOMFORT	3 (0.1)
STOMA PROLAPSE	2 (0.1)	HAEMOLYSIS	2 (0.1)	URINE OUTPUT DECREASED	3 (0.1)	FOOD ALLERGY	3 (0.1)
BLOOD SODIUM DECREASED	2 (0.1)	OPHTHALMIC HERPES ZOSTER	2 (0.1)	RHINOVIRUS INFECTION	3 (0.1)	LYMPHOMA	3 (0.1)
RED CELL DISTRIBUTION WIDTH INCREASED	2 (0.1)	INTERVERTEBRAL DISC DISORDER	2 (0.1)	BLINDNESS UNILATERAL	3 (0.1)	CHOLANGIOCARCINOMA	3 (0.1)
THERAPY NON-RESPONDER	2 (0.1)	GASTRIC PERFORATION	2 (0.1)	LUNG INFILTRATION	3 (0.1)	C-REACTIVE PROTEIN ABNORMAL	3 (0.1)

VARICELLA ZOSTER VIRUS INFECTION	2 (0.1)	ANAPHYLACTIC REACTION	2 (0.1)	PHOTOPHOBIA	3 (0.1)	BREAST PAIN	3 (0.1)
ANGIOPATHY	2 (0.1)	DYSCHEZIA	2 (0.1)	ABDOMINAL TENDERNESS	3 (0.1)	POST PROCEDURAL DIARRHOEA	3 (0.1)
GASTROINTESTINAL ULCER	2 (0.1)	GROIN PAIN	2 (0.1)	CEREBRAL HAEMORRHAGE	3 (0.1)	RETINAL DEGENERATION	3 (0.1)
APHASIA	2 (0.1)	INGUINAL MASS	2 (0.1)	CEREBRAL INFARCTION	3 (0.1)	UROSEPSIS	3 (0.1)
OCULAR HYPERAEMIA	2 (0.1)	DRUG RESISTANCE	2 (0.1)	GENITAL HERPES	3 (0.1)	BEDRIDDEN	3 (0.1)
DENTAL OPERATION	2 (0.1)	STOMA CLOSURE	2 (0.1)	COLONIC FISTULA	3 (0.1)	COGNITIVE DISORDER	3 (0.1)
SHORT-BOWEL SYNDROME	2 (0.1)	PAINFUL RESPIRATION	2 (0.1)	ANAL CANCER	3 (0.1)	HYDROCEPHALUS	3 (0.1)
HAEMOPTYSIS	2 (0.1)	MELANOCYTIC NAEVUS	2 (0.1)	JAUNDICE	3 (0.1)	MICTURITION URGENCY	3 (0.1)
ILEUS PARALYTIC	2 (0.1)	PHOTOPHOBIA	2 (0.1)	PRODUCT USE IN UNAPPROVED INDICATION	3 (0.1)	INTERMENSTRUAL BLEEDING	3 (0.1)
ANKLE FRACTURE	2 (0.1)	PORTAL VEIN THROMBOSIS	2 (0.1)	FUNGAL SKIN INFECTION	3 (0.1)	DEVICE RELATED INFECTION	3 (0.1)
MUSCULOSKELETAL STIFFNESS	2 (0.1)	GASTROINTESTINAL OEDEMA	2 (0.1)	DRUG INTERACTION	3 (0.1)	INTRA-UTERINE CONTRACEPTIVE DEVICE INSERTION	3 (0.1)
PRODUCT STORAGE ERROR	2 (0.1)	DECREASED ACTIVITY	2 (0.1)	CONJUNCTIVITIS	3 (0.1)	LARGE INTESTINAL ULCER	3 (0.1)
RECTAL CANCER	2 (0.1)	SENSITIVE SKIN	2 (0.1)	DISTURBANCE IN ATTENTION	3 (0.1)	FURUNCLE	3 (0.1)
CARDIAC	2 (0.1)	GLOSSODY	2 (0.1)	TASTE	3	MUSCLE	3 (0.1)

DISORDER		NIA		DISORDER	(0.1)	ATROPHY	
HIDRADENITIS	2 (0.1)	NEPHRECTOMY	2 (0.1)	PHARYNGEAL ULCERATION	3 (0.1)	HEPATIC ENZYME ABNORMAL	3 (0.1)
FURUNCLE	2 (0.1)	ELECTROLYTE IMBALANCE	2 (0.1)	HYPOGLYCAEMIA	3 (0.1)	HAEMORRHOIDS THROMBOSED	3 (0.1)
AUTOIMMUNE THYROIDITIS	2 (0.1)	VAGINAL INFECTION	2 (0.1)	PLEURAL EFFUSION	3 (0.1)	ABDOMINAL HERNIA	3 (0.1)
CAMPYLOBACTER GASTROENTERITIS	2 (0.1)	MUSCLE ENZYME INCREASED	2 (0.1)	NECK SURGERY	3 (0.1)	POST HERPETIC NEURALGIA	3 (0.1)
LUPUS-LIKE SYNDROME	2 (0.1)	INCREASED APPETITE	2 (0.1)	CLOSTRIDIUM TEST POSITIVE	3 (0.1)	FACE INJURY	3 (0.1)
COMPRESSION FRACTURE	2 (0.1)	GLYCOSYLATED HAEMOGLOBIN INCREASED	2 (0.1)	LIP SWELLING	3 (0.1)	SUPERINFECTION	3 (0.1)
GASTROINTESTINAL STOMA OUTPUT INCREASED	2 (0.1)	UMBILICOPLASTY	2 (0.1)	IDIOPATHIC INTRACRANIAL HYPERTENSION	3 (0.1)	HEPATITIS C	3 (0.1)
INTESTINAL MASS	2 (0.1)	VULVOVAGINAL PRURITUS	2 (0.1)	UROSEPSIS	3 (0.1)	COAGULOPATHY	3 (0.1)
MICTURITION URGENCY	2 (0.1)	FLUID INTAKE REDUCED	2 (0.1)	DIVERTICULUM	3 (0.1)	CHRONIC KIDNEY DISEASE	3 (0.1)
GENERAL SYMPTOM	2 (0.1)	PERFORATION	2 (0.1)	PNEUMONITIS	3 (0.1)	RECTAL TENESMUS	3 (0.1)
LIMB INJURY	2 (0.1)	CARDIAC FAILURE CONGESTIVE	2 (0.1)	ABSCESS RUPTURE	3 (0.1)	AMNESIA	3 (0.1)
MYOCARDITIS	2 (0.1)	BREAST MASS	2 (0.1)	ABDOMINAL INFECTION	3 (0.1)	THIRST	3 (0.1)
THYROID	2 (0.1)	CHOLANGITI	2 (0.1)	ERYTHEMA	3	SKIN	3 (0.1)

CANCER		S SCLEROSING		NODOSUM	(0.1)	FISSURES	
STRESS AT WORK	2 (0.1)	CHOLANGIOCARCINOMA	2 (0.1)	INFREQUENT BOWEL MOVEMENTS	3 (0.1)	FOOD POISONING	3 (0.1)
DELUSION	2 (0.1)	AUDITORY DISORDER	2 (0.1)	INTESTINAL FISTULA	3 (0.1)	FACE OEDEMA	3 (0.1)
EAR DISCOMFORT	2 (0.1)	HEPATOMEGALY	2 (0.1)	ANIMAL BITE	3 (0.1)	TONSILLITIS	3 (0.1)
KNEE ARTHROPLASTY	2 (0.1)	APPENDICITIS PERFORATED	2 (0.1)	POST PROCEDURAL HAEMORRHAGE	3 (0.1)	DENTAL CARE	3 (0.1)
PRODUCT SOLUBILITY ABNORMAL	2 (0.1)	PYODERMA GANGRENOSUM	2 (0.1)	RECTAL TENESMUS	3 (0.1)	AGITATION	3 (0.1)
ILEOCOLECTOMY	2 (0.1)	GALLBLADDER OPERATION	2 (0.1)	URINARY RETENTION	3 (0.1)	PRODUCT ADMINISTRATION ERROR	3 (0.1)
CHOLANGITIS	2 (0.1)	OSTEOMYELITIS	2 (0.1)	TOOTH DISORDER	3 (0.1)	GINGIVAL BLEEDING	3 (0.1)
RHINOVIRUS INFECTION	2 (0.1)	BURSITIS INFECTIVE	2 (0.1)	SUICIDE ATTEMPT	3 (0.1)	INFREQUENT BOWEL MOVEMENTS	3 (0.1)
STAPHYLOCOCCAL BACTERAEMIA	2 (0.1)	C-REACTIVE PROTEIN ABNORMAL	2 (0.1)	HERPES ZOSTER MENINGITIS	3 (0.1)	DYSPNOEA EXERTIONAL	3 (0.1)
MENINGITIS	2 (0.1)	BLISTER	2 (0.1)	PAINFUL RESPIRATION	3 (0.1)	DRUG INTERACTION	3 (0.1)
LIVER FUNCTION TEST ABNORMAL	2 (0.1)	DEVICE LEAKAGE	2 (0.1)	INCONTINENCE	3 (0.1)	LUMBAR VERTEBRAL FRACTURE	3 (0.1)
ORAL PAIN	2 (0.1)	DRY EYE	2 (0.1)	HYDRONEPHROSIS	3 (0.1)	IMPAIRED GASTRIC EMPTYING	3 (0.1)
SLEEP DEFICIT	2 (0.1)	BONE PAIN	2 (0.1)	RED CELL DISTRIBUTIO	3 (0.1)	STOOL ANALYSIS	3 (0.1)

				N WIDTH INCREASED		ABNORMAL	
RASH VESICULAR	2 (0.1)	POLYCHONDRITIS	2 (0.1)	SKIN LACERATION	3 (0.1)	ADENOCARCINOMA	3 (0.1)
MYELOSUPPRESSION	2 (0.1)	AORTIC DISSECTION	2 (0.1)	ARTHRITIS BACTERIAL	3 (0.1)	THROAT TIGHTNESS	3 (0.1)
ARTHRITIS INFECTIVE	2 (0.1)	STOMA SITE HAEMORRHAGE	2 (0.1)	PYELONEPHRITIS	3 (0.1)	DRY MOUTH	3 (0.1)
ABORTION	2 (0.1)	PALLOR	2 (0.1)	BLOOD CREATINE PHOSPHOKINASE ABNORMAL	3 (0.1)	INFECTIOUS MONONUCLEOSIS	3 (0.1)
MYOGLOBIN BLOOD INCREASED	2 (0.1)	FIBRIN D DIMER INCREASED	2 (0.1)	MULTIPLE SCLEROSIS	3 (0.1)	STOMA SITE INFLAMMATION	3 (0.1)
BLOOD LACTATE DEHYDROGENASE INCREASED	2 (0.1)	SHOCK HAEMORRHAGIC	2 (0.1)	HUNGER	3 (0.1)	WOUND HAEMORRHAGE	3 (0.1)
VARICELLA	2 (0.1)	PAPILLARY THYROID CANCER	2 (0.1)	THINKING ABNORMAL	3 (0.1)	COLORECTAL CANCER	3 (0.1)
RADICULOPATHY	2 (0.1)	TONGUE DISCOMFORT	2 (0.1)	SPINAL DISORDER	3 (0.1)	MENINGITIS ASEPTIC	3 (0.1)
CARDIOVASCULAR DISORDER	2 (0.1)	HEPATIC CANCER	2 (0.1)	ACUTE MYOCARDIAL INFARCTION	3 (0.1)	MIDDLE INSOMNIA	3 (0.1)
MAGNESIUM DEFICIENCY	2 (0.1)	LUNG NEOPLASM MALIGNANT	2 (0.1)	HEPATIC NEOPLASM	3 (0.1)	FULL BLOOD COUNT ABNORMAL	3 (0.1)
COMA	2 (0.1)	HEAVY EXPOSURE TO ULTRAVIOLET LIGHT	2 (0.1)	TOOTHACHE	3 (0.1)	PRODUCT USE COMPLAINT	3 (0.1)
COGNITIVE DISORDER	2 (0.1)	NEUTROPENIA	2 (0.1)	APHONIA	3 (0.1)	HEMIPLEGIA	3 (0.1)
STOMA SITE INFECTION	2 (0.1)	POST-ACUTE COVID-19	2 (0.1)	BLOOD ELECTROLYT	3 (0.1)	ABSCESS INTESTINAL	3 (0.1)

		SYNDROME		ES DECREASED			
TREATMENT NONCOMPLIANCE	2 (0.1)	MUSCULOSKELETAL PAIN	2 (0.1)	TENDON RUPTURE	3 (0.1)	CARDIO-RESPIRATORY ARREST	3 (0.1)
UPPER LIMB FRACTURE	2 (0.1)	LUNG INFILTRATION	2 (0.1)	DRY EYE	3 (0.1)	DRUG ERUPTION	3 (0.1)
EMOTIONAL DISORDER	2 (0.1)	SWOLLEN TONGUE	2 (0.1)	WISDOM TEETH REMOVAL	3 (0.1)	PRECANCEROUS CELLS PRESENT	3 (0.1)
GASTRIC PERFORATION	2 (0.1)	GASTROINTESTINAL OBSTRUCTION	2 (0.1)	DEVICE ISSUE	3 (0.1)	PUSTULE	3 (0.1)
CERVIX CARCINOMA	2 (0.1)	RESPIRATORY SYMPTOM	2 (0.1)	DUODENITIS	3 (0.1)	GASTRIC DISORDER	3 (0.1)
INCORRECT ROUTE OF PRODUCT ADMINISTRATION	2 (0.1)	BILIARY COLIC	2 (0.1)	EYE PRURITUS	3 (0.1)	HEART RATE IRREGULAR	3 (0.1)
TESTIS CANCER	2 (0.1)	DEMENTIA	2 (0.1)	HYPOACUSIS	3 (0.1)	URETEROLITHIASIS	3 (0.1)
MUSCULOSKELETAL DISCOMFORT	2 (0.1)	SLEEP DISORDER	2 (0.1)	ODYNOPHAGIA	3 (0.1)	SUICIDE THREAT	3 (0.1)
FLUSHING	2 (0.1)	INFREQUENT BOWEL MOVEMENTS	2 (0.1)	THERAPEUTIC PRODUCT EFFECT VARIABLE	3 (0.1)	DENTAL IMPLANTATION	3 (0.1)
DISTURBANCE IN ATTENTION	2 (0.1)	EYE DISCHARGE	2 (0.1)	LIVE BIRTH	3 (0.1)	SPINAL PAIN	3 (0.1)
FEELING HOT	2 (0.1)	HERPES ZOSTER MENINGITIS	2 (0.1)	WOUND SECRETION	3 (0.1)	PNEUMOPERITONEUM	3 (0.1)
MEGACOLON	2 (0.1)	SKIN TIGHTNESS	2 (0.1)	TEMPERATURE INTOLERANCE	3 (0.1)	EMPHYSEMA	3 (0.1)
GASTROINT	2 (0.1)	INTRAOCUL	2 (0.1)	PULMONARY	3	JOINT	3 (0.1)

ESTINAL INJURY		AR PRESSURE INCREASED		MASS	(0.1)	EFFUSION	
MUSCLE ATROPHY	2 (0.1)	LIP DISCOLOURATION	2 (0.1)	ADVERSE EVENT	3 (0.1)	SLEEP APNOEA SYNDROME	3 (0.1)
SUICIDE THREAT	2 (0.1)	TONSILLECTOMY	2 (0.1)	GASTROINTESTINAL TRACT MUCOSAL DISCOLOURATION	3 (0.1)	PHARYNGEAL OPERATION	3 (0.1)
EXPOSURE TO COMMUNICABLE DISEASE	2 (0.1)	SMALL INTESTINE ULCER	2 (0.1)	ABDOMINAL ADHESIONS	3 (0.1)	TINNITUS	3 (0.1)
INFECTIOUS MONONUCLEOSIS	2 (0.1)	EPILEPSY	2 (0.1)	MUSCLE TIGHTNESS	3 (0.1)	BLOOD ALKALINE PHOSPHATASE INCREASED	3 (0.1)
HEPATIC FUNCTION ABNORMAL	2 (0.1)	DRY MOUTH	2 (0.1)	STOMA SITE OEDEMA	3 (0.1)	RADIOTHERAPY	3 (0.1)
LYMPH NODE TUBERCULOSIS	2 (0.1)	DELIRIUM	2 (0.1)	SCIATICA	3 (0.1)	FEELING HOT	3 (0.1)
NERVOUS SYSTEM DISORDER	2 (0.1)	SCIATIC NERVE NEUROPATHY	2 (0.1)	BLOOD IRON ABNORMAL	3 (0.1)	HEPATIC CIRRHOSIS	3 (0.1)
ONYCHOMYCOSIS	2 (0.1)	INTESTINAL MASS	2 (0.1)	ADENOCARCINOMA	3 (0.1)	HEPATIC CANCER	3 (0.1)
BLOOD GLUCOSE INCREASED	2 (0.1)	OCULAR HYPERAEMIA	2 (0.1)	PANCREATITIS ACUTE	3 (0.1)	CYTOMEGALOVIRUS INFECTION REACTIVATION	3 (0.1)
PURULENT DISCHARGE	2 (0.1)	GASTRIC DILATATION	2 (0.1)	DRUG-INDUCED LIVER INJURY	3 (0.1)	HYPERKERATOSIS	3 (0.1)
DIARRHOEA	2 (0.1)	LATENT	2 (0.1)	HEART RATE	3	DIVERTICUL	3 (0.1)

INFECTIOUS		TUBERCULOSIS		ABNORMAL	(0.1)	UM	
HORMONE LEVEL ABNORMAL	2 (0.1)	STOMA SITE REACTION	2 (0.1)	BODY TEMPERATURE INCREASED	3 (0.1)	VITAMIN D DECREASED	3 (0.1)
CHOLECYSTITIS	2 (0.1)	BONE CONTUSION	2 (0.1)	HEPATIC FAILURE	3 (0.1)	GLAUCOMA	3 (0.1)
PUSTULE	2 (0.1)	LIP PAIN	2 (0.1)	ARTHRITIS ENTEROPATHIC	3 (0.1)	WOUND INFECTION	3 (0.1)
SUPERINFECTION	2 (0.1)	FAECES HARD	2 (0.1)	RENAL CYST	3 (0.1)	ARTERIAL OCCLUSIVE DISEASE	3 (0.1)
TOOTH LOSS	2 (0.1)	ARTERIAL DISORDER	2 (0.1)	BLOOD CREATININE ABNORMAL	3 (0.1)	PILONIDAL DISEASE	3 (0.1)
ELECTROCARDIOGRAM ABNORMAL	2 (0.1)	IRRITABLE BOWEL SYNDROME	2 (0.1)	PNEUMONIA ASPIRATION	3 (0.1)	CATHETERISATION CARDIAC	3 (0.1)
SKIN LESION	2 (0.1)	CHEST INJURY	2 (0.1)	ANKLE FRACTURE	3 (0.1)	MEAN CELL VOLUME INCREASED	3 (0.1)
BACK DISORDER	2 (0.1)	POOR QUALITY SLEEP	2 (0.1)	THERAPY CHANGE	3 (0.1)	RASH VESICULAR	3 (0.1)
ENCEPHALITIS	2 (0.1)	RED BLOOD CELL SEDIMENTATION RATE INCREASED	2 (0.1)	VASCULITIS	3 (0.1)	LYMPH NODE TUBERCULOSIS	3 (0.1)
IRRITABILITY	2 (0.1)	PNEUMONIA FUNGAL	2 (0.1)	RECTAL ADENOCARCINOMA	2 (0.0)	REHABILITATION THERAPY	3 (0.1)
MEDICAL DEVICE REMOVAL	2 (0.1)	CAROTID ARTERY OCCLUSION	2 (0.1)	LYMPHADENITIS	2 (0.0)	VASCULITIS	3 (0.1)
ARTHRITIS BACTERIAL	2 (0.1)	LARGE INTESTINAL ULCER	2 (0.1)	CUTANEOUS VASCULITIS	2 (0.0)	BLADDER NEOPLASM	3 (0.1)
SKIN INFECTION	2 (0.1)	ORGANISING PNEUMONIA	2 (0.1)	DRUG EFFECT LESS THAN EXPECTED	2 (0.0)	PALLOR	3 (0.1)

FIBRIN D DIMER INCREASED	2 (0.1)	FAECES SOFT	2 (0.1)	LUNG CARCINOMA CELL TYPE UNSPECIFIED STAGE IV	2 (0.0)	CORNEAL DISORDER	3 (0.1)
AXIAL SPONDYLOARTHRITIS	2 (0.1)	HYSTERECTOMY	2 (0.1)	EPSTEIN-BARR VIRUS INFECTION	2 (0.0)	HAEMATOCRIT DECREASED	3 (0.1)
DEVICE ISSUE	2 (0.1)	PHARYNGITIS	2 (0.1)	POLYPECTOMY	2 (0.0)	TENDON DISORDER	3 (0.1)
PAINFUL RESPIRATION	2 (0.1)	OVARIAN CANCER	2 (0.1)	MENSTRUAL CLOTS	2 (0.0)	OCULAR HYPERAEMIA	3 (0.1)
BLINDNESS TRANSIENT	2 (0.1)	GENERALISED OEDEMA	2 (0.1)	LOWER GASTROINTESTINAL HAEMORRHAGE	2 (0.0)	EYE OEDEMA	3 (0.1)
BILE ACID MALABSORPTION	2 (0.1)	B-CELL LYMPHOMA	2 (0.1)	APPENDICEAL ABSCESS	2 (0.0)	ENTEROCOLITIS	3 (0.1)
STOMA SITE ABSCESS	2 (0.1)	INTERVERTEBRAL DISC DEGENERATION	2 (0.1)	HEPATIC CANCER	2 (0.0)	PLEURISY	3 (0.1)
C-REACTIVE PROTEIN DECREASED	2 (0.1)	BLOOD ALBUMIN DECREASED	2 (0.1)	LABORATORY TEST ABNORMAL	2 (0.0)	DEPRESSED LEVEL OF CONSCIOUSNESS	3 (0.1)
WOUND INFECTION	2 (0.1)	SPUTUM DISCOLOURED	2 (0.1)	RADIOTHERAPY	2 (0.0)	NIGHTMARE	3 (0.1)
JOINT STIFFNESS	2 (0.1)	INTESTINAL POLYP	2 (0.1)	BLOOD ZINC DECREASED	2 (0.0)	STOMA SITE DISCHARGE	3 (0.1)
MULTIPLE ORGAN DYSFUNCTION SYNDROME	2 (0.1)	INCISION SITE PAIN	2 (0.1)	BACTERIAL SEPSIS	2 (0.0)	SUDDEN DEATH	3 (0.1)
CARDIAC ARREST	2 (0.1)	CARDIO-RESPIRATORY ARREST	2 (0.1)	EMERGENCY CARE	2 (0.0)	TOOTH FRACTURE	3 (0.1)
FEBRILE NEUTROPE	2 (0.1)	AORTIC ANEURYSM	2 (0.1)	PERITONSILLAR ABSCESS	2 (0.0)	HEPATIC MASS	3 (0.1)

NIA							
HAEMATOMA	2 (0.1)	ILEOCOLECTOMY	2 (0.1)	RETINAL VEIN OCCLUSION	2 (0.0)	PANCREATIC DISORDER	3 (0.1)
RETINAL VEIN OCCLUSION	2 (0.1)	SPONDYLITIS	2 (0.1)	LYMPHOPENIA	2 (0.0)	GALLBLADDER OPERATION	3 (0.1)
INTERMITTENT CLAUDICATION	2 (0.1)	PNEUMONITIS	2 (0.1)	STOMA OBSTRUCTION	2 (0.0)	DISABILITY	3 (0.1)
ANAL HAEMORRHAGE	2 (0.1)	SHOULDER ARTHROPLASTY	2 (0.1)	TRISMUS	2 (0.0)	PULMONARY MASS	3 (0.1)
VENA CAVA THROMBOSIS	2 (0.1)	HEPATIC CIRRHOSIS	2 (0.1)	SHOCK	2 (0.0)	EYE OPERATION	3 (0.1)
MOANING	2 (0.1)	PHARYNGITIS STREPTOCOCCAL	2 (0.1)	TACHYPNOEA	2 (0.0)	PROCEDURAL COMPLICATION	3 (0.1)
BRAIN NEOPLASM	2 (0.1)	ANKYLOSING SPONDYLITIS	2 (0.1)	RETINAL VASCULAR THROMBOSIS	2 (0.0)	HEPATIC STEATOSIS	3 (0.1)
ABDOMINAL LYMPHADENOPATHY	2 (0.1)	TREATMENT FAILURE	2 (0.1)	APPETITE DISORDER	2 (0.0)	METABOLIC SURGERY	3 (0.1)
ARTHRITIS ENTEROPATHIC	2 (0.1)	RETINAL DEGENERATION	2 (0.1)	TREATMENT NONCOMPLIANCE	2 (0.0)	VAGINAL ABSCESS	3 (0.1)
GASTROINTESTINAL FISTULA	2 (0.1)	NEUTROPHIL COUNT DECREASED	2 (0.1)	EYE INFLAMMATION	2 (0.0)	ORAL INFECTION	3 (0.1)
UPPER RESPIRATORY TRACT CONGESTION	2 (0.1)	GASTROINTESTINAL CARCINOMA	2 (0.1)	ACUTE RESPIRATORY FAILURE	2 (0.0)	MONOCYTE COUNT INCREASED	2 (0.0)
NEUTROPENIA	2 (0.1)	BLOOD BLISTER	2 (0.1)	HAEMORRHAGIC STROKE	2 (0.0)	FRACTURE PAIN	2 (0.0)
ATYPICAL PNEUMONIA	2 (0.1)	PROSTATE CANCER	2 (0.1)	VARICELLA ZOSTER	2 (0.0)	SMALL INTESTINAL	2 (0.0)

		METASTATIC		VIRUS INFECTION		RESECTION	
HERPES SIMPLEX	2 (0.1)	IMMUNE SYSTEM DISORDER	2 (0.1)	POST PROCEDURAL DIARRHOEA	2 (0.0)	HEPATITIS TOXIC	2 (0.0)
PARONYCHIA	2 (0.1)	BUNDLE BRANCH BLOCK RIGHT	2 (0.1)	FAECAL CALPROTECTIN DECREASED	2 (0.0)	TOE OPERATION	2 (0.0)
RECTAL DISCHARGE	2 (0.1)	CARDIAC VENTRICULAR THROMBOSIS	2 (0.1)	C-REACTIVE PROTEIN DECREASED	2 (0.0)	SYMPTOM RECURRENCE	2 (0.0)
NEUTROPHIL COUNT DECREASED	2 (0.1)	FACIAL DISCOMFORT	2 (0.1)	MYELOSUPPRESSION	2 (0.0)	ANGIOPATHY	2 (0.0)
MUSCLE TIGHTNESS	2 (0.1)	GASTROINTESTINAL MOTILITY DISORDER	2 (0.1)	THROAT IRRITATION	2 (0.0)	NOCARDIOSIS	2 (0.0)
PERICARDIAL EFFUSION	2 (0.1)	TUBERCULIN TEST POSITIVE	2 (0.1)	TREATMENT FAILURE	2 (0.0)	PSEUDOPOLYP	2 (0.0)
RECTAL ABSCESS	2 (0.1)	MULTIPLE ORGAN DYSFUNCTION SYNDROME	2 (0.1)	GASTROINTESTINAL FISTULA	2 (0.0)	UPPER-AIRWAY COUGH SYNDROME	2 (0.0)
FEMUR FRACTURE	2 (0.1)	DIASTOLIC DYSFUNCTION	2 (0.1)	LIP BLISTER	2 (0.0)	CONJUNCTIVITIS	2 (0.0)
MYOCARDIAL INFARCTION	2 (0.1)	THROAT CANCER	2 (0.1)	UPPER RESPIRATORY TRACT CONGESTION	2 (0.0)	RECTAL ULCER	2 (0.0)
ANAL STENOSIS	2 (0.1)	BREAST HYPERPLASIA	2 (0.1)	NERVE INJURY	2 (0.0)	RECTAL POLYP	2 (0.0)
GASTROINTESTINAL SURGERY	2 (0.1)	OEDEMA PERIPHERAL	2 (0.1)	SKIN TIGHTNESS	2 (0.0)	GOITRE	2 (0.0)

INTESTINAL FISTULA	2 (0.1)	ADRENAL INSUFFICIENCY	2 (0.1)	ABDOMINAL LYMPHADENOPATHY	2 (0.0)	SPONTANEOUS HAEMORRHAGE	2 (0.0)
LARGE INTESTINE OPERATION	2 (0.1)	ANIMAL BITE	2 (0.1)	VASCULAR GRAFT	2 (0.0)	BURSITIS INFECTIVE	2 (0.0)
HEPATITIS E	2 (0.1)	TONSILLITIS	2 (0.1)	FACIAL PAIN	2 (0.0)	HYPOXIA	2 (0.0)
HEPATITIS CHOLESTATIC	2 (0.1)	INFUSION	2 (0.1)	PERICARDITIS	2 (0.0)	PROSTATIC SPECIFIC ANTIGEN INCREASED	2 (0.0)
CRANIOFACIAL FRACTURE	1 (0.0)	SPINAL DECOMPRESSION	2 (0.1)	GASTROINTESTINAL PROCEDURAL COMPLICATION	2 (0.0)	EYE DISCHARGE	2 (0.0)
TOE OPERATION	1 (0.0)	HAEMORRHAGIC STROKE	2 (0.1)	PARAESTHESIA ORAL	2 (0.0)	GROIN PAIN	2 (0.0)
FACIAL OPERATION	1 (0.0)	BLISTER RUPTURE	2 (0.1)	TONGUE DISCOMFORT	2 (0.0)	TESTIS CANCER	2 (0.0)
SCAPULA FRACTURE	1 (0.0)	DENTAL PROSTHESIS PLACEMENT	2 (0.1)	FISTULA INFLAMMATION	2 (0.0)	PROCTOCOLECTOMY	2 (0.0)
BONE MARROW TRANSPLANT	1 (0.0)	LEFT VENTRICULAR HYPERTROPHY	2 (0.1)	HYPOPHOSPHATAEMIA	2 (0.0)	CORONARY ARTERY BYPASS	2 (0.0)
NEUROENDOCRINE TUMOUR	1 (0.0)	RHINOVIRUS INFECTION	2 (0.1)	HEPATITIS E	2 (0.0)	ANIMAL BITE	2 (0.0)
CARCINOID TUMOUR	1 (0.0)	HEPATIC CANCER METASTATIC	2 (0.1)	HEPATITIS CHOLESTATIC	2 (0.0)	BRONCHIECTASIS	2 (0.0)
APPENDIX DISORDER	1 (0.0)	HERPES SIMPLEX	2 (0.1)	POSTOPERATIVE ADHESION	2 (0.0)	DRUG INEFFECTIVE FOR UNAPPROVE	2 (0.0)

						D INDICATION	
MYASTHENIA GRAVIS	1 (0.0)	CEREBRAL DISORDER	2 (0.1)	GALLBLADDER DISORDER	2 (0.0)	PRESYNCOPE	2 (0.0)
OSTEOPOROSIS	1 (0.0)	DISTURBANCE IN ATTENTION	2 (0.1)	CEREBRAL DISORDER	2 (0.0)	HYPOVITAMINOSIS	2 (0.0)
HAEMOGLOBIN INCREASED	1 (0.0)	BLOOD CREATININE ABNORMAL	2 (0.1)	DELUSION	2 (0.0)	DISORIENTATION	2 (0.0)
INJURY	1 (0.0)	IMPAIRED DRIVING ABILITY	2 (0.1)	HYPOCALCAEMIA	2 (0.0)	INCISION SITE IMPAIRED HEALING	2 (0.0)
LYMPHOCYTE COUNT INCREASED	1 (0.0)	MINERAL SUPPLEMENTATION	2 (0.1)	STOMACH MASS	2 (0.0)	PRODUCT USE IN UNAPPROVED INDICATION	2 (0.0)
NECROSIS	1 (0.0)	THROMBOCYTOPENIA	2 (0.1)	CANCER PAIN	2 (0.0)	URINE ANALYSIS ABNORMAL	2 (0.0)
PULMONARY IMAGING PROCEDURE ABNORMAL	1 (0.0)	INTRACRANIAL ANEURYSM	2 (0.1)	FOOT DEFORMITY	2 (0.0)	OVERWEIGHT	2 (0.0)
BLADDER DIVERTICULUM	1 (0.0)	GASTRIC ULCER PERFORATION	2 (0.1)	LYMPHADENECTOMY	2 (0.0)	SENSITIVE SKIN	2 (0.0)
BURSITIS	1 (0.0)	GINGIVAL BLEEDING	2 (0.1)	PRE-EXISTING CONDITION IMPROVED	2 (0.0)	RASH PUSTULAR	2 (0.0)
SPONDYLITIS	1 (0.0)	SMALL INTESTINAL HAEMORRHAGE	2 (0.1)	CRANIOFACIAL FRACTURE	2 (0.0)	EJECTION FRACTION DECREASED	2 (0.0)
MANIA	1 (0.0)	FAECAL CALPROTECTIN ABNORMAL	2 (0.1)	LATENT TUBERCULOSIS	2 (0.0)	RENAL MASS	2 (0.0)

POLYPECTOMY	1 (0.0)	DRUG INTERACTION	2 (0.1)	GROIN PAIN	2 (0.0)	STENOSIS	2 (0.0)
GESTATIONAL DIABETES	1 (0.0)	PARALYSIS	2 (0.1)	INGUINAL MASS	2 (0.0)	SOCIAL PROBLEM	2 (0.0)
PROLONGED LABOUR	1 (0.0)	MULTIPLE FRACTURES	2 (0.1)	INTESTINAL ISCHAEMIA	2 (0.0)	SCIATIC NERVE NEUROPATHY	2 (0.0)
ABNORMAL BEHAVIOUR	1 (0.0)	COMPRESSION FRACTURE	2 (0.1)	EMOTIONAL DISORDER	2 (0.0)	AORTIC ANEURYSM RUPTURE	2 (0.0)
GRANDIOSITY	1 (0.0)	OVARIAN CYST	2 (0.1)	BACTERAEMIA	2 (0.0)	HEART RATE ABNORMAL	2 (0.0)
AGITATION	1 (0.0)	ARTERIOVENOUS MALFORMATION	2 (0.1)	GASTRIC PERFORATION	2 (0.0)	PHARYNGEAL MASS	2 (0.0)
DISORGANISED SPEECH	1 (0.0)	PAIN IN JAW	2 (0.1)	ABNORMAL WEIGHT GAIN	2 (0.0)	PHARYNGITIS	2 (0.0)
MENISCUS OPERATION	1 (0.0)	ODYNOPHAGIA	2 (0.1)	ABNORMAL DREAMS	2 (0.0)	CEREBRAL HAEMORRHAGE	2 (0.0)
POST PROCEDURAL PULMONARY EMBOLISM	1 (0.0)	WOUND INFECTION	2 (0.1)	INFUSION	2 (0.0)	BRAIN NEOPLASM MALIGNANT	2 (0.0)
MEDICAL DIET	1 (0.0)	BLADDER OPERATION	2 (0.1)	STOMA COMPLICATION	2 (0.0)	DIASTOLIC DYSFUNCTION	2 (0.0)
ENDOSCOPY SMALL INTESTINE	1 (0.0)	FULL BLOOD COUNT INCREASED	2 (0.1)	ORAL FUNGAL INFECTION	2 (0.0)	MYELITIS	2 (0.0)
RETINAL ARTERY OCCLUSION	1 (0.0)	DISORIENTATION	2 (0.1)	MUSCLE TWITCHING	2 (0.0)	PHARYNGEAL DISORDER	2 (0.0)
MACULAR ISCHAEMIA	1 (0.0)	BLOOD TEST ABNORMAL	2 (0.1)	EXPOSURE TO COMMUNICABLE DISEASE	2 (0.0)	RED BLOOD CELL SEDIMENTATION RATE	2 (0.0)

INTESTINAL PROLAPSE	1 (0.0)	FOOD ALLERGY	2 (0.1)	LOWER LIMB FRACTURE	2 (0.0)	INCREASED STREPTOCOCCAL INFECTION	2 (0.0)
GENITAL INFECTION BACTERIAL	1 (0.0)	PANIC ATTACK	2 (0.1)	FRACTURE	2 (0.0)	LYMPHOPENIA	2 (0.0)
HYPERCOAGULATION	1 (0.0)	ALLERGIC COUGH	2 (0.1)	ANAL PRURITUS	2 (0.0)	PANCREATIC CYST	2 (0.0)
SUBCLAVIAN VEIN OCCLUSION	1 (0.0)	GASTRIC ULCER HAEMORRHAGE	2 (0.1)	MEDICAL DEVICE IMPLANTATION	2 (0.0)	HIGH DENSITY LIPOPROTEIN DECREASED	2 (0.0)
SUBCLAVIAN VEIN THROMBOSIS	1 (0.0)	CHONDROCALCINOSIS	2 (0.1)	WOUND NECROSIS	2 (0.0)	URINE FLOW DECREASED	2 (0.0)
VASCULAR DEVICE OCCLUSION	1 (0.0)	JOINT EFFUSION	2 (0.1)	LEUKAEMIA	2 (0.0)	NODULE	2 (0.0)
JUGULAR VEIN OCCLUSION	1 (0.0)	HALLUCINATION	2 (0.1)	ANAPHYLACTIC SHOCK	2 (0.0)	CRYING	2 (0.0)
ANAL INFECTION	1 (0.0)	SARCOIDOSIS	2 (0.1)	GASTRIC CANCER	2 (0.0)	PNEUMONIA CRYPTOCOCCAL	2 (0.0)
HYPOALBUMINAEMIA	1 (0.0)	PLANTAR FASCIITIS	2 (0.1)	OESOPHAGITIS	2 (0.0)	SURGICAL FAILURE	2 (0.0)
TRANSFERRIN SATURATION DECREASED	1 (0.0)	ANEURYSM	2 (0.1)	DEAFNESS UNILATERAL	2 (0.0)	BONE CONTUSION	2 (0.0)
EOSINOPHIL COUNT DECREASED	1 (0.0)	TENDONITIS	2 (0.1)	DISCHARGE	2 (0.0)	CEREBRAL DISORDER	2 (0.0)
RECTAL FISSURE	1 (0.0)	MUSCLE RUPTURE	2 (0.1)	DEMYELINATION	2 (0.0)	BLADDER CANCER	2 (0.0)
ERYTHEMA NODOSUM	1 (0.0)	HAEMATEMESIS	2 (0.1)	INCREASED TENDENCY TO BRUISE	2 (0.0)	HEPATIC CANCER METASTATIC	2 (0.0)
BLOOD PHOSPHOR	1 (0.0)	HAEMATURIA	2 (0.1)	HEPATOMEGALY	2 (0.0)	COLON CANCER	2 (0.0)

US DECREASED						METASTATIC	
FISTULA DISCHARGE	1 (0.0)	ACROCHORDON	2 (0.1)	FISTULA REPAIR	2 (0.0)	PERIORBITAL CELLULITIS	2 (0.0)
VITAL FUNCTIONS ABNORMAL	1 (0.0)	MITRAL VALVE PROLAPSE	2 (0.1)	POST PROCEDURAL SEPSIS	2 (0.0)	PNEUMONIA LEGIONELLA	2 (0.0)
LUMBAR PUNCTURE ABNORMAL	1 (0.0)	LACUNAR STROKE	2 (0.1)	ESCHERICHIA URINARY TRACT INFECTION	2 (0.0)	PSOAS ABSCESS	2 (0.0)
CYTOMEGALOVIRUS INFECTION REACTIVATION	1 (0.0)	RENAL CYST	2 (0.1)	FACIAL ASYMMETRY	2 (0.0)	PAPULE	2 (0.0)
LARGE INTESTINAL ULCER HAEMORRHAGE	1 (0.0)	POSTOPERATIVE WOUND INFECTION	2 (0.1)	PARANASAL SINUS DISCOMFORT	2 (0.0)	DIALYSIS	2 (0.0)
COLOSTOMY INFECTION	1 (0.0)	EAR NEOPLASM	2 (0.1)	BLOOD UREA INCREASED	2 (0.0)	PROCEDURAL INTESTINAL PERFORATION	2 (0.0)
GLOSSODYNIA	1 (0.0)	BODY TEMPERATURE DECREASED	2 (0.1)	GINGIVAL ULCERATION	2 (0.0)	DEPRESSED MOOD	2 (0.0)
TONGUE INJURY	1 (0.0)	WOUND COMPLICATION	2 (0.1)	HERPES SIMPLEX	2 (0.0)	AORTIC THROMBOSIS	2 (0.0)
TONGUE NEOPLASM MALIGNANT STAGE UNSPECIFIED	1 (0.0)	VISUAL FIELD DEFECT	2 (0.1)	GLOSSODYNIA	2 (0.0)	PRE-EXISTING CONDITION IMPROVED	2 (0.0)
MENOPAUSAL SYMPTOMS	1 (0.0)	SALMONELLOSIS	2 (0.1)	THERAPEUTIC RESPONSE SHORTENED	2 (0.0)	ANKLE OPERATION	2 (0.0)
OBSTRUCTI	1 (0.0)	PROSTATEC	2 (0.1)	GASTROINTE	2	ABDOMINAL	2 (0.0)

VE AIRWAYS DISORDER		TOMY		STINAL EROSION	(0.0)	OPERATION	
PHARYNGEAL DISORDER	1 (0.0)	COMA	2 (0.1)	GASTROINTESTINAL MUCOSA HYPERAEMIA	2 (0.0)	BLADDER OPERATION	2 (0.0)
THERAPEUTIC PROCEDURE	1 (0.0)	CRYING	2 (0.1)	TOOTH LOSS	2 (0.0)	POST PROCEDURAL INFLAMMATION	2 (0.0)
LOW CARBOHYDRATE DIET	1 (0.0)	EYE INFECTION	1 (0.0)	LIMB INJURY	2 (0.0)	DECUBITUS ULCER	2 (0.0)
PNEUMONIA NECROTISING	1 (0.0)	HELICOBACTER INFECTION	1 (0.0)	FULL BLOOD COUNT ABNORMAL	2 (0.0)	ACNE FULMINANS	2 (0.0)
DENTAL IMPLANTATION	1 (0.0)	PLATELET TRANSFUSION	1 (0.0)	EMBOLISM VENOUS	2 (0.0)	ROTATOR CUFF REPAIR	2 (0.0)
COMPLICATION ASSOCIATED WITH DEVICE	1 (0.0)	STUPOR	1 (0.0)	PRODUCT PRESCRIBING ISSUE	2 (0.0)	ORAL SURGERY	2 (0.0)
POST PROCEDURAL FEVER	1 (0.0)	PNEUMONIA BACTERIAL	1 (0.0)	OESOPHAGEAL DYSPLASIA	2 (0.0)	HEPATIC VEIN THROMBOSIS	2 (0.0)
BILE DUCT STONE	1 (0.0)	INCREASED UPPER AIRWAY SECRETION	1 (0.0)	URINARY BLADDER HAEMORRHAGE	2 (0.0)	WHITE BLOOD CELL DISORDER	2 (0.0)
VERTEBROBASILAR ARTERY DISSECTION	1 (0.0)	PARANASAL CYST	1 (0.0)	TENOPLASTY	2 (0.0)	WHEEZING	2 (0.0)
GINGIVITIS	1 (0.0)	LIGAMENTITIS	1 (0.0)	DEVICE LOOSENING	2 (0.0)	TENDONITIS	2 (0.0)
BRONCHOSPASM	1 (0.0)	ENDOMETRIAL ADENOCARCINOMA	1 (0.0)	PUSTULAR PSORIASIS	2 (0.0)	THROAT CANCER	2 (0.0)

HYPOGLYCAEMIA	1 (0.0)	CHRONIC RESPIRATORY FAILURE	1 (0.0)	LUPUS-LIKE SYNDROME	2 (0.0)	CERVIX CARCINOMA	2 (0.0)
GASTROENTEROSTOMY	1 (0.0)	ORTHOPNOEA	1 (0.0)	LIMB OPERATION	2 (0.0)	HAND-FOOT-AND-MOUTH DISEASE	2 (0.0)
LIVEDO RETICULARIS	1 (0.0)	THYROID CALCIFICATION	1 (0.0)	DELIRIUM	2 (0.0)	SCOLIOSIS	2 (0.0)
RASH PUSTULAR	1 (0.0)	RIGHT VENTRICULAR SYSTOLIC PRESSURE INCREASED	1 (0.0)	PNEUMONIA FUNGAL	2 (0.0)	LYMPHOCYTE COUNT ABNORMAL	2 (0.0)
CLOSTRIDIAL INFECTION	1 (0.0)	WOUND SEPSIS	1 (0.0)	UPPER-AIRWAY COUGH SYNDROME	2 (0.0)	FULL BLOOD COUNT INCREASED	2 (0.0)
INTRA-ABDOMINAL HAEMORRHAGE	1 (0.0)	MATERNAL EXPOSURE DURING PREGNANCY	1 (0.0)	SPINAL COMPRESSION FRACTURE	2 (0.0)	CARDIAC STRESS TEST ABNORMAL	2 (0.0)
SOMATIC SYMPTOM DISORDER	1 (0.0)	LEUKAEMIA	1 (0.0)	ILEUS PARALYTIC	2 (0.0)	PURPURA FULMINANS	2 (0.0)
BACTERIAL TEST POSITIVE	1 (0.0)	VASCULAR GRAFT	1 (0.0)	BLOOD ALBUMIN DECREASED	2 (0.0)	DEVICE ISSUE	2 (0.0)
POST PROCEDURAL CONTUSION	1 (0.0)	MENTAL DISORDER	1 (0.0)	MEAN CELL HAEMOGLOBIN CONCENTRATION DECREASED	2 (0.0)	MUSCLE RUPTURE	2 (0.0)
GUILLAIN-BARRE SYNDROME	1 (0.0)	SPINAL CORD COMPRESSION	1 (0.0)	INTESTINAL SEPSIS	2 (0.0)	ABDOMINAL INFECTION	2 (0.0)
INFUSION SITE SWELLING	1 (0.0)	BRONCHOPULMONARY ASPERGILLOSIS	1 (0.0)	LEFT ATRIAL APPENDAGE CLOSURE IMPLANT	2 (0.0)	EAR SWELLING	2 (0.0)
BLOOD BILIRUBIN	1 (0.0)	EMOTIONAL DISORDER	1 (0.0)	WOUND HAEMORRH	2 (0.0)	HEPATIC VASCULAR	2 (0.0)

ABNORMAL				AGE		THROMBOSIS	
PARASITE STOOL TEST POSITIVE	1 (0.0)	FLANK PAIN	1 (0.0)	FACIAL PARALYSIS	2 (0.0)	PERIPHERAL ISCHAEMIA	2 (0.0)
PLATELET COUNT DECREASED	1 (0.0)	THROAT IRRITATION	1 (0.0)	FISTULA OF SMALL INTESTINE	2 (0.0)	CENTRAL VENOUS CATHETERISATION	2 (0.0)
LIPIDS ABNORMAL	1 (0.0)	LAPAROSCOPIC SURGERY	1 (0.0)	PELVIC VENOUS THROMBOSIS	2 (0.0)	MOOD SWINGS	2 (0.0)
CHRONIC KIDNEY DISEASE	1 (0.0)	ORGAN FAILURE	1 (0.0)	HEPATITIS	2 (0.0)	MENSTRUATION DELAYED	2 (0.0)
DISCHARGE	1 (0.0)	VULVOVAGINAL SWELLING	1 (0.0)	HAEMOLYSIS	2 (0.0)	PAIN IN JAW	2 (0.0)
HAEMORRHAGE SUBCUTANEOUS	1 (0.0)	WEIGHT FLUCTUATION	1 (0.0)	TONGUE NEOPLASM MALIGNANT STAGE UNSPECIFIED	2 (0.0)	ANAL STENOSIS	2 (0.0)
SPLENOMEGALY	1 (0.0)	METASTASES TO PERITONEUM	1 (0.0)	LYME DISEASE	2 (0.0)	ABDOMINAL TENDERNESS	2 (0.0)
PNEUMONIA BACTERIAL	1 (0.0)	ADENOCARCINOMA	1 (0.0)	RECTAL FISSURE	2 (0.0)	HAEMATEMESIS	2 (0.0)
IRON DEFICIENCY ANAEMIA	1 (0.0)	METASTASES TO LIVER	1 (0.0)	SLUGGISHNESS	2 (0.0)	LIPOMA	2 (0.0)
MICROCYTIC ANAEMIA	1 (0.0)	LACTIC ACIDOSIS	1 (0.0)	SKIN WARM	2 (0.0)	BLADDER CANCER RECURRENT	2 (0.0)
INFUSION SITE REACTION	1 (0.0)	GASTROINTESTINAL SURGERY	1 (0.0)	VENOUS THROMBOSIS LIMB	2 (0.0)	AGEUSIA	2 (0.0)
BLOOD CHOLESTEROL ABNORMAL	1 (0.0)	NERVOUS SYSTEM DISORDER	1 (0.0)	ANGINA PECTORIS	2 (0.0)	MENSTRUAL DISORDER	2 (0.0)

ANAL SPASM	1 (0.0)	MULTIPLE SCLEROSIS	1 (0.0)	TONSILLAR HAEMORRHAGE	2 (0.0)	SPIDER VEIN	2 (0.0)
SPUTUM INCREASED	1 (0.0)	BENIGN NEOPLASM OF URETER	1 (0.0)	EYE IRRITATION	2 (0.0)	VULVOVAGINAL MYCOTIC INFECTION	2 (0.0)
SEMEN VISCOSITY ABNORMAL	1 (0.0)	RAYNAUD'S PHENOMENON	1 (0.0)	BLOOD ALKALINE PHOSPHATASE INCREASED	2 (0.0)	CLOSTRIDIUM DIFFICILE COLITIS	2 (0.0)
SEMEN ANALYSIS ABNORMAL	1 (0.0)	LYMPHOMA	1 (0.0)	CORONARY ARTERIAL STENT INSERTION	2 (0.0)	ABDOMINAL RIGIDITY	2 (0.0)
MEDICAL DEVICE PAIN	1 (0.0)	PROSTATITIS	1 (0.0)	LARGE INTESTINE ANASTOMOSIS	2 (0.0)	SPLENOMEGALY	2 (0.0)
FRUSTRATION TOLERANCE DECREASED	1 (0.0)	JOINT LOCK	1 (0.0)	POST PROCEDURAL SWELLING	2 (0.0)	HEPATIC FUNCTION ABNORMAL	2 (0.0)
SPUTUM DISCOLOURED	1 (0.0)	BILIARY TRACT DISORDER	1 (0.0)	DYSGEUSIA	2 (0.0)	PERITONITIS	2 (0.0)
SELECTIVE ABORTION	1 (0.0)	PROSTATIC ABSCESS	1 (0.0)	DISEASE RECURRENCE	2 (0.0)	MUSCLE TIGHTNESS	2 (0.0)
ACNE PUSTULAR	1 (0.0)	THYROID OPERATION	1 (0.0)	INTESTINAL PROLAPSE	2 (0.0)	SCRATCH	2 (0.0)
DANDRUFF	1 (0.0)	UTERINE DILATION AND CURETTAGE	1 (0.0)	FIBROSIS	2 (0.0)	FEBRILE NEUTROPENIA	2 (0.0)
ABNORMAL WEIGHT GAIN	1 (0.0)	INCISIONAL DRAINAGE	1 (0.0)	POLYCHONDRITIS	2 (0.0)	PROCEDURAL HAEMORRHAGE	2 (0.0)
HYPERALDOSTERONISM	1 (0.0)	ALLERGIC RESPIRATORY SYMPTOM	1 (0.0)	ATYPICAL PNEUMONIA	2 (0.0)	CARDIOMYOPATHY ACUTE	2 (0.0)

BLOOD POTASSIUM ABNORMAL	1 (0.0)	FAILURE TO THRIVE	1 (0.0)	SPINAL OSTEOARTHRITIS	2 (0.0)	PERIPHERAL NERVE INJURY	2 (0.0)
ATROPHY	1 (0.0)	MYOCARDIAL NECROSIS MARKER INCREASED	1 (0.0)	PROCEDURAL HAEMORRHAGE	2 (0.0)	SKIN HAEMORRHAGE	2 (0.0)
TRIGGER FINGER	1 (0.0)	OVERDOSE	1 (0.0)	ANASTOMOTIC STENOSIS	2 (0.0)	PRODUCT TEMPERATURE EXCURSION ISSUE	2 (0.0)
VEIN DISORDER	1 (0.0)	VASCULAR OCCLUSION	1 (0.0)	GENERAL SYMPTOM	2 (0.0)	ORAL DISCOMFORT	2 (0.0)
MYOPERICARDITIS	1 (0.0)	TOOTH LOSS	1 (0.0)	MULTIPLE ALLERGIES	2 (0.0)	CORONARY ARTERY OCCLUSION	2 (0.0)
DRUG INEFFECTIVE FOR UNAPPROVED INDICATION	1 (0.0)	ASPERGILLOMA	1 (0.0)	PERICARDIAL EFFUSION	2 (0.0)	RENAL ABSCESS	2 (0.0)
INTERMENSTRUAL BLEEDING	1 (0.0)	GASTROINTESTINAL ULCER HAEMORRHAGE	1 (0.0)	DYSURIA	2 (0.0)	PANIC REACTION	2 (0.0)
COLORECTAL ADENOMA	1 (0.0)	HAEMATOCRIT ABNORMAL	1 (0.0)	LIVER ABSCESS	2 (0.0)	PROSTHESIS IMPLANTATION	2 (0.0)
ORCHIDECTOMY	1 (0.0)	COMPLICATION ASSOCIATED WITH DEVICE	1 (0.0)	AGITATION	2 (0.0)	VARICOSE VEIN	2 (0.0)
SOMNOLENCE	1 (0.0)	LEUKAEMIA RECURRENT	1 (0.0)	DISEASE PROGRESSION	2 (0.0)	PULMONARY CONGESTION	2 (0.0)
GLOMERULAR	1 (0.0)	LYMPHOCYTE COUNT	1 (0.0)	ILEOSTOMY CLOSURE	2 (0.0)	DRUG DEPENDENC	2 (0.0)

FILTRATION RATE DECREASED		ABNORMAL				E	
BLOOD ALKALINE PHOSPHATASE ABNORMAL	1 (0.0)	RED BLOOD CELL COUNT ABNORMAL	1 (0.0)	TROPONIN INCREASED	2 (0.0)	MULTIPLE ALLERGIES	2 (0.0)
RENAL IMPAIRMENT	1 (0.0)	DYSARTHRIA	1 (0.0)	BACTERIAL TEST POSITIVE	2 (0.0)	DIPLOPIA	2 (0.0)
INFUSION	1 (0.0)	MONTREAL COGNITIVE ASSESSMENT ABNORMAL	1 (0.0)	ENCEPHALITIS	2 (0.0)	CEREBRAL THROMBOSIS	2 (0.0)
TENDON DISORDER	1 (0.0)	TANDEM GAIT TEST ABNORMAL	1 (0.0)	BRAIN OEDEMA	2 (0.0)	MYELOSUPPRESSION	2 (0.0)
RENAL INFARCT	1 (0.0)	NASAL SEPTUM DEVIATION	1 (0.0)	ADENOCARCINOMA OF COLON	2 (0.0)	SKIN INFECTION	2 (0.0)
LIGAMENT INJURY	1 (0.0)	CALCIPHYLAXIS	1 (0.0)	PRODUCT PHYSICAL ISSUE	2 (0.0)	ALLERGIC COUGH	2 (0.0)
BACTERAEMIA	1 (0.0)	NYSTAGMUS	1 (0.0)	LIVER FUNCTION TEST ABNORMAL	2 (0.0)	APPENDIX CANCER	2 (0.0)
TREATMENT FAILURE	1 (0.0)	AGRANULOCYTOSIS	1 (0.0)	NEUTROPENIA	2 (0.0)	MYOCARDITIS	2 (0.0)
POSTURAL ORTHOSTATIC TACHYCARDIA SYNDROME	1 (0.0)	DYSKINESIA	1 (0.0)	PORTAL VEIN THROMBOSIS	2 (0.0)	PROSTATECTOMY	2 (0.0)
HEPATITIS B CORE ANTIBODY POSITIVE	1 (0.0)	SACRAL PAIN	1 (0.0)	MUCOSAL ULCERATION	2 (0.0)	INCISION SITE PAIN	2 (0.0)
HEPATITIS B SURFACE ANTIBODY	1 (0.0)	EPSTEIN-BARR VIRUS ASSOCIATE	1 (0.0)	CHRONIC LYMPHOCYTIC	2 (0.0)	LIMB MASS	2 (0.0)

POSITIVE		D LYMPHOPROLIFERATIVE DISORDER		LEUKAEMIA			
HYPOCALCAEMIA	1 (0.0)	SKIN DISCOMFORT	1 (0.0)	PERIORBITAL SWELLING	2 (0.0)	PARANASAL SINUS DISCOMFORT	2 (0.0)
RESPIRATORY ARREST	1 (0.0)	EXFOLIATIVE RASH	1 (0.0)	CARDIAC MURMUR	2 (0.0)	HEAVY EXPOSURE TO ULTRAVIOLET LIGHT	2 (0.0)
TANNING	1 (0.0)	VERTIGO POSITIONAL	1 (0.0)	COMPUTERISED TOMOGRAM ABNORMAL	2 (0.0)	YERSINIA INFECTION	2 (0.0)
SCAB	1 (0.0)	STRESS FRACTURE	1 (0.0)	MACULAR DEGENERATION	2 (0.0)	FRACTURED SACRUM	2 (0.0)
LIMB MASS	1 (0.0)	ANAL INFLAMMATION	1 (0.0)	ABDOMINAL MASS	2 (0.0)	TASTE DISORDER	2 (0.0)
CATHETER SITE THROMBOSIS	1 (0.0)	BONE MARROW DISORDER	1 (0.0)	GROIN ABSCESS	2 (0.0)	DRUG EFFECT LESS THAN EXPECTED	2 (0.0)
CATHETER REMOVAL	1 (0.0)	LEFT VENTRICULAR FAILURE	1 (0.0)	WALKING AID USER	2 (0.0)	EXPOSURE TO SARS-COV-2	2 (0.0)
BREAST RECONSTRUCTION	1 (0.0)	BONE LOSS	1 (0.0)	ORCHITIS	2 (0.0)	DYSGEUSIA	2 (0.0)
VOLVULUS	1 (0.0)	PROCEDURAL INTESTINAL PERFORATION	1 (0.0)	INTERMITTENT CLAUDICATION	2 (0.0)	HIATUS HERNIA	2 (0.0)
TRANSAMINASES INCREASED	1 (0.0)	UTERINE ENLARGEMENT	1 (0.0)	EYE MOVEMENT DISORDER	2 (0.0)	SCHIZOPHRENIA	2 (0.0)
HEPATOCELLULAR INJURY	1 (0.0)	UTERINE TENDERNESS	1 (0.0)	GASTRIC DILATATION	2 (0.0)	INTESTINAL ULCER	2 (0.0)

FUSOBACTERIUM INFECTION	1 (0.0)	ENTEROCOLITIS	1 (0.0)	HYPERSOMNIA	2 (0.0)	MENINGITIS BACTERIAL	2 (0.0)
ATRIAL SEPTAL DEFECT	1 (0.0)	BLOOD LOSS ANAEMIA	1 (0.0)	COGNITIVE DISORDER	2 (0.0)	THYROID CANCER	2 (0.0)
ISCHAEMIC STROKE	1 (0.0)	OSTEOMYELITIS BACTERIAL	1 (0.0)	SLEEP DEFICIT	2 (0.0)	BLISTER RUPTURE	2 (0.0)
ROTATOR CUFF SYNDROME	1 (0.0)	ANAL DILATION PROCEDURE	1 (0.0)	ENDOMETRIOSIS	2 (0.0)	CAMPYLOBACTER GASTROENTERITIS	2 (0.0)
SKIN BACTERIAL INFECTION	1 (0.0)	DECUBITUS ULCER	1 (0.0)	DIALYSIS	2 (0.0)	CORONARY ARTERY STENOSIS	2 (0.0)
LACRIMATION INCREASED	1 (0.0)	VOCAL CORD DISORDER	1 (0.0)	CYANOSIS	2 (0.0)	LEFT VENTRICULAR HYPERTROPHY	2 (0.0)
SINUS OPERATION	1 (0.0)	SUNBURN	1 (0.0)	BLOOD CHOLESTEROL ABNORMAL	2 (0.0)	VENOUS OCCLUSION	2 (0.0)
COVID-19 PNEUMONIA	1 (0.0)	RASH ERYTHEMATOUS	1 (0.0)	SENSITIVE SKIN	2 (0.0)	CELLULITE	2 (0.0)
CYTOGENETIC ABNORMALITY	1 (0.0)	LENTIGO MALIGNA	1 (0.0)	NEUTROPHIL COUNT ABNORMAL	2 (0.0)	MINERAL SUPPLEMENTATION	2 (0.0)
UTERINE LEIOMYOMA	1 (0.0)	COLONOSCOPY	1 (0.0)	SINUS CONGESTION	2 (0.0)	HELICOBACTER INFECTION	2 (0.0)
GENERALISED TONIC-CLONIC SEIZURE	1 (0.0)	ATAXIA	1 (0.0)	URINARY INCONTINENCE	2 (0.0)	VIRAEMIA	2 (0.0)
FOCAL DYSCOGNITIVE SEIZURES	1 (0.0)	ARTHROPOD INFESTATION	1 (0.0)	LIP ULCERATION	2 (0.0)	PYELONEPHRITIS	2 (0.0)

DIABETIC KETOACIDOSIS	1 (0.0)	PROCEDURAL VOMITING	1 (0.0)	LIVER DISORDER	2 (0.0)	DYSARTHRIA	2 (0.0)
ESCHERICHIA BACTERAEMIA	1 (0.0)	MENINGITIS BACTERIAL	1 (0.0)	INTESTINAL ANASTOMOSIS COMPLICATION	2 (0.0)	TACHYPNOEA	2 (0.0)
OTITIS MEDIA	1 (0.0)	HEPATOCELLULAR CARCINOMA	1 (0.0)	LIP DISCOLOURATION	2 (0.0)	BLOOD BLISTER	2 (0.0)
HERPES ZOSTER OTICUS	1 (0.0)	CERVIX WARTS	1 (0.0)	LIP PAIN	2 (0.0)	WEIGHT ABNORMAL	2 (0.0)
PERINEAL CYST	1 (0.0)	EMOTIONAL DISTRESS	1 (0.0)	IMPAIRED DRIVING ABILITY	2 (0.0)	PROSTATE CANCER METASTATIC	2 (0.0)
FLAP SURGERY	1 (0.0)	CYSTITIS NONINFECTIVE	1 (0.0)	ANAPHYLACTIC REACTION	2 (0.0)	ADENOMA BENIGN	2 (0.0)
HEPATITIS E ANTIBODY POSITIVE	1 (0.0)	ORCHITIS	1 (0.0)	ADVERSE FOOD REACTION	2 (0.0)	ORAL PRURITUS	2 (0.0)
PSYCHOTIC DISORDER	1 (0.0)	THERAPEUTIC RESPONSE SHORTENED	1 (0.0)	FEAR	2 (0.0)	ENTEROCOLITIS VIRAL	2 (0.0)
OCULAR ICTERUS	1 (0.0)	INTENTIONAL PRODUCT USE ISSUE	1 (0.0)	MENTAL IMPAIRMENT	2 (0.0)	MOUTH SWELLING	2 (0.0)
RETINAL DETACHMENT	1 (0.0)	SKIN PLAQUE	1 (0.0)	FIBRIN D DIMER INCREASED	2 (0.0)	DIZZINESS POSTURAL	2 (0.0)
INSURANCE ISSUE	1 (0.0)	DIPLOPIA	1 (0.0)	MUSCULOSKELETAL PAIN	2 (0.0)	TIBIA FRACTURE	2 (0.0)
PRODUCT DOSE OMISSION ISSUE	1 (0.0)	AKINESIA	1 (0.0)	SHOULDER ARTHROPLASTY	2 (0.0)	HEPATOTOXICITY	2 (0.0)
FOCAL PERITONITIS	1 (0.0)	HYPOKINESIA	1 (0.0)	BREAST HYPERPLASIA	2 (0.0)	BLINDNESS UNILATERAL	2 (0.0)
EXTREMITY NECROSIS	1 (0.0)	WISDOM TEETH	1 (0.0)	ANGER	2 (0.0)	LIMB DISCOMFOR	2 (0.0)

		REMOVAL				T	
DRUG INTOLERANCE	1 (0.0)	CAMPYLOBACTER GASTROENTERITIS	1 (0.0)	BODY TEMPERATURE DECREASED	2 (0.0)	FIBROMYALGIA	2 (0.0)
SKIN OPERATION	1 (0.0)	FEELING OF BODY TEMPERATURE CHANGE	1 (0.0)	PRESYNCOPE	2 (0.0)	MICROCYTIC ANAEMIA	2 (0.0)
RED BLOOD CELL TRANSFUSION	1 (0.0)	COLON CANCER METASTATIC	1 (0.0)	SCHIZOPHRENIA	2 (0.0)	PERIPHERAL COLDNESS	2 (0.0)
SPLEEN DISORDER	1 (0.0)	SERUM FERRITIN ABNORMAL	1 (0.0)	VENA CAVA THROMBOSIS	2 (0.0)	HYPERTENSIVE CRISIS	2 (0.0)
MUSCLE STRAIN	1 (0.0)	HAEMANGIOMA OF LIVER	1 (0.0)	MOANING	2 (0.0)	FOREIGN BODY IN GASTROINTESTINAL TRACT	2 (0.0)
PSYCHOMOTOR RETARDATION	1 (0.0)	LYME DISEASE	1 (0.0)	CLOSTRIDIUM DIFFICILE COLITIS	2 (0.0)	BLOOD LACTATE DEHYDROGENASE INCREASED	2 (0.0)
SPEECH DISORDER DEVELOPMENTAL	1 (0.0)	TREATMENT NONCOMPLIANCE	1 (0.0)	PURPURA FULMINANS	2 (0.0)	THERAPEUTIC PRODUCT EFFECT VARIABLE	2 (0.0)
STOMA SITE DISCHARGE	1 (0.0)	ANASTOMOTIC HAEMORRHAGE	1 (0.0)	OESOPHAGEAL PAIN	2 (0.0)	LUNG NEOPLASM MALIGNANT	2 (0.0)
RECTOSIGMOID CANCER METASTATIC	1 (0.0)	LOWER GASTROINTESTINAL HAEMORRHAGE	1 (0.0)	HERPES SIMPLEX ENCEPHALITIS	2 (0.0)	UPPER GASTROINTESTINAL HAEMORRHAGE	2 (0.0)
PNEUMATOSIS	1 (0.0)	HYPERTENSIVE CRISIS	1 (0.0)	PROSTATOMEGALY	2 (0.0)	RENAL FUNCTION TEST ABNORMAL	2 (0.0)
LIP BLISTER	1 (0.0)	CHRONIC	1 (0.0)	PROSTATIC	2	RECTAL	2 (0.0)

		LYMPHOCYTIC LEUKAEMIA		DISORDER	(0.0)	DISCHARGE	
CLAVICLE FRACTURE	1 (0.0)	MICROCYTIC ANAEMIA	1 (0.0)	DIPLOPIA	2 (0.0)	PRODUCT PRESCRIBING ERROR	2 (0.0)
APPENDICEAL MUCOCOELE	1 (0.0)	PNEUMONIA NECROTISING	1 (0.0)	BLOOD POTASSIUM ABNORMAL	2 (0.0)	SUSPECTED DRUG-INDUCED LIVER INJURY	2 (0.0)
PERIORBITAL CELLULITIS	1 (0.0)	TOOTH DISORDER	1 (0.0)	RESPIRATORY ARREST	2 (0.0)	STAPHYLOCOCCAL SEPSIS	2 (0.0)
MALIGNANT NEOPLASM OF UNKNOWN PRIMARY SITE	1 (0.0)	FACE OEDEMA	1 (0.0)	LIVER TRANSPLANT	2 (0.0)	EUPHORIC MOOD	2 (0.0)
COLON CANCER METASTATIC	1 (0.0)	EYELID OEDEMA	1 (0.0)	PROSTATITIS	2 (0.0)	TUBERCULOSIS	2 (0.0)
INTRUSIVE THOUGHTS	1 (0.0)	ACOUSTIC NEUROMA	1 (0.0)	RESPIRATORY SYNCYTIAL VIRUS INFECTION	2 (0.0)	OESOPHAGEAL ULCER	2 (0.0)
FEAR	1 (0.0)	VARICELLA	1 (0.0)	SPEECH DISORDER DEVELOPMENTAL	2 (0.0)	HYPONATRAEMIA	2 (0.0)
OBSESSIVE THOUGHTS	1 (0.0)	PULSELESS ELECTRICAL ACTIVITY	1 (0.0)	PSYCHOTIC DISORDER	2 (0.0)	SLEEP DEFICIT	2 (0.0)
DROOLING	1 (0.0)	BLOOD ALKALINE PHOSPHATASE INCREASED	1 (0.0)	GASTROINTESTINAL STOMA OUTPUT ABNORMAL	2 (0.0)	PAINFUL RESPIRATION	2 (0.0)
TOOTH INFECTION	1 (0.0)	JOINT RANGE OF MOTION DECREASED	1 (0.0)	LIPIDS ABNORMAL	2 (0.0)	STOMA SITE REACTION	2 (0.0)
ADENOCAR	1 (0.0)	SKIN INJURY	1 (0.0)	ESCHERICHI	2	LIVER	2 (0.0)

CINOMA				A SEPSIS	(0.0)	FUNCTION TEST ABNORMAL	
FEMALE GENITAL OPERATION	1 (0.0)	MUCOSAL ULCERATION	1 (0.0)	RASH ERYTHEMATOUS	2 (0.0)	PERIPHERAL VEIN THROMBOSIS	2 (0.0)
EUPHORIC MOOD	1 (0.0)	CORONARY ARTERY THROMBOSIS	1 (0.0)	COLONOSCOPY ABNORMAL	2 (0.0)	COMPLICATION ASSOCIATED WITH DEVICE	2 (0.0)
STRABISMUS	1 (0.0)	FIBROSIS	1 (0.0)	SUTURE RELATED COMPLICATION	2 (0.0)	ANEURYSM	2 (0.0)
TRISMUS	1 (0.0)	HAEMANGIOMA	1 (0.0)	ANAL INFECTION	2 (0.0)	CAROTID ARTERY OCCLUSION	2 (0.0)
DEMENTIA ALZHEIMER'S TYPE	1 (0.0)	ANAESTHETIC COMPLICATION	1 (0.0)	UVEITIS	2 (0.0)	SCAB	2 (0.0)
MENTAL IMPAIRMENT	1 (0.0)	PERIPHERAL VASCULAR DISORDER	1 (0.0)	COMPLETED SUICIDE	2 (0.0)	BLADDER DISORDER	2 (0.0)
TACHYPHRENIA	1 (0.0)	GLOSSITIS	1 (0.0)	ESCHERICHIA BACTERAEMIA	2 (0.0)	HAEMOPTYSIS	2 (0.0)
RESPIRATORY RATE INCREASED	1 (0.0)	LIP ERYTHEMA	1 (0.0)	CHEMOTHERAPY	2 (0.0)	GLYCOSYLATED HAEMOGLOBIN INCREASED	2 (0.0)
PHOTOPSIA	1 (0.0)	LICE INFESTATION	1 (0.0)	DERMATITIS CONTACT	2 (0.0)	OMENTAL INFARCTION	2 (0.0)
PARANOIA	1 (0.0)	COLORECTOSTOMY	1 (0.0)	TONGUE DISORDER	2 (0.0)	NEUROGENIC SHOCK	2 (0.0)
THYROID CANCER METASTATIC	1 (0.0)	LEGIONELLA INFECTION	1 (0.0)	STAPHYLOCOCCAL SEPSIS	2 (0.0)	RENAL CANCER	2 (0.0)

VENOUS OCCLUSION	1 (0.0)	RECTAL DISCHARGE	1 (0.0)	GRANULOMA	2 (0.0)	INTRAOCULAR PRESSURE INCREASED	2 (0.0)
FIBULA FRACTURE	1 (0.0)	ABNORMAL UTERINE BLEEDING	1 (0.0)	INTESTINAL ULCER	2 (0.0)	JAW OPERATION	2 (0.0)
HAEMORRHOIDS THROMBOSED	1 (0.0)	THERAPEUTIC PRODUCT EFFECT VARIABLE	1 (0.0)	THROMBOCYTOPENIA	2 (0.0)	MEDICAL DEVICE REMOVAL	2 (0.0)
GRANULOCYTE COUNT DECREASED	1 (0.0)	HEPATIC ENCEPHALOPATHY	1 (0.0)	CHONDROCALCINOSIS	2 (0.0)	SENSORY DISTURBANCE	2 (0.0)
CLOSTRIDIUM COLITIS	1 (0.0)	STOMA SITE ULCER	1 (0.0)	MOOD ALTERED	2 (0.0)	AORTIC DISSECTION	2 (0.0)
GASTRIC ULCER HAEMORRHAGE	1 (0.0)	CIRCULATORY COLLAPSE	1 (0.0)	AGEUSIA	2 (0.0)	EAR PAIN	2 (0.0)
ARTHROSCOPY	1 (0.0)	CATHETER SITE RELATED REACTION	1 (0.0)	STOMA SITE INFLAMMATION	2 (0.0)	EAR DISCOMFORT	2 (0.0)
TRANSVERSE SINUS THROMBOSIS	1 (0.0)	DUODENITIS	1 (0.0)	MENISCUS INJURY	2 (0.0)	ADVERSE EVENT	2 (0.0)
GASTROINTESTINAL MUCOSA HYPERAEMIA	1 (0.0)	DRAINAGE	1 (0.0)	CARPAL TUNNEL SYNDROME	2 (0.0)	LYMPHOCYTE COUNT DECREASED	2 (0.0)
SKIN ABRASION	1 (0.0)	CORNEAL ABRASION	1 (0.0)	CARDIAC FAILURE CONGESTIVE	2 (0.0)	PURULENT DISCHARGE	2 (0.0)
EYE DISORDER	1 (0.0)	HERNIA REPAIR	1 (0.0)	BREAST RECONSTRUCTION	2 (0.0)	MAST CELL ACTIVATION SYNDROME	2 (0.0)
PERIPHERAL COLDNESS	1 (0.0)	INFLAMMATORY PAIN	1 (0.0)	VITAMIN B12 DECREASED	2 (0.0)	CARDIAC OPERATION	2 (0.0)
BODY TEMPERATU	1 (0.0)	RETCHING	1 (0.0)	DERMAL CYST	2 (0.0)	LARYNGITIS	2 (0.0)

RE DECREASED							
STAPHYLOCOCCAL SEPSIS	1 (0.0)	IMPAIRED QUALITY OF LIFE	1 (0.0)	DUODENAL ULCER	2 (0.0)	TYPE 2 DIABETES MELLITUS	2 (0.0)
GASTROINTESTINAL ULCER HAEMORRHAGE	1 (0.0)	VARICELLA ZOSTER OESOPHAGITIS	1 (0.0)	OESOPHAGEAL CANDIDIASIS	2 (0.0)	EXPULSION OF MEDICATION	2 (0.0)
ALLERGY TO ARTHROPOD STING	1 (0.0)	HERPES SIMPLEX ENCEPHALITIS	1 (0.0)	NYSTAGMUS	2 (0.0)	MENTAL IMPAIRMENT	2 (0.0)
INTERLEUKIN-2 RECEPTOR INCREASED	1 (0.0)	RECTAL ULCER	1 (0.0)	ANEURYSM	2 (0.0)	STEROID DEPENDENCE	2 (0.0)
BRAIN STEM STROKE	1 (0.0)	SEPTIC PULMONARY EMBOLISM	1 (0.0)	CHOLECYSTITIS	2 (0.0)	ASCITES	2 (0.0)
ACUTE ABDOMEN	1 (0.0)	SYSTEMIC CANDIDA	1 (0.0)	ANGIOPATHY	2 (0.0)	INTERMITTENT CLAUDICATION	2 (0.0)
CHAPPED LIPS	1 (0.0)	HYPERTENSIVE HEART DISEASE	1 (0.0)	PANCREATIC FAILURE	2 (0.0)	CHEMOTHERAPY	2 (0.0)
AXILLARY PAIN	1 (0.0)	COMPUTERISED TOMOGRAM THORAX ABNORMAL	1 (0.0)	PATHOGEN RESISTANCE	2 (0.0)	ONYCHOMADESIS	2 (0.0)
SLEEP TERROR	1 (0.0)	RED BLOOD CELL ABNORMALITY	1 (0.0)	GLOMERULAR FILTRATION RATE DECREASED	2 (0.0)	VEIN DISORDER	2 (0.0)
PARKINSON'S DISEASE	1 (0.0)	SPINAL DEFORMITY	1 (0.0)	OXYGEN SATURATION DECREASED	2 (0.0)	PULMONARY VENOUS THROMBOSIS	2 (0.0)
THINKING	1 (0.0)	THYROID	1 (0.0)	HAEMOGLO	2	PULMONAR	2 (0.0)

ABNORMAL		MASS		BIN INCREASED	(0.0)	Y HAEMORRHAGE	
OESOPHAGEAL CANDIDIASIS	1 (0.0)	TENDON INJURY	1 (0.0)	FRUSTRATION TOLERANCE DECREASED	2 (0.0)	GRIP STRENGTH DECREASED	2 (0.0)
TONSILLITIS	1 (0.0)	CAROTID ARTERY DISEASE	1 (0.0)	CAMPYLOBACTER INFECTION	2 (0.0)	THERAPEUTIC PROCEDURE	2 (0.0)
MENINGITIS ASEPTIC	1 (0.0)	HEPATIC ARTERY HAEMORRHAGE	1 (0.0)	VAGINAL ABSCESS	2 (0.0)	ONYCHOMYCOSIS	2 (0.0)
COXSACKIE VIRAL INFECTION	1 (0.0)	HEPATIC ARTERY ANEURYSM	1 (0.0)	FEMUR FRACTURE	2 (0.0)	GASTRIC ULCER PERFORATION	2 (0.0)
ARTERITIS	1 (0.0)	OSTEITIS	1 (0.0)	STOMA SITE ULCER	2 (0.0)	EYE INFECTION	2 (0.0)
DERMATITIS ATOPIC	1 (0.0)	ANGER	1 (0.0)	BELL'S PALSY	2 (0.0)	EYE PRURITUS	2 (0.0)
PRODUCT LOT NUMBER ISSUE	1 (0.0)	HUNGER	1 (0.0)	LYMPH NODE PAIN	2 (0.0)	GASTROINTESTINAL BACTERIAL INFECTION	2 (0.0)
GASTROINTESTINAL VIRAL INFECTION	1 (0.0)	TOOTH ABSCESS	1 (0.0)	HALLUCINATION	2 (0.0)	LOW DENSITY LIPOPROTEIN ABNORMAL	2 (0.0)
CARPAL TUNNEL SYNDROME	1 (0.0)	SUBCUTANEOUS ABSCESS	1 (0.0)	GASTRIC INFECTION	2 (0.0)	FIBRIN D DIMER INCREASED	2 (0.0)
ALLERGY TO PLANTS	1 (0.0)	FRUSTRATION TOLERANCE DECREASED	1 (0.0)	HAEMOLYTIC ANAEMIA	2 (0.0)	OBESITY	2 (0.0)
RHEUMATOID NODULE	1 (0.0)	PULMONARY FIBROSIS	1 (0.0)	SENSATION OF FOREIGN BODY	2 (0.0)	GRANULOMA	2 (0.0)
ADENOCARCINOMA METASTATI	1 (0.0)	PRODUCT USE COMPLAINT	1 (0.0)	BONE MARROW DISORDER	2 (0.0)	CARDIOMYOPATHY	2 (0.0)

C							
ESCHERICHIA SEPSIS	1 (0.0)	LIP ULCERATION	1 (0.0)	EYE OPERATION	2 (0.0)	LUPUS-LIKE SYNDROME	2 (0.0)
PERIPHERAL NERVE INJURY	1 (0.0)	HYPOGAMMAGLOBULINAEMIA	1 (0.0)	INTRA-ABDOMINAL HAEMORRHAGE	2 (0.0)	VARICELLA	2 (0.0)
BLASTOMYCOSIS	1 (0.0)	PSEUDOMONAS INFECTION	1 (0.0)	PHARYNGEAL SWELLING	2 (0.0)	LIPIDS ABNORMAL	2 (0.0)
LARGE INTESTINE EROSION	1 (0.0)	TRAUMATIC LUNG INJURY	1 (0.0)	WEIGHT FLUCTUATION	2 (0.0)	LEUKOCYTOSIS	2 (0.0)
HEMIPARESIS	1 (0.0)	LARGE INTESTINE EROSION	1 (0.0)	IRON DEFICIENCY ANAEMIA	2 (0.0)	INFUSION	2 (0.0)
NYSTAGMUS	1 (0.0)	GASTROINTESTINAL MUCOSAL DISORDER	1 (0.0)	HEPATIC CYTOLYSIS	2 (0.0)	IMMUNOSUPPRESSANT DRUG THERAPY	2 (0.0)
CEREBRAL ARTERY STENOSIS	1 (0.0)	PSORIATIC ARTHROPATHY	1 (0.0)	HAEMANGIOMA OF BONE	2 (0.0)	ESSENTIAL HYPERTENSION	2 (0.0)
BRAIN NEOPLASM MALIGNANT	1 (0.0)	BLADDER DISORDER	1 (0.0)	ASPIRATION	2 (0.0)	VITAMIN B12 DEFICIENCY	2 (0.0)
SINUS DISORDER	1 (0.0)	SCAB	1 (0.0)	VENTRICULAR EXTRASYSTOLES	2 (0.0)	VITREOUS DETACHMENT	2 (0.0)
MUCOSAL HAEMORRHAGE	1 (0.0)	SEMEN DISCOLOURATION	1 (0.0)	TUBERCULOSIS	2 (0.0)	BLADDER HYPERTROPHY	2 (0.0)
ABDOMINAL WALL ABSCESS	1 (0.0)	CANDIDA ENDOPHTHALMITIS	1 (0.0)	ENDOSCOPY ABNORMAL	2 (0.0)	FEELING OF DESPAIR	2 (0.0)
NEUTROPHIL COUNT ABNORMAL	1 (0.0)	SITTING DISABILITY	1 (0.0)	GAMMA-GLUTAMYLTRANSFERASE INCREASED	2 (0.0)	ROTAVIRUS INFECTION	2 (0.0)
NASAL OEDEMA	1 (0.0)	EAR DISORDER	1 (0.0)	FEBRILE NEUTROPENIA	2 (0.0)	PLASMA CELL MYELOMA	2 (0.0)

NASAL DISORDER	1 (0.0)	SINUS HEADACHE	1 (0.0)	MICROCYTIC ANAEMIA	2 (0.0)	NEUTROPHILIA	2 (0.0)
LIP ULCERATION	1 (0.0)	PRECANCEROUS CELLS PRESENT	1 (0.0)	RENAL ATROPHY	1 (0.0)	EYE INJURY	2 (0.0)
HERPES SIMPLEX OESOPHAGITIS	1 (0.0)	BLOOD IMMUNOGLOBULIN G DECREASED	1 (0.0)	DECUBITUS ULCER	1 (0.0)	ABORTION	2 (0.0)
MUCOSAL INFLAMMATION	1 (0.0)	GENITAL BURNING SENSATION	1 (0.0)	BLADDER OPERATION	1 (0.0)	BODY TEMPERATURE ABNORMAL	2 (0.0)
ODYNOPHAGIA	1 (0.0)	PITUITARY TUMOUR BENIGN	1 (0.0)	PNEUMOCYSTIS JIROVECII INFECTION	1 (0.0)	GASTROINTESTINAL OEDEMA	2 (0.0)
BLOODY DISCHARGE	1 (0.0)	CARDIAC PACEMAKER INSERTION	1 (0.0)	BONE MARROW TRANSPLANT	1 (0.0)	DECREASED ACTIVITY	2 (0.0)
ENDODONTIC PROCEDURE	1 (0.0)	DIABETIC KETOACIDOSIS	1 (0.0)	SCAPULA FRACTURE	1 (0.0)	HOSPICE CARE	2 (0.0)
GINGIVAL BLEEDING	1 (0.0)	PELVIC INFECTION	1 (0.0)	DRAINAGE	1 (0.0)	RENAL INJURY	2 (0.0)
MUSCLE OPERATION	1 (0.0)	VEIN DISORDER	1 (0.0)	INGUINAL HERNIA	1 (0.0)	THROMBOCYTOPENIA	2 (0.0)
BARTHOLIN'S CYST	1 (0.0)	ALCOHOL ABUSE	1 (0.0)	ABNORMAL BEHAVIOUR	1 (0.0)	MUSCLE STRAIN	2 (0.0)
PLEURISY	1 (0.0)	IMMUNOGLOBULINS ABNORMAL	1 (0.0)	GRANDIOSITY	1 (0.0)	FOREIGN BODY IN THROAT	2 (0.0)
BLADDER PERFORATION	1 (0.0)	INFECTION SUSCEPTIBILITY INCREASED	1 (0.0)	DISORGANISED SPEECH	1 (0.0)	THROAT CLEARING	2 (0.0)
ATRIOVENTRICULAR DISSOCIATION	1 (0.0)	URINARY BLADDER ABSCESS	1 (0.0)	PERIPHERAL NERVE OPERATION	1 (0.0)	DERMATITIS ATOPIC	2 (0.0)
CHOLINERGIC	1 (0.0)	FREEZING PHENOMEN	1 (0.0)	THROMBOTIC STROKE	1 (0.0)	SYNOVIAL CYST	2 (0.0)

SYNDROME		ON					
VENTRICULAR TACHYCARDIA	1 (0.0)	THERAPEUTIC PRODUCT EFFECT DELAYED	1 (0.0)	SKIN MASS	1 (0.0)	LYME DISEASE	2 (0.0)
VENTRICULAR EXTRASYSTOLES	1 (0.0)	SLEEP DEFICIT	1 (0.0)	GASTROINTESTINAL SOUNDS ABNORMAL	1 (0.0)	CHOLESTATIC LIVER INJURY	2 (0.0)
EJECTION FRACTION DECREASED	1 (0.0)	COGNITIVE DISORDER	1 (0.0)	GALLBLADDER CANCER	1 (0.0)	EPSTEIN-BARR VIRUS INFECTION REACTIVATION	2 (0.0)
PERIPHERAL ISCHAEMIA	1 (0.0)	INCISION SITE IMPAIRED HEALING	1 (0.0)	APPENDIX CANCER	1 (0.0)	INGUINAL HERNIA	2 (0.0)
ASTHENOPIA	1 (0.0)	BONE GRAFT	1 (0.0)	PULMONARY TOXICITY	1 (0.0)	HEPATITIS	2 (0.0)
BLINDNESS UNILATERAL	1 (0.0)	ONYCHOMADESIS	1 (0.0)	BLOOD BLISTER	1 (0.0)	BONE PAIN	2 (0.0)
CYTOMEGALOVIRUS GASTROINTESTINAL INFECTION	1 (0.0)	ONYCHOCLASIS	1 (0.0)	POSTOPERATIVE THROMBOSIS	1 (0.0)	OCULAR ICTERUS	2 (0.0)
BRAIN STEM INFARCTION	1 (0.0)	LYMPH NODE TUBERCULOSIS	1 (0.0)	AXIAL SPONDYLOARTHRITIS	1 (0.0)	ANAL FISTULA	2 (0.0)
ARACHNOID CYST	1 (0.0)	RADIOTHERAPY	1 (0.0)	GINGIVAL DISORDER	1 (0.0)	COVID-19 PNEUMONIA	2 (0.0)
NEUROGENIC SHOCK	1 (0.0)	ANGIOEDEMA	1 (0.0)	WEIGHT GAIN POOR	1 (0.0)	TOOTH INFECTION	2 (0.0)
PNEUMONIA FUNGAL	1 (0.0)	DEMENTIA ALZHEIMER'S TYPE	1 (0.0)	WOUND ABSCESS	1 (0.0)	DEVICE PHYSICAL PROPERTY ISSUE	2 (0.0)
VASCULAR DEVICE INFECTION	1 (0.0)	PULMONARY VENOUS THROMBOSIS	1 (0.0)	OBSTRUCTIVE PANCREATITIS	1 (0.0)	PULMONARY SEPSIS	2 (0.0)
MAST CELL	1 (0.0)	LUMBAR	1 (0.0)	HYPERBILIR	1	PERONEAL	2 (0.0)

ACTIVATION SYNDROME		SPINAL STENOSIS		UBINAEMIA	(0.0)	NERVE PALSY	
LIMB DISCOMFORT	1 (0.0)	ANAL ABSCESS	1 (0.0)	LIPASE INCREASED	1 (0.0)	HERPES ZOSTER MENINGITIS	2 (0.0)
INTESTINAL ISCHAEMIA	1 (0.0)	MUSCULOSKELETAL CHEST PAIN	1 (0.0)	BRAIN OPERATION	1 (0.0)	DENTAL PROSTHESIS PLACEMENT	2 (0.0)
TUMOUR RUPTURE	1 (0.0)	PALLIATIVE CARE	1 (0.0)	BODY MASS INDEX ABNORMAL	1 (0.0)	HERPES ZOSTER OTICUS	2 (0.0)
TUMOUR HAEMORRHAGE	1 (0.0)	GASTROINTESTINAL ULCER PERFORATION	1 (0.0)	SKIN DISCOMFORT	1 (0.0)	MEDICAL DEVICE PAIN	2 (0.0)
ANORECTAL SWELLING	1 (0.0)	MOUTH SWELLING	1 (0.0)	PLANTAR FASCIITIS	1 (0.0)	CHRONIC MYELOID LEUKAEMIA	2 (0.0)
PRODUCT DOSE OMISSION IN ERROR	1 (0.0)	URINE FLOW DECREASED	1 (0.0)	MALIGNANT MELANOMA STAGE III	1 (0.0)	ACUTE RESPIRATORY FAILURE	2 (0.0)
ORAL HERPES ZOSTER	1 (0.0)	NECK SURGERY	1 (0.0)	NONINFECTIVE GINGIVITIS	1 (0.0)	HEPATITIS B REACTIVATION	2 (0.0)
LIMB OPERATION	1 (0.0)	RECTAL ABSCESS	1 (0.0)	THROMBOTIC THROMBOCYTOPENIC PURPURA	1 (0.0)	FOLLICULAR LYMPHOMA	2 (0.0)
ENTEROSTOMY CLOSURE	1 (0.0)	UTERINE MALPOSITION	1 (0.0)	INTERNATIONAL NORMALISED RATIO DECREASED	1 (0.0)	HEPATITIS B	2 (0.0)
CHEMOTHERAPY	1 (0.0)	UTERINE LEIOMYOMA	1 (0.0)	PROTHROMBIN TIME RATIO DECREASED	1 (0.0)	BLOOD LOSS ANAEMIA	2 (0.0)
HILAR LYMPHADENOPATHY	1 (0.0)	GASTROINTESTINAL ULCER	1 (0.0)	SKIN REACTION	1 (0.0)	DROWNING	2 (0.0)

GRANULOMA	1 (0.0)	MYOCARDIAL ISCHAEMIA	1 (0.0)	HEPATITIS B REACTIVATION	1 (0.0)	APPENDIX DISORDER	2 (0.0)
VERTEBRAL FORAMINAL STENOSIS	1 (0.0)	PRODUCT STORAGE ERROR	1 (0.0)	HYPERPLASTIC CHOLECYSTOPATHY	1 (0.0)	PNEUMONIA BACTERIAL	2 (0.0)
RED BLOOD CELL SEDIMENTATION RATE INCREASED	1 (0.0)	TROPONIN INCREASED	1 (0.0)	CELL DEATH	1 (0.0)	THYROID NEOPLASM	2 (0.0)
CSF VIRUS IDENTIFIED	1 (0.0)	KERATOACANTHOMA	1 (0.0)	SACRAL PAIN	1 (0.0)	LUNG OPACITY	2 (0.0)
COLITIS ISCHAEMIC	1 (0.0)	GENITAL HERPES	1 (0.0)	JOINT LOCK	1 (0.0)	CARDIAC ABLATION	2 (0.0)
ABDOMINAL MASS	1 (0.0)	DERMAL CYST	1 (0.0)	LYMPH NODE TUBERCULOSIS	1 (0.0)	SALMONELLOSIS	2 (0.0)
SKIN ODOUR ABNORMAL	1 (0.0)	LIVER ABSCESS	1 (0.0)	BENIGN NEOPLASM OF OPTIC NERVE	1 (0.0)	MEGACOLON	2 (0.0)
SKIN IRRITATION	1 (0.0)	THYROID CANCER	1 (0.0)	POST PROCEDURAL DISCHARGE	1 (0.0)	PURULENCE	2 (0.0)
ORBITAL DECOMPRESSION	1 (0.0)	ENDOMETRIOSIS	1 (0.0)	STOMA SITE DISCOMFORT	1 (0.0)	OESOPHAGEAL CARCINOMA	2 (0.0)
CERVICAL SPINAL STENOSIS	1 (0.0)	NON-HODGKIN'S LYMPHOMA	1 (0.0)	HEAT STROKE	1 (0.0)	ILEECTOMY	2 (0.0)
HYPOXIA	1 (0.0)	SKIN MASS	1 (0.0)	LIGAMENT DISORDER	1 (0.0)	ADENOCARCINOMA OF COLON	2 (0.0)
INFECTION PARASITIC	1 (0.0)	PHARYNGEAL OPERATION	1 (0.0)	HEPATIC CYST	1 (0.0)	LIVER ABSCESS	2 (0.0)
NASAL SEPTAL OPERATION	1 (0.0)	MESENTERIC ARTERIAL OCCLUSION	1 (0.0)	TENDON DISLOCATION	1 (0.0)	LOCALISED OEDEMA	2 (0.0)
TURBINOPL	1 (0.0)	POSTURAL	1 (0.0)	PARASITE	1	CARBON	2 (0.0)

ASTY		TREMOR		STOOL TEST POSITIVE	(0.0)	DIOXIDE DECREASED	
SPLENIC VEIN THROMBOSIS	1 (0.0)	DEMYELINATION	1 (0.0)	DIARRHOEA INFECTIOUS	1 (0.0)	MOUTH HAEMORRHAGE	2 (0.0)
TEMPERATURE INTOLERANCE	1 (0.0)	VITAMIN B12 DECREASED	1 (0.0)	SOMATIC SYMPTOM DISORDER	1 (0.0)	SLUGGISHNESS	2 (0.0)
EXPOSURE DURING PREGNANCY	1 (0.0)	THERMAL BURN	1 (0.0)	INTESTINAL STEATOSIS	1 (0.0)	CLAVICLE FRACTURE	2 (0.0)
CHRONIC GASTRITIS	1 (0.0)	GINGIVAL SWELLING	1 (0.0)	LACTOSE INTOLERANCE	1 (0.0)	GESTATIONAL DIABETES	1 (0.0)
SUSPECTED DRUG-INDUCED LIVER INJURY	1 (0.0)	STEATORRHOEA	1 (0.0)	POLYARTHRITIS	1 (0.0)	PERIORAL DERMATITIS	1 (0.0)
PRODUCT PRESCRIBING ERROR	1 (0.0)	VITREORETINAL TRACTION SYNDROME	1 (0.0)	MYOCARDIAL NECROSIS MARKER INCREASED	1 (0.0)	CSF WHITE BLOOD CELL COUNT DECREASED	1 (0.0)
HYPERTRIGLYCERIDAEMIA	1 (0.0)	INTESTINAL PSEUDO-OBSTRUCTION	1 (0.0)	VERTIGO POSITIONAL	1 (0.0)	INCREASED UPPER AIRWAY SECRETION	1 (0.0)
OVARIAN CYST RUPTURED	1 (0.0)	FUNCTIONAL GASTROINTESTINAL DISORDER	1 (0.0)	SEMEN ANALYSIS ABNORMAL	1 (0.0)	LIGAMENTITIS	1 (0.0)
HYPERAESTHESIA	1 (0.0)	MICROGRAPHIC SKIN SURGERY	1 (0.0)	SEMEN VISCOSITY ABNORMAL	1 (0.0)	VARICELLA MENINGITIS	1 (0.0)
ALLODYNIA	1 (0.0)	ABNORMAL WEIGHT GAIN	1 (0.0)	EXFOLIATIVE RASH	1 (0.0)	SPINAL CORD HAEMATOMA	1 (0.0)
FAECAL VOLUME DECREASED	1 (0.0)	THYROIDECTOMY	1 (0.0)	PLATELET DISORDER	1 (0.0)	PRODUCT CONTAMINATION	1 (0.0)

RED BLOOD CELL COUNT INCREASED	1 (0.0)	HEPATITIS TOXIC	1 (0.0)	BLOOD ALKALINE PHOSPHATASE ABNORMAL	1 (0.0)	WOUND DEHISCENCE	1 (0.0)
GENITAL ABSCESS	1 (0.0)	MULTIMORBIDITY	1 (0.0)	MESENTERIC VEIN THROMBOSIS	1 (0.0)	CONTRAINDICATION TO MEDICAL TREATMENT	1 (0.0)
HAND DERMATITIS	1 (0.0)	HIV INFECTION	1 (0.0)	HEPATIC ENZYME ABNORMAL	1 (0.0)	MALIGNANT NEOPLASM OF EYELID	1 (0.0)
MULTIPLE ALLERGIES	1 (0.0)	METABOLIC DISORDER	1 (0.0)	ONYCHOMYCOSIS	1 (0.0)	TENOPLASTY	1 (0.0)
APPENDICEAL ABSCESS	1 (0.0)	HEPATECTOMY	1 (0.0)	METABOLIC DISORDER	1 (0.0)	INTRA-ABDOMINAL FLUID COLLECTION	1 (0.0)
EOSINOPHILIA	1 (0.0)	GASTROINTESTINAL TRACT IRRITATION	1 (0.0)	FOREIGN BODY INGESTION	1 (0.0)	SALIVARY GLAND PAIN	1 (0.0)
BLOOD BILIRUBIN DECREASED	1 (0.0)	EYE CONTUSION	1 (0.0)	HEPATIC PAIN	1 (0.0)	PAROTID GLAND ENLARGEMENT	1 (0.0)
ESCHERICHIA TEST POSITIVE	1 (0.0)	MULTIPLE SCLEROSIS RELAPSE	1 (0.0)	GASTRIC PROLAPSE	1 (0.0)	THERAPEUTIC PRODUCT INEFFECTIVE	1 (0.0)
CSF WHITE BLOOD CELL COUNT POSITIVE	1 (0.0)	LICHENOID KERATOSIS	1 (0.0)	VASCULAR OCCLUSION	1 (0.0)	GOUT	1 (0.0)
TOOTH ABSCESS	1 (0.0)	GENITAL BLISTER	1 (0.0)	COAGULOPATHY	1 (0.0)	TONSILLOLITH	1 (0.0)
ISCHAEMIC SKIN ULCER	1 (0.0)	VULVOVAGINAL DISCOMFORT	1 (0.0)	LOSS OF CONTROL OF LEGS	1 (0.0)	ARTHROPOD BITE	1 (0.0)
ANAPHYLACTIC SHOCK	1 (0.0)	VULVOVAGINAL PAIN	1 (0.0)	AUTOIMMUNE HEPATITIS	1 (0.0)	HYPERTROPHIC CARDIOMYOPATHY	1 (0.0)

KELOID SCAR	1 (0.0)	VULVOVAGINAL ERYTHEMA	1 (0.0)	PERITONEAL ABSCESS	1 (0.0)	BREAST NEOPLASM	1 (0.0)
HYPOTHYROIDISM	1 (0.0)	VULVAL DISORDER	1 (0.0)	SKIN PLAQUE	1 (0.0)	DRAIN PLACEMENT	1 (0.0)
OROPHARYNGEAL DISCOMFORT	1 (0.0)	BARRETT'S OESOPHAGUS	1 (0.0)	INTENTIONAL PRODUCT USE ISSUE	1 (0.0)	PRECANCEROUS LESION OF DIGESTIVE TRACT	1 (0.0)
NONSPECIFIC REACTION	1 (0.0)	BEHCET'S SYNDROME	1 (0.0)	HYPERPHAGIA	1 (0.0)	GASTROINTESTINAL MUCOSA HYPERAEMIA	1 (0.0)
SKIN TEXTURE ABNORMAL	1 (0.0)	COMPUTERISED TOMOGRAM HEART ABNORMAL	1 (0.0)	GRIP STRENGTH DECREASED	1 (0.0)	LARGE INTESTINE EROSION	1 (0.0)
OLIGOMENORRHOEA	1 (0.0)	PURULENT DISCHARGE	1 (0.0)	LANGERHANS' CELL HISTIOCYTOSIS	1 (0.0)	ORGAN FAILURE	1 (0.0)
DEMYELINATION	1 (0.0)	CARDIAC DYSFUNCTION	1 (0.0)	MUSCLE OPERATION	1 (0.0)	NASAL SEPTAL OPERATION	1 (0.0)
LACUNAR INFARCTION	1 (0.0)	METASTASES TO LYMPH NODES	1 (0.0)	ADENOCARCINOMA METASTATIC	1 (0.0)	ERUCTATION	1 (0.0)
ADVERSE EVENT	1 (0.0)	BILE DUCT STENOSIS	1 (0.0)	ASCITES	1 (0.0)	NEPHRITIS	1 (0.0)
LAPAROTOMY	1 (0.0)	BACTERIAL INFECTION INDEX INCREASED	1 (0.0)	CEREBRAL ISCHAEMIA	1 (0.0)	TINEA INFECTION	1 (0.0)
RESPIRATORY TRACT INFECTION VIRAL	1 (0.0)	HEPATIC FUNCTION ABNORMAL	1 (0.0)	METAL POISONING	1 (0.0)	LYMPHATIC SYSTEM NEOPLASM	1 (0.0)
GASTROOESOPHAGEAL SPHINCTER INSUFFICIE	1 (0.0)	INTRACARDIAC THROMBUS	1 (0.0)	ANAL INFLAMMATION	1 (0.0)	PERIPORTAL OEDEMA	1 (0.0)

NCY							
VIRAEMIA	1 (0.0)	RENAL HAEMORRHAGE	1 (0.0)	BRONCHOSPASM	1 (0.0)	ARTERIOSCLEROSIS	1 (0.0)
GASTROINTESTINAL OEDEMA	1 (0.0)	THERAPEUTIC PROCEDURE	1 (0.0)	LUMBAR PUNCTURE ABNORMAL	1 (0.0)	PURPURA	1 (0.0)
BEDRIDDEN	1 (0.0)	RECTAL POLYP	1 (0.0)	CHRONIC SINUSITIS	1 (0.0)	RECTAL CANCER STAGE IV	1 (0.0)
LIPASE INCREASED	1 (0.0)	PULMONARY TUBERCULOSIS	1 (0.0)	VITH NERVE PARALYSIS	1 (0.0)	MYELIN OLIGODENDROCYTE GLYCOPROTEIN ANTIBODY-ASSOCIATED DISEASE	1 (0.0)
RECTAL STENOSIS	1 (0.0)	MIDDLE CEREBRAL ARTERY STROKE	1 (0.0)	INCISION SITE DISCHARGE	1 (0.0)	VENTRICULAR TACHYCARDIA	1 (0.0)
NECROTISING MYOSITIS	1 (0.0)	LEFT ATRIAL ENLARGEMENT	1 (0.0)	BLOOD GROWTH HORMONE DECREASED	1 (0.0)	CUTANEOUS T-CELL LYMPHOMA	1 (0.0)
SQUAMOUS CELL CARCINOMA	1 (0.0)	ANASTOMOTIC LEAK	1 (0.0)	HAEMATOCRIT ABNORMAL	1 (0.0)	HAEMATOLOGICAL MALIGNANCY	1 (0.0)
EXCESSIVE GRANULATION TISSUE	1 (0.0)	TOE OPERATION	1 (0.0)	CEREBRAL ARTERY STENOSIS	1 (0.0)	DISEASE COMPLICATION	1 (0.0)
POST THROMBOTIC SYNDROME	1 (0.0)	METABOLIC ACIDOSIS	1 (0.0)	HEMIPARESIS	1 (0.0)	LIP HAEMORRHAGE	1 (0.0)
PETIT MAL EPILEPSY	1 (0.0)	FOLLICULAR LYMPHOMA	1 (0.0)	ARACHNOID CYST	1 (0.0)	OESOPHAGEAL ULCER HAEMORRHAGE	1 (0.0)
CHROMATOPSIA	1 (0.0)	WHITE BLOOD	1 (0.0)	CATHETER REMOVAL	1 (0.0)	HEPATIC NEOPLASM	1 (0.0)

		CELL DISORDER					
THALAMIC INFARCTION	1 (0.0)	GASTRIC DISORDER	1 (0.0)	CATHETER SITE THROMBOSIS	1 (0.0)	CIRCULATORY COLLAPSE	1 (0.0)
ORCHITIS	1 (0.0)	END STAGE RENAL DISEASE	1 (0.0)	FLUID REPLACEMENT	1 (0.0)	PROSTATE CANCER STAGE IV	1 (0.0)
BOWEN'S DISEASE	1 (0.0)	ANASTOMOTIC COMPLICATION	1 (0.0)	PHARYNGITIS STREPTOCOCCAL	1 (0.0)	LUNG CARCINOMA CELL TYPE UNSPECIFIED STAGE IV	1 (0.0)
EYE OEDEMA	1 (0.0)	ENDOSCOPY SMALL INTESTINE ABNORMAL	1 (0.0)	SCRATCH	1 (0.0)	VAGINAL HAEMORRHAGE	1 (0.0)
PREMENSTRUAL SYNDROME	1 (0.0)	PROSTATIC SPECIFIC ANTIGEN INCREASED	1 (0.0)	HYPOALBUMINAEMIA	1 (0.0)	BLOOD TESTOSTERONE DECREASED	1 (0.0)
NAIL BED TENDERNESS	1 (0.0)	HAEMORRHOIDAL HAEMORRHAGE	1 (0.0)	EYE DISCHARGE	1 (0.0)	ABDOMINAL MASS	1 (0.0)
CARDIOMYOPATHY ACUTE	1 (0.0)	DYSGEUSIA	1 (0.0)	CATHETER PLACEMENT	1 (0.0)	DIVERTICULAR PERFORATION	1 (0.0)
ACNE VARIOLIFORMIS	1 (0.0)	AORTIC THROMBOSIS	1 (0.0)	BONE FISSURE	1 (0.0)	SACROILIITIS	1 (0.0)
AGRANULOCYTOSIS	1 (0.0)	GENITAL HERPES ZOSTER	1 (0.0)	ANTIPHOSPHOLIPID SYNDROME	1 (0.0)	OROPHARYNGEAL NEOPLASM BENIGN	1 (0.0)
BLOOD UREA INCREASED	1 (0.0)	EYE IRRITATION	1 (0.0)	IMMUNE SYSTEM DISORDER	1 (0.0)	FLUCTUANCE	1 (0.0)
AMYLASE INCREASED	1 (0.0)	POSTOPERATIVE ILEUS	1 (0.0)	LIVEDO RETICULARIS	1 (0.0)	CANCER IN REMISSION	1 (0.0)
PNEUMONIA	1 (0.0)	CARDIAC	1 (0.0)	GASTROENT	1	BRAIN	1 (0.0)

VIRAL		STRESS TEST ABNORMAL		EROSTOMY	(0.0)	INJURY	
HYPERVOLAEMIA	1 (0.0)	CARDIAC DEVICE IMPLANTATION	1 (0.0)	SARCOIDOSIS	1 (0.0)	DEVICE FASTENER ISSUE	1 (0.0)
MYOCARDITIS BACTERIAL	1 (0.0)	OESOPHAGEAL OBSTRUCTION	1 (0.0)	SPINAL CORD COMPRESSION	1 (0.0)	DEVICE LOOSENING	1 (0.0)
INFECTIOUS PLEURAL EFFUSION	1 (0.0)	OSTEOMALACIA	1 (0.0)	INCORRECT DOSAGE ADMINISTERED	1 (0.0)	DENTAL PROSTHESIS REMOVAL	1 (0.0)
OPPORTUNISTIC INFECTION	1 (0.0)	HEPATITIS	1 (0.0)	ORGAN TRANSPLANT	1 (0.0)	SKIN INDURATION	1 (0.0)
DEFAECATION DISORDER	1 (0.0)	PELVIC VENOUS THROMBOSIS	1 (0.0)	OROPHARYNGEAL BLISTERING	1 (0.0)	FLUID REPLACEMENT	1 (0.0)
CARTILAGE INJURY	1 (0.0)	MONOPARESIS	1 (0.0)	CERVIX WARTS	1 (0.0)	ANAPHYLACTIC SHOCK	1 (0.0)
GASTROINTESTINAL EROSION	1 (0.0)	OESOPHAGEAL ULCER HAEMORRHAGE	1 (0.0)	CORONARY ARTERY STENOSIS	1 (0.0)	ALTERED STATE OF CONSCIOUSNESS	1 (0.0)
RETINAL TEAR	1 (0.0)	ANORECTAL CELLULITIS	1 (0.0)	CORONARY ARTERY DISEASE	1 (0.0)	ACUTE HEPATITIS B	1 (0.0)
HEPATITIS TOXIC	1 (0.0)	RECTAL FISSURE	1 (0.0)	DRUG ABUSE	1 (0.0)	NERVOUS SYSTEM DISORDER	1 (0.0)
PROTEIN URINE PRESENT	1 (0.0)	RESPIRATORY DISTRESS	1 (0.0)	VOCAL CORD DISORDER	1 (0.0)	COMPLEX REGIONAL PAIN SYNDROME	1 (0.0)
MEAN PLATELET VOLUME DECREASED	1 (0.0)	PNEUMONIA STREPTOCOCCAL	1 (0.0)	PALLIATIVE CARE	1 (0.0)	SINUS TACHYCARDIA	1 (0.0)
URINE FLOW	1 (0.0)	HOSPICE CARE	1 (0.0)	BEDRIDDEN	1 (0.0)	INFILTRATIO	1 (0.0)

DECREASED						N	
BLADDER PAIN	1 (0.0)	VENOUS THROMBOSIS LIMB	1 (0.0)	CORONAVIRUS INFECTION	1 (0.0)	GASTROINTESTINAL SOUNDS ABNORMAL	1 (0.0)
CONCOMITANT DISEASE AGGRAVATED	1 (0.0)	IMPAIRED GASTRIC EMPTYING	1 (0.0)	MENINGITIS ASEPTIC	1 (0.0)	TYMPANIC MEMBRANE PERFORATION	1 (0.0)
LIVER TRANSPLANT	1 (0.0)	SOMNAMBULISM	1 (0.0)	TONSILLITIS	1 (0.0)	HEPATIC CYST INFECTION	1 (0.0)
GALLBLADDER OBSTRUCTION	1 (0.0)	COVID-19 PNEUMONIA	1 (0.0)	ENDODONTIC PROCEDURE	1 (0.0)	ARTERIAL STENOSIS	1 (0.0)
VESICAL FISTULA	1 (0.0)	AFFECTIVE DISORDER	1 (0.0)	TANDEM GAIT TEST ABNORMAL	1 (0.0)	INTESTINAL STENOSIS	1 (0.0)
EFFUSION	1 (0.0)	HEPATITIS B REACTIVATION	1 (0.0)	MONTREAL COGNITIVE ASSESSMENT ABNORMAL	1 (0.0)	AUTOIMMUNE THYROIDITIS	1 (0.0)
FISTULA OF SMALL INTESTINE	1 (0.0)	HEPATORENAL SYNDROME	1 (0.0)	DYSARTHRIA	1 (0.0)	VIRAL HEPATITIS CARRIER	1 (0.0)
SERRATED POLYPOSIS SYNDROME	1 (0.0)	URINE PROTEIN/CREATININE RATIO INCREASED	1 (0.0)	ATAXIA	1 (0.0)	ABORTION MISSED	1 (0.0)
DIVERSION COLITIS	1 (0.0)	BLOOD CHLORIDE INCREASED	1 (0.0)	SPLINTER HAEMORRHAGES	1 (0.0)	PLAGUE	1 (0.0)
FACIAL PARALYSIS	1 (0.0)	MONOCLONAL GAMMOPATHY	1 (0.0)	ADENOVIRUS INFECTION	1 (0.0)	FOCAL PERITONITIS	1 (0.0)
GASTROINTESTINAL CARCINOMA	1 (0.0)	CRYPTITIS	1 (0.0)	UTERINE LEIOMYOMA	1 (0.0)	PRODUCT DOSE OMISSION ISSUE	1 (0.0)

CEREBELLAR INFARCTION	1 (0.0)	CLOSTRIDIAL INFECTION	1 (0.0)	CYTOGENETIC ABNORMALITY	1 (0.0)	INSURANCE ISSUE	1 (0.0)
NAIL BED BLEEDING	1 (0.0)	INTESTINAL VASCULAR DISORDER	1 (0.0)	ARTICULAR CALCIFICATION	1 (0.0)	MENINGIOMA BENIGN	1 (0.0)
LOSS OF CONTROL OF LEGS	1 (0.0)	ECHOCARDIOGRAM	1 (0.0)	SERUM FERRITIN INCREASED	1 (0.0)	METABOLIC ACIDOSIS	1 (0.0)
BELL'S PALSY	1 (0.0)	TICK-BORNE FEVER	1 (0.0)	TRANSAMINASES INCREASED	1 (0.0)	PSYCHOTIC DISORDER	1 (0.0)
CEREBRAL ISCHAEMIA	1 (0.0)	INTRA-ABDOMINAL HAEMORRHAGE	1 (0.0)	RHEGMATOGENOUS RETINAL DETACHMENT	1 (0.0)	STRABISMUS	1 (0.0)
ONYCHOLYSIS	1 (0.0)	MACULAR DEGENERATION	1 (0.0)	HYPERTRIGLYCERIDAEMIA	1 (0.0)	AFFECTIVE DISORDER	1 (0.0)
ALBUMIN GLOBULIN RATIO DECREASED	1 (0.0)	ACCIDENTAL EXPOSURE TO PRODUCT	1 (0.0)	ORCHITIS NONINFECTIVE	1 (0.0)	SOMNAMBULISM	1 (0.0)
LYMPHOCYTE PERCENTAGE DECREASED	1 (0.0)	HEART RATE IRREGULAR	1 (0.0)	JOINT EFFUSION	1 (0.0)	PERIORBITAL PAIN	1 (0.0)
BLOOD MAGNESIUM DECREASED	1 (0.0)	CORONARY ARTERY EMBOLISM	1 (0.0)	HAEMOTHORAX	1 (0.0)	KERATITIS	1 (0.0)
NEUTROPHIL PERCENTAGE INCREASED	1 (0.0)	GRANULOMA	1 (0.0)	RECTAL PERFORATION	1 (0.0)	UMBILICAL HERNIA PERFORATION	1 (0.0)
BLOOD OSMOLARITY DECREASED	1 (0.0)	VAGINAL POLYP	1 (0.0)	ISCHAEMIC SKIN ULCER	1 (0.0)	EXTREMITY NECROSIS	1 (0.0)
PROTHROM	1 (0.0)	DISSEMINAT	1 (0.0)	EOSINOPHIL	1	CENTRAL	1 (0.0)

BIN TIME PROLONGED		ED TUBERCULOSIS		COUNT DECREASED	(0.0)	VENOUS CATHETER REMOVAL	
GLOBULINS INCREASED	1 (0.0)	APHASIA	1 (0.0)	TRANSFERRIN SATURATION DECREASED	1 (0.0)	FUSOBACTERIUM INFECTION	1 (0.0)
MEAN CELL VOLUME DECREASED	1 (0.0)	BOWEL MOVEMENT IRREGULARITY	1 (0.0)	HEPATOCELLULAR CARCINOMA	1 (0.0)	ENTERITIS	1 (0.0)
MEAN CELL HAEMOGLOBIN CONCENTRATION DECREASED	1 (0.0)	SPEECH DISORDER DEVELOPMENTAL	1 (0.0)	JUGULAR VEIN OCCLUSION	1 (0.0)	BENIGN SMALL INTESTINAL NEOPLASM	1 (0.0)
BLOOD UREA NITROGEN/ CREATININE RATIO DECREASED	1 (0.0)	LIGHT CHAIN ANALYSIS INCREASED	1 (0.0)	EYELID CYST	1 (0.0)	AUTOIMMUNE HEPATITIS	1 (0.0)
BLOOD ALBUMIN DECREASED	1 (0.0)	MEAN CELL HAEMOGLOBIN CONCENTRATION DECREASED	1 (0.0)	RED BLOOD CELL SEDIMENTATION RATE INCREASED	1 (0.0)	CRANIOTOMY	1 (0.0)
MEAN CELL HAEMOGLOBIN DECREASED	1 (0.0)	PSEUDOPOLYP	1 (0.0)	RENAL SURGERY	1 (0.0)	CEREBRAL VENTRICLE DILATATION	1 (0.0)
CORONARY ARTERY OCCLUSION	1 (0.0)	NOCARDIOSIS	1 (0.0)	JOINT SURGERY	1 (0.0)	RETINAL DRUSEN	1 (0.0)
URINE KETONE BODY PRESENT	1 (0.0)	SUPERINFECTION	1 (0.0)	SPLEEN DISORDER	1 (0.0)	TYPE IIA HYPERLIPIDAEMIA	1 (0.0)
PROTEIN TOTAL INCREASED	1 (0.0)	PNEUMONIA CYTOMEGALOVIRAL	1 (0.0)	RED BLOOD CELL TRANSFUSION	1 (0.0)	DIVERTICULUM INTESTINAL	1 (0.0)

BLOOD CHLORIDE DECREASED	1 (0.0)	OCULAR MELANOMA	1 (0.0)	EXTREMITY NECROSIS	1 (0.0)	VASECTOMY	1 (0.0)
IMMATURE GRANULOCYTE PERCENTAGE INCREASED	1 (0.0)	ALCOHOLIC LIVER DISEASE	1 (0.0)	FLAP SURGERY	1 (0.0)	COCHLEA IMPLANT	1 (0.0)
WRIST FRACTURE	1 (0.0)	HEPATIC FAILURE	1 (0.0)	FAECAL VOLUME INCREASED	1 (0.0)	SINUS OPERATION	1 (0.0)
NEPHRITIS	1 (0.0)	SPONTANEOUS BACTERIAL PERITONITIS	1 (0.0)	PERINEAL CYST	1 (0.0)	APPENDICITIS NONINFECTIVE	1 (0.0)
RENAL CANCER	1 (0.0)	CIRRHOSIS ALCOHOLIC	1 (0.0)	PERIORBITAL INFLAMMATION	1 (0.0)	GASTROENTERITIS SALMONELLA	1 (0.0)
SHOCK HAEMORRHAGIC	1 (0.0)	TRANSFERRIN SATURATION INCREASED	1 (0.0)	INFUSION SITE INJURY	1 (0.0)	MICROGRAPHIC SKIN SURGERY	1 (0.0)
GASTROINTESTINAL MUCOSAL EXFOLIATION	1 (0.0)	ACTIVATED PARTIAL THROMBOPLASTIN TIME PROLONGED	1 (0.0)	VITREOUS DETACHMENT	1 (0.0)	SKIN PLAQUE	1 (0.0)
UPPER-AIRWAY COUGH SYNDROME	1 (0.0)	IRON BINDING CAPACITY TOTAL INCREASED	1 (0.0)	CYTOMEGALOVIRUS GASTROINTESTINAL INFECTION	1 (0.0)	CORNEAL OPACITY	1 (0.0)
DISABILITY	1 (0.0)	VITAMIN B12 INCREASED	1 (0.0)	UTERINE ENLARGEMENT	1 (0.0)	HERPES OPHTHALMIC	1 (0.0)
HYDRONEPHROSIS	1 (0.0)	IRON BINDING CAPACITY UNSATURATED	1 (0.0)	CLAVICLE FRACTURE	1 (0.0)	GASTROINTESTINAL ADENOCARCINOMA	1 (0.0)

PAROTITIS	1 (0.0)	INCREASED SKIN FRAGILITY	1 (0.0)	FISTULOTOMY	1 (0.0)	NEOPLASM SKIN	1 (0.0)
RECTAL PERFORATION	1 (0.0)	GASTROINTESTINAL SOUNDS ABNORMAL	1 (0.0)	ACOUSTIC NEUROMA	1 (0.0)	PROSTATIC DISORDER	1 (0.0)
ERYTHEMA MULTIFORME	1 (0.0)	PERIORBITAL PAIN	1 (0.0)	ABDOMINAL SEPSIS	1 (0.0)	PROTEIN TOTAL ABNORMAL	1 (0.0)
PSOAS ABSCESS	1 (0.0)	EYE OEDEMA	1 (0.0)	PSYCHOMOTOR RETARDATION	1 (0.0)	HETEROPHORIA	1 (0.0)
RESTRICTIVE PULMONARY DISEASE	1 (0.0)	SKIN SQUAMOUS CELL CARCINOMA RECURRENT	1 (0.0)	MICROGRAPHIC SKIN SURGERY	1 (0.0)	CATARACT NUCLEAR	1 (0.0)
RHINITIS	1 (0.0)	UNDERDOSE	1 (0.0)	MULTIPLE FRACTURES	1 (0.0)	RETINOPATHY HYPERTENSIVE	1 (0.0)
INTESTINAL MUCOSAL HYPERTROPHY	1 (0.0)	MEAN PLATELET VOLUME DECREASED	1 (0.0)	PLAGUE	1 (0.0)	COLLOID BRAIN CYST	1 (0.0)
PNEUMOMEDIASTINUM	1 (0.0)	MONOCYTE PERCENTAGE INCREASED	1 (0.0)	BLOOD PHOSPHORUS DECREASED	1 (0.0)	PANCREATIC FAILURE	1 (0.0)
BACTERIAL FOOD POISONING	1 (0.0)	EXPOSURE TO COMMUNICABLE DISEASE	1 (0.0)	BURKITT'S LYMPHOMA	1 (0.0)	PANCREATIC ENZYMES DECREASED	1 (0.0)
MUCOSAL DISORDER	1 (0.0)	CUSHING'S SYNDROME	1 (0.0)	ONYCHOCLASIS	1 (0.0)	DEVICE RELATED SEPSIS	1 (0.0)
GASTROINTESTINAL NECROSIS	1 (0.0)	CEREBROSPINAL FLUID RETENTION	1 (0.0)	PNEUMONIA NECROTISING	1 (0.0)	DEMENTIA ALZHEIMER'S TYPE	1 (0.0)
OSTEOTOMY	1 (0.0)	PUSTULAR PSORIASIS	1 (0.0)	JAW FRACTURE	1 (0.0)	LIGAMENT INJURY	1 (0.0)

RENAL CELL CARCINOMA	1 (0.0)	CARDIAC ABLATION	1 (0.0)	CHOLECYSTITIS INFECTIVE	1 (0.0)	BLOOD ELASTASE DECREASED	1 (0.0)
VULVOVAGINAL DRYNESS	1 (0.0)	PSOAS ABSCESS	1 (0.0)	GRANULOCYTE COUNT DECREASED	1 (0.0)	PHARYNGEAL HAEMORRHAGE	1 (0.0)
STOMA OBSTRUCTION	1 (0.0)	LIP SWELLING	1 (0.0)	HAEMORRHOIDS THROMBOSED	1 (0.0)	PERIORBITAL SWELLING	1 (0.0)
INTESTINAL MUCOSAL TEAR	1 (0.0)	OESOPHAGEAL OPERATION	1 (0.0)	OVARIAN VEIN THROMBOSIS	1 (0.0)	NASAL DRYNESS	1 (0.0)
RENAL NEOPLASM	1 (0.0)	ARTHRITIS BACTERIAL	1 (0.0)	OSTEOMYELITIS BACTERIAL	1 (0.0)	GASTRIC ULCER	1 (0.0)
ENTEROSTOMY	1 (0.0)	NASAL SEPTAL OPERATION	1 (0.0)	POLLAKIURIA	1 (0.0)	ELECTROCARDIOGRAM ST-T SEGMENT ABNORMAL	1 (0.0)
GRIP STRENGTH DECREASED	1 (0.0)	ANAEMIA OF CHRONIC DISEASE	1 (0.0)	EMBOLIC STROKE	1 (0.0)	MUSCLE OPERATION	1 (0.0)
ATRIAL THROMBOSIS	1 (0.0)	HEPATIC VASCULAR THROMBOSIS	1 (0.0)	VENOUS OPERATION	1 (0.0)	HILAR LYMPHADENOPATHY	1 (0.0)
FOOT FRACTURE	1 (0.0)	INTRA-ABDOMINAL FLUID COLLECTION	1 (0.0)	HEART RATE DECREASED	1 (0.0)	RETINITIS	1 (0.0)
CHOKING SENSATION	1 (0.0)	FACTOR V LEIDEN MUTATION	1 (0.0)	UTERINE TENDERNESS	1 (0.0)	RENAL INFARCT	1 (0.0)
EAR CONGESTION	1 (0.0)	PSEUDOPOLYPOSIS	1 (0.0)	BRAIN STEM INFARCTION	1 (0.0)	INTRACARDIAC THROMBUS	1 (0.0)
SUNBURN	1 (0.0)	ENTEROVESICAL FISTULA	1 (0.0)	BLOOD ELECTROLYTES ABNORMAL	1 (0.0)	DEVICE LEAKAGE	1 (0.0)

CORONAVIRUS INFECTION	1 (0.0)	CLOSTRIDIUM TEST POSITIVE	1 (0.0)	VANCOMYCIN INFUSION REACTION	1 (0.0)	DISLOCATION OF VERTEBRA	1 (0.0)
ERYSIPELAS	1 (0.0)	ORAL MUCOSAL DISCOLOURATION	1 (0.0)	CELLULITIS STAPHYLOCOCCAL	1 (0.0)	INTESTINAL PSEUDO-OBSTRUCTION	1 (0.0)
JOINT EFFUSION	1 (0.0)	PRECANCEROUS CONDITION	1 (0.0)	HYPERCOAGULATION	1 (0.0)	URINARY TRACT DISORDER	1 (0.0)
CHOLESTATIC LIVER INJURY	1 (0.0)	STREPTOCOCCAL INFECTION	1 (0.0)	PNEUMONIA CRYPTOCOCCAL	1 (0.0)	VITREORETINAL TRACTION SYNDROME	1 (0.0)
OROPHARYNGEAL SQUAMOUS CELL CARCINOMA	1 (0.0)	INTERVERTEBRAL DISCITIS	1 (0.0)	ENTEROSTOMY	1 (0.0)	STEATORRHOEA	1 (0.0)
PULMONARY CALCIFICATION	1 (0.0)	DROWNING	1 (0.0)	COCCIDIOIDOMYCOSIS	1 (0.0)	GINGIVAL SWELLING	1 (0.0)
ACUTE MYOCARDIAL INFARCTION	1 (0.0)	PERICARDIAL EFFUSION	1 (0.0)	BRAIN STEM STROKE	1 (0.0)	CHRONIC LYMPHOCYTIC LEUKAEMIA	1 (0.0)
GLIONEURONAL TUMOUR	1 (0.0)	ACUTE HEPATITIS B	1 (0.0)	THERAPEUTIC PROCEDURE	1 (0.0)	SQUAMOUS CELL CARCINOMA OF LUNG	1 (0.0)
RECTAL PROLAPSE REPAIR	1 (0.0)	ALTERED STATE OF CONSCIOUSNESS	1 (0.0)	ANAEMIA OF CHRONIC DISEASE	1 (0.0)	HAEMANGIOMA OF SKIN	1 (0.0)
BLADDER REPAIR	1 (0.0)	NEPHRITIS	1 (0.0)	DRUG INEFFECTIVE FOR UNAPPROVED INDICATION	1 (0.0)	HAIR DISORDER	1 (0.0)
STREPTOCOCCAL	1 (0.0)	TYPE IIA HYPERLIPID	1 (0.0)	INTRADUCTAL	1 (0.0)	HAEMATOLOGICAL	1 (0.0)

INFECTION		AEMIA		PROLIFERATIVE BREAST LESION		NEOPLASM	
MILIARIA	1 (0.0)	RETINAL DRUSEN	1 (0.0)	DYSPNOEA EXERTIONAL	1 (0.0)	SELECTIVE ABORTION	1 (0.0)
VIRAL RASH	1 (0.0)	IMMUNOSUPPRESSANT DRUG THERAPY	1 (0.0)	SELF-INJURIOUS IDEATION	1 (0.0)	EATING DISORDER	1 (0.0)
INFREQUENT BOWEL MOVEMENTS	1 (0.0)	INDURATION	1 (0.0)	AGRANULOCYTOSIS	1 (0.0)	INGROWN HAIR	1 (0.0)
URINE ANALYSIS ABNORMAL	1 (0.0)	PRECANCEROUS LESION OF DIGESTIVE TRACT	1 (0.0)	PULSELESS ELECTRICAL ACTIVITY	1 (0.0)	BLOOD ELECTROLYTES DECREASED	1 (0.0)
HYPERLIPIDAEMIA	1 (0.0)	RECTAL TENESMUS	1 (0.0)	VARICELLA	1 (0.0)	GASTROINTESTINAL OBSTRUCTION	1 (0.0)
PSEUDOSTROKE	1 (0.0)	PRODUCT PRESCRIBING ERROR	1 (0.0)	VARICELLA ZOSTER OESOPHAGITIS	1 (0.0)	SPUTUM INCREASED	1 (0.0)
FISTULA REPAIR	1 (0.0)	ASPHYXIA	1 (0.0)	COMPRESSION FRACTURE	1 (0.0)	INCORRECT ROUTE OF PRODUCT ADMINISTRATION	1 (0.0)
PULMONARY PAIN	1 (0.0)	VAGINAL ULCERATION	1 (0.0)	HAIR GROWTH ABNORMAL	1 (0.0)	INFUSION SITE REACTION	1 (0.0)
LEUKOCYTOSIS	1 (0.0)	COLLOID BRAIN CYST	1 (0.0)	COLON INJURY	1 (0.0)	BLOOD BILIRUBIN ABNORMAL	1 (0.0)
REMISSION NOT ACHIEVED	1 (0.0)	RETINOPATHY HYPERTENSIVE	1 (0.0)	CHANGE OF BOWEL HABIT	1 (0.0)	INFUSION SITE SWELLING	1 (0.0)
ISCHAEMIA	1 (0.0)	PROTEIN TOTAL INCREASED	1 (0.0)	SPINAL ANAESTHESIA	1 (0.0)	FREEZING PHENOMENON	1 (0.0)

PELVIC FLUID COLLECTION	1 (0.0)	CLOSTRIDIUM DIFFICILE COLITIS	1 (0.0)	PNEUMATOSIS	1 (0.0)	URINARY BLADDER ABSCESS	1 (0.0)
OESOPHAGEAL ULCER	1 (0.0)	DEAFNESS BILATERAL	1 (0.0)	CHALAZION	1 (0.0)	IMMUNOGLOBULINS ABNORMAL	1 (0.0)
SINUS CONGESTION	1 (0.0)	HYPOXIC-ISCHAEMIC ENCEPHALOPATHY	1 (0.0)	HAEMORRHAGE INTRACRANIAL	1 (0.0)	NAIL DISCOLOURATION	1 (0.0)
UTERINE SPASM	1 (0.0)	LUNG ABSCESS	1 (0.0)	PARANASAL SINUS HYPERSECRETION	1 (0.0)	BONE CANCER	1 (0.0)
BLOOD CALCIUM DECREASED	1 (0.0)	PHARYNGEAL ULCERATION	1 (0.0)	MUSCLE RUPTURE	1 (0.0)	ALCOHOL ABUSE	1 (0.0)
CANDIDA INFECTION	1 (0.0)	HERPANGINA	1 (0.0)	MUSCLE STRAIN	1 (0.0)	BRONCHIAL DISORDER	1 (0.0)
ANASTOMOTIC STENOSIS	1 (0.0)	URINARY HESITATION	1 (0.0)	BIPOLAR DISORDER	1 (0.0)	RESPIRATION ABNORMAL	1 (0.0)
MACULAR OEDEMA	1 (0.0)	TOXICITY TO VARIOUS AGENTS	1 (0.0)	BARTHOLIN'S CYST	1 (0.0)	POST PROCEDURAL CONTUSION	1 (0.0)
INTESTINAL ULCER PERFORATION	1 (0.0)	PSEUDOPORPHYRIA	1 (0.0)	MYOCARDITIS BACTERIAL	1 (0.0)	ATROPHY	1 (0.0)
LYMPH NODE PAIN	1 (0.0)	ARTERIAL HAEMORRHAGE	1 (0.0)	VASCULAR DEVICE OCCLUSION	1 (0.0)	DRY EYE	1 (0.0)
MEDICAL DEVICE SITE INFLAMMATION	1 (0.0)	ULCER	1 (0.0)	SUBCLAVIAN VEIN THROMBOSIS	1 (0.0)	FUNCTIONAL GASTROINTESTINAL DISORDER	1 (0.0)
MEDICAL DEVICE SITE RASH	1 (0.0)	UPPER GASTROINTESTINAL HAEMORRH	1 (0.0)	SUBCLAVIAN VEIN OCCLUSION	1 (0.0)	MEDICAL DEVICE SITE JOINT INFECTION	1 (0.0)

		AGE					
MEDICAL DEVICE SITE ERYTHEMA	1 (0.0)	BREAST PAIN	1 (0.0)	PANCREATECTOMY	1 (0.0)	URETERAL STENT INSERTION	1 (0.0)
HYPERCALCIURIA	1 (0.0)	FACIAL ASYMMETRY	1 (0.0)	PNEUMONIA VIRAL	1 (0.0)	ENTERITIS INFECTIOUS	1 (0.0)
NEPHROCALCINOSIS	1 (0.0)	HEMIPLEGIA	1 (0.0)	COLITIS ISCHAEMIC	1 (0.0)	MORTON'S NEURALGIA	1 (0.0)
GASTROINTESTINAL CANCER METASTATIC	1 (0.0)	SQUAMOUS CELL CARCINOMA OF THE TONGUE	1 (0.0)	THALAMIC INFARCTION	1 (0.0)	POST-ACUTE COVID-19 SYNDROME	1 (0.0)
BREAST ABSCESS	1 (0.0)	RENAL STONE REMOVAL	1 (0.0)	BONE DENSITY DECREASED	1 (0.0)	HYPERKALAEMIA	1 (0.0)
PNEUMONIA BORDETELLA	1 (0.0)	PLEURISY	1 (0.0)	SKIN FRAGILITY	1 (0.0)	DUODENAL ULCER HAEMORRHAGE	1 (0.0)
GASTRIC CYST	1 (0.0)	CEREBRAL VENTRICLE DILATATION	1 (0.0)	PHLEBITIS	1 (0.0)	PHYSIOTHERAPY	1 (0.0)
OSTEOCHONDRITIS	1 (0.0)	DERMATITIS ATOPIC	1 (0.0)	PELVIC PAIN	1 (0.0)	INCREASED TENDENCY TO BRUISE	1 (0.0)
THROMBOPHLEBITIS	1 (0.0)	HIGH DENSITY LIPOPROTEIN DECREASED	1 (0.0)	HAEMORRHOIDAL HAEMORRHAGE	1 (0.0)	ELECTROCARDIOGRAM ABNORMAL	1 (0.0)
EPIGLOTTITIS	1 (0.0)	CRANIOTOMY	1 (0.0)	DERMATITIS ATOPIC	1 (0.0)	PNEUMONIA FUNGAL	1 (0.0)
LIVER INJURY	1 (0.0)	MUSCLE STRAIN	1 (0.0)	ARTERITIS	1 (0.0)	ENDODONTIC PROCEDURE	1 (0.0)
PUSTULAR PSORIASIS	1 (0.0)	ELECTROCARDIOGRAM ST-T SEGMENT ABNORMAL	1 (0.0)	ENDOCARDITIS	1 (0.0)	VAGINAL DISCHARGE	1 (0.0)
DRUG INTERACTIO	1 (0.0)	BLADDER NEOPLASM	1 (0.0)	COXSACKIE VIRAL	1 (0.0)	LACTIC ACIDOSIS	1 (0.0)

N				INFECTION			
THALASSAEMIA	1 (0.0)	BLOOD ELASTASE DECREASED	1 (0.0)	HERNIA REPAIR	1 (0.0)	HYDROTHORAX	1 (0.0)
BLOOD IRON ABNORMAL	1 (0.0)	PANCREATIC ENZYMES DECREASED	1 (0.0)	ALLERGY TO ARTHROPOD STING	1 (0.0)	RESTLESSNESS	1 (0.0)
SENSITIVE SKIN	1 (0.0)	BLADDER HYPERTROPHY	1 (0.0)	PITUITARY TUMOUR	1 (0.0)	MEDICAL DIET	1 (0.0)
ANGER	1 (0.0)	VITREOUS DETACHMENT	1 (0.0)	LEFT VENTRICULAR FAILURE	1 (0.0)	LOW CARBOHYDRATE DIET	1 (0.0)
JAW OPERATION	1 (0.0)	BLOOD IRON ABNORMAL	1 (0.0)	FOLLICULAR DISORDER	1 (0.0)	ANAESTHETIC COMPLICATION	1 (0.0)
MUSCLE HYPERTROPHY	1 (0.0)	VITAMIN B1 DECREASED	1 (0.0)	PRECANCEROUS SKIN LESION	1 (0.0)	SMALL INTESTINAL HAEMORRHAGE	1 (0.0)
INTESTINAL TRANSIT TIME DECREASED	1 (0.0)	OESOPHAGEAL DILATION PROCEDURE	1 (0.0)	MICTURITION DISORDER	1 (0.0)	STOMA SITE ULCER	1 (0.0)
GLIOBLASTOMA	1 (0.0)	UTERINE MASS	1 (0.0)	THROMBOLYSIS	1 (0.0)	TONGUE INJURY	1 (0.0)
CRYPTOSPORIDIOSIS INFECTION	1 (0.0)	LOOSE TOOTH	1 (0.0)	BRUXISM	1 (0.0)	GLOSSODYNIA	1 (0.0)
RECTAL POLYP	1 (0.0)	OCCIPITAL LOBE STROKE	1 (0.0)	SPINAL ARTERY THROMBOSIS	1 (0.0)	HEPATIC ENCEPHALOPATHY	1 (0.0)
NOCARDIOSIS	1 (0.0)	PORTAL HYPERTENSION	1 (0.0)	PROCEDURAL SITE REACTION	1 (0.0)	LARGE INTESTINAL ULCER HAEMORRHAGE	1 (0.0)
PETECHIAE	1 (0.0)	HERPES ZOSTER DISSEMINATED	1 (0.0)	HYPOTHYROIDISM	1 (0.0)	SHOCK	1 (0.0)

HAEMOPHAGOCYTIC LYMPHOHISTIOCYTOSIS	1 (0.0)	FRACTURED SACRUM	1 (0.0)	GASTROINTESTINAL VIRAL INFECTION	1 (0.0)	BILE DUCT STONE	1 (0.0)
DISSEMINATED INTRAVASCULAR COAGULATION	1 (0.0)	PULMONARY HAEMORRHAGE	1 (0.0)	SUPERFICIAL VEIN THROMBOSIS	1 (0.0)	CARDIAC PACEMAKER INSERTION	1 (0.0)
ENCEPHALITIS HERPES	1 (0.0)	PULMONARY VALVE REPLACEMENT	1 (0.0)	AXILLARY VEIN THROMBOSIS	1 (0.0)	POST PROCEDURAL FEVER	1 (0.0)
VITREOUS OPACITIES	1 (0.0)	PATIENT-DEVICE INCOMPATIBILITY	1 (0.0)	EXPULSION OF MEDICATION	1 (0.0)	PNEUMONIA NECROTISING	1 (0.0)
GASTROINTESTINAL MICROORGANISM OVERGROWTH	1 (0.0)	CENTRAL VENOUS CATHETERISATION	1 (0.0)	ACNE VARIOLIFORMIS	1 (0.0)	LIGAMENT SPRAIN	1 (0.0)
DERMATITIS CONTACT	1 (0.0)	TENDERNESS	1 (0.0)	DRUG EFFECT FASTER THAN EXPECTED	1 (0.0)	POSTMENOPAUSE	1 (0.0)
STENT PLACEMENT	1 (0.0)	ASTHMA EXERCISE INDUCED	1 (0.0)	ADRENAL DISORDER	1 (0.0)	BLOOD OESTROGEN INCREASED	1 (0.0)
LOW DENSITY LIPOPROTEIN ABNORMAL	1 (0.0)	METASTATIC BRONCHIAL CARCINOMA	1 (0.0)	NASAL OEDEMA	1 (0.0)	RECTAL ADENOCARCINOMA	1 (0.0)
CHOLANGITIS ACUTE	1 (0.0)	HEART RATE ABNORMAL	1 (0.0)	GENITAL BURNING SENSATION	1 (0.0)	URINE OUTPUT DECREASED	1 (0.0)
FEMORAL NECK FRACTURE	1 (0.0)	INTESTINAL FISTULA	1 (0.0)	PSORIATIC ARTHROPATHY	1 (0.0)	AXIAL SPONDYLOARTHRITIS	1 (0.0)
PNEUMOPE	1 (0.0)	STOMA	1 (0.0)	WHEELCHAI	1	OVARIAN	1 (0.0)

RITONEUM		COMPLICATION		R USER	(0.0)	CYST RUPTURED	
PNEUMONIA ASPIRATION	1 (0.0)	QUALITY OF LIFE DECREASED	1 (0.0)	SINUS HEADACHE	1 (0.0)	PNEUMATOSIS INTESTINALIS	1 (0.0)
PNEUMONITIS	1 (0.0)	STOMA SITE INDURATION	1 (0.0)	EAR DISORDER	1 (0.0)	TESTICULAR PAIN	1 (0.0)
POST PROCEDURAL SEPSIS	1 (0.0)	STOMA SITE ABSCESS	1 (0.0)	INTRAOCULAR PRESSURE INCREASED	1 (0.0)	HYPERTRIGLYCERIDAEMIA	1 (0.0)
TONGUE ULCERATION	1 (0.0)	MYOSITIS	1 (0.0)	LIPOSUCTION	1 (0.0)	PROTHROMBIN TIME SHORTENED	1 (0.0)
BREAST CANCER	1 (0.0)	CATARACT NUCLEAR	1 (0.0)	NECROSIS	1 (0.0)	PREGNANCY	1 (0.0)
RHEGMATOGENOUS RETINAL DETACHMENT	1 (0.0)	HETEROPHORIA	1 (0.0)	LYMPHOCYTE COUNT INCREASED	1 (0.0)	OPEN FRACTURE	1 (0.0)
FOLLICULAR LYMPHOMA	1 (0.0)	OBESITY	1 (0.0)	BLASTOMYCOSIS	1 (0.0)	COCCYDYNIA	1 (0.0)
PULMONARY TUBERCULOSIS	1 (0.0)	HYDROCEPHALUS	1 (0.0)	RENAL NEOPLASM	1 (0.0)	LOSS OF THERAPEUTIC RESPONSE	1 (0.0)
PSYCHIATRIC CARE	1 (0.0)	VITAMIN B12 DEFICIENCY	1 (0.0)	MENSTRUAL DISORDER	1 (0.0)	MACULAR ISCHAEMIA	1 (0.0)
ENDOMETRIOSIS	1 (0.0)	PROTEIN TOTAL ABNORMAL	1 (0.0)	HOSPICE CARE	1 (0.0)	RETINAL ARTERY OCCLUSION	1 (0.0)
CHOLECYSTITIS ACUTE	1 (0.0)	ELECTROCARDIOGRAM ABNORMAL	1 (0.0)	BONE LOSS	1 (0.0)	PERIPHERAL VASCULAR DISORDER	1 (0.0)
PSEUDOPOLYP	1 (0.0)	OCULAR DISCOMFORT	1 (0.0)	BLADDER SPASM	1 (0.0)	MUSCLE INJURY	1 (0.0)
STOMACH MASS	1 (0.0)	URETHRAL STENOSIS	1 (0.0)	ATELECTASIS	1 (0.0)	ABSCESS DRAINAGE	1 (0.0)
CHEST TUBE INSERTION	1 (0.0)	RADIATION PROCTITIS	1 (0.0)	LIMB MASS	1 (0.0)	RECTAL FISSURE	1 (0.0)

COCCIDIOIDOMYCOSIS	1 (0.0)	GENITAL SWELLING	1 (0.0)	ASTHENOPIA	1 (0.0)	PARKINSON'S DISEASE	1 (0.0)
RECTAL LESION	1 (0.0)	MALIGNANT PERITONEAL NEOPLASM	1 (0.0)	ENDOSCOPY	1 (0.0)	ESCHERICHIA SEPSIS	1 (0.0)
INTESTINAL POLYP	1 (0.0)	CARDIAC PROCEDURE COMPLICATION	1 (0.0)	GIANT CELL ARTERITIS	1 (0.0)	URETERAL STENT REMOVAL	1 (0.0)
HEPATIC CIRRHOSIS	1 (0.0)	DUODENAL ULCER HAEMORRHAGE	1 (0.0)	CHOLINERGIC SYNDROME	1 (0.0)	OLIGOMENORRHOEA	1 (0.0)
WOUND SEPSIS	1 (0.0)	URETERAL STENT REMOVAL	1 (0.0)	ATRIOVENTRICULAR DISSOCIATION	1 (0.0)	SKIN TEXTURE ABNORMAL	1 (0.0)
WOUND CELLULITIS	1 (0.0)	TIBIA FRACTURE	1 (0.0)	BLADDER PERFORATION	1 (0.0)	PREMENSTRUAL SYNDROME	1 (0.0)
BRAIN OEDEMA	1 (0.0)	EUPHORIC MOOD	1 (0.0)	DEVICE MALFUNCTION	1 (0.0)	HYPERTROPHY OF TONGUE PAPILLAE	1 (0.0)
STEROID DEPENDENCE	1 (0.0)	STAPHYLOCOCCAL SEPSIS	1 (0.0)	INJECTION SITE PAIN	1 (0.0)	RADIUS FRACTURE	1 (0.0)
CARDIO-RESPIRATORY ARREST	1 (0.0)	DERMATITIS ACNEIFORM	1 (0.0)	INJECTION	1 (0.0)	STEROID THERAPY	1 (0.0)
ACUTE FEBRILE NEUTROPHILIC DERMATOSIS	1 (0.0)	PROTHROMBIN TIME SHORTENED	1 (0.0)	PULMONARY FIBROSIS	1 (0.0)	EMERGENCY CARE	1 (0.0)
INCISION SITE DISCHARGE	1 (0.0)	INTESTINAL DILATATION	1 (0.0)	BLOODY DISCHARGE	1 (0.0)	TONGUE DISORDER	1 (0.0)
INCISION SITE RASH	1 (0.0)	CENTRAL NERVOUS SYSTEM LESION	1 (0.0)	MUCOSAL INFLAMMATION	1 (0.0)	ORAL MUCOSAL BLISTERING	1 (0.0)

INCISION SITE ERYTHEMA	1 (0.0)	MYELODYSPLASTIC SYNDROME	1 (0.0)	HERPES SIMPLEX OESOPHAGITIS	1 (0.0)	BLOOD VISCOSITY INCREASED	1 (0.0)
MIGRAINE WITH AURA	1 (0.0)	ROTATOR CUFF REPAIR	1 (0.0)	NASAL DISORDER	1 (0.0)	HYPERACUSIS	1 (0.0)
METASTATIC MALIGNANT MELANOMA	1 (0.0)	WOLFF-PARKINSON-WHITE SYNDROME	1 (0.0)	DRUG TOLERANCE	1 (0.0)	NAIL GROWTH ABNORMAL	1 (0.0)
CEREBRAL VENOUS SINUS THROMBOSIS	1 (0.0)	SPLENOMEGALY	1 (0.0)	CYSTITIS NONINFECTIVE	1 (0.0)	NAIL RIDGING	1 (0.0)
FOOD ALLERGY	1 (0.0)	PERIORBITAL CELLULITIS	1 (0.0)	CORONARY ARTERY THROMBOSIS	1 (0.0)	NAIL DISORDER	1 (0.0)
FAECES HARD	1 (0.0)	EXPIRED PRODUCT ADMINISTERED	1 (0.0)	MIDDLE INSOMNIA	1 (0.0)	ORCHITIS	1 (0.0)
POLLAKIURIA	1 (0.0)	VIRAL DIARRHOEA	1 (0.0)	RESTLESSNESS	1 (0.0)	DYSPNOEA AT REST	1 (0.0)
MEDICAL DEVICE SITE GRANULOMA	1 (0.0)	GASTROENTERITIS VIRAL	1 (0.0)	RESPIRATORY RATE INCREASED	1 (0.0)	DRUG LEVEL DECREASED	1 (0.0)
PROSTHESIS IMPLANTATION	1 (0.0)	SUBARACHNOID HAEMORRHAGE	1 (0.0)	TACHYPHRENIA	1 (0.0)	ORAL PAIN	1 (0.0)
ACUTE HEPATIC FAILURE	1 (0.0)	VITAMIN C DEFICIENCY	1 (0.0)	DEMENTIA ALZHEIMER'S TYPE	1 (0.0)	INNER EAR DISORDER	1 (0.0)
EXPULSION OF MEDICATION	1 (0.0)	LARYNGEAL DISORDER	1 (0.0)	PARKINSON'S DISEASE	1 (0.0)	ORAL MUCOSAL ERUPTION	1 (0.0)
EARLY SATIETY	1 (0.0)	GASTRIC PH DECREASED	1 (0.0)	SLEEP TERROR	1 (0.0)	BORDERLINE PERSONALIT	1 (0.0)

HYPOMAGNESAEMIA	1 (0.0)	DRUG ERUPTION	1 (0.0)	AXILLARY PAIN	1 (0.0)	Y DISORDER TREATMENT NONCOMPLIANCE	1 (0.0)
RENAL CYST	1 (0.0)	EPSTEIN-BARR VIRUS INFECTION REACTIVATION	1 (0.0)	HYPERPARATHYROIDISM	1 (0.0)	BOWEL PREPARATION	1 (0.0)
VASCULAR GRAFT	1 (0.0)	LAPAROSCOPY	1 (0.0)	BREAST CANCER METASTATIC	1 (0.0)	DISEASE PROGRESSION	1 (0.0)
DUODENITIS	1 (0.0)	COLON CANCER STAGE III	1 (0.0)	ANORECTAL SWELLING	1 (0.0)	COLONOSCOPY ABNORMAL	1 (0.0)
INCISIONAL HERNIA	1 (0.0)	GRIP STRENGTH DECREASED	1 (0.0)	TUMOUR HAEMORRHAGE	1 (0.0)	SMALL INTESTINAL ANASTOMOSIS	1 (0.0)
METABOLIC ACIDOSIS	1 (0.0)	METAMORPHOPSIA	1 (0.0)	TUMOUR RUPTURE	1 (0.0)	VITAL FUNCTIONS ABNORMAL	1 (0.0)
HEPATOTOXICITY	1 (0.0)	CRANIOFACIAL FRACTURE	1 (0.0)	URETERIC OBSTRUCTION	1 (0.0)	STRESS FRACTURE	1 (0.0)
MEDICATION ERROR	1 (0.0)	BRONCHIAL SECRETION RETENTION	1 (0.0)	STEVENS-JOHNSON SYNDROME	1 (0.0)	CYTOLOGY ABNORMAL	1 (0.0)
NERVE INJURY	1 (0.0)	PNEUMONIA RESPIRATORY SYNCYTIAL VIRAL	1 (0.0)	INTESTINAL TRANSIT TIME INCREASED	1 (0.0)	RECURRENT CANCER	1 (0.0)
ADENOMA BENIGN	1 (0.0)	DEVICE ISSUE	1 (0.0)	GENITAL INFECTION BACTERIAL	1 (0.0)	HYPOALBUMINAEMIA	1 (0.0)
NASAL OBSTRUCTION	1 (0.0)	TONGUE COATED	1 (0.0)	ANASTOMOTIC HAEMORRHAGE	1 (0.0)	INVASIVE DUCTAL BREAST CARCINOMA	1 (0.0)
WHEEZING	1 (0.0)	HYPERSENSITIVITY PNEUMONITIS	1 (0.0)	ELBOW OPERATION	1 (0.0)	ALVEOLITIS	1 (0.0)

STRESS URINARY INCONTINENCE	1 (0.0)	HEART FAILURE WITH REDUCED EJECTION FRACTION	1 (0.0)	BOWEN'S DISEASE	1 (0.0)	OPTIC ATROPHY	1 (0.0)
NASAL HERPES	1 (0.0)	PERIPHERAL VEIN THROMBOSIS	1 (0.0)	CERVICAL SPINAL STENOSIS	1 (0.0)	GLUCOSE TOLERANCE IMPAIRED	1 (0.0)
SLOW SPEECH	1 (0.0)	LOOSE BODY IN JOINT	1 (0.0)	MYOCARDIAL ISCHAEMIA	1 (0.0)	MOOD ALTERED	1 (0.0)
POST HERPETIC NEURALGIA	1 (0.0)	APPENDIX CANCER	1 (0.0)	GASTROINTESTINAL ULCER PERFORATION	1 (0.0)	HYPERTRANSAMINASAEMIA	1 (0.0)
MENINGITIS VIRAL	1 (0.0)	PRODUCT LOT NUMBER ISSUE	1 (0.0)	SKIN IRRITATION	1 (0.0)	HYPOKINESIA	1 (0.0)
AXILLARY VEIN THROMBOSIS	1 (0.0)	URINE ANALYSIS ABNORMAL	1 (0.0)	SKIN ODOUR ABNORMAL	1 (0.0)	AKINESIA	1 (0.0)
SUPERFICIAL VEIN THROMBOSIS	1 (0.0)	BRAIN NEOPLASM BENIGN	1 (0.0)	TENDON DISORDER	1 (0.0)	LIP PAIN	1 (0.0)
HYPOPHOSPHATAEMIA	1 (0.0)	LOWER RESPIRATORY TRACT INFECTION VIRAL	1 (0.0)	LYMPHOEDEMA	1 (0.0)	ORAL MUCOSAL ERYTHEMA	1 (0.0)
PROCEDURAL NAUSEA	1 (0.0)	MYOCARDIAL INJURY	1 (0.0)	ENTEROSTOMY CLOSURE	1 (0.0)	PLATELET COUNT ABNORMAL	1 (0.0)
MENISCUS INJURY	1 (0.0)	MYELOPATHY	1 (0.0)	OBSTRUCTION GASTRIC	1 (0.0)	RAYNAUD'S PHENOMENON	1 (0.0)
STOOL ANALYSIS ABNORMAL	1 (0.0)	POLYARTHRITIS	1 (0.0)	ADHESIOLYSIS	1 (0.0)	ALANINE AMINOTRANSFERASE	1 (0.0)

						ABNORMAL	
STAPHYLOCOCCAL SKIN INFECTION	1 (0.0)	OCCUPATIONAL EXPOSURE TO TOXIC AGENT	1 (0.0)	TURBINOPLASTY	1 (0.0)	SYSTEMIC INFECTION	1 (0.0)
INFECTED FISTULA	1 (0.0)	EXPOSURE TO RADIATION	1 (0.0)	NASAL SEPTAL OPERATION	1 (0.0)	GASTRIC ULCER HAEMORRHAGE	1 (0.0)
COLORECTAL CANCER	1 (0.0)	BRAIN NEOPLASM MALIGNANT	1 (0.0)	MENOPAUSE	1 (0.0)	OESOPHAGEAL STENOSIS	1 (0.0)
DIVERTICULUM OESOPHAGEAL	1 (0.0)	BLOOD LACTATE DEHYDROGENASE INCREASED	1 (0.0)	KERATOACANTHOMA	1 (0.0)	HEMIPARESIS	1 (0.0)
SMALL INTESTINE ULCER	1 (0.0)	BLOOD BETA-D-GLUCAN INCREASED	1 (0.0)	TRICHORRHEXIS	1 (0.0)	OPPORTUNISTIC INFECTION	1 (0.0)
STOMA SITE ULCER	1 (0.0)	KL-6 INCREASED	1 (0.0)	PHARYNGOTONSILLITIS	1 (0.0)	INFECTIOUS PLEURAL EFFUSION	1 (0.0)
ENCEPHALITIS AUTOIMMUNE	1 (0.0)	HYPERPARATHYROIDISM	1 (0.0)	GASTRIC DISORDER	1 (0.0)	PHOTOPSIA	1 (0.0)
BLOOD CREATININE ABNORMAL	1 (0.0)	ANORECTAL ULCER	1 (0.0)	HYPERKALAEMIA	1 (0.0)	CARDIAC HYPERTROPHY	1 (0.0)
JOINT WARMTH	1 (0.0)	HYPOPHOSPHATAEMIA	1 (0.0)	VASCULAR DEVICE INFECTION	1 (0.0)	MEDICAL DEVICE SITE INFECTION	1 (0.0)
TONGUE PRURITUS	1 (0.0)	HYPOCALCAEMIA	1 (0.0)	SKIN ATROPHY	1 (0.0)	POOR QUALITY PRODUCT ADMINISTERED	1 (0.0)
ORAL PRURITUS	1 (0.0)	FISTULA INFLAMMATION	1 (0.0)	CHAPPED LIPS	1 (0.0)	BONE MARROW OEDEMA SYNDROME	1 (0.0)

LUMBAR SPINAL STENOSIS	1 (0.0)	PYODERMA	1 (0.0)	ACUTE ABDOMEN	1 (0.0)	RENAL SURGERY	1 (0.0)
DENGUE FEVER	1 (0.0)	SKIN EROSION	1 (0.0)	TESTIS CANCER	1 (0.0)	VENOUS INJURY	1 (0.0)
PAPILLARY THYROID CANCER	1 (0.0)	LENTIGO	1 (0.0)	HEPATIC VEIN THROMBOSIS	1 (0.0)	SKIN ODOUR ABNORMAL	1 (0.0)
GASTROINTESTINAL NEUROENDOCRINE CARCINOMA	1 (0.0)	SHORT-BOWEL SYNDROME	1 (0.0)	PROSTATIC ABSCESS	1 (0.0)	SKIN INJURY	1 (0.0)
WEIGHT FLUCTUATION	1 (0.0)	SPINAL PAIN	1 (0.0)	KELOID SCAR	1 (0.0)	SKIN PAPILLOMA	1 (0.0)
GASTROINTESTINAL HYPERMOTILITY	1 (0.0)	ORAL PAPULE	1 (0.0)	HEPATECTOMY	1 (0.0)	HYPOGLYCAEMIA	1 (0.0)
THORACIC OPERATION	1 (0.0)	GASTROINTESTINAL PROCEDURAL COMPLICATION	1 (0.0)	TOOTH ABSCESS	1 (0.0)	ENTEROCOLITIS HAEMORRHAGIC	1 (0.0)
BODY FAT DISORDER	1 (0.0)	PATELLA FRACTURE	1 (0.0)	BLOOD BILIRUBIN DECREASED	1 (0.0)	WHITE BLOOD CELL COUNT	1 (0.0)
THIRST	1 (0.0)	RADICULOPATHY	1 (0.0)	EOSINOPHILIA	1 (0.0)	PERIRECTAL ABSCESS	1 (0.0)
TENDERNESS	1 (0.0)	PULMONARY HISTOPLASMOSIS	1 (0.0)	RECTAL ULCER	1 (0.0)	GASTROINTESTINAL BACTERIAL OVERGROWTH	1 (0.0)
LOCALISED OEDEMA	1 (0.0)	BUTTOCK INJURY	1 (0.0)	DEVICE LEAKAGE	1 (0.0)	POLYURIA	1 (0.0)
LEFT VENTRICULAR FAILURE	1 (0.0)	PRODUCT DISPENSING ERROR	1 (0.0)	HAND DERMATITIS	1 (0.0)	TONSILLAR DISORDER	1 (0.0)
EYE	1 (0.0)	LOCALISED	1 (0.0)	POSTURAL	1	HEPATOCEL	1 (0.0)

HAEMORRHAGE		MELANOMA		TREMOR	(0.0)	LULAR INJURY	
PHARYNGEAL MASS	1 (0.0)	SENSORY DISTURBANCE	1 (0.0)	MESENTERIC ARTERIAL OCCLUSION	1 (0.0)	METASTATIC SQUAMOUS CELL CARCINOMA	1 (0.0)
UMBILICAL HERNIA	1 (0.0)	LARGE INTESTINE ANASTOMOSIS	1 (0.0)	GENITAL ABSCESS	1 (0.0)	SQUAMOUS CELL CARCINOMA OF HEAD AND NECK	1 (0.0)
DEVICE BREAKAGE	1 (0.0)	URETERAL STENT INSERTION	1 (0.0)	NON-HODGKIN'S LYMPHOMA	1 (0.0)	PNEUMONIA VIRAL	1 (0.0)
ADVERSE REACTION	1 (0.0)	ADENOCARCINOMA OF COLON	1 (0.0)	BLOOD POTASSIUM INCREASED	1 (0.0)	AMYLASE INCREASED	1 (0.0)
BILIARY OBSTRUCTION	1 (0.0)	CATHETERISATION CARDIAC	1 (0.0)	RED BLOOD CELL COUNT INCREASED	1 (0.0)	RETINAL VEIN THROMBOSIS	1 (0.0)
INTESTINAL ULCER	1 (0.0)	SUICIDE THREAT	1 (0.0)	DYSMENORRHOEA	1 (0.0)	SKIN ULCER HAEMORRHAGE	1 (0.0)
ANORECTAL OPERATION	1 (0.0)	OCCUPATIONAL EXPOSURE TO SUNLIGHT	1 (0.0)	INTRACRANIAL ANEURYSM	1 (0.0)	ACROCHORDON	1 (0.0)
ABNORMAL DREAMS	1 (0.0)	EXPOSURE TO TOXIC AGENT	1 (0.0)	NECROTISING MYOSITIS	1 (0.0)	MULTIPLE SCLEROSIS	1 (0.0)
COELIAC ARTERY COMPRESSION SYNDROME	1 (0.0)	HERPES ZOSTER OTICUS	1 (0.0)	BEHCET'S SYNDROME	1 (0.0)	FRUSTRATION TOLERANCE DECREASED	1 (0.0)
EYE INFLAMMATION	1 (0.0)	FEBRILE NEUTROPENIA	1 (0.0)	RECTAL STENOSIS	1 (0.0)	EAR DISORDER	1 (0.0)
POSTPOLYPECTOMY SYNDROME	1 (0.0)	LIPOSUCTION	1 (0.0)	BARRETT'S OESOPHAGUS	1 (0.0)	STREPTOCOCCUS TEST POSITIVE	1 (0.0)
FISTULA	1 (0.0)	BURKITT'S	1 (0.0)	DENTAL	1	VERTIGO	1 (0.0)

INFLAMMATION		LYMPHOMA		RESTORATION FAILURE	(0.0)	POSITIONAL	
VAGINAL CYST	1 (0.0)	CORNEAL PERFORATION	1 (0.0)	BURSITIS	1 (0.0)	BLOOD DISORDER	1 (0.0)
BREAST CYST	1 (0.0)	SPINAL NERVE STIMULATOR REMOVAL	1 (0.0)	VULVAL DISORDER	1 (0.0)	EPSTEIN-BARR VIRUS ASSOCIATED LYMPHOPROLIFERATIVE DISORDER	1 (0.0)
DYSMENORRHOEA	1 (0.0)	CARDIAC MURMUR	1 (0.0)	VULVOVAGINAL ERYTHEMA	1 (0.0)	FUNGAL SKIN INFECTION	1 (0.0)
ADVERSE FOOD REACTION	1 (0.0)	MEDICAL DEVICE SITE PAIN	1 (0.0)	VULVOVAGINAL PAIN	1 (0.0)	HAEMORRHAGE SUBCUTANEOUS	1 (0.0)
GENITAL INFECTION FUNGAL	1 (0.0)	PROSTHESIS IMPLANTATION	1 (0.0)	VULVOVAGINAL DISCOMFORT	1 (0.0)	APPENDICEAL ABSCESS	1 (0.0)
NIPPLE DISORDER	1 (0.0)	HEPATOCELLULAR INJURY	1 (0.0)	VULVOVAGINAL PRURITUS	1 (0.0)	MUSCLE DISORDER	1 (0.0)
VULVOVAGINAL MYCOTIC INFECTION	1 (0.0)	FACIAL PAIN	1 (0.0)	GENITAL BLISTER	1 (0.0)	DRUG WITHDRAWAL SYNDROME	1 (0.0)
ENDOCRINE DISORDER	1 (0.0)	YERSINIA INFECTION	1 (0.0)	LICHENOID KERATOSIS	1 (0.0)	VARICELLA ZOSTER VIRUS INFECTION	1 (0.0)
ANXIETY DISORDER	1 (0.0)	HYPOPITUITARISM	1 (0.0)	SEMINOMA	1 (0.0)	BLOOD ALBUMIN DECREASED	1 (0.0)
BLOOD URINE PRESENT	1 (0.0)	DERMATITIS ALLERGIC	1 (0.0)	CHRONIC TRAUMATIC ENCEPHALOPATHY	1 (0.0)	ANIMAL ATTACK	1 (0.0)
RENAL ABSCESS	1 (0.0)	GINGIVITIS	1 (0.0)	NONSPECIFIC REACTION	1 (0.0)	FAILURE TO THRIVE	1 (0.0)
APPENDICIT	1 (0.0)	ORAL	1 (0.0)	GENERAL	1	IRREGULAR	1 (0.0)

IS NONINFECTIVE		DISCOMFORT		PHYSICAL CONDITION NORMAL	(0.0)	SLEEP PHASE	
CAESAREAN SECTION	1 (0.0)	ILEOCAECAL RESECTION	1 (0.0)	ABNORMAL UTERINE BLEEDING	1 (0.0)	LIP DISORDER	1 (0.0)
DYSARTHRIA	1 (0.0)	VITAMIN D DEFICIENCY	1 (0.0)	ENDOSCOPY SMALL INTESTINE	1 (0.0)	CORNEAL ABRASION	1 (0.0)
HAEMORRHAGIC STROKE	1 (0.0)	ANORECTAL POLYP	1 (0.0)	OBSTRUCTIVE AIRWAYS DISORDER	1 (0.0)	DRAINAGE	1 (0.0)
BREAST DISORDER	1 (0.0)	CRANIOCEREBRAL INJURY	1 (0.0)	TUBERCULIN TEST POSITIVE	1 (0.0)	PHOTOSENSITIVITY REACTION	1 (0.0)
COMPUTERISED TOMOGRAM ABNORMAL	1 (0.0)	TRAUMATIC INTRACRANIAL HAEMORRHAGE	1 (0.0)	HAEMANGIOMA	1 (0.0)	FEELING DRUNK	1 (0.0)
NODULE	1 (0.0)	SKIN OPERATION	1 (0.0)	GASTROINTESTINAL MUCOSAL DISORDER	1 (0.0)	CHONDROCALCINOSIS	1 (0.0)
TONGUE OEDEMA	1 (0.0)	DRUG LEVEL DECREASED	1 (0.0)	HELLP SYNDROME	1 (0.0)	POST PROCEDURAL DISCOMFORT	1 (0.0)
PHARYNGITIS	1 (0.0)	DYSPNOEA AT REST	1 (0.0)	SUNBURN	1 (0.0)	CERVICAL POLYP	1 (0.0)
MYOSITIS	1 (0.0)	GASTROINTESTINAL STOMA COMPLICATION	1 (0.0)	EAR CONGESTION	1 (0.0)	RENAL HAEMATOMA	1 (0.0)
FINE MOTOR SKILL DYSFUNCTION	1 (0.0)	TUBERCULOSIS	1 (0.0)	LARGE INTESTINE EROSION	1 (0.0)	CEREBRAL ARTERY EMBOLISM	1 (0.0)
MENINGIOMA	1 (0.0)	NODULE	1 (0.0)	CATHETER SITE RELATED	1 (0.0)	ANGIOEDEMA	1 (0.0)

				REACTION			
PURPURA	1 (0.0)	SKIN PROCEDURAL COMPLICATION	1 (0.0)	MENOPAUSAL SYMPTOMS	1 (0.0)	CUSHINGOID	1 (0.0)
BILIRUBIN CONJUGATED INCREASED	1 (0.0)	TOOTHACHE	1 (0.0)	BACTERIAL INFECTION INDEX INCREASED	1 (0.0)	BLOOD PRESSURE ORTHOSTATIC ABNORMAL	1 (0.0)
HEPATIC FIBROSIS	1 (0.0)	POST PROCEDURAL SWELLING	1 (0.0)	BILE DUCT STENOSIS	1 (0.0)	BENIGN NEOPLASM OF URETER	1 (0.0)
CARBOHYDRATE ANTIGEN 19-9 INCREASED	1 (0.0)	PANCREATIC CARCINOMA STAGE IV	1 (0.0)	METASTASES TO LYMPH NODES	1 (0.0)	TUBERCULIN TEST POSITIVE	1 (0.0)
HEPATIC INFECTION	1 (0.0)	VENTRICULAR EXTRASYSTOLES	1 (0.0)	EXPOSURE DURING PREGNANCY	1 (0.0)	TANNING	1 (0.0)
BILE DUCT STENT INSERTION	1 (0.0)	DROP ATTACKS	1 (0.0)	PREGNANCY	1 (0.0)	DYSKINESIA	1 (0.0)
SURGICAL PROCEDURE REPEATED	1 (0.0)	MENIERE'S DISEASE	1 (0.0)	RESPIRATORY TRACT INFECTION VIRAL	1 (0.0)	METAPNEUMOVIRUS INFECTION	1 (0.0)
SCAR EXCISION	1 (0.0)	TOOTH INJURY	1 (0.0)	GENITAL HERPES ZOSTER	1 (0.0)	SALPINGECTOMY	1 (0.0)
SERUM SICKNESS	1 (0.0)	SELF-INJURIOUS IDEATION	1 (0.0)	REMISSION NOT ACHIEVED	1 (0.0)	ORCHIDECTOMY	1 (0.0)
ANIMAL BITE	1 (0.0)	SQUAMOUS CELL CARCINOMA OF HEAD AND NECK	1 (0.0)	POST PROCEDURAL DISCOMFORT	1 (0.0)	COLORECTAL ADENOMA	1 (0.0)
URETERIC OBSTRUCTI	1 (0.0)	METASTATIC SQUAMOUS	1 (0.0)	MENSTRUATION DELAYED	1 (0.0)	AGRANULOCYTOSIS	1 (0.0)

ON		CELL CARCINOMA					
URINARY TRACT OBSTRUCTION	1 (0.0)	VENTRICULAR FIBRILLATION	1 (0.0)	DEFAECATION DISORDER	1 (0.0)	VULVOVAGINAL SWELLING	1 (0.0)
HIP ARTHROPLASTY	1 (0.0)	TEMPERATURE REGULATION DISORDER	1 (0.0)	ANASTOMOTIC ULCER HAEMORRHAGE	1 (0.0)	STREPTOCOCCAL SEPSIS	1 (0.0)
MASTECTOMY	1 (0.0)	CEREBRAL ARTERY EMBOLISM	1 (0.0)	INFLAMMATION OF WOUND	1 (0.0)	BUTTERFLY RASH	1 (0.0)
MECHANICAL ILEUS	1 (0.0)	LUMBAR VERTEBRAL FRACTURE	1 (0.0)	EMBEDDED DEVICE	1 (0.0)	RED BLOOD CELL COUNT ABNORMAL	1 (0.0)
NECK MASS	1 (0.0)	OXYGEN SATURATION ABNORMAL	1 (0.0)	POOR QUALITY SLEEP	1 (0.0)	LEUKAEMIA RECURRENT	1 (0.0)
CYTOMEGALOVIRUS TEST POSITIVE	1 (0.0)	THIRST	1 (0.0)	CHROMATOPSIA	1 (0.0)	HAEMATOCRIT ABNORMAL	1 (0.0)
VITAMIN D DECREASED	1 (0.0)	FOREIGN BODY SENSATION IN EYES	1 (0.0)	EXCESSIVE GRANULATION TISSUE	1 (0.0)	NEUROENDOCRINE CARCINOMA	1 (0.0)
DERMATITIS	1 (0.0)	CORNEAL DISORDER	1 (0.0)	HEPATIC FUNCTION ABNORMAL	1 (0.0)	ASPERGILLOMA	1 (0.0)
GASTROINTESTINAL PROCEDURAL COMPLICATION	1 (0.0)	INFUSION RELATED REACTION	1 (0.0)	OCULAR ICTERUS	1 (0.0)	ALLERGIC RESPIRATORY SYMPTOM	1 (0.0)
OSTEOPLASTY	1 (0.0)	PERICARDITIS	1 (0.0)	ANORECTAL CELLULITIS	1 (0.0)	INCISIONAL DRAINAGE	1 (0.0)
HYPERAEMIA	1 (0.0)	INTERNAL HERNIA	1 (0.0)	OESOPHAGEAL ULCER HAEMORRHAGE	1 (0.0)	UTERINE DILATION AND CURETTAGE	1 (0.0)
VIRAL	1 (0.0)	PEPTIC	1 (0.0)	DROOLING	1	THYROID	1 (0.0)

UPPER RESPIRATORY TRACT INFECTION		ULCER			(0.0)	OPERATION	
OVARIAN ABSCESS	1 (0.0)	ABDOMINAL INJURY	1 (0.0)	OBSESSIVE THOUGHTS	1 (0.0)	STRESS AT WORK	1 (0.0)
MUSCLE TWITCHING	1 (0.0)	MICTURITION URGENCY	1 (0.0)	INTRUSIVE THOUGHTS	1 (0.0)	RENAL NEOPLASM	1 (0.0)
ANAPHYLACTIC REACTION	1 (0.0)	BLOOD LACTIC ACID INCREASED	1 (0.0)	PARANOIA	1 (0.0)	PULMONARY INFARCTION	1 (0.0)
ANGIOEDEMA	1 (0.0)	ORAL FUNGAL INFECTION	1 (0.0)	PHOTOPSIA	1 (0.0)	GASTRITIS EROSIVE	1 (0.0)
PRODUCT PHYSICAL ISSUE	1 (0.0)	ORAL INFECTION	1 (0.0)	OSTEOMALACIA	1 (0.0)	LUNG HYPOINFLATION	1 (0.0)
PANIC DISORDER	1 (0.0)	PSEUDOSTROKE	1 (0.0)	BLOOD DISORDER	1 (0.0)	CARDIOMEGALY	1 (0.0)
DYSGEUSIA	1 (0.0)	HEAD DISCOMFORT	1 (0.0)	OESOPHAGEAL OBSTRUCTION	1 (0.0)	PATHOLOGICAL DISSECTION	1 (0.0)
RETINAL VEIN THROMBOSIS	1 (0.0)	TENSION HEADACHE	1 (0.0)	SPINAL NERVE STIMULATOR REMOVAL	1 (0.0)	ACQUIRED DIAPHRAGMATIC EVENTRATION	1 (0.0)
SUTURE RUPTURE	1 (0.0)	METASTATIC CARCINOMA OF THE BLADDER	1 (0.0)	ANORECTAL INFECTION	1 (0.0)	TEMPERATURE REGULATION DISORDER	1 (0.0)
FUNCTIONAL GASTROINTESTINAL DISORDER	1 (0.0)	INJURY ASSOCIATED WITH DEVICE	1 (0.0)	WEIGHT ABNORMAL	1 (0.0)	ALBUMIN GLOBULIN RATIO ABNORMAL	1 (0.0)
GASTROENTERITIS NOROVIRUS	1 (0.0)	HELICOBACTER GASTRITIS	1 (0.0)	EXTRADURAL ABSCESS	1 (0.0)	LIPID METABOLISM DISORDER	1 (0.0)
EMBOLIC CEREBRAL INFARCTION	1 (0.0)	ABNORMAL LOSS OF WEIGHT	1 (0.0)	LUNG ABSCESS	1 (0.0)	BRONCHOSPASM	1 (0.0)
HIP	1 (0.0)	INTESTINAL	1 (0.0)	THROAT	1	MACULAR	1 (0.0)

FRACTURE		SEPSIS		TIGHTNESS	(0.0)	HOLE	
EYE IRRITATION	1 (0.0)	INFLUENZA A VIRUS TEST POSITIVE	1 (0.0)	TICK-BORNE FEVER	1 (0.0)	SKIN OPERATION	1 (0.0)
MUSCLE RIGIDITY	1 (0.0)	POOR PERIPHERAL CIRCULATION	1 (0.0)	ECHOCARDIOGRAM	1 (0.0)	CALCIPHYLAXIS	1 (0.0)
PHARMACOPHOBIA	1 (0.0)	EXCESSIVE CERUMEN PRODUCTION	1 (0.0)	CHEST INJURY	1 (0.0)	CHALAZION	1 (0.0)
DYSTONIA	1 (0.0)	JOINT WARMTH	1 (0.0)	DIVERSION COLITIS	1 (0.0)	OTITIS MEDIA	1 (0.0)
CENTRAL NERVOUS SYSTEM VASCULITIS	1 (0.0)	ABDOMINAL LYMPHADENOPATHY	1 (0.0)	SERRATED POLYPOSIS SYNDROME	1 (0.0)	NASAL SEPTUM DEVIATION	1 (0.0)
FACIAL ASYMMETRY	1 (0.0)	ORAL DISORDER	1 (0.0)	PHARYNGEAL ERYTHEMA	1 (0.0)	IMPLANTABLE CARDIAC MONITOR INSERTION	1 (0.0)
SPERM CONCENTRATION DECREASED	1 (0.0)	LARGE INTESTINE OPERATION	1 (0.0)	PHARYNGEAL INFLAMMATION	1 (0.0)	DIABETIC KETOACIDOSIS	1 (0.0)
ORAL DISCOMFORT	1 (0.0)	PERIPHERAL NERVE INJURY	1 (0.0)	EFFUSION	1 (0.0)	FOCAL DYSCOGNITIVE SEIZURES	1 (0.0)
SUDDEN DEATH	1 (0.0)	ANIMAL ATTACK	1 (0.0)	HYDROURETER	1 (0.0)	PULMONARY CALCIFICATION	1 (0.0)
COLONOSCOPY	1 (0.0)	PROCEDURAL HAEMORRHAGE	1 (0.0)	VESICAL FISTULA	1 (0.0)	COLONOSCOPY	1 (0.0)
ABDOMINOPLASTY	1 (0.0)	TUMOUR EXCISION	1 (0.0)	GALLBLADDER OBSTRUCTION	1 (0.0)	GENERALISED TONIC-CLONIC SEIZURE	1 (0.0)
TOOTH	1 (0.0)	LOCALISED	1 (0.0)	METABOLIC	1	LENTIGO	1 (0.0)

EXTRACTION		OEDEMA		SURGERY	(0.0)	MALIGNA	
ADENOCARCINOMA OF COLON	1 (0.0)	PAROTITIS	1 (0.0)	ENDOSCOPY SMALL INTESTINE ABNORMAL	1 (0.0)	SUNBURN	1 (0.0)
HYPERAESTHESIA TEETH	1 (0.0)	PRURITUS GENITAL	1 (0.0)	END STAGE RENAL DISEASE	1 (0.0)	STOMA SITE INFECTION	1 (0.0)
PARAINFLUENZAE VIRUS INFECTION	1 (0.0)	TASTE DISORDER	1 (0.0)	INCORRECT ROUTE OF PRODUCT ADMINISTRATION	1 (0.0)	SIALOADENITIS	1 (0.0)
FOOD INTOLERANCE	1 (0.0)	BLADDER CANCER RECURRENT	1 (0.0)	CIRCULATORY COLLAPSE	1 (0.0)	INCORRECT PRODUCT ADMINISTRATION DURATION	1 (0.0)
PULMONARY CONGESTION	1 (0.0)	SUBDURAL EFFUSION	1 (0.0)	POST-ACUTE COVID-19 SYNDROME	1 (0.0)	CARBUNCLE	1 (0.0)
PERSONALITY CHANGE	1 (0.0)	BETA HAEMOLYTIC STREPTOCOCCAL INFECTION	1 (0.0)	IMMATURE GRANULOCYTE PERCENTAGE INCREASED	1 (0.0)	EYELID OEDEMA	1 (0.0)
AFFECTIVE DISORDER	1 (0.0)	BLOOD MAGNESIUM ABNORMAL	1 (0.0)	BLOOD CHLORIDE DECREASED	1 (0.0)	HEARING AID USER	1 (0.0)
VISUAL PROCESSING DISORDER	1 (0.0)	LARGE INTESTINAL ULCER PERFORATION	1 (0.0)	PROTEIN TOTAL INCREASED	1 (0.0)	TRAUMATIC LUNG INJURY	1 (0.0)
BLOOD CREATINE PHOSPHOKINASE ABNORMAL	1 (0.0)	ENTEROCOCCUS TEST POSITIVE	1 (0.0)	URINE KETONE BODY PRESENT	1 (0.0)	POSTMENOPAUSAL HAEMORRHAGE	1 (0.0)
WEIGHT LOSS POOR	1 (0.0)	ILEUS	1 (0.0)	MEAN PLATELET	1 (0.0)	UTERINE ENLARGEME	1 (0.0)

				VOLUME DECREASED		NT	
MENSTRUAL DISORDER	1 (0.0)	ESCHERICHIA TEST POSITIVE	1 (0.0)	PROTEIN URINE PRESENT	1 (0.0)	BLOOD CREATININE ABNORMAL	1 (0.0)
BREAST PAIN	1 (0.0)	CORONARY ARTERIAL STENT INSERTION	1 (0.0)	MEAN CELL HAEMOGLOBIN DECREASED	1 (0.0)	TONGUE COATED	1 (0.0)
SPINAL PAIN	1 (0.0)	LIP BLISTER	1 (0.0)	BLOOD UREA NITROGEN/CREATININE RATIO DECREASED	1 (0.0)	OESOPHAGEAL DISORDER	1 (0.0)
DYSPHEMIA	1 (0.0)	ORAL MUCOSAL BLISTERING	1 (0.0)	MEAN CELL VOLUME DECREASED	1 (0.0)	RESTRICTIVE PULMONARY DISEASE	1 (0.0)
COLONOSCOPY ABNORMAL	1 (0.0)	CARDIOGENIC SHOCK	1 (0.0)	GLOBULINS INCREASED	1 (0.0)	TUMOUR EXCISION	1 (0.0)
GENITAL LESION	1 (0.0)	RESPIRATORY SYNCYTIAL VIRUS BRONCHIOLITIS	1 (0.0)	PROTHROMBIN TIME PROLONGED	1 (0.0)	CARDIAC REHABILITATION THERAPY	1 (0.0)
TYMPANOPLASTY	1 (0.0)	NEUROMYELITIS OPTICA SPECTRUM DISORDER	1 (0.0)	BLOOD OSMOLARITY DECREASED	1 (0.0)	ARTHROSCOPIC SURGERY	1 (0.0)
PERIPHERAL VEIN THROMBOSIS	1 (0.0)	DUODENAL STENOSIS	1 (0.0)	NEUTROPHIL PERCENTAGE INCREASED	1 (0.0)	NASAL ULCER	1 (0.0)
ANTIBODY TEST ABNORMAL	1 (0.0)	ARTERIAL RUPTURE	1 (0.0)	LYMPHOCYTE PERCENTAGE DECREASED	1 (0.0)	FIBROSIS	1 (0.0)
CARDIOVASCULAR	1 (0.0)	HAEMOBILIA	1 (0.0)	ALBUMIN GLOBULIN	1 (0.0)	MALIGNANT NEOPLASM	1 (0.0)

SYMPTOM				RATIO DECREASED		OF UNKNOWN PRIMARY SITE	
WOUND HAEMORRH AGE	1 (0.0)	WHEELCHAI R USER	1 (0.0)	PELVIC INFECTION	1 (0.0)	UTERINE HAEMORRH AGE	1 (0.0)
VITAMIN B12 DEFICIENCY	1 (0.0)	CARDIOVAS CULAR DISORDER	1 (0.0)	COLOSTOMY INFECTION	1 (0.0)	DRUG-DEVIC E INTERACTIO N	1 (0.0)
CENTRAL VENOUS CATHETERI SATION	1 (0.0)	CHEMOTHE RAPY	1 (0.0)	LIPOMA	1 (0.0)	APPENDICE AL MUCOCOEL E	1 (0.0)
SCRATCH	1 (0.0)	EROSIVE DUODENITIS	1 (0.0)	GINGIVITIS	1 (0.0)	WOUND INFECTION STAPHYLOC OCCAL	1 (0.0)
PNEUMOCY STIS TEST POSITIVE	1 (0.0)	BREAST PROCEDUR AL COMPLICATI ON	1 (0.0)	VERTEBROB ASILAR ARTERY DISSECTION	1 (0.0)	VENOUS OPERATION	1 (0.0)
CYTOMEGA LOVIRUS VIRAEMIA	1 (0.0)	MASTECTO MY	1 (0.0)	DIABETIC KETOACIDO SIS	1 (0.0)	HYPORESPO NSIVE TO STIMULI	1 (0.0)
BLOOD ALKALINE PHOSPHAT ASE INCREASED	1 (0.0)	DIFFUSE LARGE B-CELL LYMPHOMA	1 (0.0)	PITUITARY TUMOUR BENIGN	1 (0.0)	HAND DEFORMITY	1 (0.0)
PLATELET COUNT ABNORMAL	1 (0.0)	HAEMORRH AGE URINARY TRACT	1 (0.0)	ORGANISING PNEUMONIA	1 (0.0)	NIPPLE DISORDER	1 (0.0)
INCREASED APPETITE	1 (0.0)	FINGER DEFORMITY	1 (0.0)	CANDIDA ENDOPHTHA LMITIS	1 (0.0)	BLEEDING TIME PROLONGE D	1 (0.0)
HYPERKALA EMIA	1 (0.0)	MUSCLE CONTRACTI ONS INVOLUNTA	1 (0.0)	MUSCLE DISORDER	1 (0.0)	SUPRAVENT RICULAR TACHYCARD IA	1 (0.0)

		RY					
SENSORY DISTURBANCE	1 (0.0)	TRISMUS	1 (0.0)	BREAST CANCER STAGE I	1 (0.0)	CATHETER SITE INFECTION	1 (0.0)
STOMA SITE INFLAMMATION	1 (0.0)	OEDEMA	1 (0.0)	URINE PROTEIN/CREATININE RATIO INCREASED	1 (0.0)	GIARDIASIS	1 (0.0)
BARTHOLINITIS	1 (0.0)	EYE PRURITUS	1 (0.0)	LIGHT CHAIN ANALYSIS INCREASED	1 (0.0)	PROCEDURAL VOMITING	1 (0.0)
NORMAL NEWBORN	1 (0.0)	AGITATION	1 (0.0)	RHINITIS	1 (0.0)	ARTHROPOD INFESTATION	1 (0.0)
PREMATURE LABOUR	1 (0.0)	GASTRIC BYPASS	1 (0.0)	BREAST MASS	1 (0.0)	LIP BLISTER	1 (0.0)
FACE OEDEMA	1 (0.0)	PRODUCT LABEL ISSUE	1 (0.0)	NEPHRITIS	1 (0.0)	HAEMANGIOMA OF LIVER	1 (0.0)
PREMATURE RUPTURE OF MEMBRANES	1 (0.0)	RIGHT VENTRICULAR DYSFUNCTION	1 (0.0)	PRECANCEROUS CONDITION	1 (0.0)	SERUM FERRITIN ABNORMAL	1 (0.0)
CERVICAL INCOMPETENCE	1 (0.0)	BLOOD ZINC DECREASED	1 (0.0)	ORAL MUCOSAL DISCOLOURATION	1 (0.0)	DEAFNESS TRANSITORY	1 (0.0)
HYPERPYREXIA	1 (0.0)	TYPE 2 DIABETES MELLITUS	1 (0.0)	OSTEOTOMY	1 (0.0)	EAR INFECTION FUNGAL	1 (0.0)
RADIATION ASSOCIATED PAIN	1 (0.0)	BLOOD PRESSURE SYSTOLIC ABNORMAL	1 (0.0)	PSOAS ABSCESS	1 (0.0)	POST PROCEDURAL HAEMATOMA	1 (0.0)
IMMUNOGLOBULINS DECREASED	1 (0.0)	GASTROINTESTINAL WALL THINNING	1 (0.0)	GASTROINTESTINAL NECROSIS	1 (0.0)	SPINAL NERVE STIMULATOR IMPLANTATION	1 (0.0)
FEMALE	1 (0.0)	PROCEDUR	1 (0.0)	VAGINAL	1	FEELING OF	1 (0.0)

GENITAL TRACT FISTULA		AL COMPLICATION		ULCERATION	(0.0)	BODY TEMPERATURE CHANGE	
INFLUENZA A VIRUS TEST POSITIVE	1 (0.0)	DIVERTICULAR PERFORATION	1 (0.0)	VAGINAL POLYP	1 (0.0)	LEGIONELLA INFECTION	1 (0.0)
PULMONARY VENOUS THROMBOSIS	1 (0.0)	WRIST SURGERY	1 (0.0)	MYOCARDITIS	1 (0.0)	FIBULA FRACTURE	1 (0.0)
ECTOPIC PREGNANCY	1 (0.0)	CEREBRAL ISCHAEMIA	1 (0.0)	INTESTINAL MUCOSAL HYPERTROPHY	1 (0.0)	COLORECTOSTOMY	1 (0.0)
HERPES SIMPLEX HEPATITIS	1 (0.0)	ENTEROCUTANEOUS FISTULA	1 (0.0)	GENERALISED OEDEMA	1 (0.0)	DRUG CLEARANCE INCREASED	1 (0.0)
CARDIAC ANEURYSM	1 (0.0)	INTERMENSTRUAL BLEEDING	1 (0.0)	GASTROENTERITIS ESCHERICHIA COLI	1 (0.0)	ARTHRODESIS	1 (0.0)
THORACIC HAEMORRHAGE	1 (0.0)	THYROID NEOPLASM	1 (0.0)	SCOLIOSIS	1 (0.0)	MENTAL STATUS CHANGES	1 (0.0)
INFLAMMATORY MARKER DECREASED	1 (0.0)	OPHTHALMIC HERPES SIMPLEX	1 (0.0)	MONOCLONAL GAMMOPATHY	1 (0.0)	THYROID CANCER METASTATIC	1 (0.0)
YERSINIA TEST POSITIVE	1 (0.0)	OPEN GLOBE INJURY	1 (0.0)	BLOOD CHLORIDE INCREASED	1 (0.0)	FEMALE STERILISATION	1 (0.0)
NOROVIRUS TEST POSITIVE	1 (0.0)	EPIGASTRIC DISCOMFORT	1 (0.0)	ARTHROPOD BITE	1 (0.0)	ANAL DILATION PROCEDURE	1 (0.0)
GUT DERIVED INFECTION	1 (0.0)	ENCEPHALITIS	1 (0.0)	ROCKY MOUNTAIN SPOTTED FEVER	1 (0.0)	ORGANISING PNEUMONIA	1 (0.0)
ANORECTAL STENOSIS	1 (0.0)	SMALL INTESTINE CARCINOMA	1 (0.0)	HYPERLIPIDAEMIA	1 (0.0)	RECTAL PROLAPSE	1 (0.0)
JOINT NOISE	1 (0.0)	PRODUCT ADMINISTRA	1 (0.0)	DIABETES MELLITUS	1 (0.0)	HAIR GROWTH	1 (0.0)

		TION ERROR		INADEQUATE CONTROL		ABNORMAL	
MILLER FISHER SYNDROME	1 (0.0)	ILEECTOMY	1 (0.0)	RENAL VEIN THROMBOSIS	1 (0.0)	UNDERWEIGHT	1 (0.0)
POST PROCEDURAL CONSTIPATION	1 (0.0)	HARVEY-BRADSHAW INDEX ABNORMAL	1 (0.0)	GUILLAIN-BARRE SYNDROME	1 (0.0)	PERFORATION	1 (0.0)
PANCREATIC FAILURE	1 (0.0)	HENOCH-SCHONLEIN PURPURA	1 (0.0)	OROPHARYNGEAL SQUAMOUS CELL CARCINOMA	1 (0.0)	OSTEITIS	1 (0.0)
MENSTRUAL CLOTS	1 (0.0)	FAECAL CALPROTECTIN DECREASED	1 (0.0)	PRECANCEROUS LESION OF DIGESTIVE TRACT	1 (0.0)	LICE INFESTATION	1 (0.0)
PANCREATIC DISORDER	1 (0.0)	PANIC REACTION	1 (0.0)	INDURATION	1 (0.0)	LIP ERYTHEMA	1 (0.0)
BLOOD ZINC DECREASED	1 (0.0)	HYPERAESTHESIA TEETH	1 (0.0)	CYST RUPTURE	1 (0.0)	GLOSSITIS	1 (0.0)
EAR SWELLING	1 (0.0)	URETERIC CANCER	1 (0.0)	STAPHYLOCOCCAL BACTERAEMIA	1 (0.0)	DISTURBANCE IN ATTENTION	1 (0.0)
PERIPHERAL ARTERY THROMBOSIS	1 (0.0)	PNEUMONIA LEGIONELLA	1 (0.0)	MEDICAL DEVICE SITE INJURY	1 (0.0)	SKIN ABRASION	1 (0.0)
NONINFECTIVE ENCEPHALITIS	1 (0.0)	FAECALOMA	1 (0.0)	APHASIA	1 (0.0)	ANORECTAL SWELLING	1 (0.0)
HEPATIC ENZYME ABNORMAL	1 (0.0)	ARTHROSCOPIC SURGERY	1 (0.0)	AGRAPHIA	1 (0.0)	TRANSVERSE SINUS THROMBOSIS	1 (0.0)
EXOSTOSIS	1 (0.0)	FACIAL BONES	1 (0.0)	NASAL DISCHARGE	1 (0.0)	ARTHROSCOPY	1 (0.0)

		FRACTURE		DISCOLOURATION			
PROTEIN TOTAL DECREASED	1 (0.0)	PROCEDURAL NAUSEA	1 (0.0)	FACIAL OPERATION	1 (0.0)	CLOSTRIDIUM COLITIS	1 (0.0)
PSORIATIC ARTHROPATHY	1 (0.0)	ORGAN TRANSPLANT	1 (0.0)	VULVOVAGINAL DRYNESS	1 (0.0)	BACTERIAL TEST POSITIVE	1 (0.0)
BIOCHEMICAL PREGNANCY	1 (0.0)	EYE COLOUR CHANGE	1 (0.0)	RENAL CELL CARCINOMA	1 (0.0)	HYPERTENSIVE HEART DISEASE	1 (0.0)
PHYSICAL DECONDITIONING	1 (0.0)	BACTERIAL SEPSIS	1 (0.0)	IMMUNE THROMBOCYTOPENIA	1 (0.0)	RETCHING	1 (0.0)
MENINGITIS BACTERIAL	1 (0.0)	HUMERUS FRACTURE	1 (0.0)	RENAL STONE REMOVAL	1 (0.0)	INFLAMMATORY PAIN	1 (0.0)
EXTRADURAL ABSCESS	1 (0.0)	ELBOW OPERATION	1 (0.0)	SQUAMOUS CELL CARCINOMA OF THE TONGUE	1 (0.0)	INTERLEUKIN-2 RECEPTOR INCREASED	1 (0.0)
TRANSURETHRAL PROSTATECTOMY	1 (0.0)	MACULAR HOLE	1 (0.0)	OVARIAN CYST RUPTURED	1 (0.0)	TOOTH ABSCESS	1 (0.0)
PRE-EXISTING CONDITION IMPROVED	1 (0.0)	ACUTE HAEMORRHAGIC ULCERATIVE COLITIS	1 (0.0)	INADEQUATE DIET	1 (0.0)	PANIC DISORDER	1 (0.0)
ANASTOMOTIC FISTULA	1 (0.0)	GLUCOSE-6-PHOSPHATE DEHYDROGENASE DEFICIENCY	1 (0.0)	MAGNETIC RESONANCE IMAGING ABNORMAL	1 (0.0)	ERYSIPELAS	1 (0.0)
GASTRITIS BACTERIAL	1 (0.0)	VARICOSE VEIN	1 (0.0)	PSEUDOPORPHYRIA	1 (0.0)	DISCOURAGEMENT	1 (0.0)
MEDICAL DEVICE CHANGE	1 (0.0)	APPETITE DISORDER	1 (0.0)	TOXICITY TO VARIOUS AGENTS	1 (0.0)	INTRACRANIAL ANEURYSM	1 (0.0)
HYPERTHERMIA	1 (0.0)	HAEMORRHAGIC	1 (0.0)	URINARY HESITATION	1 (0.0)	NON-HIGH-DENSITY	1 (0.0)

		DIATHESIS				LIPOPROTEIN CHOLESTEROL INCREASED	
BLOOD ACID PHOSPHATASE INCREASED	1 (0.0)	EXTRANODAL MARGINAL ZONE B-CELL LYMPHOMA (MALT TYPE)	1 (0.0)	HERPANGINA	1 (0.0)	VERY LOW DENSITY LIPOPROTEIN INCREASED	1 (0.0)
SHOULDER OPERATION	1 (0.0)	LYMPHADENECTOMY	1 (0.0)	GOITRE	1 (0.0)	CHOKING SENSATION	1 (0.0)
PURULENCE	1 (0.0)	DISABILITY	1 (0.0)	PULMONARY CALCIFICATION	1 (0.0)	BREAST MASS	1 (0.0)
PHARYNGOTONSILLITIS	1 (0.0)	NEUROMYOPATHY	1 (0.0)	BILIARY COLIC	1 (0.0)	THYROIDECTOMY	1 (0.0)
VASECTOMY	1 (0.0)	NEUROPATHY PERIPHERAL	1 (0.0)	RETINAL OEDEMA	1 (0.0)	PSEUDOMONAS INFECTION	1 (0.0)
HEART RATE IRREGULAR	1 (0.0)	WEIGHT BEARING DIFFICULTY	1 (0.0)	POLYCYSTIC OVARIAN SYNDROME	1 (0.0)	HYPOGAMMAGLOBULINAEMIA	1 (0.0)
HYPERTHERMIA MALIGNANT	1 (0.0)	EXERCISE LACK OF	1 (0.0)	EMPHYSEMA	1 (0.0)	SEPTIC PULMONARY EMBOLISM	1 (0.0)
RETICULOCYTE COUNT DECREASED	1 (0.0)	GOUT	1 (0.0)	HERPES ZOSTER DISSEMINATED	1 (0.0)	SITTING DISABILITY	1 (0.0)
TOOTH IMPACTED	1 (0.0)	HAEMODYNAMIC INSTABILITY	1 (0.0)	CARDIAC FLUTTER	1 (0.0)	INTRA-ABDOMINAL HAEMATOMA	1 (0.0)
ENDOMETRIAL NEOPLASM	1 (0.0)	TROPONIN T INCREASED	1 (0.0)	LAPAROTOMY	1 (0.0)	RHEUMATOID NODULE	1 (0.0)
TUMOUR PAIN	1 (0.0)	ARTERIOSCLEROSIS CORONARY ARTERY	1 (0.0)	WOUND CLOSURE	1 (0.0)	ALLERGY TO PLANTS	1 (0.0)
BODY TEMPERATU	1 (0.0)	HEART VALVE	1 (0.0)	TYMPANIC MEMBRANE	1 (0.0)	HEPATIC ARTERY	1 (0.0)

RE ABNORMAL		INCOMPETENCE		PERFORATION		ANEURYSM	
PARVOVIRUS INFECTION	1 (0.0)	TEMPERATURE INTOLERANCE	1 (0.0)	LACUNAR INFARCTION	1 (0.0)	ARTERIAL DISORDER	1 (0.0)
HEPATITIS ACUTE	1 (0.0)	RENAL CANCER	1 (0.0)	ALLODYNIA	1 (0.0)	HEPATIC ARTERY HAEMORRHAGE	1 (0.0)
SUTURE RELATED COMPLICATION	1 (0.0)	OBSTRUCTIVE NEPHROPATHY	1 (0.0)	HYPERAESTHESIA	1 (0.0)	CAROTID ARTERY DISEASE	1 (0.0)
LIPOMA	1 (0.0)	HYDRONEPHROSIS	1 (0.0)	PORTAL HYPERTENSION	1 (0.0)	TENDON INJURY	1 (0.0)
PRECANCEROUS CONDITION	1 (0.0)	URETEROLITHIASIS	1 (0.0)	DEAFNESS BILATERAL	1 (0.0)	THYROID MASS	1 (0.0)
NEUROLOGICAL SYMPTOM	1 (0.0)	URINE PHOSPHORUS INCREASED	1 (0.0)	DEAFNESS TRANSITORY	1 (0.0)	SPINAL DEFORMITY	1 (0.0)
CELLULITIS ORBITAL	1 (0.0)	URINE CALCIUM INCREASED	1 (0.0)	PULMONARY PAIN	1 (0.0)	RED BLOOD CELL ABNORMALITY	1 (0.0)
GASTROINTESTINAL ANASTOMOTIC STENOSIS	1 (0.0)	URINARY TRACT OBSTRUCTION	1 (0.0)	MIGRAINE-TRIGGERED SEIZURE	1 (0.0)	IRRITABLE BOWEL SYNDROME	1 (0.0)
ABDOMINAL HERNIA	1 (0.0)	CALCULUS URETHRAL	1 (0.0)	JOINT DISLOCATION	1 (0.0)	CHEST INJURY	1 (0.0)
POSTOPERATIVE ADHESION	1 (0.0)	SPIDER VEIN	1 (0.0)	PARADOXICAL PSORIASIS	1 (0.0)	COMPUTERISED TOMOGRAM THORAX ABNORMAL	1 (0.0)
DEPRESSIVE SYMPTOM	1 (0.0)	JAUNDICE ACHOLURIC	1 (0.0)	FAT TISSUE INCREASED	1 (0.0)	TOOTH LOSS	1 (0.0)
CEREBRAL	1 (0.0)	LARYNGEAL	1 (0.0)	DANDRUFF	1	BONE	1 (0.0)

DISORDER		NEOPLASM			(0.0)	DENSITY DECREASED	
BRAIN OPERATION	1 (0.0)	HERPES ZOSTER INFECTION NEUROLOGICAL	1 (0.0)	ACNE PUSTULAR	1 (0.0)	TRANSIENT GLOBAL AMNESIA	1 (0.0)
GASTRIC ULCER	1 (0.0)	MYELOSUPPRESSION	1 (0.0)	VITREORETINAL TRACTION SYNDROME	1 (0.0)	BLOOD IMMUNOGLOBULIN G DECREASED	1 (0.0)
RECTAL ULCER HAEMORRHAGE	1 (0.0)	SCRATCH	1 (0.0)	STEATORRHOEA	1 (0.0)	ABDOMINAL WALL ABSCESS	1 (0.0)
ANAL CANCER	1 (0.0)	RECTAL SPASM	1 (0.0)	GINGIVAL SWELLING	1 (0.0)	BRONCHOPULMONARY ASPERGILLOSIS	1 (0.0)
BILE DUCT STENT REMOVAL	1 (0.0)	NAIL INFECTION	1 (0.0)	GASTRIC BYPASS	1 (0.0)	FOOD AVERSION	1 (0.0)
WEIGHT ABNORMAL	1 (0.0)	PERIPHERAL COLDNESS	1 (0.0)	FEMALE GENITAL OPERATION	1 (0.0)	ATRIOVENTRICULAR BLOCK	1 (0.0)
LIP HAEMORRHAGE	1 (0.0)	AGEUSIA	1 (0.0)	GASTROINTESTINAL BACTERIAL INFECTION	1 (0.0)	ATRIAL THROMBOSIS	1 (0.0)
MOUTH HAEMORRHAGE	1 (0.0)	SUSPECTED COVID-19	1 (0.0)	BENIGN NEOPLASM	1 (0.0)	MAGNESIUM DEFICIENCY	1 (0.0)
LYMPHATIC SYSTEM NEOPLASM	1 (0.0)	ANOSMIA	1 (0.0)	OCULAR MELANOMA	1 (0.0)	DRY THROAT	1 (0.0)
GALLBLADDER OEDEMA	1 (0.0)	AORTIC STENOSIS	1 (0.0)	PNEUMONIA CYTOMEGALOVIRAL	1 (0.0)	PULSE ABSENT	1 (0.0)
PROCEDURAL INTESTINAL PERFORATION	1 (0.0)	RENAL CELL CARCINOMA	1 (0.0)	PERTUSSIS	1 (0.0)	MONOPARESIS	1 (0.0)
DIVERTICUL	1 (0.0)	HEPATIC	1 (0.0)	SUPERFICIAL	1	MUSCLE	1 (0.0)

UM		MASS		INJURY OF EYE	(0.0)	TWITCHING	
INCISION SITE IMPAIRED HEALING	1 (0.0)	ANGIOPATHY	1 (0.0)	APLASTIC ANAEMIA	1 (0.0)	MUCOSAL HAEMORRHAGE	1 (0.0)
HEPATOSPLENOMEGALY	1 (0.0)	DYSPLASIA	1 (0.0)	DRUG SPECIFIC ANTIBODY PRESENT	1 (0.0)	SYSTEMIC CANDIDA	1 (0.0)
PERIPHERAL VEIN OCCLUSION	1 (0.0)	CAUDA EQUINA SYNDROME	1 (0.0)	BLOOD TESTOSTERONE ABNORMAL	1 (0.0)	ONYCHOCLASIS	1 (0.0)
PELVIC VENOUS THROMBOSIS	1 (0.0)	LUMBAR RADICULOPATHY	1 (0.0)	DEVICE RELATED INFECTION	1 (0.0)	BONE GRAFT	1 (0.0)
ORAL CANDIDIASIS	1 (0.0)	INVASIVE LOBULAR BREAST CARCINOMA	1 (0.0)	PATIENT-DEVICE INCOMPATIBILITY	1 (0.0)	TONGUE OEDEMA	1 (0.0)
PANCREATIC CARCINOMA	1 (0.0)	SINUS ARRHYTHMIA	1 (0.0)	SYSTEMIC INFECTION	1 (0.0)	MICTURITION FREQUENCY DECREASED	1 (0.0)
ENERGY INCREASED	1 (0.0)	NASAL OPERATION	1 (0.0)	INTESTINAL ULCER PERFORATION	1 (0.0)	PNEUMONIA ASPIRATION	1 (0.0)
HAND-FOOT-AND-MOUTH DISEASE	1 (0.0)	CORTICAL HAND STROKE	1 (0.0)	ANAL DILATION PROCEDURE	1 (0.0)	INFECTED DERMAL CYST	1 (0.0)
MUSCLE SWELLING	1 (0.0)	FAT NECROSIS	1 (0.0)	PULMONARY VALVE REPLACEMENT	1 (0.0)	URINE ABNORMALITY	1 (0.0)
DROWNING	1 (0.0)	DISTURBANCE IN SOCIAL BEHAVIOUR	1 (0.0)	OESOPHAGEAL ULCER	1 (0.0)	NERVE BLOCK	1 (0.0)
PRODUCT CONTAMINATION	1 (0.0)	BLOOD IRON INCREASED	1 (0.0)	PELVIC FLUID COLLECTION	1 (0.0)	BILIARY TRACT DISORDER	1 (0.0)

OVERGROWTH FUNGAL	1 (0.0)	SERUM FERRITIN INCREASED	1 (0.0)	STOMA SITE ODOUR	1 (0.0)	VIRAL RASH	1 (0.0)
HUMAN PAPILLOMA VIRUS REACTIVATION	1 (0.0)	EJECTION FRACTION DECREASED	1 (0.0)	VULVOVAGINAL INJURY	1 (0.0)	MILIARIA	1 (0.0)
TOOTH DISCOLOURATION	1 (0.0)	ANTI FACTOR XA ACTIVITY DECREASED	1 (0.0)	URETHRAL STENOSIS	1 (0.0)	DIAPHRAGMATIC HERNIA	1 (0.0)
BLOOD CREATINE INCREASED	1 (0.0)	GLOMERULAR FILTRATION RATE ABNORMAL	1 (0.0)	HEPATIC INFECTION	1 (0.0)	TONGUE PRURITUS	1 (0.0)
ORAL SURGERY	1 (0.0)	SYMPTOM RECURRENCE	1 (0.0)	AGGRESSION	1 (0.0)	GINGIVAL BLISTER	1 (0.0)
BLOOD THYROID STIMULATING HORMONE INCREASED	1 (0.0)	ANAL FISSURE HAEMORRHAGE	1 (0.0)	MOOD SWINGS	1 (0.0)	HERPES ZOSTER REACTIVATION	1 (0.0)
WOUND ABSCESS	1 (0.0)	CANCER PAIN	1 (0.0)	TENDON OPERATION	1 (0.0)	VOLVULUS	1 (0.0)
CUTANEOUS T-CELL LYMPHOMA	1 (0.0)	PERIORBITAL SWELLING	1 (0.0)	HYPERALDOSTERONISM	1 (0.0)	LUMBAR SPINAL STENOSIS	1 (0.0)
POSTOPERATIVE THROMBOSIS	1 (0.0)	POST HERPETIC NEURALGIA	1 (0.0)	ANAL SPASM	1 (0.0)	CORNEAL PERFORATION	1 (0.0)
BLOOD BLISTER	1 (0.0)	BLOOD PH INCREASED	1 (0.0)	POSTURAL ORTHOSTATIC TACHYCARDIA SYNDROME	1 (0.0)	JOINT WARMTH	1 (0.0)
POST PROCEDURAL	1 (0.0)	DENTAL CARIES	1 (0.0)	ANGIOEDEMA	1 (0.0)	DYSMENORRHOEA	1 (0.0)

INFLAMMATION LARGE INTESTINE ANASTOMOSIS	1 (0.0)	SJOGREN'S SYNDROME	1 (0.0)	RENAL INFARCT	1 (0.0)	VAGINAL PROLAPSE	1 (0.0)
SENSATION OF FOREIGN BODY	1 (0.0)	SEPTIC ARTHRITIS STAPHYLOCOCCAL	1 (0.0)	RENAL HAEMORRHAGE	1 (0.0)	THROMBOPHLEBITIS	1 (0.0)
MYELIN OLIGODENDROCYTE GLYCOPROTEIN ANTIBODY-ASSOCIATED DISEASE	1 (0.0)	INCOHERENT	1 (0.0)	ENDOMETRIAL CANCER	1 (0.0)	PETECHIAE	1 (0.0)
ABDOMINAL RIGIDITY	1 (0.0)	INTRAOCULAR PRESSURE TEST ABNORMAL	1 (0.0)	MALIGNANT NEOPLASM PROGRESSION	1 (0.0)	LIVER SCAN ABNORMAL	1 (0.0)
PERIPORTAL OEDEMA	1 (0.0)	OCULAR PROCEDURAL COMPLICATION	1 (0.0)	GASTRIC CYST	1 (0.0)	SPLEEN ATROPHY	1 (0.0)
BLADDER HYPERTROPHY	1 (0.0)	OCULAR STROKE	1 (0.0)	PNEUMONIA BORDETELLA	1 (0.0)	TENDERNESS	1 (0.0)
COCCYDYNIA	1 (0.0)	INCISION SITE HAEMORRHAGE	1 (0.0)	BREAST ABSCESS	1 (0.0)	ORAL HERPES ZOSTER	1 (0.0)
SHOULDER FRACTURE	1 (0.0)	INCISIONAL HERNIA	1 (0.0)	MEDICAL DEVICE SITE ERYTHEMA	1 (0.0)	PRODUCT DOSE OMISSION IN ERROR	1 (0.0)
DRAINAGE	1 (0.0)	INCISION SITE ABSCESS	1 (0.0)	MEDICAL DEVICE SITE RASH	1 (0.0)	CERVICAL SPINAL STENOSIS	1 (0.0)
PRODUCT PRESCRIBIN	1 (0.0)	GINGIVAL PAIN	1 (0.0)	MEDICAL DEVICE SITE	1 (0.0)	HELPLESSNESS	1 (0.0)

G ISSUE				INFLAMMATION			
BLOOD TESTOSTERONE ABNORMAL	1 (0.0)	EXOPHTHALMOS	1 (0.0)	TENDERNESS	1 (0.0)	ISCHAEMIC STROKE	1 (0.0)
URINE OUTPUT DECREASED	1 (0.0)	BLOOD CREATININE DECREASED	1 (0.0)	ADVERSE REACTION	1 (0.0)	LATENT TUBERCULOSIS	1 (0.0)
PERTUSSIS	1 (0.0)	HYPERVOLAEMIA	1 (0.0)	CENTRAL VENOUS CATHETERISATION	1 (0.0)	CSF VIRUS IDENTIFIED	1 (0.0)
OEDEMA	1 (0.0)	BREAST DISCOMFORT	1 (0.0)	HORMONE LEVEL ABNORMAL	1 (0.0)	VERTEBRAL FORAMINAL STENOSIS	1 (0.0)
TROPONIN INCREASED	1 (0.0)	LIVER TRANSPLANT	1 (0.0)	PARONYCHIA	1 (0.0)	ESCHERICHIA TEST POSITIVE	1 (0.0)
TONSILLOLITH	1 (0.0)	SCROTAL ABSCESS	1 (0.0)	THALASSAEMIA	1 (0.0)	FAECAL VOLUME DECREASED	1 (0.0)
ATTENTION DEFICIT HYPERACTIVITY DISORDER	1 (0.0)	BREAST CANCER MALE	1 (0.0)	RADIATION PROCTITIS	1 (0.0)	RHINALGIA	1 (0.0)
SPINAL FUSION SURGERY	1 (0.0)	LIMB MASS	1 (0.0)	LOOSE TOOTH	1 (0.0)	SPLENIC VEIN THROMBOSIS	1 (0.0)
NEPHROURETERECTOMY	1 (0.0)	STOMACH MASS	1 (0.0)	UTERINE MASS	1 (0.0)	SUICIDE ATTEMPT	1 (0.0)
FEELING OF DESPAIR	1 (0.0)	DRUG MONITORING PROCEDURE NOT PERFORMED	1 (0.0)	VITAMIN B1 DECREASED	1 (0.0)	PLANTAR FASCIITIS	1 (0.0)
BOWEL MOVEMENT IRREGULARI	1 (0.0)	COLOSTOMY BAG USER	1 (0.0)	INTERVERTEBRAL DISC DISORDER	1 (0.0)	INFECTION PARASITIC	1 (0.0)

TY							
INTESTINAL ANASTOMO SIS COMPLICAT ION	1 (0.0)	ANASTOMO TIC ULCER	1 (0.0)	SPINAL STENOSIS	1 (0.0)	EOSINOPHIL IA	1 (0.0)
ABSCESS DRAINAGE	1 (0.0)	HEPATOTOX ICITY	1 (0.0)	LARGE INTESTINAL ULCER HAEMORRH AGE	1 (0.0)	SINGLE FUNCTIONA L KIDNEY	1 (0.0)
SMALL INTESTINAL ANASTOMO SIS	1 (0.0)	CERVICAL VERTEBRAL FRACTURE	1 (0.0)	CAMPYLOBA CTER GASTROENT ERITIS	1 (0.0)	NECK INJURY	1 (0.0)
MELAENA	1 (0.0)	MONOPLEGI A	1 (0.0)	STOMA SITE INDURATION	1 (0.0)	ORBITAL DECOMPRE SSION	1 (0.0)
PROCEDUR AL SITE REACTION	1 (0.0)	ANKLE ARTHROPLA STY	1 (0.0)	INFECTIOUS MONONUCL EOSIS	1 (0.0)	PALLIATIVE CARE	1 (0.0)
BRUXISM	1 (0.0)	NEUTROPHI L COUNT INCREASED	1 (0.0)	QUALITY OF LIFE DECREASED	1 (0.0)	MUSCULOS KELETAL CHEST PAIN	1 (0.0)
THROMBOL YSIS	1 (0.0)	DIVERTICULI TIS INTESTINAL PERFORATE D	1 (0.0)	CRYING	1 (0.0)	FUNGAL FOOT INFECTION	1 (0.0)
GINGIVAL PAIN	1 (0.0)	PCO2 ABNORMAL	1 (0.0)	CARDIO-RES PIRATORY ARREST	1 (0.0)	LIPASE INCREASED	1 (0.0)
HYPERTRAN SAMINASAE MIA	1 (0.0)	ABDOMINAL TENDERNES S	1 (0.0)	SUBARACHN OID HAEMORRH AGE	1 (0.0)	MULTIPLE SCLEROSIS RELAPSE	1 (0.0)
BLADDER SPASM	1 (0.0)	LIMB DISCOMFOR T	1 (0.0)	UMBILICOPL ASTY	1 (0.0)	EYE CONTUSION	1 (0.0)
TENDON RUPTURE	1 (0.0)	LIGAMENT PAIN	1 (0.0)	VIRAL DIARRHOEA	1 (0.0)	GASTROINT ESTINAL TRACT IRRITATION	1 (0.0)
HOSPICE	1 (0.0)	PURULENCE	1 (0.0)	EXPIRED	1	OROPHARY	1 (0.0)

CARE				PRODUCT ADMINISTERED	(0.0)	NGEAL DISCOMFORT	
GLAUCOMA	1 (0.0)	PNEUMOPERITONEUM	1 (0.0)	GASTROINTESTINAL DILATION PROCEDURE	1 (0.0)	EYE COLOUR CHANGE	1 (0.0)
PRODUCT COLOUR ISSUE	1 (0.0)	SEROSITIS	1 (0.0)	THYROID CANCER RECURRENT	1 (0.0)	LYMPHOMATOID PAPULOSIS	1 (0.0)
DIVERTICULUM INTESTINAL HAEMORRHAGIC	1 (0.0)	PLEURAL EFFUSION	1 (0.0)	SPLENIC INFARCTION	1 (0.0)	TOOTH IMPACTED	1 (0.0)
STOMA SITE REACTION	1 (0.0)	LOW DENSITY LIPOPROTEIN INCREASED	1 (0.0)	WOLFF-PARKINSON-WHITE SYNDROME	1 (0.0)	ERECTILE DYSFUNCTION	1 (0.0)
DISSEMINATED MYCOBACTERIUM AVIUM COMPLEX INFECTION	1 (0.0)	BODY MASS INDEX ABNORMAL	1 (0.0)	ROTATOR CUFF REPAIR	1 (0.0)	RADICAL PROSTATECTOMY	1 (0.0)
		LIPASE INCREASED	1 (0.0)	UMBILICAL HERNIA REPAIR	1 (0.0)	HYPOTHYROIDISM	1 (0.0)
		HYPERBILIRUBINAEMIA	1 (0.0)	CENTRAL NERVOUS SYSTEM LESION	1 (0.0)	CAMPYLOBACTER INFECTION	1 (0.0)
		STOMAL HERNIA	1 (0.0)	CARDIAC PROCEDURE COMPLICATION	1 (0.0)	UTERINE LEIOMYOMA	1 (0.0)
		TRICHORRHEXIS	1 (0.0)	EXTRA DOSE ADMINISTERED	1 (0.0)	UTERINE MALPOSITION	1 (0.0)
		MOUTH HAEMORRHAGE	1 (0.0)	FIBROMYALGIA	1 (0.0)	ISCHAEMIC SKIN ULCER	1 (0.0)

NECK MASS	1 (0.0)	MUSCLE DISCOMFORT	1 (0.0)	CSF WHITE BLOOD CELL COUNT POSITIVE	1 (0.0)
BLOOD CALCIUM INCREASED	1 (0.0)	HAEMORRHAGE SUBCUTANEOUS	1 (0.0)	ACCIDENTAL EXPOSURE TO PRODUCT	1 (0.0)
RENAL TUBULAR NECROSIS	1 (0.0)	BLOOD LACTATE DEHYDROGENASE INCREASED	1 (0.0)	MACULAR DEGENERATION	1 (0.0)
LIMB DEFORMITY	1 (0.0)	HEPATOCELLULAR INJURY	1 (0.0)	CONCOMITANT DISEASE AGGRAVATED	1 (0.0)
THERAPY NON-RESPONDER	1 (0.0)	MICTURITION URGENCY	1 (0.0)	BLADDER PAIN	1 (0.0)
BREAST RECONSTRUCTION	1 (0.0)	ENTEROVESICAL FISTULA	1 (0.0)	COMPUTERISED TOMOGRAM HEART ABNORMAL	1 (0.0)
CYTOMEGALOVIRUS CHORIORETINITIS	1 (0.0)	MYCOBACTERIUM TUBERCULOSIS COMPLEX TEST POSITIVE	1 (0.0)	CARDIAC DEVICE IMPLANTATION	1 (0.0)
ISCHAEMIA	1 (0.0)	GENITAL INFECTION FUNGAL	1 (0.0)	POSTOPERATIVE ILEUS	1 (0.0)
INTERMITTENT CLAUDICATION	1 (0.0)	MAGNESIUM DEFICIENCY	1 (0.0)	GASTRIC OPERATION	1 (0.0)
ATRIAL SEPTAL DEFECT	1 (0.0)	METASTASES TO PERITONEUM	1 (0.0)	METABOLIC DISORDER	1 (0.0)
MENINGITIS	1 (0.0)	TONGUE	1	HIV	1 (0.0)

TUBERCULOUS		ULCERATION	(0.0)	INFECTION	
PICKWICKIAN SYNDROME	1 (0.0)	PNEUMOPERITONEUM	1 (0.0)	MULTIMORBIDITY	1 (0.0)
THROMBOCYTOSIS	1 (0.0)	FEMORAL NECK FRACTURE	1 (0.0)	PETIT MAL EPILEPSY	1 (0.0)
HAEMATOLOGICAL INFECTION	1 (0.0)	PHARYNGITIS	1 (0.0)	MELAENA	1 (0.0)
JAUNDICE	1 (0.0)	GASTRIC PH DECREASED	1 (0.0)	POST THROMBOTIC SYNDROME	1 (0.0)
ACUTE MYELOID LEUKAEMIA	1 (0.0)	LARYNGEAL DISORDER	1 (0.0)	CARDIAC DYSFUNCTION	1 (0.0)
CHRONIC MYELOID LEUKAEMIA	1 (0.0)	VITAMIN C DEFICIENCY	1 (0.0)	DIPLEGIA	1 (0.0)
PELVIC ABSCESS	1 (0.0)	GASTROINTESTINAL MICROORGANISM OVERGROWTH	1 (0.0)	RED CELL DISTRIBUTION WIDTH INCREASED	1 (0.0)
NEUTROPHIL COUNT ABNORMAL	1 (0.0)	TONGUE COATED	1 (0.0)	MONOCYTE PERCENTAGE INCREASED	1 (0.0)
PERIPHERAL NERVE DECOMPRESSION	1 (0.0)	LENTIGO	1 (0.0)	MEAN PLATELET VOLUME DECREASED	1 (0.0)
PRE-EXISTING CONDITION IMPROVED	1 (0.0)	SKIN EROSION	1 (0.0)	IRON BINDING CAPACITY UNSATURATED INCREASED	1 (0.0)
THROMBOTIC STROKE	1 (0.0)	PYODERMA	1 (0.0)	VITAMIN B12 INCREASED	1 (0.0)
GINGIVAL	1 (0.0)	CHEST TUBE	1	IRON	1 (0.0)

DISORDER		INSERTION	(0.0)	BINDING CAPACITY TOTAL INCREASED	
TONGUE DISORDER	1 (0.0)	KLEBSIELLA INFECTION	1 (0.0)	ACTIVATED PARTIAL THROMBOPLASTIN TIME PROLONGED	1 (0.0)
OOPHORITIS	1 (0.0)	VAGINAL DISCHARGE	1 (0.0)	TRANSFERRIN SATURATION INCREASED	1 (0.0)
AXILLARY MASS	1 (0.0)	ORAL DISORDER	1 (0.0)	INTESTINAL VASCULAR DISORDER	1 (0.0)
CONTRAINDICATION TO MEDICAL TREATMENT	1 (0.0)	BLOOD ACID PHOSPHATASE INCREASED	1 (0.0)	CRYPTITIS	1 (0.0)
RENAL ATROPHY	1 (0.0)	BLOOD BILIRUBIN INCREASED	1 (0.0)	COELIAC DISEASE	1 (0.0)
MEDICAL DEVICE REMOVAL	1 (0.0)	HYPERTENSIVE CRISIS	1 (0.0)	GASTROINTESTINAL MUCOSAL EXFOLIATION	1 (0.0)
PRODUCT CONTAMINATION	1 (0.0)	METASTASES TO LIVER	1 (0.0)	DISSEMINATED TUBERCULOSIS	1 (0.0)
OBSTRUCTIVE PANCREATITIS	1 (0.0)	SCARLET FEVER	1 (0.0)	CORONARY ARTERY EMBOLISM	1 (0.0)
BILE DUCT STONE	1 (0.0)	MASTITIS	1 (0.0)	WEIGHT GAIN POOR	1 (0.0)
LYMPHADENITIS	1 (0.0)	ATRIAL SEPTAL DEFECT	1 (0.0)	CEREBELLAR INFARCTION	1 (0.0)
PYURIA	1 (0.0)	MYOGLOBIN	1	PNEUMOME	1 (0.0)

		BLOOD INCREASED	(0.0)	DIASTINUM	
BACTERIAL COLITIS	1 (0.0)	PERONEAL NERVE PALSY	1 (0.0)	ENDOCARDITIS	1 (0.0)
LUNG CARCINOMA CELL TYPE UNSPECIFIED STAGE IV	1 (0.0)	SKIN PROCEDURAL COMPLICATION	1 (0.0)	EMBOLIC STROKE	1 (0.0)
NERVE INJURY	1 (0.0)	SKIN OPERATION	1 (0.0)	CEREBROSPINAL FLUID RETENTION	1 (0.0)
ANAL FISTULA INFECTION	1 (0.0)	TRAUMATIC INTRACRANIAL HAEMORRHAGE	1 (0.0)	CUSHING'S SYNDROME	1 (0.0)
PNEUMOCYSTIS JIROVECII INFECTION	1 (0.0)	CRANIOCEREBRAL INJURY	1 (0.0)	EXPOSURE TO COMMUNICABLE DISEASE	1 (0.0)
VAGINAL ABSCESS	1 (0.0)	AORTIC ANEURYSM	1 (0.0)	CRYPTOSPORIDIOSIS INFECTION	1 (0.0)
SUPERFICIAL INJURY OF EYE	1 (0.0)	VITAMIN D DEFICIENCY	1 (0.0)	PROCEDURAL NAUSEA	1 (0.0)
PRODUCT USE IN UNAPPROVED INDICATION	1 (0.0)	METABOLIC ACIDOSIS	1 (0.0)	BLOOD GLUCOSE DECREASED	1 (0.0)
ORAL PAIN	1 (0.0)	INCISIONAL HERNIA	1 (0.0)	GLIOBLASTOMA	1 (0.0)
GENERAL SYMPTOM	1 (0.0)	HYPOMAGNESAEMIA	1 (0.0)	SYSTEMIC LUPUS ERYTHEMATOSUS RASH	1 (0.0)
HYPERTROPHIC CARDIOMYOPATHY	1 (0.0)	EARLY SATIETY	1 (0.0)	INTESTINAL TRANSIT TIME DECREASED	1 (0.0)

ARTHROPOD BITE	1 (0.0)	CEREBRAL VENOUS SINUS THROMBOSIS	1 (0.0)	BLOOD CREATINE PHOSPHOKINASE ABNORMAL	1 (0.0)
CSF WHITE BLOOD CELL COUNT DECREASED	1 (0.0)	MIGRAINE WITH AURA	1 (0.0)	PSYCHIATRIC CARE	1 (0.0)
CHONDROPATHY	1 (0.0)	PULMONARY HISTOPLASMOSIS	1 (0.0)	FACIAL BONES FRACTURE	1 (0.0)
INTESTINAL ULCER	1 (0.0)	RADICULOPATHY	1 (0.0)	SKIN SQUAMOUS CELL CARCINOMA RECURRENT	1 (0.0)
ILEOCOLOSTOMY	1 (0.0)	PATELLA FRACTURE	1 (0.0)	AUTOIMMUNE NEUTROPENIA	1 (0.0)
HYPOXIA	1 (0.0)	ALLERGIC RESPIRATORY SYMPTOM	1 (0.0)	HEPATORENAL SYNDROME	1 (0.0)
INFECTED DERMAL CYST	1 (0.0)	ALLERGIC SINUSITIS	1 (0.0)	CIRRHOSIS ALCOHOLIC	1 (0.0)
PNEUMONIA ESCHERICHIA	1 (0.0)	INJURY ASSOCIATED WITH DEVICE	1 (0.0)	SPONTANEOUS BACTERIAL PERITONITIS	1 (0.0)
ANAL STENOSIS	1 (0.0)	LUMBAR SPINAL STENOSIS	1 (0.0)	ALCOHOLIC LIVER DISEASE	1 (0.0)
PAPULE	1 (0.0)	CARDIAC VENTRICULAR THROMBOSIS	1 (0.0)	URETERIC CANCER	1 (0.0)
SUBDURAL ABSCESS	1 (0.0)	LUMBAR VERTEBRAL FRACTURE	1 (0.0)	DERMATITIS CONTACT	1 (0.0)
VERTEBROB	1 (0.0)	DIVERTICUL	1	DENTAL	1 (0.0)

ASILAR STROKE		UM OESOPHAGEAL	(0.0)	RESTORATION FAILURE	
PERIPHERAL NERVE OPERATION	1 (0.0)	COLORECTAL CANCER	1 (0.0)	MUSCLE CONTRACTIONS INVOLUNTARY	1 (0.0)
PORTAL VEIN EMBOLISM	1 (0.0)	STAPHYLOCOCCAL SKIN INFECTION	1 (0.0)	PULMONARY FIBROSIS	1 (0.0)
SNAKE BITE	1 (0.0)	BREAST CONSERVING SURGERY	1 (0.0)	PARANASAL CYST	1 (0.0)
LOCAL REACTION	1 (0.0)	BLOOD LACTIC ACID INCREASED	1 (0.0)	INTERVERTEBRAL DISCITIS	1 (0.0)
BREAST NEOPLASM	1 (0.0)	DEVICE PHYSICAL PROPERTY ISSUE	1 (0.0)	OESOPHAGEAL OPERATION	1 (0.0)
HEART RATE DECREASED	1 (0.0)	DISEASE COMPLICATION	1 (0.0)	MUCOSAL DISORDER	1 (0.0)
INFECTIOUS MONONUCLEOSIS	1 (0.0)	ARTERIOVENOUS MALFORMATION	1 (0.0)	BACTERIAL FOOD POISONING	1 (0.0)
CUTANEOUS VASCULITIS	1 (0.0)	TOOTH INJURY	1 (0.0)	CEREBRAL INFARCTION	1 (0.0)
PURPURA	1 (0.0)	PROCEDURAL NAUSEA	1 (0.0)	INTESTINAL METASTASIS	1 (0.0)
ARTERIOSCLEROSIS	1 (0.0)	RESPIRATORY DISTRESS	1 (0.0)	OCCIPITAL LOBE STROKE	1 (0.0)
		HEPATITIS B SURFACE ANTIBODY POSITIVE	1 (0.0)	REMISSION NOT ACHIEVED	1 (0.0)
		HEPATITIS B CORE ANTIBODY POSITIVE	1 (0.0)	SKIN MACERATION	1 (0.0)
		HEPATITIS E	1	PROTEIN	1 (0.0)

ANTIBODY POSITIVE	(0.0)	TOTAL INCREASED	
ANASTOMOTIC LEAK	1 (0.0)	BLADDER REPAIR	1 (0.0)
LEFT ATRIAL ENLARGEMENT	1 (0.0)	RECTAL PROLAPSE REPAIR	1 (0.0)
MIDDLE CEREBRAL ARTERY STROKE	1 (0.0)	GLIONEURONAL TUMOUR	1 (0.0)
INTESTINAL MUCOSAL TEAR	1 (0.0)	ANAL HAEMORRHAGE	1 (0.0)
OESOPHAGITIS BACTERIAL	1 (0.0)	UNINTENDED PREGNANCY	1 (0.0)
PULMONARY INFARCTION	1 (0.0)	ARTERIAL HAEMORRHAGE	1 (0.0)
VENTRICULAR FIBRILLATION	1 (0.0)	LUNG ABSCESS	1 (0.0)
CERVIX CARCINOMA	1 (0.0)	HYPOXIC-ISCHAEMIC ENCEPHALOPATHY	1 (0.0)
EXCESSIVE CERUMEN PRODUCTION	1 (0.0)	ASPHYXIA	1 (0.0)
INFARCTION	1 (0.0)	INTESTINAL SEPSIS	1 (0.0)
SMALL INTESTINAL ANASTOMOSIS	1 (0.0)	OEDEMA MUCOSAL	1 (0.0)
DENGUE FEVER	1 (0.0)	MYOSITIS	1 (0.0)
PLATELET COUNT ABNORMAL	1 (0.0)	MACULAR OEDEMA	1 (0.0)
HAND	1	UTERINE	1 (0.0)

FRACTURE	(0.0)	SPASM	
LARGE INTESTINAL ULCER PERFORATION	1 (0.0)	BLOOD GLUCOSE ABNORMAL	1 (0.0)
BLOOD MAGNESIUM ABNORMAL	1 (0.0)	EYELID PTOSIS	1 (0.0)
PRODUCT DOSE OMISSION IN ERROR	1 (0.0)	DYSLIPIDAEMIA	1 (0.0)
ADRENAL MASS	1 (0.0)	FINGER DEFORMITY	1 (0.0)
ENTEROCOLITIS	1 (0.0)	SKIN ULCER	1 (0.0)
BODY FAT DISORDER	1 (0.0)	MITE ALLERGY	1 (0.0)
ABDOMINAL INJURY	1 (0.0)	BLOOD TRIGLYCERIDES ABNORMAL	1 (0.0)
INTERNAL HERNIA	1 (0.0)	HIGH DENSITY LIPOPROTEIN ABNORMAL	1 (0.0)
GASTROINTESTINAL HYPERMOTILITY	1 (0.0)	BRADYCARDIA	1 (0.0)
JOINT WARMTH	1 (0.0)	UPPER RESPIRATORY TRACT CONGESTION	1 (0.0)
BILE ACID MALABSORPTION	1 (0.0)	UNDERDOSE	1 (0.0)
GASTROINTESTINAL NEUROENDOCRINE	1 (0.0)	MENINGITIS VIRAL	1 (0.0)

CARCINOMA			
GLOMERULAR FILTRATION RATE ABNORMAL	1 (0.0)	ISCHAEMIA	1 (0.0)
PNEUMONIA STREPTOCOCCAL	1 (0.0)	MUSCLE ENZYME INCREASED	1 (0.0)
POST-TRAUMATIC NECK SYNDROME	1 (0.0)	GENITAL SWELLING	1 (0.0)
DIFFUSE LARGE B-CELL LYMPHOMA	1 (0.0)	OESOPHAGEAL DILATION PROCEDURE	1 (0.0)
GASTROINTESTINAL NEUROENDOCRINE TUMOUR	1 (0.0)	ACUTE PROMYELOCYTIC LEUKAEMIA	1 (0.0)
EYE HAEMORRHAGE	1 (0.0)	THYROID DISORDER	1 (0.0)
LYMPHOMA	1 (0.0)	ADRENAL DISORDER	1 (0.0)
EROSIVE DUODENITIS	1 (0.0)	OSTEOCHONDRITIS	1 (0.0)
HAEMOBILIA	1 (0.0)	BURKITT'S LYMPHOMA	1 (0.0)
ARTERIAL RUPTURE	1 (0.0)	METASTATIC BRONCHIAL CARCINOMA	1 (0.0)
DUODENAL STENOSIS	1 (0.0)	ASTHMA EXERCISE INDUCED	1 (0.0)
NEUROMYELITIS OPTICA SPECTRUM DISORDER	1 (0.0)	CHAPPED LIPS	1 (0.0)
RESPIRATORY SYNCYTIAL VIRUS BRONCHIOLI	1 (0.0)	GASTROINTESTINAL CANCER METASTATIC	1 (0.0)

TIS			
CARDIOGENIC SHOCK	1 (0.0)	NEPHROCALCINOSIS	1 (0.0)
ANORECTAL OPERATION	1 (0.0)	HYPERCALCIURIA	1 (0.0)
BILIARY OBSTRUCTION	1 (0.0)	TREATMENT DELAYED	1 (0.0)
ESCHERICHIA TEST POSITIVE	1 (0.0)	SUPERFICIAL VEIN THROMBOSIS	1 (0.0)
ENTEROCOCCUS TEST POSITIVE	1 (0.0)	MUSCLE HYPERTROPHY	1 (0.0)
THYROID NEOPLASM	1 (0.0)	JEJUNOSTOMY	1 (0.0)
ENTEROCUTANEOUS FISTULA	1 (0.0)	MYELODYSPLASTIC SYNDROME	1 (0.0)
SUBCUTANEOUS ABSCESS	1 (0.0)	HERPES OESOPHAGITIS	1 (0.0)
DIVERTICULAR PERFORATION	1 (0.0)	LEFT VENTRICULAR FAILURE	1 (0.0)
MEDICAL DEVICE REMOVAL	1 (0.0)	SKIN COSMETIC PROCEDURE	1 (0.0)
PURPURA	1 (0.0)	B-CELL LYMPHOMA	1 (0.0)
MENINGIOMA	1 (0.0)	PHARYNGEAL INFLAMMATION	1 (0.0)
FINE MOTOR SKILL DYSFUNCTION	1 (0.0)	DRUG SPECIFIC ANTIBODY PRESENT	1 (0.0)
PARALYSIS	1 (0.0)	ACUTE HEPATIC FAILURE	1 (0.0)
RIGHT	1	HAEMATOTO	1 (0.0)

VENTRICULAR DYSFUNCTION	(0.0)	XICITY	
PRODUCT LABEL ISSUE	1 (0.0)	ORTHOPAEDIC PROCEDURE	1 (0.0)
CAESAREAN SECTION	1 (0.0)	MALIGNANT PERITONEAL NEOPLASM	1 (0.0)
SMALL INTESTINAL ULCER HAEMORRHAGE	1 (0.0)	VENOUS THROMBOSIS	1 (0.0)
APPENDICITIS NONINFECTIVE	1 (0.0)	EXPIRED PRODUCT ADMINISTERED	1 (0.0)
HAEMORRHAGE URINARY TRACT	1 (0.0)	VULVOVAGINAL PRURITUS	1 (0.0)
POSTPOLYPECTOMY SYNDROME	1 (0.0)	ENDOMETRIOSIS	1 (0.0)
ACUTE RESPIRATORY DISTRESS SYNDROME	1 (0.0)	AUTONOMIC NERVOUS SYSTEM IMBALANCE	1 (0.0)
HYPOGAMMAGLOBULINAEMIA	1 (0.0)	LOOSE BODY IN JOINT	1 (0.0)
SKIN BACTERIAL INFECTION	1 (0.0)	EPIGLOTTITIS	1 (0.0)
RECTOSIGMOID CANCER METASTATIC	1 (0.0)	HEART FAILURE WITH REDUCED EJECTION FRACTION	1 (0.0)
SKIN WEEPING	1 (0.0)	HYPERSENSITIVITY	1 (0.0)

		PNEUMONITIS	
INTESTINAL ATONY	1 (0.0)	METAMORPHOPSIA	1 (0.0)
HYPOTONIA	1 (0.0)	CHOLECYSTITIS	1 (0.0)
OPEN GLOBE INJURY	1 (0.0)	COLON CANCER STAGE III	1 (0.0)
OPHTHALMIC HERPES SIMPLEX	1 (0.0)	LAPAROSCOPY	1 (0.0)
SKIN HAEMORRHAGE	1 (0.0)	CHOLANGITIS ACUTE	1 (0.0)
SERUM SICKNESS	1 (0.0)	UMBILICOPLASTY	1 (0.0)
SEASONAL ALLERGY	1 (0.0)	OPHTHALMIC VASCULAR THROMBOSIS	1 (0.0)
SCAR EXCISION	1 (0.0)	VITREOUS OPACITIES	1 (0.0)
SURGICAL PROCEDURE REPEATED	1 (0.0)	CALCULUS BLADDER	1 (0.0)
LUMBAR PUNCTURE	1 (0.0)	TONSIL CANCER	1 (0.0)
STOMA SITE REACTION	1 (0.0)	HAEMOPHAGOCYTIC LYMPHOHISTIOCYTOSIS	1 (0.0)
ACROCHORDON	1 (0.0)	BRAIN OEDEMA	1 (0.0)
HENOCH-SCHONLEIN PURPURA	1 (0.0)	WOUND CELLULITIS	1 (0.0)
OVARIAN ABSCESS	1 (0.0)	WOUND SEPSIS	1 (0.0)
VIRAL UPPER RESPIRATORY TRACT INFECTION	1 (0.0)	PNEUMONIA RESPIRATORY SYNCYTIAL	1 (0.0)

		VIRAL	
HYPERAEMIA	1 (0.0)	BRONCHIAL SECRETION RETENTION	1 (0.0)
OSTEOPLASTY	1 (0.0)	RECTAL LESION	1 (0.0)
GINGIVAL BLEEDING	1 (0.0)	COCCIDIOIDOMYCOSIS	1 (0.0)
SPONDYLITIS	1 (0.0)	ANORECTAL ULCER	1 (0.0)
FEMALE GENITAL TRACT FISTULA	1 (0.0)	POLYARTHRITIS	1 (0.0)
SMALL INTESTINE CARCINOMA	1 (0.0)	MYELOPATHY	1 (0.0)
NECK MASS	1 (0.0)	MYOCARDIAL INJURY	1 (0.0)
MECHANICAL ILEUS	1 (0.0)	LOWER RESPIRATORY TRACT INFECTION VIRAL	1 (0.0)
EPIGASTRIC DISCOMFORT	1 (0.0)	BRAIN NEOPLASM BENIGN	1 (0.0)
TEMPOROMANDIBULAR JOINT SYNDROME	1 (0.0)	HYPERTONIC BLADDER	1 (0.0)
LOCALISED OEDEMA	1 (0.0)	CHOLECYSTITIS ACUTE	1 (0.0)
MULTIPLE ORGAN DYSFUNCTION SYNDROME	1 (0.0)	INCISION SITE RASH	1 (0.0)
HYPERAESTHESIA TEETH	1 (0.0)	INCISION SITE DISCHARGE	1 (0.0)
EYE SWELLING	1 (0.0)	LOCALISED MELANOMA	1 (0.0)
OESOPHAGE	1	ACUTE	1 (0.0)

AL STENOSIS	(0.0)	FEBRILE NEUTROPHILIC DERMATOSIS	
SKIN ABRASION	1 (0.0)	SLOW SPEECH	1 (0.0)
MEDICATION ERROR	1 (0.0)	NASAL HERPES	1 (0.0)
OSTEOMYELITIS	1 (0.0)	STRESS URINARY INCONTINENCE	1 (0.0)
COLONOSCOPY	1 (0.0)	ORAL FUNGAL INFECTION	1 (0.0)
CENTRAL NERVOUS SYSTEM VASCULITIS	1 (0.0)	PERFORATED ULCER	1 (0.0)
DYSTONIA	1 (0.0)	OVARIAN CANCER	1 (0.0)
PHARMACOPHOBIA	1 (0.0)	NASAL OBSTRUCTION	1 (0.0)
AUTOIMMUNE THYROIDITIS	1 (0.0)	INTESTINAL DILATATION	1 (0.0)
MUSCLE RIGIDITY	1 (0.0)	PAPULOPUSTULAR ROSACEA	1 (0.0)
EMBOLIC CEREBRAL INFARCTION	1 (0.0)	BUTTOCK INJURY	1 (0.0)
HEMIPLEGIA	1 (0.0)	ENCEPHALITIS HERPES	1 (0.0)
FUNCTIONAL GASTROINTESTINAL DISORDER	1 (0.0)	DISSEMINATED INTRAVASCULAR COAGULATION	1 (0.0)
SUTURE RUPTURE	1 (0.0)	DROP ATTACKS	1 (0.0)

NEUROMYOPATHY	1 (0.0)	INFLUENZA A VIRUS TEST POSITIVE	1 (0.0)
MITRAL VALVE PROLAPSE	1 (0.0)	PANCREATIC CARCINOMA STAGE IV	1 (0.0)
FOREIGN BODY IN GASTROINTESTINAL TRACT	1 (0.0)	MEDICATION ERROR	1 (0.0)
BLINDNESS TRANSIENT	1 (0.0)	SEMEN VISCOSITY ABNORMAL	1 (0.0)
DYSPHEMIA	1 (0.0)	ANORECTAL POLYP	1 (0.0)
VISUAL PROCESSING DISORDER	1 (0.0)	ORAL PAPULE	1 (0.0)
HAEMORRHAGIC DIATHESIS	1 (0.0)	DERMATITIS ALLERGIC	1 (0.0)
VARICOSE VEIN	1 (0.0)	HYPOPITUITARISM	1 (0.0)
AFFECTIVE DISORDER	1 (0.0)	HYPERPARATHYROIDISM	1 (0.0)
PERSONALITY CHANGE	1 (0.0)	MEDICAL DEVICE SITE GRANULOMA	1 (0.0)
PULMONARY SEPSIS	1 (0.0)	KL-6 INCREASED	1 (0.0)
SKIN BURNING SENSATION	1 (0.0)	BLOOD BETA-D-GLUCAN INCREASED	1 (0.0)
FOOD INTOLERANCE	1 (0.0)	METASTATIC MALIGNANT MELANOMA	1 (0.0)
DENTAL PROSTHESIS PLACEMENT	1 (0.0)	INCISION SITE ERYTHEMA	1 (0.0)

PARAINFLUENZAE VIRUS INFECTION	1 (0.0)	HIDRADENITIS	1 (0.0)
PERITONEAL DIALYSIS	1 (0.0)	BUNDLE BRANCH BLOCK RIGHT	1 (0.0)
NORMAL NEWBORN	1 (0.0)	HELICOBACTER GASTRITIS	1 (0.0)
MACULAR HOLE	1 (0.0)	METASTATIC CARCINOMA OF THE BLADDER	1 (0.0)
LARYNGEAL NEOPLASM	1 (0.0)	ENCEPHALITIS AUTOIMMUNE	1 (0.0)
THROAT CANCER	1 (0.0)	CYTOKINE STORM	1 (0.0)
JAUNDICE ACHOLURIC	1 (0.0)	TENSION HEADACHE	1 (0.0)
CARDIOVASCULAR SYMPTOM	1 (0.0)	HEAD DISCOMFORT	1 (0.0)
CALCULUS URETHRAL	1 (0.0)	FACIAL DISCOMFORT	1 (0.0)
URINARY TRACT OBSTRUCTION	1 (0.0)	SARCOIDOSIS	1 (0.0)
URINE CALCIUM INCREASED	1 (0.0)	CORONARY ARTERIAL STENT INSERTION	1 (0.0)
URINE PHOSPHORUS INCREASED	1 (0.0)	RESPIRATORY RATE DECREASED	1 (0.0)
DIVERTICULUM INTESTINAL	1 (0.0)	INFUSION RELATED REACTION	1 (0.0)
OBSTRUCTIV	1	FOREIGN	1 (0.0)

E NEPHROPATHY	(0.0)	BODY SENSATION IN EYES	
GOUT	1 (0.0)	OXYGEN SATURATION ABNORMAL	1 (0.0)
ABDOMINAL RIGIDITY	1 (0.0)	HYPOAESTHESIA ORAL	1 (0.0)
TYMPANOPLASTY	1 (0.0)	MENIERE'S DISEASE	1 (0.0)
PERIPHERAL VASCULAR DISORDER	1 (0.0)	ADVERSE REACTION	1 (0.0)
GASTROOESOPHAGEAL SPHINCTER INSUFFICIENCY	1 (0.0)	UMBILICAL HERNIA	1 (0.0)
COCCYDYNIA	1 (0.0)	PARATHYROID TUMOUR	1 (0.0)
ANAL FISTULA INFECTION	1 (0.0)	SUBDURAL EFFUSION	1 (0.0)
ENDOMETRIAL ADENOCARCINOMA	1 (0.0)	BLINDNESS TRANSIENT	1 (0.0)
DISSEMINATED MYCOBACTERIUM AVIUM COMPLEX INFECTION	1 (0.0)	PRURITUS GENITAL	1 (0.0)
ANAL STENOSIS	1 (0.0)	THORACIC OPERATION	1 (0.0)
PNEUMONIA ESCHERICHIA	1 (0.0)	ORAL DISORDER	1 (0.0)
ILEOCOLOSTOMY	1 (0.0)	ABDOMINAL LYMPHADENOPATHY	1 (0.0)
B-CELL LYMPHOMA	1 (0.0)	SICKLE CELL ANAEMIA	1 (0.0)

POST PROCEDURAL PULMONARY EMBOLISM	1 (0.0)	PHARYNGEAL POLYP	1 (0.0)
MENISCUS OPERATION	1 (0.0)	POOR PERIPHERAL CIRCULATION	1 (0.0)
INCREASED APPETITE	1 (0.0)	WHEELCHAIR USER	1 (0.0)
PORTAL VEIN EMBOLISM	1 (0.0)	APPETITE DISORDER	1 (0.0)
PREMATURE RUPTURE OF MEMBRANES	1 (0.0)	SPINAL DECOMPRESSION	1 (0.0)
FACE OEDEMA	1 (0.0)	ABNORMAL LOSS OF WEIGHT	1 (0.0)
PREMATURE LABOUR	1 (0.0)	GASTRIC NEOPLASM	1 (0.0)
NOROVIRUS TEST POSITIVE	1 (0.0)	HORMONE LEVEL ABNORMAL	1 (0.0)
YERSINIA TEST POSITIVE	1 (0.0)	ANXIETY DISORDER	1 (0.0)
INFLAMMATORY MARKER DECREASED	1 (0.0)	ENDOCRINE DISORDER	1 (0.0)
EAR NEOPLASM	1 (0.0)	BREAST CYST	1 (0.0)
FACIAL DISCOMFORT	1 (0.0)	VAGINAL CYST	1 (0.0)
THORACIC HAEMORRHAGE	1 (0.0)	ASPIRATION	1 (0.0)
CARDIAC ANEURYSM	1 (0.0)	BREAST PROCEDURAL COMPLICATI	1 (0.0)

		ON	
ECTOPIC PREGNANCY	1 (0.0)	FLANK PAIN	1 (0.0)
INFLUENZA A VIRUS TEST POSITIVE	1 (0.0)	RENAL PAIN	1 (0.0)
SPINAL DECOMPRESSION	1 (0.0)	YELLOW SKIN	1 (0.0)
LUMBAR RADICULOPATHY	1 (0.0)	COELIAC ARTERY COMPRESSION SYNDROME	1 (0.0)
CAUDA EQUINA SYNDROME	1 (0.0)	HAEMORRHAGIC STROKE	1 (0.0)
MUSCLE ENZYME INCREASED	1 (0.0)	NEUTROPHIL COUNT ABNORMAL	1 (0.0)
CERVICAL INCOMPETENCE	1 (0.0)	FAECES SOFT	1 (0.0)
PARENTERAL NUTRITION	1 (0.0)	BILIARY COLIC	1 (0.0)
CHRONIC GASTRITIS	1 (0.0)	FOOD INTOLERANCE	1 (0.0)
INCOHERENT	1 (0.0)	BLADDER DISCOMFORT	1 (0.0)
BIOCHEMICAL PREGNANCY	1 (0.0)	CHOLESTASIS	1 (0.0)
FRACTURE PAIN	1 (0.0)	NORMOCHROMIC NORMOCYTIC ANAEMIA	1 (0.0)
ANKLE ARTHROPLASTY	1 (0.0)	IMMUNE THROMBOCYTOPENIA	1 (0.0)
MONOPLEGIA	1 (0.0)	BILE DUCT STENT INSERTION	1 (0.0)

ROTATOR CUFF SYNDROME	1 (0.0)	GANGRENE	1 (0.0)
PROTEIN TOTAL DECREASED	1 (0.0)	BETA HAEMOLYTIC STREPTOCOCCAL INFECTION	1 (0.0)
PANCREATIC DISORDER	1 (0.0)	GAMMA-GLUTAMYLTRANSFERASE INCREASED	1 (0.0)
DISTURBANCE IN SOCIAL BEHAVIOUR	1 (0.0)	HEPATIC INFECTION	1 (0.0)
FAT NECROSIS	1 (0.0)	CARBOHYDRATE ANTIGEN 19-9 INCREASED	1 (0.0)
SINUS ARRHYTHMIA	1 (0.0)	HEPATIC FIBROSIS	1 (0.0)
INVASIVE LOBULAR BREAST CARCINOMA	1 (0.0)	BILIRUBIN CONJUGATED INCREASED	1 (0.0)
BREAST DISCOMFORT	1 (0.0)	EXTRASYSTOLES	1 (0.0)
BUNDLE BRANCH BLOCK RIGHT	1 (0.0)	BREAST DISORDER	1 (0.0)
BLOOD CREATININE DECREASED	1 (0.0)	GASTRIC BYPASS	1 (0.0)
GUT DERIVED INFECTION	1 (0.0)	URETERIC OBSTRUCTION	1 (0.0)
MEDICAL DEVICE	1 (0.0)	MULTIPLE FRACTURES	1 (0.0)

CHANGE			
COLOSTOMY BAG USER	1 (0.0)	EMOTIONAL POVERTY	1 (0.0)
GASTRITIS BACTERIAL	1 (0.0)	CEREBRAL ISCHAEMIA	1 (0.0)
HYPERTONIC BLADDER	1 (0.0)	WRIST SURGERY	1 (0.0)
BENIGN PROSTATIC HYPERPLASIA	1 (0.0)	IMMATURE PLATELET FRACTION INCREASED	1 (0.0)
INTESTINAL POLYP	1 (0.0)	ONYCHOLYSIS	1 (0.0)
GASTROINTESTINAL CARCINOMA	1 (0.0)	PITYRIASIS ROSEA	1 (0.0)
HAEMORRHAGIC CYST	1 (0.0)	EXPOSURE TO RADIATION	1 (0.0)
ARTERIAL DISORDER	1 (0.0)	OCCUPATIONAL EXPOSURE TO TOXIC AGENT	1 (0.0)
VISUAL FIELD DEFECT	1 (0.0)	EXPOSURE TO TOXIC AGENT	1 (0.0)
ANASTOMOTIC FISTULA	1 (0.0)	OCCUPATIONAL EXPOSURE TO SUNLIGHT	1 (0.0)
RENAL TUBULAR NECROSIS	1 (0.0)	GIANT CELL ARTERITIS	1 (0.0)
TRANSURETHRAL PROSTATECTOMY	1 (0.0)	SCLERITIS	1 (0.0)
OCULAR STROKE	1 (0.0)	FOLLICULAR DISORDER	1 (0.0)
OCULAR PROCEDURAL	1 (0.0)	HAEMOLYTIC ANAEMIA	1 (0.0)

COMPLICATION			
INTRAOCULAR PRESSURE TEST ABNORMAL	1 (0.0)	NON-CIRRHOTIC PORTAL HYPERTENSION	1 (0.0)
PELVIC ABSCESS	1 (0.0)	ABDOMINOPLASTY	1 (0.0)
EYE INFECTION STAPHYLOCOCCAL	1 (0.0)	POLYPECTOMY	1 (0.0)
ECCHYMOSIS	1 (0.0)	PORTAL HYPERTENSION	1 (0.0)
DRUG ERUPTION	1 (0.0)	GASTROENTERITIS NOROVIRUS	1 (0.0)
ACUTE MYELOID LEUKAEMIA	1 (0.0)	EOSINOPHILIC OESOPHAGITIS	1 (0.0)
HERPES SIMPLEX HEPATITIS	1 (0.0)	ACQUIRED OESOPHAGEAL WEB	1 (0.0)
NEUROENDOCRINE TUMOUR	1 (0.0)	RECTOCELE	1 (0.0)
BLOOD IMMUNOGLOBULIN E INCREASED	1 (0.0)	PELVIC FLOOR DYSFUNCTION	1 (0.0)
HYPERTHERMIA	1 (0.0)	CHRONIC GASTRITIS	1 (0.0)
EOSINOPHILIC OESOPHAGITIS	1 (0.0)	HYPERCOAGULATION	1 (0.0)
MALIGNANT POLYP	1 (0.0)	RADIATION INJURY	1 (0.0)
BLOOD IMMUNOGLOBULIN E	1 (0.0)	PRODUCT LOT NUMBER	1 (0.0)

ABNORMAL		ISSUE	
PEPTIC ULCER HELICOBACTER	1 (0.0)	PARATHYROID TUMOUR BENIGN	1 (0.0)
HELICOBACTER INFECTION	1 (0.0)	CYTOMEGALOVIRUS TEST POSITIVE	1 (0.0)
HAEMATOLOGICAL INFECTION	1 (0.0)	URINARY TRACT OBSTRUCTION	1 (0.0)
EYE INFARCTION	1 (0.0)	UTERINE ARTERY EMBOLISATION	1 (0.0)
LACUNAR STROKE	1 (0.0)	MITRAL VALVE PROLAPSE	1 (0.0)
STRESS AT WORK	1 (0.0)	GENITAL LESION	1 (0.0)
GASTROINTESTINAL ANASTOMOTIC STENOSIS	1 (0.0)	WEIGHT LOSS POOR	1 (0.0)
NEUROLOGICAL SYMPTOM	1 (0.0)	PRODUCT QUALITY ISSUE	1 (0.0)
NONINFECTIVE ENCEPHALITIS	1 (0.0)	EXTRANODAL MARGINAL ZONE B-CELL LYMPHOMA (MALT TYPE)	1 (0.0)
PERIPHERAL ARTERY THROMBOSIS	1 (0.0)	LACUNAR STROKE	1 (0.0)
ABSCESS DRAINAGE	1 (0.0)	GLUCOSE-6-PHOSPHATE DEHYDROGENASE DEFICIENCY	1 (0.0)

RUBBER SENSITIVITY	1 (0.0)	ECCHYMOSIS	1 (0.0)
HEPATITIS ACUTE	1 (0.0)	GASTROINTESTINAL WALL THINNING	1 (0.0)
PARVOVIRUS INFECTION	1 (0.0)	BLOOD PRESSURE SYSTOLIC ABNORMAL	1 (0.0)
LUNG ADENOCARCINOMA	1 (0.0)	FAECALOMA	1 (0.0)
BODY TEMPERATURE ABNORMAL	1 (0.0)	INTERVERTEBRAL DISC DISORDER	1 (0.0)
TUMOUR PAIN	1 (0.0)	SKIN WEEPING	1 (0.0)
ENDOMETRIAL NEOPLASM	1 (0.0)	DIARRHOEA INFECTIOUS	1 (0.0)
RENAL INJURY	1 (0.0)	AORTIC ANEURYSM	1 (0.0)
TOOTH IMPACTED	1 (0.0)	CERVICAL INCOMPETENCE	1 (0.0)
COELIAC DISEASE	1 (0.0)	PREMATURE RUPTURE OF MEMBRANES	1 (0.0)
MUSCLE SWELLING	1 (0.0)	RENAL CELL CARCINOMA	1 (0.0)
AXILLARY MASS	1 (0.0)	AORTIC STENOSIS	1 (0.0)
OOPHORITIS	1 (0.0)	ANOSMIA	1 (0.0)
WOUND SEPSIS	1 (0.0)	SUSPECTED COVID-19	1 (0.0)
DISSEMINATED INTRAVASCULAR	1 (0.0)	ACUTE HAEMORRHAGIC ULCERATIVE	1 (0.0)

COAGULATION		COLITIS	
ISCHAEMIA	1 (0.0)	ELBOW OPERATION	1 (0.0)
ABORTION SPONTANEOUS	1 (0.0)	HUMERUS FRACTURE	1 (0.0)
THERAPY NON-RESPONDER	1 (0.0)	BARTHOLINITIS	1 (0.0)
HEPATOSPLENOMEGALY	1 (0.0)	CYTOMEGALOVIRUS VIRAEMIA	1 (0.0)
PYURIA	1 (0.0)	PNEUMOCYSTIS TEST POSITIVE	1 (0.0)
THROMBOCYTOSIS	1 (0.0)	EXERCISE LACK OF	1 (0.0)
GASTROINTESTINAL MOTILITY DISORDER	1 (0.0)	WEIGHT BEARING DIFFICULTY	1 (0.0)
BILE DUCT STONE	1 (0.0)	ANTIBODY TEST ABNORMAL	1 (0.0)
PRODUCT TASTE ABNORMAL	1 (0.0)	BLOOD SODIUM ABNORMAL	1 (0.0)
SUBDURAL ABSCESS	1 (0.0)	ANAL FISSURE HAEMORRHAGE	1 (0.0)
STUPOR	1 (0.0)	FEMALE GENITAL TRACT FISTULA	1 (0.0)
PHOTOSENSITIVITY REACTION	1 (0.0)	IMMUNOGLOBULINS DECREASED	1 (0.0)
SKIN FISSURES	1 (0.0)	GLOMERULAR FILTRATION RATE ABNORMAL	1 (0.0)

NEPHROURETERECTOMY	1 (0.0)	ANTI FACTOR XA ACTIVITY DECREASED	1 (0.0)
CHONDROPATHY	1 (0.0)	BODY TEMPERATURE DECREASED	1 (0.0)
ATTENTION DEFICIT HYPERACTIVITY DISORDER	1 (0.0)	TEMPERATURE INTOLERANCE	1 (0.0)
DENTAL IMPLANTATION	1 (0.0)	HEART VALVE INCOMPETENCE	1 (0.0)
MYASTHENIA GRAVIS	1 (0.0)	ARTERIOSCLEROSIS CORONARY ARTERY	1 (0.0)
GINGIVAL PAIN	1 (0.0)	TROPONIN T INCREASED	1 (0.0)
BLOOD THYROID STIMULATING HORMONE INCREASED	1 (0.0)	HAEMODYNAMIC INSTABILITY	1 (0.0)
PERIPHERAL NERVE DECOMPRESSION	1 (0.0)	MYOGLOBIN BLOOD INCREASED	1 (0.0)
ORGAN FAILURE	1 (0.0)	RADIATION ASSOCIATED PAIN	1 (0.0)
LAPAROSCOPIC SURGERY	1 (0.0)	HEPATITIS FULMINANT	1 (0.0)
OVERGROWTH FUNGAL	1 (0.0)	CYST RUPTURE	1 (0.0)
PRODUCT CONTAMINATION	1 (0.0)	HYPERPYREXIA	1 (0.0)
CARDIAC	1	CORTICAL	1 (0.0)

INFECTION	(0.0)	HAND	
		STROKE	
		CARDIAC VENTRICULAR THROMBOSIS	1 (0.0)
		POST PROCEDURAL CONSTIPATION	1 (0.0)
		NASAL OPERATION	1 (0.0)
		MILLER FISHER SYNDROME	1 (0.0)
		JOINT NOISE	1 (0.0)
		ANORECTAL STENOSIS	1 (0.0)
		EXOPHTHALMOS	1 (0.0)
		INCISION SITE ABSCESS	1 (0.0)
		INCISIONAL HERNIA	1 (0.0)
		INCISION SITE HAEMORRHAGE	1 (0.0)
		NAIL INFECTION	1 (0.0)
		RECTAL SPASM	1 (0.0)
		HERPES ZOSTER INFECTION NEUROLOGICAL	1 (0.0)
		PHARYNGEAL PERFORATION	1 (0.0)

TONGUE NEOPLASM MALIGNANT STAGE UNSPECIFIED	1 (0.0)
TRICHORRHEXIS	1 (0.0)
EXTRADURAL ABSCESS	1 (0.0)
PHYSICAL DECONDITIONING	1 (0.0)
STAPHYLOCOCCAL BACTERAEMIA	1 (0.0)
BONE LOSS	1 (0.0)
SEPTIC ARTHRITIS STAPHYLOCOCCAL	1 (0.0)
SJOGREN'S SYNDROME	1 (0.0)
DENTAL CARIES	1 (0.0)
BLOOD PH INCREASED	1 (0.0)
PCO2 ABNORMAL	1 (0.0)
DIVERTICULITIS INTESTINAL PERFORATED	1 (0.0)
NEUTROPHIL COUNT INCREASED	1 (0.0)
CERVICAL VERTEBRAL FRACTURE	1 (0.0)
SERUM FERRITIN INCREASED	1 (0.0)

BLOOD IRON INCREASED	1 (0.0)
HEPATOMEGALY	1 (0.0)
RETICULOCYTE COUNT DECREASED	1 (0.0)
HYPERTHERMIA MALIGNANT	1 (0.0)
SMALL INTESTINAL PERFORATION	1 (0.0)
ELECTROLYTE IMBALANCE	1 (0.0)
NORMOCYTIC ANAEMIA	1 (0.0)
REFEEDING SYNDROME	1 (0.0)
VULVOVAGINAL CANDIDIASIS	1 (0.0)
HYPOMAGNESAEMIA	1 (0.0)
DELIRIUM	1 (0.0)
T-CELL LYMPHOMA	1 (0.0)
BLOOD THYROID STIMULATING HORMONE INCREASED	1 (0.0)
DRUG MONITORING PROCEDURE NOT PERFORMED	1 (0.0)
BREAST CANCER MALE	1 (0.0)

SCROTAL ABSCESS	1 (0.0)
BLOOD CALCIUM INCREASED	1 (0.0)
NECK MASS	1 (0.0)
LIMB DEFORMITY	1 (0.0)
DERMATITIS	1 (0.0)
BACTERIAL COLITIS	1 (0.0)
BILE DUCT STENT REMOVAL	1 (0.0)
RECTAL ULCER HAEMORRHAGE	1 (0.0)
BILE ACID MALABSORPTION	1 (0.0)
DEPRESSIVE SYMPTOM	1 (0.0)
FAECAL CALPROTECTIN ABNORMAL	1 (0.0)
EYE MOVEMENT DISORDER	1 (0.0)
CELLULITIS ORBITAL	1 (0.0)
RECTAL OBSTRUCTION	1 (0.0)
SEROSITIS	1 (0.0)
PNEUMONIA STAPHYLOCOCCAL	1 (0.0)
PNEUMONIA INFLUENZAL	1 (0.0)
LIGAMENT PAIN	1 (0.0)
COLONIC	1 (0.0)

ABSCESS	
DIVERTICULUM INTESTINAL HAEMORRHAGIC	1 (0.0)
ENERGY INCREASED	1 (0.0)
LEUKAEMIA	1 (0.0)
RIGHT VENTRICULAR SYSTOLIC PRESSURE INCREASED	1 (0.0)
THYROID CALCIFICATION	1 (0.0)
ARTERIOVENOUS MALFORMATION	1 (0.0)
ORTHOPNOEA	1 (0.0)
CHRONIC RESPIRATORY FAILURE	1 (0.0)
PICKWICKIAN SYNDROME	1 (0.0)
MENINGITIS TUBERCULOUS	1 (0.0)
ATRIAL SEPTAL DEFECT	1 (0.0)
INTESTINAL MALROTATION	1 (0.0)
CYTOMEGALOVIRUS CHORIORETINITIS	1 (0.0)
PELVIC VENOUS	1 (0.0)

THROMBOSIS	
PERIPHERAL VEIN OCCLUSION	1 (0.0)
VENA CAVA THROMBOSIS	1 (0.0)
STOMA COMPLICATION	1 (0.0)
URINARY TRACT DISCOMFORT	1 (0.0)
BIPOLAR DISORDER	1 (0.0)
MANIA	1 (0.0)
BLADDER DIVERTICULUM	1 (0.0)
PULMONARY IMAGING PROCEDURE ABNORMAL	1 (0.0)
GALLBLADDER OEDEMA	1 (0.0)
CENTRAL NERVOUS SYSTEM VASCULITIS	1 (0.0)
FACIAL OPERATION	1 (0.0)
NEUROENDOCRINE TUMOUR	1 (0.0)
CARCINOID TUMOUR	1 (0.0)
KAPOSI'S SARCOMA	1 (0.0)
HUNGER	1 (0.0)
PLATELET TRANSFUSION	1 (0.0)

EAR NEOPLASM	1 (0.0)
ARTERIAL RUPTURE	1 (0.0)
VEIN RUPTURE	1 (0.0)
PARONYCHIA	1 (0.0)
ENTEROVESICAL FISTULA	1 (0.0)
PSEUDOPOLYPOSIS	1 (0.0)
FACTOR V LEIDEN MUTATION	1 (0.0)
ESCHERICHIA URINARY TRACT INFECTION	1 (0.0)
CEREBRAL VENOUS THROMBOSIS	1 (0.0)
PRODUCT COLOUR ISSUE	1 (0.0)
TOOTH DISCOLOURATION	1 (0.0)
HUMAN PAPILLOMA VIRUS REACTIVATION	1 (0.0)
LOCAL REACTION	1 (0.0)
SNAKE BITE	1 (0.0)
VASCULAR STENT THROMBOSIS	1 (0.0)
VERTEBROBASILAR STROKE	1 (0.0)

VISUAL FIELD DEFECT	1 (0.0)
PROLONGED LABOUR	1 (0.0)
